# Supplementary material for: Gene dosage effects of 22q11.2 copy number variants on in-vivo measures of white matter axonal density and dispersion
Source: Mol Psychiatry. 2026 Feb 20;31(7):3687–98. doi: 10.1038/s41380-026-03489-4 (PMC13268960; doi:10.1038/s41380-026-03489-4)
Supplement: Supplementary file 1 — Supplementary Information - Content [file 41380_2026_3489_MOESM1_ESM.docx]

***Content in supplementary information:***

A description of the quality assessment of diffusion magnetic resonance images and sensitivity analyses, including figures showing point estimates of group differences and tables presenting baseline characteristics of the sample included in the study. Supplementary tables show model summaries of group differences and age effects.
