# Supplementary material for: Gene dosage effects of 22q11.2 copy number variants on in-vivo measures of white matter axonal density and dispersion
Source: Mol Psychiatry. 2026 Feb 20;31(7):3687–98. doi: 10.1038/s41380-026-03489-4 (PMC13268960; doi:10.1038/s41380-026-03489-4)
Supplement: Supplementary file 2 — Supplementary Information [file 41380_2026_3489_MOESM2_ESM.docx]

Supplementary Information

**Supplementary note 1. Participants and quality assessment of diffusion magnetic resonance images.**

Information on study procedures, inclusion/exclusion criteria, and ascertainment criteria are detailed in(1–3); Briefly, 22qDel and 22qDup carriers were recruited nationwide from clinics, national support groups, and other online avenues while control participants were recruited through online postings and flyers posted throughout the community. Participants with significant neurological or medical conditions (unrelated to 22q11.2 CNVs) affecting brain structure or function, previous head trauma with loss of consciousness, insufficient English fluency, and/or substance abuse/dependence within the past 6 months were excluded. All participants gave verbal and written informed consent to participate in the study. Participants under 18 years of age provided written assent and their parent/guardian provided written consent. All study procedures and documents conform to the standards of the Declaration of Helsinki and were approved by the University of California Los Angeles (UCLA) and Stanford University Institutional Review Boards. All diffusion MRI (dMRI) and T1-weighted (T1w) data from the 22q11.2 CNV dataset were visually inspected and images with excessive artifacts were excluded from downstream analyses (i.e., scans from 18 participants due to head motion), yielding a total of 151 scans for pre-processing across 109 participants. After the completion of the pre-processing pipeline, we further excluded participants using a statistical approach to exclude data of insufficient quality. Here, we extracted out the squared voxel displacement across all intracerebral voxels from the EDDY outputs and averaged these values across all volumes to obtain an index of image quality and excluded individuals with values higher than 3 standard deviations from the mean of the participants derived from the same scanner site. This resulted in exclusion of two scans, yielding a total of 149 scans across 109 participants. Kruskal-Wallis rank sum test did not reveal any statistically significant group difference in image quality between 22qDel, 22Dup and controls *H*(2) =5.08, p = .08). There was no significant correlation between image quality and baseline age, r(107) = -.01, 95 CI% = -.19, .19, p = .99).

**Supplementary note 2. Sensitivity analyses**

To test the robustness of our results, we ran several sensitivity analyses using data derived from the same scanner site across 22q11.2 CNV carriers and controls. That is, only scans from UCLA and Stanford for 22qDel vs controls, and only scans derived from UCLA for the remaining comparisons (including gene dosage analyses). Thus, site (i.e., UCLA and Stanford) was included as a fixed covariate only for the 22qDel vs control comparisons. We derived cerebrospinal fluid volume (CSF), white matter volume (WMvol) and estimated intracranial volume (ICV) using the recon-all function from FreeSurfer v.6.0.0. We ran four different models using GAMM, including models adjusting for: 1) age and sex, 2) age, sex, and CSF, 3) age, sex, and white matter volume (WMvol), 4) age, sex, and ICV on data derived from the same scanner site.

References

1. Jalbrzikowski M, Lin A, Vajdi A, Grigoryan V, Kushan L, Ching CRK, et al. Longitudinal trajectories of cortical development in 22q11.2 copy number variants and typically developing controls. Mol Psychiatry. 2022 Oct;27(10):4181–90.

2. Seitz-Holland J, Lyons M, Kushan L, Lin A, Villalon-Reina JE, Cho KIK, et al. Opposing white matter microstructure abnormalities in 22q11.2 deletion and duplication carriers. Transl Psychiatry. 2021 Nov 10;11(1):1–11.

3. Jalbrzikowski M, Jonas R, Senturk D, Patel A, Chow C, Green MF, et al. Structural abnormalities in cortical volume, thickness, and surface area in 22q11.2 microdeletion syndrome: Relationship with psychotic symptoms. NeuroImage Clin. 2013 Oct 14;3:405–15.

sTable 1. Baseline demographic characteristics for UCLA and Stanford participants

|  | 22qDeletion | | 22qDuplication | Control | | Overall | |
| --- | --- | --- | --- | --- | --- | --- | --- |
|  | **Stanford (N=6)** | **UCLA (N=44)** | **UCLA (N=24)** | **Stanford (N=7)** | **UCLA (N=28)** | **Stanford (N=13)** | **UCLA (N=96)** |
| Age |  |  |  |  |  |  |  |
| Mean (SD) | 30.7 (7.22) | 19.4 (8.86) | 21.6 (14.1) | 29.8 (5.41) | 19.0 (8.88) | 30.2 (6.05) | 19.8 (10.4) |
| Median [Min, Max] | 27.9 [22.5, 40.3] | 19.8 [7.40, 51.1] | 14.5 [8.33, 49.4] | 31.8 [21.6, 36.9] | 17.9 [7.81, 45.3] | 28.6 [21.6, 40.3] | 16.7 [7.40, 51.1] |
| Sex |  |  |  |  |  |  |  |
| Female | 3 (50.0%) | 29 (65.9%) | 13 (54.2%) | 3 (42.9%) | 19 (67.9%) | 6 (46.2%) | 61 (63.5%) |
| Male | 3 (50.0%) | 15 (34.1%) | 11 (45.8%) | 4 (57.1%) | 9 (32.1%) | 7 (53.8%) | 35 (36.5%) |

*Notes.* 7 controls, 11 22q11.2 deletion carriers, and 10 22q11.2 duplication carriers had two scans; 2 controls and 4 22q11.2 deletion carriers had three scans.

sTable 2. Baseline characteristics for Human Connectome Project control participants

|  | HCP1 (N=212) | HCP2 (N=190) | HCP3 (N=57) | HCP4 (N=84) | HCP5 (N=199) | HCP6 (N=16) | HCP7 (N=97) | Overall (N=855) |
| --- | --- | --- | --- | --- | --- | --- | --- | --- |
| Age |  |  |  |  |  |  |  |  |
| Mean (SD) | 22.2 (14.0) | 21.8 (13.7) | 42.8 (4.56) | 19.9 (12.3) | 13.9 (3.84) | 23.1 (14.0) | 27.5 (14.9) | 21.9 (13.6) |
| Median [Min, Max] | 14.7 [8.08, 51.1] | 15.3 [8.08, 51.1] | 41.3 [36.6, 50.8] | 14.8 [8.00, 48.4] | 13.5 [8.08, 21.8] | 16.9 [8.08, 47.3] | 20.8 [8.00, 49.8] | 15.3 [8.00, 51.1] |
| Sex |  |  |  |  |  |  |  |  |
| Female | 119 (56.1%) | 102 (53.7%) | 33 (57.9%) | 48 (57.1%) | 105 (52.8%) | 10 (62.5%) | 46 (47.4%) | 463  (54.2%) |
| Male | 93 (43.9%) | 88 (46.3%) | 24 (42.1%) | 36 (42.9%) | 94 (47.2%) | 6 (37.5%) | 51 (52.6%) | 392  (45.8%) |

sTable 3. Number of scans across all participants across all sites

| Age Group | 22qDel | 22qDup | Control |
| --- | --- | --- | --- |
| 7-13 | 18 | 11 | 294 |
| 13-19 | 9 | 11 | 261 |
| 19-25 | 24 | 2 | 96 |
| 25-31 | 12 | 0 | 6 |
| 31-37 | 1 | 3 | 23 |
| >37 | 5 | 7 | 221 |

*Notes.* Binned age groups in steps of six. Sample size (n scans) across age groups by across 22q11.2 deletion, 22q11.2 duplication, and control participants.


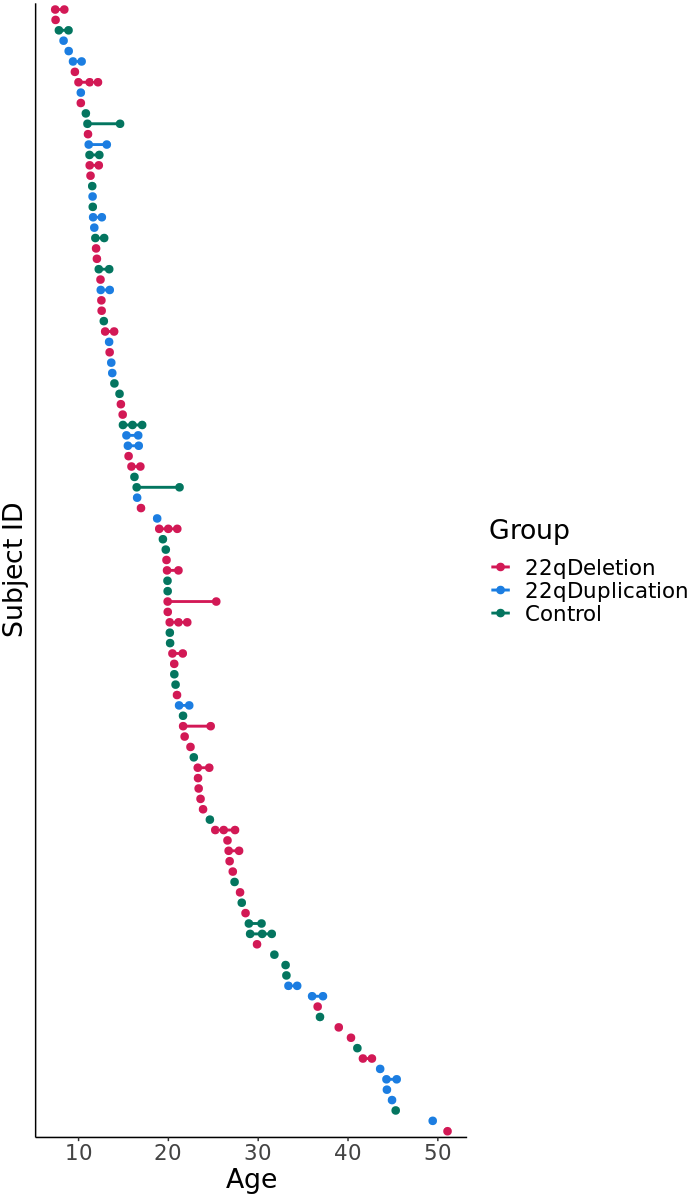

sFigure 1. Participant age at each scan for the participants from the UCLA and Stanford cohorts.
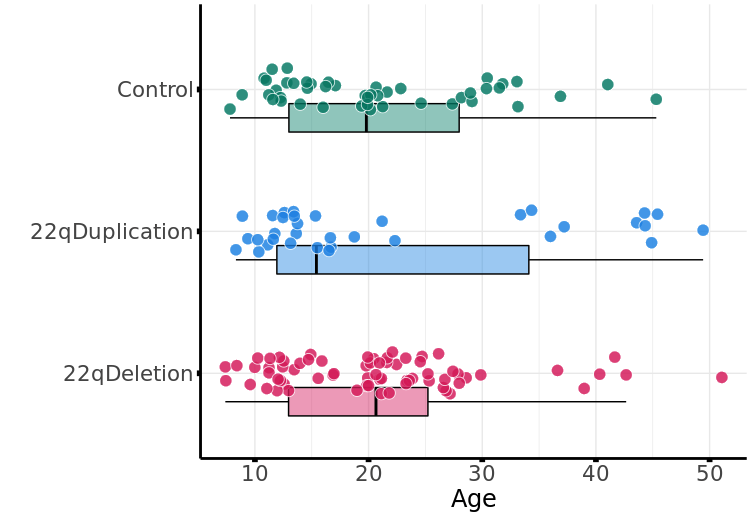


sFigure 2. Age distribution for analyses comparing 22q11.2 deletion, 22q11.2 duplication, and controls across the UCLA and Stanford cohorts. Kruskal-Wallis rank sum test indicated no statistically significant group difference in age between groups at baseline H(2) =1.24, p = .54).


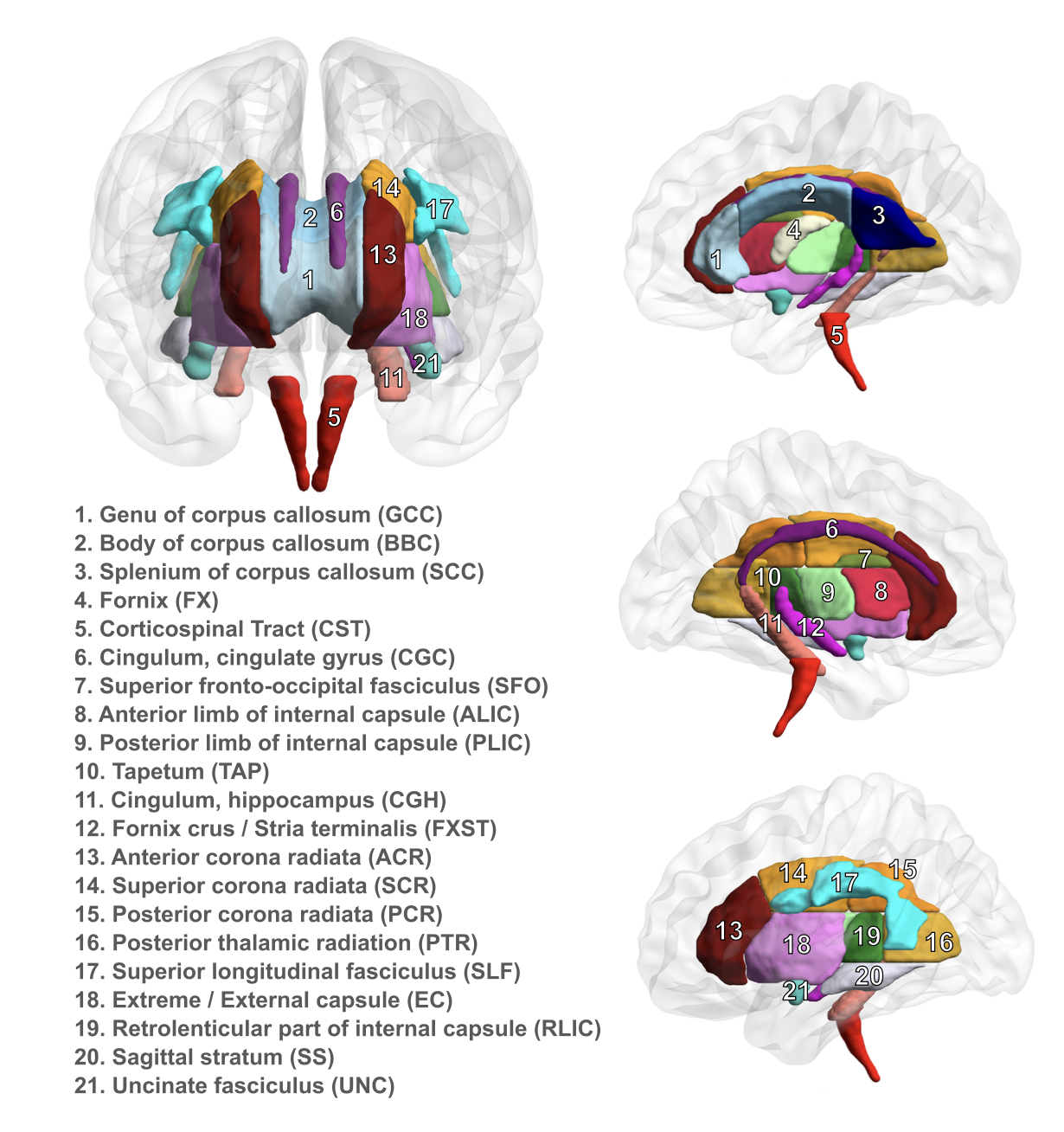


**sFigure 3.** Brain map of the region of interest derived from the John Hopkins University white matter atlas. In addition, measures from the full corpus callosum (CC), internal capsule (IC), corona radiata (CR) were obtained for the current study.

**sFigure 4.** Point estimates of group differences in intracellular volume fraction (ICVF) using individuals derived from the same scanner site only (green), with either white matter volume (WMvol, grey), intracranial volume (ICV, yellow), or cerebrospinal fluid volume (CSFvol, brown) included as covariates. Controls are used as the reference group when compared to 22qDel and 22qDup carriers. 22qDel carriers are used as the reference group when compared to 22qDup carriers.

**sFigure 5.** Point estimates of group differences in orientation dispersion index (ODI) using individuals derived from the same scanner site only (green) with either white matter volume (WMvol, grey), intracranial volume (ICV, yellow), or cerebrospinal fluid volume (CSFvol, brown) included as covariates. Controls are used as the reference group when compared to 22qDel and 22qDup carriers. 22qDel carriers are used as the reference group when compared to 22qDup carriers.

**sFigure 6.** Point estimates of group differences in isotropic volume fraction (ISO) using individuals derived from the same scanner site only (green) with either white matter volume (WMvol, grey), intracranial volume (ICV, yellow), or cerebrospinal fluid volume (CSFvol, brown) included as covariates. Controls are used as the reference group when compared to 22qDel and 22qDup carriers. 22qDel carriers are used as the reference group when compared to 22qDup carriers.


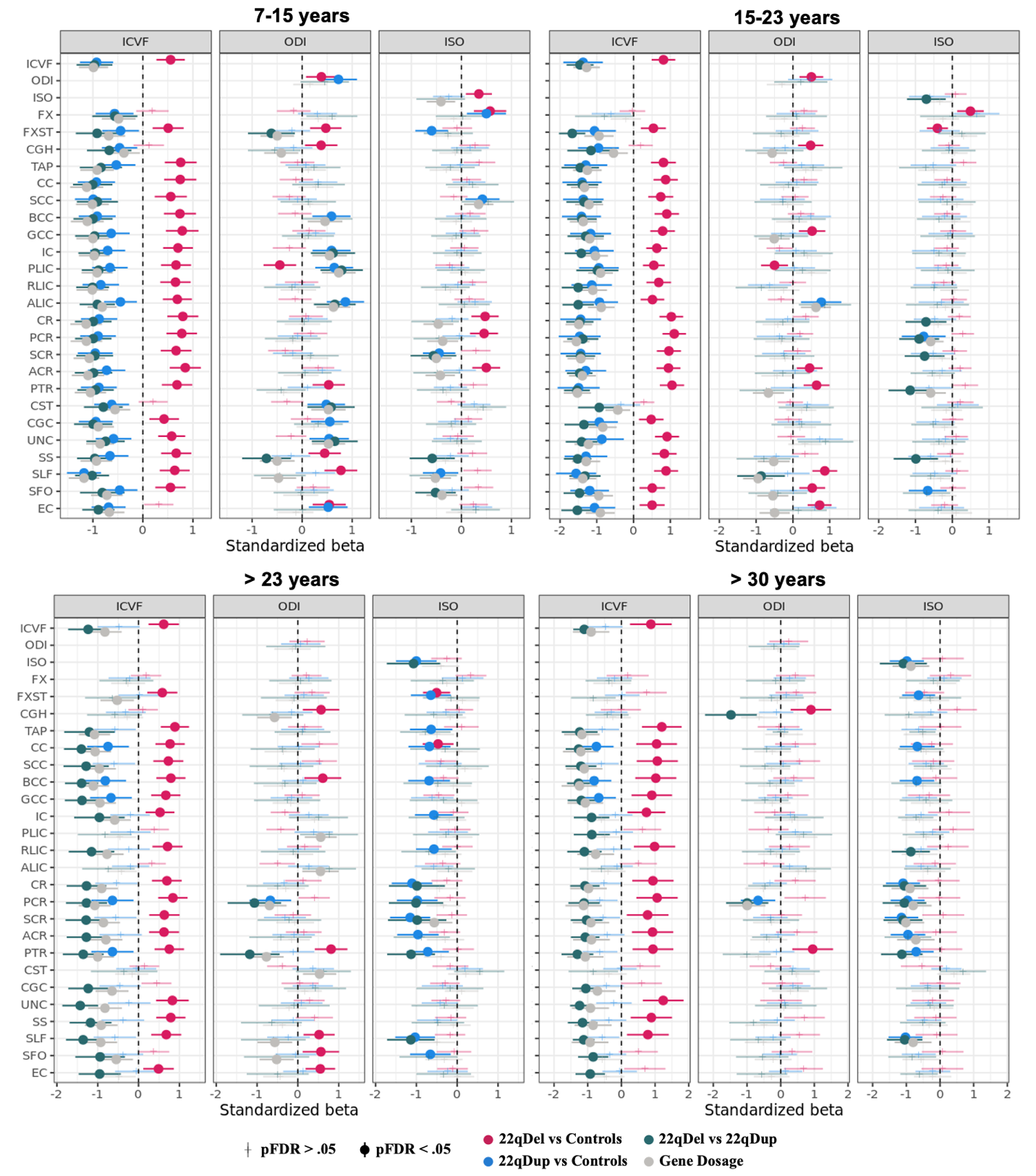


**sFigure 7.** Point estimates of group differences in global and regional intracellular volume fraction (ICVF), orientation dispersion index (ODI), isotropic volume fraction (ISO) across age groups: 7-15years (top left, n scans; control = 435, 22qDel = 22, 22qDup = 16), 15-23 years (top right, n scans; control = 215, 22qDel = 22, 22qDup = 8), >23 years (bottom left, n scans; control = 251, 22qDel = 25, 22qDup = 10), > 30 years (bottom right, n scans; control = 246, 22qDel = 6, 22qDup = 10).

| sTable 4. Main Results - Group differences across 22q11.2 CNV carriers and controls | | | | | | |
| --- | --- | --- | --- | --- | --- | --- |
| Dependent variable | **Analysis** | **Std.B.** | **SE** | **T-value** | **P-value** | **pFDR** |
| ICVF.combat | Control (Ref) vs 22q11.2Deletion | 0.496 | 0.071 | 6.954 | 0.000 | 0.000 |
| ISO.combat | Control (Ref) vs 22q11.2Deletion | 0.068 | 0.055 | 1.233 | 0.218 | 0.280 |
| ODI.combat | Control (Ref) vs 22q11.2Deletion | 0.247 | 0.066 | 3.741 | 0.000 | 0.000 |
| ICVF.ACR.combat | Control (Ref) vs 22q11.2Deletion | 0.709 | 0.084 | 8.416 | 0.000 | 0.000 |
| ICVF.ALIC.combat | Control (Ref) vs 22q11.2Deletion | 0.345 | 0.058 | 5.935 | 0.000 | 0.000 |
| ICVF.BCC.combat | Control (Ref) vs 22q11.2Deletion | 0.745 | 0.094 | 7.966 | 0.000 | 0.000 |
| ICVF.CC.combat | Control (Ref) vs 22q11.2Deletion | 0.728 | 0.094 | 7.788 | 0.000 | 0.000 |
| ICVF.CGC.combat | Control (Ref) vs 22q11.2Deletion | 0.371 | 0.083 | 4.488 | 0.000 | 0.000 |
| ICVF.CGH.combat | Control (Ref) vs 22q11.2Deletion | 0.118 | 0.074 | 1.605 | 0.109 | 0.156 |
| ICVF.CR.combat | Control (Ref) vs 22q11.2Deletion | 0.716 | 0.084 | 8.556 | 0.000 | 0.000 |
| ICVF.CST.combat | Control (Ref) vs 22q11.2Deletion | 0.193 | 0.085 | 2.270 | 0.023 | 0.038 |
| ICVF.EC.combat | Control (Ref) vs 22q11.2Deletion | 0.357 | 0.075 | 4.756 | 0.000 | 0.000 |
| ICVF.FX.combat | Control (Ref) vs 22q11.2Deletion | 0.130 | 0.094 | 1.386 | 0.166 | 0.221 |
| ICVF.FXST.combat | Control (Ref) vs 22q11.2Deletion | 0.426 | 0.078 | 5.426 | 0.000 | 0.000 |
| ICVF.GCC.combat | Control (Ref) vs 22q11.2Deletion | 0.731 | 0.096 | 7.638 | 0.000 | 0.000 |
| ICVF.IC.combat | Control (Ref) vs 22q11.2Deletion | 0.473 | 0.069 | 6.864 | 0.000 | 0.000 |
| ICVF.UNC.combat | Control (Ref) vs 22q11.2Deletion | 0.625 | 0.066 | 9.454 | 0.000 | 0.000 |
| ICVF.PCR.combat | Control (Ref) vs 22q11.2Deletion | 0.784 | 0.085 | 9.187 | 0.000 | 0.000 |
| ICVF.PLIC.combat | Control (Ref) vs 22q11.2Deletion | 0.429 | 0.070 | 6.178 | 0.000 | 0.000 |
| ICVF.PTR.combat | Control (Ref) vs 22q11.2Deletion | 0.745 | 0.090 | 8.231 | 0.000 | 0.000 |
| ICVF.RLIC.combat | Control (Ref) vs 22q11.2Deletion | 0.577 | 0.086 | 6.745 | 0.000 | 0.000 |
| ICVF.SCC.combat | Control (Ref) vs 22q11.2Deletion | 0.577 | 0.094 | 6.148 | 0.000 | 0.000 |
| ICVF.SCR.combat | Control (Ref) vs 22q11.2Deletion | 0.625 | 0.085 | 7.349 | 0.000 | 0.000 |
| ICVF.SFO.combat | Control (Ref) vs 22q11.2Deletion | 0.364 | 0.078 | 4.666 | 0.000 | 0.000 |
| ICVF.SLF.combat | Control (Ref) vs 22q11.2Deletion | 0.619 | 0.084 | 7.336 | 0.000 | 0.000 |
| ICVF.SS.combat | Control (Ref) vs 22q11.2Deletion | 0.679 | 0.086 | 7.869 | 0.000 | 0.000 |
| ICVF.TAP.combat | Control (Ref) vs 22q11.2Deletion | 0.762 | 0.090 | 8.426 | 0.000 | 0.000 |
| ISO.ACR.combat | Control (Ref) vs 22q11.2Deletion | 0.163 | 0.072 | 2.274 | 0.023 | 0.038 |
| ISO.ALIC.combat | Control (Ref) vs 22q11.2Deletion | 0.012 | 0.084 | 0.143 | 0.886 | 0.910 |
| ISO.BCC.combat | Control (Ref) vs 22q11.2Deletion | -0.062 | 0.077 | -0.808 | 0.419 | 0.493 |
| ISO.CC.combat | Control (Ref) vs 22q11.2Deletion | -0.095 | 0.076 | -1.257 | 0.209 | 0.272 |
| ISO.CGC.combat | Control (Ref) vs 22q11.2Deletion | -0.017 | 0.072 | -0.235 | 0.814 | 0.857 |
| ISO.CGH.combat | Control (Ref) vs 22q11.2Deletion | 0.072 | 0.076 | 0.947 | 0.344 | 0.414 |
| ISO.CR.combat | Control (Ref) vs 22q11.2Deletion | 0.143 | 0.069 | 2.080 | 0.038 | 0.059 |
| ISO.CST.combat | Control (Ref) vs 22q11.2Deletion | -0.026 | 0.084 | -0.304 | 0.761 | 0.812 |
| ISO.EC.combat | Control (Ref) vs 22q11.2Deletion | -0.044 | 0.078 | -0.566 | 0.572 | 0.638 |
| ISO.FX.combat | Control (Ref) vs 22q11.2Deletion | 0.394 | 0.086 | 4.596 | 0.000 | 0.000 |
| ISO.FXST.combat | Control (Ref) vs 22q11.2Deletion | -0.297 | 0.083 | -3.604 | 0.000 | 0.001 |
| ISO.GCC.combat | Control (Ref) vs 22q11.2Deletion | -0.014 | 0.083 | -0.163 | 0.871 | 0.898 |
| ISO.IC.combat | Control (Ref) vs 22q11.2Deletion | -0.033 | 0.071 | -0.467 | 0.640 | 0.701 |
| ISO.UNC.combat | Control (Ref) vs 22q11.2Deletion | -0.028 | 0.075 | -0.378 | 0.706 | 0.767 |
| ISO.PCR.combat | Control (Ref) vs 22q11.2Deletion | 0.158 | 0.067 | 2.343 | 0.019 | 0.032 |
| ISO.PLIC.combat | Control (Ref) vs 22q11.2Deletion | -0.127 | 0.086 | -1.476 | 0.140 | 0.196 |
| ISO.PTR.combat | Control (Ref) vs 22q11.2Deletion | 0.187 | 0.080 | 2.328 | 0.020 | 0.033 |
| ISO.RLIC.combat | Control (Ref) vs 22q11.2Deletion | -0.001 | 0.070 | -0.012 | 0.990 | 0.990 |
| ISO.SCC.combat | Control (Ref) vs 22q11.2Deletion | -0.165 | 0.076 | -2.166 | 0.031 | 0.049 |
| ISO.SCR.combat | Control (Ref) vs 22q11.2Deletion | 0.083 | 0.071 | 1.168 | 0.243 | 0.307 |
| ISO.SFO.combat | Control (Ref) vs 22q11.2Deletion | 0.086 | 0.083 | 1.031 | 0.303 | 0.372 |
| ISO.SLF.combat | Control (Ref) vs 22q11.2Deletion | 0.103 | 0.066 | 1.555 | 0.120 | 0.171 |
| ISO.SS.combat | Control (Ref) vs 22q11.2Deletion | 0.016 | 0.079 | 0.204 | 0.838 | 0.867 |
| ISO.TAP.combat | Control (Ref) vs 22q11.2Deletion | 0.216 | 0.089 | 2.413 | 0.016 | 0.027 |
| ODI.ACR.combat | Control (Ref) vs 22q11.2Deletion | 0.216 | 0.086 | 2.516 | 0.012 | 0.021 |
| ODI.ALIC.combat | Control (Ref) vs 22q11.2Deletion | -0.241 | 0.080 | -3.030 | 0.003 | 0.005 |
| ODI.BCC.combat | Control (Ref) vs 22q11.2Deletion | 0.188 | 0.089 | 2.112 | 0.035 | 0.055 |
| ODI.CC.combat | Control (Ref) vs 22q11.2Deletion | 0.180 | 0.088 | 2.054 | 0.040 | 0.063 |
| ODI.CGC.combat | Control (Ref) vs 22q11.2Deletion | -0.028 | 0.100 | -0.276 | 0.783 | 0.833 |
| ODI.CGH.combat | Control (Ref) vs 22q11.2Deletion | 0.414 | 0.091 | 4.546 | 0.000 | 0.000 |
| ODI.CR.combat | Control (Ref) vs 22q11.2Deletion | 0.117 | 0.085 | 1.387 | 0.166 | 0.221 |
| ODI.CST.combat | Control (Ref) vs 22q11.2Deletion | -0.272 | 0.093 | -2.918 | 0.004 | 0.007 |
| ODI.EC.combat | Control (Ref) vs 22q11.2Deletion | 0.502 | 0.085 | 5.884 | 0.000 | 0.000 |
| ODI.FX.combat | Control (Ref) vs 22q11.2Deletion | 0.037 | 0.100 | 0.367 | 0.714 | 0.770 |
| ODI.FXST.combat | Control (Ref) vs 22q11.2Deletion | 0.287 | 0.086 | 3.317 | 0.001 | 0.002 |
| ODI.GCC.combat | Control (Ref) vs 22q11.2Deletion | 0.214 | 0.092 | 2.336 | 0.020 | 0.033 |
| ODI.IC.combat | Control (Ref) vs 22q11.2Deletion | -0.242 | 0.077 | -3.144 | 0.002 | 0.003 |
| ODI.UNC.combat | Control (Ref) vs 22q11.2Deletion | 0.007 | 0.091 | 0.073 | 0.942 | 0.952 |
| ODI.PCR.combat | Control (Ref) vs 22q11.2Deletion | 0.195 | 0.090 | 2.158 | 0.031 | 0.050 |
| ODI.PLIC.combat | Control (Ref) vs 22q11.2Deletion | -0.391 | 0.083 | -4.682 | 0.000 | 0.000 |
| ODI.PTR.combat | Control (Ref) vs 22q11.2Deletion | 0.617 | 0.086 | 7.151 | 0.000 | 0.000 |
| ODI.RLIC.combat | Control (Ref) vs 22q11.2Deletion | 0.007 | 0.086 | 0.086 | 0.932 | 0.944 |
| ODI.SCC.combat | Control (Ref) vs 22q11.2Deletion | 0.074 | 0.092 | 0.804 | 0.422 | 0.494 |
| ODI.SCR.combat | Control (Ref) vs 22q11.2Deletion | -0.099 | 0.090 | -1.098 | 0.272 | 0.340 |
| ODI.SFO.combat | Control (Ref) vs 22q11.2Deletion | 0.380 | 0.084 | 4.550 | 0.000 | 0.000 |
| ODI.SLF.combat | Control (Ref) vs 22q11.2Deletion | 0.669 | 0.089 | 7.539 | 0.000 | 0.000 |
| ODI.SS.combat | Control (Ref) vs 22q11.2Deletion | 0.304 | 0.093 | 3.289 | 0.001 | 0.002 |
| ODI.TAP.combat | Control (Ref) vs 22q11.2Deletion | -0.086 | 0.102 | -0.846 | 0.398 | 0.472 |
| ICVF.combat | Control (Ref) vs 22q11.2Duplication | -0.675 | 0.099 | -6.851 | 0.000 | 0.000 |
| ISO.combat | Control (Ref) vs 22q11.2Duplication | -0.332 | 0.077 | -4.303 | 0.000 | 0.000 |
| ODI.combat | Control (Ref) vs 22q11.2Duplication | 0.324 | 0.094 | 3.460 | 0.001 | 0.001 |
| ICVF.ACR.combat | Control (Ref) vs 22q11.2Duplication | -0.660 | 0.120 | -5.485 | 0.000 | 0.000 |
| ICVF.ALIC.combat | Control (Ref) vs 22q11.2Duplication | -0.321 | 0.082 | -3.924 | 0.000 | 0.000 |
| ICVF.BCC.combat | Control (Ref) vs 22q11.2Duplication | -0.952 | 0.129 | -7.361 | 0.000 | 0.000 |
| ICVF.CC.combat | Control (Ref) vs 22q11.2Duplication | -0.931 | 0.130 | -7.157 | 0.000 | 0.000 |
| ICVF.CGC.combat | Control (Ref) vs 22q11.2Duplication | -0.679 | 0.111 | -6.112 | 0.000 | 0.000 |
| ICVF.CGH.combat | Control (Ref) vs 22q11.2Duplication | -0.392 | 0.101 | -3.874 | 0.000 | 0.000 |
| ICVF.CR.combat | Control (Ref) vs 22q11.2Duplication | -0.771 | 0.118 | -6.533 | 0.000 | 0.000 |
| ICVF.CST.combat | Control (Ref) vs 22q11.2Duplication | -0.355 | 0.118 | -3.019 | 0.003 | 0.005 |
| ICVF.EC.combat | Control (Ref) vs 22q11.2Duplication | -0.445 | 0.104 | -4.288 | 0.000 | 0.000 |
| ICVF.FX.combat | Control (Ref) vs 22q11.2Duplication | -0.423 | 0.128 | -3.307 | 0.001 | 0.002 |
| ICVF.FXST.combat | Control (Ref) vs 22q11.2Duplication | -0.347 | 0.110 | -3.153 | 0.002 | 0.003 |
| ICVF.GCC.combat | Control (Ref) vs 22q11.2Duplication | -0.750 | 0.134 | -5.610 | 0.000 | 0.000 |
| ICVF.IC.combat | Control (Ref) vs 22q11.2Duplication | -0.487 | 0.100 | -4.893 | 0.000 | 0.000 |
| ICVF.UNC.combat | Control (Ref) vs 22q11.2Duplication | -0.403 | 0.102 | -3.951 | 0.000 | 0.000 |
| ICVF.PCR.combat | Control (Ref) vs 22q11.2Duplication | -0.831 | 0.121 | -6.876 | 0.000 | 0.000 |
| ICVF.PLIC.combat | Control (Ref) vs 22q11.2Duplication | -0.452 | 0.103 | -4.393 | 0.000 | 0.000 |
| ICVF.PTR.combat | Control (Ref) vs 22q11.2Duplication | -0.873 | 0.128 | -6.822 | 0.000 | 0.000 |
| ICVF.RLIC.combat | Control (Ref) vs 22q11.2Duplication | -0.642 | 0.120 | -5.373 | 0.000 | 0.000 |
| ICVF.SCC.combat | Control (Ref) vs 22q11.2Duplication | -0.892 | 0.129 | -6.931 | 0.000 | 0.000 |
| ICVF.SCR.combat | Control (Ref) vs 22q11.2Duplication | -0.821 | 0.117 | -7.023 | 0.000 | 0.000 |
| ICVF.SFO.combat | Control (Ref) vs 22q11.2Duplication | -0.493 | 0.106 | -4.644 | 0.000 | 0.000 |
| ICVF.SLF.combat | Control (Ref) vs 22q11.2Duplication | -0.945 | 0.116 | -8.149 | 0.000 | 0.000 |
| ICVF.SS.combat | Control (Ref) vs 22q11.2Duplication | -0.629 | 0.125 | -5.040 | 0.000 | 0.000 |
| ICVF.TAP.combat | Control (Ref) vs 22q11.2Duplication | -0.650 | 0.130 | -4.997 | 0.000 | 0.000 |
| ISO.ACR.combat | Control (Ref) vs 22q11.2Duplication | -0.259 | 0.099 | -2.618 | 0.009 | 0.016 |
| ISO.ALIC.combat | Control (Ref) vs 22q11.2Duplication | -0.088 | 0.113 | -0.778 | 0.437 | 0.506 |
| ISO.BCC.combat | Control (Ref) vs 22q11.2Duplication | -0.153 | 0.104 | -1.468 | 0.142 | 0.198 |
| ISO.CC.combat | Control (Ref) vs 22q11.2Duplication | -0.116 | 0.103 | -1.126 | 0.260 | 0.327 |
| ISO.CGC.combat | Control (Ref) vs 22q11.2Duplication | -0.209 | 0.104 | -2.004 | 0.045 | 0.071 |
| ISO.CGH.combat | Control (Ref) vs 22q11.2Duplication | -0.055 | 0.107 | -0.509 | 0.611 | 0.674 |
| ISO.CR.combat | Control (Ref) vs 22q11.2Duplication | -0.356 | 0.094 | -3.784 | 0.000 | 0.000 |
| ISO.CST.combat | Control (Ref) vs 22q11.2Duplication | 0.203 | 0.110 | 1.838 | 0.066 | 0.098 |
| ISO.EC.combat | Control (Ref) vs 22q11.2Duplication | -0.023 | 0.102 | -0.230 | 0.818 | 0.858 |
| ISO.FX.combat | Control (Ref) vs 22q11.2Duplication | 0.357 | 0.123 | 2.907 | 0.004 | 0.007 |
| ISO.FXST.combat | Control (Ref) vs 22q11.2Duplication | -0.473 | 0.108 | -4.365 | 0.000 | 0.000 |
| ISO.GCC.combat | Control (Ref) vs 22q11.2Duplication | -0.160 | 0.114 | -1.401 | 0.162 | 0.218 |
| ISO.IC.combat | Control (Ref) vs 22q11.2Duplication | -0.207 | 0.097 | -2.139 | 0.033 | 0.052 |
| ISO.UNC.combat | Control (Ref) vs 22q11.2Duplication | -0.252 | 0.100 | -2.511 | 0.012 | 0.021 |
| ISO.PCR.combat | Control (Ref) vs 22q11.2Duplication | -0.333 | 0.096 | -3.454 | 0.001 | 0.001 |
| ISO.PLIC.combat | Control (Ref) vs 22q11.2Duplication | -0.223 | 0.120 | -1.851 | 0.064 | 0.096 |
| ISO.PTR.combat | Control (Ref) vs 22q11.2Duplication | -0.341 | 0.112 | -3.037 | 0.002 | 0.005 |
| ISO.RLIC.combat | Control (Ref) vs 22q11.2Duplication | -0.218 | 0.096 | -2.267 | 0.024 | 0.039 |
| ISO.SCC.combat | Control (Ref) vs 22q11.2Duplication | 0.041 | 0.111 | 0.372 | 0.710 | 0.769 |
| ISO.SCR.combat | Control (Ref) vs 22q11.2Duplication | -0.465 | 0.093 | -4.983 | 0.000 | 0.000 |
| ISO.SFO.combat | Control (Ref) vs 22q11.2Duplication | -0.354 | 0.109 | -3.259 | 0.001 | 0.002 |
| ISO.SLF.combat | Control (Ref) vs 22q11.2Duplication | -0.434 | 0.098 | -4.414 | 0.000 | 0.000 |
| ISO.SS.combat | Control (Ref) vs 22q11.2Duplication | -0.296 | 0.107 | -2.770 | 0.006 | 0.010 |
| ISO.TAP.combat | Control (Ref) vs 22q11.2Duplication | -0.283 | 0.127 | -2.224 | 0.026 | 0.043 |
| ODI.ACR.combat | Control (Ref) vs 22q11.2Duplication | 0.144 | 0.120 | 1.199 | 0.231 | 0.296 |
| ODI.ALIC.combat | Control (Ref) vs 22q11.2Duplication | 0.554 | 0.112 | 4.931 | 0.000 | 0.000 |
| ODI.BCC.combat | Control (Ref) vs 22q11.2Duplication | 0.387 | 0.124 | 3.119 | 0.002 | 0.004 |
| ODI.CC.combat | Control (Ref) vs 22q11.2Duplication | 0.165 | 0.123 | 1.345 | 0.179 | 0.238 |
| ODI.CGC.combat | Control (Ref) vs 22q11.2Duplication | 0.402 | 0.137 | 2.948 | 0.003 | 0.006 |
| ODI.CGH.combat | Control (Ref) vs 22q11.2Duplication | -0.169 | 0.127 | -1.338 | 0.181 | 0.240 |
| ODI.CR.combat | Control (Ref) vs 22q11.2Duplication | -0.066 | 0.119 | -0.555 | 0.579 | 0.643 |
| ODI.CST.combat | Control (Ref) vs 22q11.2Duplication | 0.422 | 0.132 | 3.190 | 0.001 | 0.003 |
| ODI.EC.combat | Control (Ref) vs 22q11.2Duplication | 0.361 | 0.125 | 2.881 | 0.004 | 0.007 |
| ODI.FX.combat | Control (Ref) vs 22q11.2Duplication | 0.238 | 0.137 | 1.739 | 0.082 | 0.120 |
| ODI.FXST.combat | Control (Ref) vs 22q11.2Duplication | -0.024 | 0.118 | -0.207 | 0.836 | 0.867 |
| ODI.GCC.combat | Control (Ref) vs 22q11.2Duplication | 0.073 | 0.125 | 0.581 | 0.561 | 0.628 |
| ODI.IC.combat | Control (Ref) vs 22q11.2Duplication | 0.311 | 0.108 | 2.894 | 0.004 | 0.007 |
| ODI.UNC.combat | Control (Ref) vs 22q11.2Duplication | 0.420 | 0.134 | 3.136 | 0.002 | 0.003 |
| ODI.PCR.combat | Control (Ref) vs 22q11.2Duplication | -0.305 | 0.129 | -2.368 | 0.018 | 0.031 |
| ODI.PLIC.combat | Control (Ref) vs 22q11.2Duplication | 0.374 | 0.115 | 3.251 | 0.001 | 0.002 |
| ODI.PTR.combat | Control (Ref) vs 22q11.2Duplication | 0.015 | 0.128 | 0.116 | 0.908 | 0.923 |
| ODI.RLIC.combat | Control (Ref) vs 22q11.2Duplication | -0.172 | 0.123 | -1.398 | 0.162 | 0.218 |
| ODI.SCC.combat | Control (Ref) vs 22q11.2Duplication | -0.070 | 0.130 | -0.540 | 0.589 | 0.652 |
| ODI.SCR.combat | Control (Ref) vs 22q11.2Duplication | -0.213 | 0.128 | -1.665 | 0.096 | 0.140 |
| ODI.SFO.combat | Control (Ref) vs 22q11.2Duplication | 0.096 | 0.121 | 0.792 | 0.429 | 0.500 |
| ODI.SLF.combat | Control (Ref) vs 22q11.2Duplication | 0.028 | 0.133 | 0.211 | 0.833 | 0.867 |
| ODI.SS.combat | Control (Ref) vs 22q11.2Duplication | -0.239 | 0.135 | -1.775 | 0.076 | 0.112 |
| ODI.TAP.combat | Control (Ref) vs 22q11.2Duplication | 0.113 | 0.145 | 0.784 | 0.433 | 0.504 |
| ICVF.combat | 22q11.2Deletion (Ref) vs 22q11.2Duplication | -0.955 | 0.096 | -9.896 | 0.000 | 0.000 |
| ISO.combat | 22q11.2Deletion (Ref) vs 22q11.2Duplication | -0.510 | 0.115 | -4.440 | 0.000 | 0.000 |
| ODI.combat | 22q11.2Deletion (Ref) vs 22q11.2Duplication | 0.142 | 0.136 | 1.046 | 0.298 | 0.368 |
| ICVF.ACR.combat | 22q11.2Deletion (Ref) vs 22q11.2Duplication | -1.104 | 0.098 | -11.235 | 0.000 | 0.000 |
| ICVF.ALIC.combat | 22q11.2Deletion (Ref) vs 22q11.2Duplication | -0.804 | 0.091 | -8.862 | 0.000 | 0.000 |
| ICVF.BCC.combat | 22q11.2Deletion (Ref) vs 22q11.2Duplication | -1.172 | 0.100 | -11.675 | 0.000 | 0.000 |
| ICVF.CC.combat | 22q11.2Deletion (Ref) vs 22q11.2Duplication | -1.155 | 0.102 | -11.281 | 0.000 | 0.000 |
| ICVF.CGC.combat | 22q11.2Deletion (Ref) vs 22q11.2Duplication | -0.946 | 0.106 | -8.961 | 0.000 | 0.000 |
| ICVF.CGH.combat | 22q11.2Deletion (Ref) vs 22q11.2Duplication | -0.633 | 0.126 | -5.015 | 0.000 | 0.000 |
| ICVF.CR.combat | 22q11.2Deletion (Ref) vs 22q11.2Duplication | -1.103 | 0.099 | -11.105 | 0.000 | 0.000 |
| ICVF.CST.combat | 22q11.2Deletion (Ref) vs 22q11.2Duplication | -0.524 | 0.139 | -3.766 | 0.000 | 0.001 |
| ICVF.EC.combat | 22q11.2Deletion (Ref) vs 22q11.2Duplication | -0.799 | 0.098 | -8.168 | 0.000 | 0.000 |
| ICVF.FX.combat | 22q11.2Deletion (Ref) vs 22q11.2Duplication | -0.510 | 0.158 | -3.231 | 0.002 | 0.003 |
| ICVF.FXST.combat | 22q11.2Deletion (Ref) vs 22q11.2Duplication | -0.815 | 0.112 | -7.302 | 0.000 | 0.000 |
| ICVF.GCC.combat | 22q11.2Deletion (Ref) vs 22q11.2Duplication | -1.137 | 0.107 | -10.597 | 0.000 | 0.000 |
| ICVF.IC.combat | 22q11.2Deletion (Ref) vs 22q11.2Duplication | -0.926 | 0.100 | -9.280 | 0.000 | 0.000 |
| ICVF.UNC.combat | 22q11.2Deletion (Ref) vs 22q11.2Duplication | -0.886 | 0.095 | -9.313 | 0.000 | 0.000 |
| ICVF.PCR.combat | 22q11.2Deletion (Ref) vs 22q11.2Duplication | -1.097 | 0.103 | -10.598 | 0.000 | 0.000 |
| ICVF.PLIC.combat | 22q11.2Deletion (Ref) vs 22q11.2Duplication | -0.812 | 0.114 | -7.101 | 0.000 | 0.000 |
| ICVF.PTR.combat | 22q11.2Deletion (Ref) vs 22q11.2Duplication | -1.147 | 0.103 | -11.113 | 0.000 | 0.000 |
| ICVF.RLIC.combat | 22q11.2Deletion (Ref) vs 22q11.2Duplication | -1.041 | 0.101 | -10.356 | 0.000 | 0.000 |
| ICVF.SCC.combat | 22q11.2Deletion (Ref) vs 22q11.2Duplication | -1.035 | 0.117 | -8.851 | 0.000 | 0.000 |
| ICVF.SCR.combat | 22q11.2Deletion (Ref) vs 22q11.2Duplication | -1.074 | 0.103 | -10.418 | 0.000 | 0.000 |
| ICVF.SFO.combat | 22q11.2Deletion (Ref) vs 22q11.2Duplication | -0.908 | 0.113 | -8.034 | 0.000 | 0.000 |
| ICVF.SLF.combat | 22q11.2Deletion (Ref) vs 22q11.2Duplication | -1.080 | 0.098 | -11.053 | 0.000 | 0.000 |
| ICVF.SS.combat | 22q11.2Deletion (Ref) vs 22q11.2Duplication | -1.074 | 0.103 | -10.455 | 0.000 | 0.000 |
| ICVF.TAP.combat | 22q11.2Deletion (Ref) vs 22q11.2Duplication | -1.034 | 0.127 | -8.118 | 0.000 | 0.000 |
| ISO.ACR.combat | 22q11.2Deletion (Ref) vs 22q11.2Duplication | -0.585 | 0.134 | -4.366 | 0.000 | 0.000 |
| ISO.ALIC.combat | 22q11.2Deletion (Ref) vs 22q11.2Duplication | -0.193 | 0.161 | -1.197 | 0.234 | 0.298 |
| ISO.BCC.combat | 22q11.2Deletion (Ref) vs 22q11.2Duplication | -0.219 | 0.150 | -1.454 | 0.149 | 0.203 |
| ISO.CC.combat | 22q11.2Deletion (Ref) vs 22q11.2Duplication | -0.044 | 0.163 | -0.267 | 0.790 | 0.837 |
| ISO.CGC.combat | 22q11.2Deletion (Ref) vs 22q11.2Duplication | -0.273 | 0.141 | -1.933 | 0.056 | 0.085 |
| ISO.CGH.combat | 22q11.2Deletion (Ref) vs 22q11.2Duplication | -0.222 | 0.145 | -1.537 | 0.128 | 0.180 |
| ISO.CR.combat | 22q11.2Deletion (Ref) vs 22q11.2Duplication | -0.640 | 0.130 | -4.930 | 0.000 | 0.000 |
| ISO.CST.combat | 22q11.2Deletion (Ref) vs 22q11.2Duplication | 0.356 | 0.147 | 2.428 | 0.017 | 0.029 |
| ISO.EC.combat | 22q11.2Deletion (Ref) vs 22q11.2Duplication | -0.134 | 0.137 | -0.976 | 0.332 | 0.403 |
| ISO.FX.combat | 22q11.2Deletion (Ref) vs 22q11.2Duplication | -0.023 | 0.178 | -0.129 | 0.898 | 0.919 |
| ISO.FXST.combat | 22q11.2Deletion (Ref) vs 22q11.2Duplication | -0.233 | 0.153 | -1.515 | 0.133 | 0.186 |
| ISO.GCC.combat | 22q11.2Deletion (Ref) vs 22q11.2Duplication | -0.144 | 0.160 | -0.895 | 0.373 | 0.446 |
| ISO.IC.combat | 22q11.2Deletion (Ref) vs 22q11.2Duplication | -0.259 | 0.136 | -1.910 | 0.059 | 0.089 |
| ISO.UNC.combat | 22q11.2Deletion (Ref) vs 22q11.2Duplication | -0.257 | 0.145 | -1.775 | 0.079 | 0.116 |
| ISO.PCR.combat | 22q11.2Deletion (Ref) vs 22q11.2Duplication | -0.657 | 0.131 | -5.000 | 0.000 | 0.000 |
| ISO.PLIC.combat | 22q11.2Deletion (Ref) vs 22q11.2Duplication | -0.133 | 0.150 | -0.888 | 0.377 | 0.448 |
| ISO.PTR.combat | 22q11.2Deletion (Ref) vs 22q11.2Duplication | -0.669 | 0.135 | -4.944 | 0.000 | 0.000 |
| ISO.RLIC.combat | 22q11.2Deletion (Ref) vs 22q11.2Duplication | -0.346 | 0.128 | -2.696 | 0.008 | 0.015 |
| ISO.SCC.combat | 22q11.2Deletion (Ref) vs 22q11.2Duplication | 0.265 | 0.170 | 1.559 | 0.122 | 0.173 |
| ISO.SCR.combat | 22q11.2Deletion (Ref) vs 22q11.2Duplication | -0.618 | 0.132 | -4.689 | 0.000 | 0.000 |
| ISO.SFO.combat | 22q11.2Deletion (Ref) vs 22q11.2Duplication | -0.625 | 0.136 | -4.611 | 0.000 | 0.000 |
| ISO.SLF.combat | 22q11.2Deletion (Ref) vs 22q11.2Duplication | -0.609 | 0.128 | -4.776 | 0.000 | 0.000 |
| ISO.SS.combat | 22q11.2Deletion (Ref) vs 22q11.2Duplication | -0.504 | 0.125 | -4.050 | 0.000 | 0.000 |
| ISO.TAP.combat | 22q11.2Deletion (Ref) vs 22q11.2Duplication | -0.467 | 0.158 | -2.962 | 0.004 | 0.007 |
| ODI.ACR.combat | 22q11.2Deletion (Ref) vs 22q11.2Duplication | -0.121 | 0.160 | -0.757 | 0.451 | 0.520 |
| ODI.ALIC.combat | 22q11.2Deletion (Ref) vs 22q11.2Duplication | 0.743 | 0.138 | 5.395 | 0.000 | 0.000 |
| ODI.BCC.combat | 22q11.2Deletion (Ref) vs 22q11.2Duplication | 0.200 | 0.160 | 1.248 | 0.215 | 0.278 |
| ODI.CC.combat | 22q11.2Deletion (Ref) vs 22q11.2Duplication | 0.002 | 0.161 | 0.013 | 0.990 | 0.990 |
| ODI.CGC.combat | 22q11.2Deletion (Ref) vs 22q11.2Duplication | 0.359 | 0.166 | 2.159 | 0.033 | 0.053 |
| ODI.CGH.combat | 22q11.2Deletion (Ref) vs 22q11.2Duplication | -0.511 | 0.156 | -3.265 | 0.002 | 0.003 |
| ODI.CR.combat | 22q11.2Deletion (Ref) vs 22q11.2Duplication | -0.204 | 0.157 | -1.296 | 0.198 | 0.259 |
| ODI.CST.combat | 22q11.2Deletion (Ref) vs 22q11.2Duplication | 0.555 | 0.152 | 3.650 | 0.000 | 0.001 |
| ODI.EC.combat | 22q11.2Deletion (Ref) vs 22q11.2Duplication | -0.095 | 0.161 | -0.589 | 0.557 | 0.626 |
| ODI.FX.combat | 22q11.2Deletion (Ref) vs 22q11.2Duplication | 0.246 | 0.168 | 1.462 | 0.147 | 0.203 |
| ODI.FXST.combat | 22q11.2Deletion (Ref) vs 22q11.2Duplication | -0.295 | 0.151 | -1.952 | 0.054 | 0.082 |
| ODI.GCC.combat | 22q11.2Deletion (Ref) vs 22q11.2Duplication | -0.111 | 0.171 | -0.654 | 0.515 | 0.585 |
| ODI.IC.combat | 22q11.2Deletion (Ref) vs 22q11.2Duplication | 0.493 | 0.145 | 3.411 | 0.001 | 0.002 |
| ODI.UNC.combat | 22q11.2Deletion (Ref) vs 22q11.2Duplication | 0.433 | 0.171 | 2.539 | 0.013 | 0.022 |
| ODI.PCR.combat | 22q11.2Deletion (Ref) vs 22q11.2Duplication | -0.483 | 0.157 | -3.077 | 0.003 | 0.005 |
| ODI.PLIC.combat | 22q11.2Deletion (Ref) vs 22q11.2Duplication | 0.672 | 0.153 | 4.407 | 0.000 | 0.000 |
| ODI.PTR.combat | 22q11.2Deletion (Ref) vs 22q11.2Duplication | -0.601 | 0.145 | -4.158 | 0.000 | 0.000 |
| ODI.RLIC.combat | 22q11.2Deletion (Ref) vs 22q11.2Duplication | -0.246 | 0.151 | -1.628 | 0.107 | 0.154 |
| ODI.SCC.combat | 22q11.2Deletion (Ref) vs 22q11.2Duplication | -0.124 | 0.167 | -0.741 | 0.461 | 0.529 |
| ODI.SCR.combat | 22q11.2Deletion (Ref) vs 22q11.2Duplication | -0.106 | 0.165 | -0.646 | 0.520 | 0.589 |
| ODI.SFO.combat | 22q11.2Deletion (Ref) vs 22q11.2Duplication | -0.213 | 0.166 | -1.288 | 0.201 | 0.262 |
| ODI.SLF.combat | 22q11.2Deletion (Ref) vs 22q11.2Duplication | -0.541 | 0.159 | -3.409 | 0.001 | 0.002 |
| ODI.SS.combat | 22q11.2Deletion (Ref) vs 22q11.2Duplication | -0.639 | 0.156 | -4.090 | 0.000 | 0.000 |
| ODI.TAP.combat | 22q11.2Deletion (Ref) vs 22q11.2Duplication | 0.173 | 0.174 | 0.993 | 0.323 | 0.394 |
| ICVF.combat | GeneDosage (1 = del, 2 = con, 3 dup) | -0.779 | 0.078 | -9.930 | 0.000 | 0.000 |
| ISO.combat | GeneDosage (1 = del, 2 = con, 3 dup) | -0.229 | 0.063 | -3.630 | 0.000 | 0.001 |
| ODI.combat | GeneDosage (1 = del, 2 = con, 3 dup) | -0.036 | 0.076 | -0.476 | 0.634 | 0.697 |
| ICVF.ACR.combat | GeneDosage (1 = del, 2 = con, 3 dup) | -0.957 | 0.093 | -10.301 | 0.000 | 0.000 |
| ICVF.ALIC.combat | GeneDosage (1 = del, 2 = con, 3 dup) | -0.486 | 0.066 | -7.363 | 0.000 | 0.000 |
| ICVF.BCC.combat | GeneDosage (1 = del, 2 = con, 3 dup) | -1.113 | 0.101 | -11.016 | 0.000 | 0.000 |
| ICVF.CC.combat | GeneDosage (1 = del, 2 = con, 3 dup) | -1.088 | 0.101 | -10.732 | 0.000 | 0.000 |
| ICVF.CGC.combat | GeneDosage (1 = del, 2 = con, 3 dup) | -0.672 | 0.091 | -7.417 | 0.000 | 0.000 |
| ICVF.CGH.combat | GeneDosage (1 = del, 2 = con, 3 dup) | -0.313 | 0.083 | -3.780 | 0.000 | 0.000 |
| ICVF.CR.combat | GeneDosage (1 = del, 2 = con, 3 dup) | -1.010 | 0.092 | -11.001 | 0.000 | 0.000 |
| ICVF.CST.combat | GeneDosage (1 = del, 2 = con, 3 dup) | -0.356 | 0.094 | -3.800 | 0.000 | 0.000 |
| ICVF.EC.combat | GeneDosage (1 = del, 2 = con, 3 dup) | -0.551 | 0.083 | -6.646 | 0.000 | 0.000 |
| ICVF.FX.combat | GeneDosage (1 = del, 2 = con, 3 dup) | -0.326 | 0.105 | -3.099 | 0.002 | 0.004 |
| ICVF.FXST.combat | GeneDosage (1 = del, 2 = con, 3 dup) | -0.564 | 0.088 | -6.445 | 0.000 | 0.000 |
| ICVF.GCC.combat | GeneDosage (1 = del, 2 = con, 3 dup) | -1.013 | 0.104 | -9.711 | 0.000 | 0.000 |
| ICVF.IC.combat | GeneDosage (1 = del, 2 = con, 3 dup) | -0.675 | 0.078 | -8.610 | 0.000 | 0.000 |
| ICVF.UNC.combat | GeneDosage (1 = del, 2 = con, 3 dup) | -0.784 | 0.073 | -10.799 | 0.000 | 0.000 |
| ICVF.PCR.combat | GeneDosage (1 = del, 2 = con, 3 dup) | -1.089 | 0.093 | -11.666 | 0.000 | 0.000 |
| ICVF.PLIC.combat | GeneDosage (1 = del, 2 = con, 3 dup) | -0.622 | 0.078 | -7.932 | 0.000 | 0.000 |
| ICVF.PTR.combat | GeneDosage (1 = del, 2 = con, 3 dup) | -1.082 | 0.099 | -10.912 | 0.000 | 0.000 |
| ICVF.RLIC.combat | GeneDosage (1 = del, 2 = con, 3 dup) | -0.836 | 0.094 | -8.861 | 0.000 | 0.000 |
| ICVF.SCC.combat | GeneDosage (1 = del, 2 = con, 3 dup) | -0.938 | 0.102 | -9.170 | 0.000 | 0.000 |
| ICVF.SCR.combat | GeneDosage (1 = del, 2 = con, 3 dup) | -0.954 | 0.093 | -10.275 | 0.000 | 0.000 |
| ICVF.SFO.combat | GeneDosage (1 = del, 2 = con, 3 dup) | -0.586 | 0.087 | -6.769 | 0.000 | 0.000 |
| ICVF.SLF.combat | GeneDosage (1 = del, 2 = con, 3 dup) | -1.002 | 0.092 | -10.935 | 0.000 | 0.000 |
| ICVF.SS.combat | GeneDosage (1 = del, 2 = con, 3 dup) | -0.921 | 0.096 | -9.602 | 0.000 | 0.000 |
| ICVF.TAP.combat | GeneDosage (1 = del, 2 = con, 3 dup) | -0.993 | 0.101 | -9.873 | 0.000 | 0.000 |
| ISO.ACR.combat | GeneDosage (1 = del, 2 = con, 3 dup) | -0.278 | 0.081 | -3.410 | 0.001 | 0.001 |
| ISO.ALIC.combat | GeneDosage (1 = del, 2 = con, 3 dup) | -0.058 | 0.094 | -0.617 | 0.537 | 0.606 |
| ISO.BCC.combat | GeneDosage (1 = del, 2 = con, 3 dup) | -0.021 | 0.086 | -0.243 | 0.808 | 0.854 |
| ISO.CC.combat | GeneDosage (1 = del, 2 = con, 3 dup) | 0.031 | 0.086 | 0.357 | 0.721 | 0.775 |
| ISO.CGC.combat | GeneDosage (1 = del, 2 = con, 3 dup) | -0.088 | 0.082 | -1.083 | 0.279 | 0.347 |
| ISO.CGH.combat | GeneDosage (1 = del, 2 = con, 3 dup) | -0.090 | 0.086 | -1.048 | 0.295 | 0.366 |
| ISO.CR.combat | GeneDosage (1 = del, 2 = con, 3 dup) | -0.307 | 0.078 | -3.951 | 0.000 | 0.000 |
| ISO.CST.combat | GeneDosage (1 = del, 2 = con, 3 dup) | 0.130 | 0.092 | 1.402 | 0.161 | 0.218 |
| ISO.EC.combat | GeneDosage (1 = del, 2 = con, 3 dup) | 0.031 | 0.088 | 0.353 | 0.724 | 0.776 |
| ISO.FX.combat | GeneDosage (1 = del, 2 = con, 3 dup) | -0.186 | 0.099 | -1.873 | 0.061 | 0.092 |
| ISO.FXST.combat | GeneDosage (1 = del, 2 = con, 3 dup) | 0.039 | 0.093 | 0.417 | 0.677 | 0.738 |
| ISO.GCC.combat | GeneDosage (1 = del, 2 = con, 3 dup) | -0.067 | 0.093 | -0.723 | 0.470 | 0.536 |
| ISO.IC.combat | GeneDosage (1 = del, 2 = con, 3 dup) | -0.079 | 0.080 | -0.988 | 0.323 | 0.394 |
| ISO.UNC.combat | GeneDosage (1 = del, 2 = con, 3 dup) | -0.099 | 0.085 | -1.166 | 0.244 | 0.307 |
| ISO.PCR.combat | GeneDosage (1 = del, 2 = con, 3 dup) | -0.310 | 0.077 | -4.021 | 0.000 | 0.000 |
| ISO.PLIC.combat | GeneDosage (1 = del, 2 = con, 3 dup) | 0.003 | 0.097 | 0.030 | 0.976 | 0.982 |
| ISO.PTR.combat | GeneDosage (1 = del, 2 = con, 3 dup) | -0.343 | 0.091 | -3.760 | 0.000 | 0.000 |
| ISO.RLIC.combat | GeneDosage (1 = del, 2 = con, 3 dup) | -0.114 | 0.079 | -1.450 | 0.147 | 0.203 |
| ISO.SCC.combat | GeneDosage (1 = del, 2 = con, 3 dup) | 0.175 | 0.089 | 1.977 | 0.048 | 0.074 |
| ISO.SCR.combat | GeneDosage (1 = del, 2 = con, 3 dup) | -0.315 | 0.079 | -3.995 | 0.000 | 0.000 |
| ISO.SFO.combat | GeneDosage (1 = del, 2 = con, 3 dup) | -0.266 | 0.091 | -2.915 | 0.004 | 0.007 |
| ISO.SLF.combat | GeneDosage (1 = del, 2 = con, 3 dup) | -0.312 | 0.078 | -4.014 | 0.000 | 0.000 |
| ISO.SS.combat | GeneDosage (1 = del, 2 = con, 3 dup) | -0.168 | 0.088 | -1.907 | 0.057 | 0.086 |
| ISO.TAP.combat | GeneDosage (1 = del, 2 = con, 3 dup) | -0.341 | 0.101 | -3.363 | 0.001 | 0.002 |
| ODI.ACR.combat | GeneDosage (1 = del, 2 = con, 3 dup) | -0.128 | 0.098 | -1.311 | 0.190 | 0.250 |
| ODI.ALIC.combat | GeneDosage (1 = del, 2 = con, 3 dup) | 0.492 | 0.090 | 5.452 | 0.000 | 0.000 |
| ODI.BCC.combat | GeneDosage (1 = del, 2 = con, 3 dup) | 0.022 | 0.101 | 0.214 | 0.831 | 0.867 |
| ODI.CC.combat | GeneDosage (1 = del, 2 = con, 3 dup) | -0.081 | 0.099 | -0.816 | 0.415 | 0.490 |
| ODI.CGC.combat | GeneDosage (1 = del, 2 = con, 3 dup) | 0.222 | 0.112 | 1.977 | 0.048 | 0.074 |
| ODI.CGH.combat | GeneDosage (1 = del, 2 = con, 3 dup) | -0.459 | 0.103 | -4.470 | 0.000 | 0.000 |
| ODI.CR.combat | GeneDosage (1 = del, 2 = con, 3 dup) | -0.139 | 0.096 | -1.444 | 0.149 | 0.203 |
| ODI.CST.combat | GeneDosage (1 = del, 2 = con, 3 dup) | 0.451 | 0.106 | 4.268 | 0.000 | 0.000 |
| ODI.EC.combat | GeneDosage (1 = del, 2 = con, 3 dup) | -0.291 | 0.100 | -2.923 | 0.004 | 0.007 |
| ODI.FX.combat | GeneDosage (1 = del, 2 = con, 3 dup) | 0.081 | 0.112 | 0.723 | 0.470 | 0.536 |
| ODI.FXST.combat | GeneDosage (1 = del, 2 = con, 3 dup) | -0.252 | 0.098 | -2.581 | 0.010 | 0.017 |
| ODI.GCC.combat | GeneDosage (1 = del, 2 = con, 3 dup) | -0.160 | 0.103 | -1.559 | 0.119 | 0.171 |
| ODI.IC.combat | GeneDosage (1 = del, 2 = con, 3 dup) | 0.374 | 0.087 | 4.285 | 0.000 | 0.000 |
| ODI.UNC.combat | GeneDosage (1 = del, 2 = con, 3 dup) | 0.205 | 0.105 | 1.950 | 0.051 | 0.079 |
| ODI.PCR.combat | GeneDosage (1 = del, 2 = con, 3 dup) | -0.324 | 0.103 | -3.158 | 0.002 | 0.003 |
| ODI.PLIC.combat | GeneDosage (1 = del, 2 = con, 3 dup) | 0.535 | 0.094 | 5.705 | 0.000 | 0.000 |
| ODI.PTR.combat | GeneDosage (1 = del, 2 = con, 3 dup) | -0.564 | 0.099 | -5.668 | 0.000 | 0.000 |
| ODI.RLIC.combat | GeneDosage (1 = del, 2 = con, 3 dup) | -0.089 | 0.099 | -0.899 | 0.369 | 0.442 |
| ODI.SCC.combat | GeneDosage (1 = del, 2 = con, 3 dup) | -0.101 | 0.104 | -0.965 | 0.335 | 0.405 |
| ODI.SCR.combat | GeneDosage (1 = del, 2 = con, 3 dup) | -0.013 | 0.102 | -0.124 | 0.901 | 0.920 |
| ODI.SFO.combat | GeneDosage (1 = del, 2 = con, 3 dup) | -0.299 | 0.097 | -3.097 | 0.002 | 0.004 |
| ODI.SLF.combat | GeneDosage (1 = del, 2 = con, 3 dup) | -0.600 | 0.103 | -5.800 | 0.000 | 0.000 |
| ODI.SS.combat | GeneDosage (1 = del, 2 = con, 3 dup) | -0.397 | 0.105 | -3.766 | 0.000 | 0.000 |
| ODI.TAP.combat | GeneDosage (1 = del, 2 = con, 3 dup) | 0.137 | 0.115 | 1.190 | 0.234 | 0.298 |

| sTable 5. Sensitivity analysis - Group differences across individuals scanned at the same scanner site | | | | | | | |
| --- | --- | --- | --- | --- | --- | --- | --- |
| Dependent variable | **Analysis** | **Std.B.** | | **SE** | **T-value** | **P-value** | **pFDR** |
| ICVF | Control (Ref) vs 22q11.2Deletion | 0.721 | | 0.138 | 5.215 | 0.000 | 0.000 |
| ISO | Control (Ref) vs 22q11.2Deletion | 0.163 | | 0.127 | 1.290 | 0.200 | 0.276 |
| ODI | Control (Ref) vs 22q11.2Deletion | 0.229 | | 0.163 | 1.398 | 0.165 | 0.244 |
| ICVF-ACR | Control (Ref) vs 22q11.2Deletion | 1.036 | | 0.156 | 6.632 | 0.000 | 0.000 |
| ICVF-ALIC | Control (Ref) vs 22q11.2Deletion | 0.553 | | 0.118 | 4.685 | 0.000 | 0.000 |
| ICVF-BCC | Control (Ref) vs 22q11.2Deletion | 1.093 | | 0.170 | 6.447 | 0.000 | 0.000 |
| ICVF-CC | Control (Ref) vs 22q11.2Deletion | 1.070 | | 0.172 | 6.214 | 0.000 | 0.000 |
| ICVF-CGC | Control (Ref) vs 22q11.2Deletion | 0.596 | | 0.150 | 3.971 | 0.000 | 0.000 |
| ICVF-CGH | Control (Ref) vs 22q11.2Deletion | 0.203 | | 0.165 | 1.230 | 0.221 | 0.296 |
| ICVF-CR | Control (Ref) vs 22q11.2Deletion | 0.997 | | 0.164 | 6.067 | 0.000 | 0.000 |
| ICVF-CST | Control (Ref) vs 22q11.2Deletion | 0.219 | | 0.192 | 1.141 | 0.256 | 0.337 |
| ICVF-EC | Control (Ref) vs 22q11.2Deletion | 0.537 | | 0.131 | 4.106 | 0.000 | 0.000 |
| ICVF-FX | Control (Ref) vs 22q11.2Deletion | 0.112 | | 0.207 | 0.543 | 0.588 | 0.681 |
| ICVF-FXST | Control (Ref) vs 22q11.2Deletion | 0.668 | | 0.157 | 4.256 | 0.000 | 0.000 |
| ICVF-GCC | Control (Ref) vs 22q11.2Deletion | 1.149 | | 0.163 | 7.042 | 0.000 | 0.000 |
| ICVF-IC | Control (Ref) vs 22q11.2Deletion | 0.772 | | 0.137 | 5.640 | 0.000 | 0.000 |
| ICVF-UNC | Control (Ref) vs 22q11.2Deletion | 0.745 | | 0.138 | 5.385 | 0.000 | 0.000 |
| ICVF-PCR | Control (Ref) vs 22q11.2Deletion | 1.027 | | 0.174 | 5.897 | 0.000 | 0.000 |
| ICVF-PLIC | Control (Ref) vs 22q11.2Deletion | 0.652 | | 0.154 | 4.239 | 0.000 | 0.000 |
| ICVF-PTR | Control (Ref) vs 22q11.2Deletion | 1.051 | | 0.179 | 5.859 | 0.000 | 0.000 |
| ICVF-RLIC | Control (Ref) vs 22q11.2Deletion | 0.890 | | 0.171 | 5.204 | 0.000 | 0.000 |
| ICVF-SCC | Control (Ref) vs 22q11.2Deletion | 0.834 | | 0.197 | 4.235 | 0.000 | 0.000 |
| ICVF-SCR | Control (Ref) vs 22q11.2Deletion | 0.876 | | 0.170 | 5.141 | 0.000 | 0.000 |
| ICVF-SFO | Control (Ref) vs 22q11.2Deletion | 0.572 | | 0.150 | 3.817 | 0.000 | 0.001 |
| ICVF-SLF | Control (Ref) vs 22q11.2Deletion | 0.903 | | 0.161 | 5.623 | 0.000 | 0.000 |
| ICVF-SS | Control (Ref) vs 22q11.2Deletion | 0.996 | | 0.166 | 5.986 | 0.000 | 0.000 |
| ICVF-TAP | Control (Ref) vs 22q11.2Deletion | 0.964 | | 0.196 | 4.922 | 0.000 | 0.000 |
| ISO-ACR | Control (Ref) vs 22q11.2Deletion | 0.371 | | 0.167 | 2.217 | 0.029 | 0.052 |
| ISO-ALIC | Control (Ref) vs 22q11.2Deletion | 0.029 | | 0.188 | 0.153 | 0.879 | 0.928 |
| ISO-BCC | Control (Ref) vs 22q11.2Deletion | -0.036 | | 0.190 | -0.191 | 0.849 | 0.903 |
| ISO-CC | Control (Ref) vs 22q11.2Deletion | -0.111 | | 0.185 | -0.600 | 0.550 | 0.641 |
| ISO-CGC | Control (Ref) vs 22q11.2Deletion | 0.015 | | 0.166 | 0.091 | 0.928 | 0.955 |
| ISO-CGH | Control (Ref) vs 22q11.2Deletion | 0.202 | | 0.181 | 1.115 | 0.267 | 0.349 |
| ISO-CR | Control (Ref) vs 22q11.2Deletion | 0.326 | | 0.159 | 2.059 | 0.042 | 0.073 |
| ISO-CST | Control (Ref) vs 22q11.2Deletion | -0.027 | | 0.177 | -0.150 | 0.881 | 0.928 |
| ISO-EC | Control (Ref) vs 22q11.2Deletion | -0.060 | | 0.162 | -0.372 | 0.711 | 0.790 |
| ISO-FX | Control (Ref) vs 22q11.2Deletion | 0.583 | | 0.195 | 2.989 | 0.003 | 0.008 |
| ISO-FXST | Control (Ref) vs 22q11.2Deletion | -0.267 | | 0.196 | -1.364 | 0.175 | 0.254 |
| ISO-GCC | Control (Ref) vs 22q11.2Deletion | 0.007 | | 0.195 | 0.035 | 0.972 | 0.976 |
| ISO-IC | Control (Ref) vs 22q11.2Deletion | -0.036 | | 0.155 | -0.232 | 0.817 | 0.879 |
| ISO-UNC | Control (Ref) vs 22q11.2Deletion | -0.045 | | 0.179 | -0.254 | 0.800 | 0.863 |
| ISO-PCR | Control (Ref) vs 22q11.2Deletion | 0.322 | | 0.154 | 2.091 | 0.039 | 0.070 |
| ISO-PLIC | Control (Ref) vs 22q11.2Deletion | -0.166 | | 0.173 | -0.961 | 0.338 | 0.428 |
| ISO-PTR | Control (Ref) vs 22q11.2Deletion | 0.388 | | 0.162 | 2.395 | 0.018 | 0.035 |
| ISO-RLIC | Control (Ref) vs 22q11.2Deletion | 0.024 | | 0.159 | 0.151 | 0.880 | 0.928 |
| ISO-SCC | Control (Ref) vs 22q11.2Deletion | -0.233 | | 0.166 | -1.401 | 0.164 | 0.244 |
| ISO-SCR | Control (Ref) vs 22q11.2Deletion | 0.156 | | 0.166 | 0.942 | 0.348 | 0.439 |
| ISO-SFO | Control (Ref) vs 22q11.2Deletion | 0.133 | | 0.192 | 0.693 | 0.490 | 0.588 |
| ISO-SLF | Control (Ref) vs 22q11.2Deletion | 0.226 | | 0.135 | 1.670 | 0.098 | 0.157 |
| ISO-SS | Control (Ref) vs 22q11.2Deletion | 0.077 | | 0.176 | 0.438 | 0.662 | 0.752 |
| ISO-TAP | Control (Ref) vs 22q11.2Deletion | 0.429 | | 0.188 | 2.280 | 0.025 | 0.045 |
| ODI-ACR | Control (Ref) vs 22q11.2Deletion | 0.264 | | 0.188 | 1.406 | 0.162 | 0.243 |
| ODI-ALIC | Control (Ref) vs 22q11.2Deletion | -0.386 | | 0.185 | -2.083 | 0.040 | 0.071 |
| ODI-BCC | Control (Ref) vs 22q11.2Deletion | 0.197 | | 0.180 | 1.094 | 0.277 | 0.359 |
| ODI-CC | Control (Ref) vs 22q11.2Deletion | 0.209 | | 0.176 | 1.192 | 0.236 | 0.315 |
| ODI-CGC | Control (Ref) vs 22q11.2Deletion | -0.089 | | 0.207 | -0.429 | 0.669 | 0.757 |
| ODI-CGH | Control (Ref) vs 22q11.2Deletion | 0.372 | | 0.202 | 1.839 | 0.069 | 0.114 |
| ODI-CR | Control (Ref) vs 22q11.2Deletion | 0.127 | | 0.183 | 0.698 | 0.487 | 0.586 |
| ODI-CST | Control (Ref) vs 22q11.2Deletion | -0.385 | | 0.206 | -1.875 | 0.063 | 0.106 |
| ODI-EC | Control (Ref) vs 22q11.2Deletion | 0.610 | | 0.184 | 3.309 | 0.001 | 0.003 |
| ODI-FX | Control (Ref) vs 22q11.2Deletion | 0.020 | | 0.214 | 0.092 | 0.927 | 0.955 |
| ODI-FXST | Control (Ref) vs 22q11.2Deletion | 0.427 | | 0.187 | 2.278 | 0.025 | 0.045 |
| ODI-GCC | Control (Ref) vs 22q11.2Deletion | 0.256 | | 0.196 | 1.307 | 0.194 | 0.270 |
| ODI-IC | Control (Ref) vs 22q11.2Deletion | -0.412 | | 0.174 | -2.365 | 0.020 | 0.037 |
| ODI-UNC | Control (Ref) vs 22q11.2Deletion | -0.026 | | 0.204 | -0.128 | 0.899 | 0.940 |
| ODI-PCR | Control (Ref) vs 22q11.2Deletion | 0.256 | | 0.190 | 1.343 | 0.182 | 0.262 |
| ODI-PLIC | Control (Ref) vs 22q11.2Deletion | -0.591 | | 0.175 | -3.379 | 0.001 | 0.002 |
| ODI-PTR | Control (Ref) vs 22q11.2Deletion | 0.804 | | 0.171 | 4.695 | 0.000 | 0.000 |
| ODI-RLIC | Control (Ref) vs 22q11.2Deletion | 0.007 | | 0.192 | 0.034 | 0.973 | 0.976 |
| ODI-SCC | Control (Ref) vs 22q11.2Deletion | 0.115 | | 0.189 | 0.610 | 0.543 | 0.636 |
| ODI-SCR | Control (Ref) vs 22q11.2Deletion | -0.175 | | 0.193 | -0.909 | 0.366 | 0.457 |
| ODI-SFO | Control (Ref) vs 22q11.2Deletion | 0.444 | | 0.177 | 2.513 | 0.013 | 0.026 |
| ODI-SLF | Control (Ref) vs 22q11.2Deletion | 0.921 | | 0.184 | 5.005 | 0.000 | 0.000 |
| ODI-SS | Control (Ref) vs 22q11.2Deletion | 0.541 | | 0.201 | 2.695 | 0.008 | 0.017 |
| ODI-TAP | Control (Ref) vs 22q11.2Deletion | -0.127 | | 0.218 | -0.584 | 0.560 | 0.652 |
| ICVF | Control (Ref) vs 22q11.2Duplication | -0.929 | | 0.169 | -5.508 | 0.000 | 0.000 |
| ISO | Control (Ref) vs 22q11.2Duplication | -0.499 | | 0.184 | -2.708 | 0.009 | 0.018 |
| ODI | Control (Ref) vs 22q11.2Duplication | 0.353 | | 0.230 | 1.530 | 0.131 | 0.200 |
| ICVF-ACR | Control (Ref) vs 22q11.2Duplication | -1.052 | | 0.217 | -4.851 | 0.000 | 0.000 |
| ICVF-ALIC | Control (Ref) vs 22q11.2Duplication | -0.625 | | 0.152 | -4.106 | 0.000 | 0.000 |
| ICVF-BCC | Control (Ref) vs 22q11.2Duplication | -1.361 | | 0.207 | -6.592 | 0.000 | 0.000 |
| ICVF-CC | Control (Ref) vs 22q11.2Duplication | -1.340 | | 0.211 | -6.348 | 0.000 | 0.000 |
| ICVF-CGC | Control (Ref) vs 22q11.2Duplication | -0.966 | | 0.176 | -5.484 | 0.000 | 0.000 |
| ICVF-CGH | Control (Ref) vs 22q11.2Duplication | -0.775 | | 0.188 | -4.115 | 0.000 | 0.000 |
| ICVF-CR | Control (Ref) vs 22q11.2Duplication | -1.102 | | 0.208 | -5.296 | 0.000 | 0.000 |
| ICVF-CST | Control (Ref) vs 22q11.2Duplication | -0.596 | | 0.240 | -2.487 | 0.015 | 0.030 |
| ICVF-EC | Control (Ref) vs 22q11.2Duplication | -0.720 | | 0.183 | -3.942 | 0.000 | 0.000 |
| ICVF-FX | Control (Ref) vs 22q11.2Duplication | -0.758 | | 0.251 | -3.024 | 0.004 | 0.008 |
| ICVF-FXST | Control (Ref) vs 22q11.2Duplication | -0.697 | | 0.181 | -3.854 | 0.000 | 0.001 |
| ICVF-GCC | Control (Ref) vs 22q11.2Duplication | -1.134 | | 0.233 | -4.856 | 0.000 | 0.000 |
| ICVF-IC | Control (Ref) vs 22q11.2Duplication | -0.881 | | 0.168 | -5.244 | 0.000 | 0.000 |
| ICVF-UNC | Control (Ref) vs 22q11.2Duplication | -0.563 | | 0.174 | -3.230 | 0.002 | 0.004 |
| ICVF-PCR | Control (Ref) vs 22q11.2Duplication | -1.127 | | 0.208 | -5.409 | 0.000 | 0.000 |
| ICVF-PLIC | Control (Ref) vs 22q11.2Duplication | -0.840 | | 0.175 | -4.802 | 0.000 | 0.000 |
| ICVF-PTR | Control (Ref) vs 22q11.2Duplication | -1.167 | | 0.208 | -5.600 | 0.000 | 0.000 |
| ICVF-RLIC | Control (Ref) vs 22q11.2Duplication | -1.039 | | 0.188 | -5.518 | 0.000 | 0.000 |
| ICVF-SCC | Control (Ref) vs 22q11.2Duplication | -1.229 | | 0.226 | -5.445 | 0.000 | 0.000 |
| ICVF-SCR | Control (Ref) vs 22q11.2Duplication | -1.109 | | 0.204 | -5.432 | 0.000 | 0.000 |
| ICVF-SFO | Control (Ref) vs 22q11.2Duplication | -0.823 | | 0.203 | -4.052 | 0.000 | 0.000 |
| ICVF-SLF | Control (Ref) vs 22q11.2Duplication | -1.119 | | 0.186 | -6.003 | 0.000 | 0.000 |
| ICVF-SS | Control (Ref) vs 22q11.2Duplication | -0.944 | | 0.209 | -4.526 | 0.000 | 0.000 |
| ICVF-TAP | Control (Ref) vs 22q11.2Duplication | -1.062 | | 0.235 | -4.523 | 0.000 | 0.000 |
| ISO-ACR | Control (Ref) vs 22q11.2Duplication | | -0.409 | 0.238 | -1.716 | 0.091 | 0.147 |
| ISO-ALIC | Control (Ref) vs 22q11.2Duplication | | 0.026 | 0.269 | 0.095 | 0.925 | 0.955 |
| ISO-BCC | Control (Ref) vs 22q11.2Duplication | | -0.231 | 0.265 | -0.873 | 0.386 | 0.479 |
| ISO-CC | Control (Ref) vs 22q11.2Duplication | | -0.182 | 0.271 | -0.672 | 0.504 | 0.599 |
| ISO-CGC | Control (Ref) vs 22q11.2Duplication | | -0.310 | 0.247 | -1.256 | 0.214 | 0.287 |
| ISO-CGH | Control (Ref) vs 22q11.2Duplication | | -0.032 | 0.248 | -0.127 | 0.899 | 0.940 |
| ISO-CR | Control (Ref) vs 22q11.2Duplication | | -0.577 | 0.233 | -2.477 | 0.016 | 0.030 |
| ISO-CST | Control (Ref) vs 22q11.2Duplication | | 0.446 | 0.233 | 1.909 | 0.061 | 0.104 |
| ISO-EC | Control (Ref) vs 22q11.2Duplication | | -0.248 | 0.187 | -1.325 | 0.189 | 0.267 |
| ISO-FX | Control (Ref) vs 22q11.2Duplication | | 0.543 | 0.274 | 1.983 | 0.051 | 0.090 |
| ISO-FXST | Control (Ref) vs 22q11.2Duplication | | -0.596 | 0.240 | -2.485 | 0.015 | 0.030 |
| ISO-GCC | Control (Ref) vs 22q11.2Duplication | | -0.306 | 0.258 | -1.183 | 0.241 | 0.320 |
| ISO-IC | Control (Ref) vs 22q11.2Duplication | | -0.239 | 0.244 | -0.980 | 0.330 | 0.420 |
| ISO-UNC | Control (Ref) vs 22q11.2Duplication | | -0.377 | 0.237 | -1.590 | 0.116 | 0.180 |
| ISO-PCR | Control (Ref) vs 22q11.2Duplication | | -0.653 | 0.230 | -2.834 | 0.006 | 0.013 |
| ISO-PLIC | Control (Ref) vs 22q11.2Duplication | | -0.229 | 0.251 | -0.913 | 0.365 | 0.457 |
| ISO-PTR | Control (Ref) vs 22q11.2Duplication | | -0.770 | 0.235 | -3.282 | 0.002 | 0.004 |
| ISO-RLIC | Control (Ref) vs 22q11.2Duplication | | -0.384 | 0.229 | -1.675 | 0.099 | 0.157 |
| ISO-SCC | Control (Ref) vs 22q11.2Duplication | | 0.086 | 0.298 | 0.289 | 0.774 | 0.838 |
| ISO-SCR | Control (Ref) vs 22q11.2Duplication | | -0.650 | 0.227 | -2.863 | 0.006 | 0.012 |
| ISO-SFO | Control (Ref) vs 22q11.2Duplication | | -0.629 | 0.262 | -2.403 | 0.019 | 0.036 |
| ISO-SLF | Control (Ref) vs 22q11.2Duplication | | -0.670 | 0.215 | -3.121 | 0.003 | 0.006 |
| ISO-SS | Control (Ref) vs 22q11.2Duplication | | -0.506 | 0.217 | -2.332 | 0.023 | 0.042 |
| ISO-TAP | Control (Ref) vs 22q11.2Duplication | | -0.471 | 0.265 | -1.776 | 0.080 | 0.132 |
| ODI-ACR | Control (Ref) vs 22q11.2Duplication | | 0.026 | 0.266 | 0.098 | 0.923 | 0.955 |
| ODI-ALIC | Control (Ref) vs 22q11.2Duplication | | 0.661 | 0.236 | 2.805 | 0.007 | 0.014 |
| ODI-BCC | Control (Ref) vs 22q11.2Duplication | | 0.261 | 0.259 | 1.007 | 0.317 | 0.407 |
| ODI-CC | Control (Ref) vs 22q11.2Duplication | | -0.018 | 0.261 | -0.070 | 0.945 | 0.964 |
| ODI-CGC | Control (Ref) vs 22q11.2Duplication | | 0.404 | 0.265 | 1.524 | 0.132 | 0.201 |
| ODI-CGH | Control (Ref) vs 22q11.2Duplication | | -0.327 | 0.259 | -1.264 | 0.210 | 0.284 |
| ODI-CR | Control (Ref) vs 22q11.2Duplication | | -0.283 | 0.253 | -1.118 | 0.268 | 0.349 |
| ODI-CST | Control (Ref) vs 22q11.2Duplication | | 0.310 | 0.265 | 1.170 | 0.246 | 0.325 |
| ODI-EC | Control (Ref) vs 22q11.2Duplication | | 0.551 | 0.255 | 2.163 | 0.034 | 0.062 |
| ODI-FX | Control (Ref) vs 22q11.2Duplication | | 0.014 | 0.260 | 0.053 | 0.958 | 0.973 |
| ODI-FXST | Control (Ref) vs 22q11.2Duplication | | -0.079 | 0.251 | -0.315 | 0.754 | 0.823 |
| ODI-GCC | Control (Ref) vs 22q11.2Duplication | | -0.087 | 0.265 | -0.327 | 0.744 | 0.818 |
| ODI-IC | Control (Ref) vs 22q11.2Duplication | | 0.317 | 0.231 | 1.370 | 0.175 | 0.254 |
| ODI-UNC | Control (Ref) vs 22q11.2Duplication | | 0.734 | 0.281 | 2.613 | 0.011 | 0.022 |
| ODI-PCR | Control (Ref) vs 22q11.2Duplication | | -0.538 | 0.259 | -2.075 | 0.042 | 0.073 |
| ODI-PLIC | Control (Ref) vs 22q11.2Duplication | | 0.340 | 0.233 | 1.458 | 0.149 | 0.224 |
| ODI-PTR | Control (Ref) vs 22q11.2Duplication | | 0.009 | 0.267 | 0.034 | 0.973 | 0.976 |
| ODI-RLIC | Control (Ref) vs 22q11.2Duplication | | -0.392 | 0.251 | -1.561 | 0.123 | 0.189 |
| ODI-SCC | Control (Ref) vs 22q11.2Duplication | | -0.219 | 0.268 | -0.818 | 0.416 | 0.507 |
| ODI-SCR | Control (Ref) vs 22q11.2Duplication | | -0.471 | 0.249 | -1.890 | 0.063 | 0.106 |
| ODI-SFO | Control (Ref) vs 22q11.2Duplication | | -0.002 | 0.262 | -0.007 | 0.995 | 0.995 |
| ODI-SLF | Control (Ref) vs 22q11.2Duplication | | 0.014 | 0.280 | 0.051 | 0.960 | 0.973 |
| ODI-SS | Control (Ref) vs 22q11.2Duplication | | -0.524 | 0.279 | -1.879 | 0.064 | 0.107 |
| ODI-TAP | Control (Ref) vs 22q11.2Duplication | | 0.284 | 0.288 | 0.988 | 0.327 | 0.417 |
| ICVF | 22q11.2Deletion (Ref) vs 22q11.2Duplication | | -1.372 | 0.139 | -9.856 | 0.000 | 0.000 |
| ISO | 22q11.2Deletion (Ref) vs 22q11.2Duplication | | -0.713 | 0.159 | -4.483 | 0.000 | 0.000 |
| ODI | 22q11.2Deletion (Ref) vs 22q11.2Duplication | | 0.260 | 0.206 | 1.264 | 0.209 | 0.284 |
| ICVF-ACR | 22q11.2Deletion (Ref) vs 22q11.2Duplication | | -1.568 | 0.152 | -10.350 | 0.000 | 0.000 |
| ICVF-ALIC | 22q11.2Deletion (Ref) vs 22q11.2Duplication | | -1.207 | 0.123 | -9.804 | 0.000 | 0.000 |
| ICVF-BCC | 22q11.2Deletion (Ref) vs 22q11.2Duplication | | -1.670 | 0.144 | -11.594 | 0.000 | 0.000 |
| ICVF-CC | 22q11.2Deletion (Ref) vs 22q11.2Duplication | | -1.645 | 0.147 | -11.171 | 0.000 | 0.000 |
| ICVF-CGC | 22q11.2Deletion (Ref) vs 22q11.2Duplication | | -1.362 | 0.150 | -9.088 | 0.000 | 0.000 |
| ICVF-CGH | 22q11.2Deletion (Ref) vs 22q11.2Duplication | | -0.920 | 0.180 | -5.107 | 0.000 | 0.000 |
| ICVF-CR | 22q11.2Deletion (Ref) vs 22q11.2Duplication | | -1.556 | 0.155 | -10.040 | 0.000 | 0.000 |
| ICVF-CST | 22q11.2Deletion (Ref) vs 22q11.2Duplication | | -0.743 | 0.202 | -3.681 | 0.000 | 0.001 |
| ICVF-EC | 22q11.2Deletion (Ref) vs 22q11.2Duplication | | -1.167 | 0.138 | -8.428 | 0.000 | 0.000 |
| ICVF-FX | 22q11.2Deletion (Ref) vs 22q11.2Duplication | | -0.705 | 0.231 | -3.053 | 0.003 | 0.007 |
| ICVF-FXST | 22q11.2Deletion (Ref) vs 22q11.2Duplication | | -1.201 | 0.155 | -7.748 | 0.000 | 0.000 |
| ICVF-GCC | 22q11.2Deletion (Ref) vs 22q11.2Duplication | | -1.649 | 0.151 | -10.951 | 0.000 | 0.000 |
| ICVF-IC | 22q11.2Deletion (Ref) vs 22q11.2Duplication | | -1.367 | 0.141 | -9.704 | 0.000 | 0.000 |
| ICVF-UNC | 22q11.2Deletion (Ref) vs 22q11.2Duplication | | -1.201 | 0.152 | -7.892 | 0.000 | 0.000 |
| ICVF-PCR | 22q11.2Deletion (Ref) vs 22q11.2Duplication | | -1.520 | 0.160 | -9.474 | 0.000 | 0.000 |
| ICVF-PLIC | 22q11.2Deletion (Ref) vs 22q11.2Duplication | | -1.172 | 0.166 | -7.061 | 0.000 | 0.000 |
| ICVF-PTR | 22q11.2Deletion (Ref) vs 22q11.2Duplication | | -1.595 | 0.159 | -10.039 | 0.000 | 0.000 |
| ICVF-RLIC | 22q11.2Deletion (Ref) vs 22q11.2Duplication | | -1.513 | 0.143 | -10.571 | 0.000 | 0.000 |
| ICVF-SCC | 22q11.2Deletion (Ref) vs 22q11.2Duplication | | -1.475 | 0.170 | -8.680 | 0.000 | 0.000 |
| ICVF-SCR | 22q11.2Deletion (Ref) vs 22q11.2Duplication | | -1.513 | 0.157 | -9.617 | 0.000 | 0.000 |
| ICVF-SFO | 22q11.2Deletion (Ref) vs 22q11.2Duplication | | -1.336 | 0.161 | -8.314 | 0.000 | 0.000 |
| ICVF-SLF | 22q11.2Deletion (Ref) vs 22q11.2Duplication | | -1.518 | 0.146 | -10.400 | 0.000 | 0.000 |
| ICVF-SS | 22q11.2Deletion (Ref) vs 22q11.2Duplication | | -1.521 | 0.149 | -10.196 | 0.000 | 0.000 |
| ICVF-TAP | 22q11.2Deletion (Ref) vs 22q11.2Duplication | | -1.473 | 0.183 | -8.042 | 0.000 | 0.000 |
| ISO-ACR | 22q11.2Deletion (Ref) vs 22q11.2Duplication | | -0.825 | 0.190 | -4.341 | 0.000 | 0.000 |
| ISO-ALIC | 22q11.2Deletion (Ref) vs 22q11.2Duplication | | -0.204 | 0.237 | -0.862 | 0.391 | 0.479 |
| ISO-BCC | 22q11.2Deletion (Ref) vs 22q11.2Duplication | | -0.293 | 0.222 | -1.319 | 0.190 | 0.267 |
| ISO-CC | 22q11.2Deletion (Ref) vs 22q11.2Duplication | | -0.020 | 0.239 | -0.085 | 0.932 | 0.955 |
| ISO-CGC | 22q11.2Deletion (Ref) vs 22q11.2Duplication | | -0.386 | 0.203 | -1.897 | 0.061 | 0.104 |
| ISO-CGH | 22q11.2Deletion (Ref) vs 22q11.2Duplication | | -0.294 | 0.202 | -1.455 | 0.149 | 0.224 |
| ISO-CR | 22q11.2Deletion (Ref) vs 22q11.2Duplication | | -0.897 | 0.185 | -4.856 | 0.000 | 0.000 |
| ISO-CST | 22q11.2Deletion (Ref) vs 22q11.2Duplication | | 0.406 | 0.215 | 1.885 | 0.063 | 0.106 |
| ISO-EC | 22q11.2Deletion (Ref) vs 22q11.2Duplication | | -0.073 | 0.188 | -0.390 | 0.698 | 0.778 |
| ISO-FX | 22q11.2Deletion (Ref) vs 22q11.2Duplication | | -0.050 | 0.259 | -0.194 | 0.846 | 0.903 |
| ISO-FXST | 22q11.2Deletion (Ref) vs 22q11.2Duplication | | -0.298 | 0.225 | -1.325 | 0.188 | 0.267 |
| ISO-GCC | 22q11.2Deletion (Ref) vs 22q11.2Duplication | | -0.149 | 0.234 | -0.639 | 0.524 | 0.617 |
| ISO-IC | 22q11.2Deletion (Ref) vs 22q11.2Duplication | | -0.319 | 0.198 | -1.612 | 0.111 | 0.174 |
| ISO-UNC | 22q11.2Deletion (Ref) vs 22q11.2Duplication | | -0.300 | 0.218 | -1.378 | 0.171 | 0.251 |
| ISO-PCR | 22q11.2Deletion (Ref) vs 22q11.2Duplication | | -0.920 | 0.186 | -4.939 | 0.000 | 0.000 |
| ISO-PLIC | 22q11.2Deletion (Ref) vs 22q11.2Duplication | | -0.148 | 0.221 | -0.668 | 0.505 | 0.599 |
| ISO-PTR | 22q11.2Deletion (Ref) vs 22q11.2Duplication | | -0.956 | 0.192 | -4.974 | 0.000 | 0.000 |
| ISO-RLIC | 22q11.2Deletion (Ref) vs 22q11.2Duplication | | -0.475 | 0.181 | -2.620 | 0.010 | 0.021 |
| ISO-SCC | 22q11.2Deletion (Ref) vs 22q11.2Duplication | | 0.331 | 0.252 | 1.316 | 0.192 | 0.267 |
| ISO-SCR | 22q11.2Deletion (Ref) vs 22q11.2Duplication | | -0.849 | 0.193 | -4.395 | 0.000 | 0.000 |
| ISO-SFO | 22q11.2Deletion (Ref) vs 22q11.2Duplication | | -0.864 | 0.203 | -4.267 | 0.000 | 0.000 |
| ISO-SLF | 22q11.2Deletion (Ref) vs 22q11.2Duplication | | -0.887 | 0.177 | -5.003 | 0.000 | 0.000 |
| ISO-SS | 22q11.2Deletion (Ref) vs 22q11.2Duplication | | -0.711 | 0.179 | -3.980 | 0.000 | 0.000 |
| ISO-TAP | 22q11.2Deletion (Ref) vs 22q11.2Duplication | | -0.816 | 0.214 | -3.807 | 0.000 | 0.001 |
| ODI-ACR | 22q11.2Deletion (Ref) vs 22q11.2Duplication | | -0.103 | 0.234 | -0.441 | 0.660 | 0.752 |
| ODI-ALIC | 22q11.2Deletion (Ref) vs 22q11.2Duplication | | 1.117 | 0.197 | 5.675 | 0.000 | 0.000 |
| ODI-BCC | 22q11.2Deletion (Ref) vs 22q11.2Duplication | | 0.370 | 0.232 | 1.597 | 0.114 | 0.177 |
| ODI-CC | 22q11.2Deletion (Ref) vs 22q11.2Duplication | | 0.073 | 0.235 | 0.310 | 0.757 | 0.823 |
| ODI-CGC | 22q11.2Deletion (Ref) vs 22q11.2Duplication | | 0.606 | 0.235 | 2.575 | 0.012 | 0.023 |
| ODI-CGH | 22q11.2Deletion (Ref) vs 22q11.2Duplication | | -0.667 | 0.231 | -2.884 | 0.005 | 0.010 |
| ODI-CR | 22q11.2Deletion (Ref) vs 22q11.2Duplication | | -0.236 | 0.232 | -1.016 | 0.312 | 0.402 |
| ODI-CST | 22q11.2Deletion (Ref) vs 22q11.2Duplication | | 0.837 | 0.214 | 3.907 | 0.000 | 0.000 |
| ODI-EC | 22q11.2Deletion (Ref) vs 22q11.2Duplication | | -0.118 | 0.234 | -0.503 | 0.616 | 0.708 |
| ODI-FX | 22q11.2Deletion (Ref) vs 22q11.2Duplication | | 0.418 | 0.251 | 1.667 | 0.099 | 0.157 |
| ODI-FXST | 22q11.2Deletion (Ref) vs 22q11.2Duplication | | -0.454 | 0.220 | -2.068 | 0.041 | 0.073 |
| ODI-GCC | 22q11.2Deletion (Ref) vs 22q11.2Duplication | | -0.091 | 0.250 | -0.365 | 0.716 | 0.790 |
| ODI-IC | 22q11.2Deletion (Ref) vs 22q11.2Duplication | | 0.768 | 0.209 | 3.670 | 0.000 | 0.001 |
| ODI-UNC | 22q11.2Deletion (Ref) vs 22q11.2Duplication | | 0.627 | 0.247 | 2.543 | 0.013 | 0.025 |
| ODI-PCR | 22q11.2Deletion (Ref) vs 22q11.2Duplication | | -0.669 | 0.231 | -2.899 | 0.005 | 0.010 |
| ODI-PLIC | 22q11.2Deletion (Ref) vs 22q11.2Duplication | | 1.039 | 0.218 | 4.762 | 0.000 | 0.000 |
| ODI-PTR | 22q11.2Deletion (Ref) vs 22q11.2Duplication | | -0.916 | 0.212 | -4.325 | 0.000 | 0.000 |
| ODI-RLIC | 22q11.2Deletion (Ref) vs 22q11.2Duplication | | -0.379 | 0.221 | -1.717 | 0.089 | 0.146 |
| ODI-SCC | 22q11.2Deletion (Ref) vs 22q11.2Duplication | | -0.170 | 0.243 | -0.700 | 0.486 | 0.586 |
| ODI-SCR | 22q11.2Deletion (Ref) vs 22q11.2Duplication | | -0.099 | 0.247 | -0.399 | 0.691 | 0.776 |
| ODI-SFO | 22q11.2Deletion (Ref) vs 22q11.2Duplication | | -0.306 | 0.240 | -1.271 | 0.207 | 0.284 |
| ODI-SLF | 22q11.2Deletion (Ref) vs 22q11.2Duplication | | -0.805 | 0.227 | -3.548 | 0.001 | 0.001 |
| ODI-SS | 22q11.2Deletion (Ref) vs 22q11.2Duplication | | -0.943 | 0.226 | -4.177 | 0.000 | 0.000 |
| ODI-TAP | 22q11.2Deletion (Ref) vs 22q11.2Duplication | | 0.227 | 0.261 | 0.867 | 0.388 | 0.479 |
| ICVF | GeneDosage (1 = del, 2 = con, 3 dup) | | -0.686 | 0.070 | -9.856 | 0.000 | 0.000 |
| ISO | GeneDosage (1 = del, 2 = con, 3 dup) | | -0.357 | 0.080 | -4.483 | 0.000 | 0.000 |
| ODI | GeneDosage (1 = del, 2 = con, 3 dup) | | 0.130 | 0.103 | 1.264 | 0.209 | 0.284 |
| ICVF-ACR | GeneDosage (1 = del, 2 = con, 3 dup) | | -0.784 | 0.076 | -10.350 | 0.000 | 0.000 |
| ICVF-ALIC | GeneDosage (1 = del, 2 = con, 3 dup) | | -0.603 | 0.062 | -9.804 | 0.000 | 0.000 |
| ICVF-BCC | GeneDosage (1 = del, 2 = con, 3 dup) | | -0.835 | 0.072 | -11.594 | 0.000 | 0.000 |
| ICVF-CC | GeneDosage (1 = del, 2 = con, 3 dup) | | -0.822 | 0.074 | -11.171 | 0.000 | 0.000 |
| ICVF-CGC | GeneDosage (1 = del, 2 = con, 3 dup) | | -0.681 | 0.075 | -9.088 | 0.000 | 0.000 |
| ICVF-CGH | GeneDosage (1 = del, 2 = con, 3 dup) | | -0.460 | 0.090 | -5.107 | 0.000 | 0.000 |
| ICVF-CR | GeneDosage (1 = del, 2 = con, 3 dup) | | -0.778 | 0.077 | -10.040 | 0.000 | 0.000 |
| ICVF-CST | GeneDosage (1 = del, 2 = con, 3 dup) | | -0.371 | 0.101 | -3.681 | 0.000 | 0.001 |
| ICVF-EC | GeneDosage (1 = del, 2 = con, 3 dup) | | -0.584 | 0.069 | -8.428 | 0.000 | 0.000 |
| ICVF-FX | GeneDosage (1 = del, 2 = con, 3 dup) | | -0.353 | 0.115 | -3.054 | 0.003 | 0.007 |
| ICVF-FXST | GeneDosage (1 = del, 2 = con, 3 dup) | | -0.600 | 0.077 | -7.748 | 0.000 | 0.000 |
| ICVF-GCC | GeneDosage (1 = del, 2 = con, 3 dup) | | -0.824 | 0.075 | -10.951 | 0.000 | 0.000 |
| ICVF-IC | GeneDosage (1 = del, 2 = con, 3 dup) | | -0.683 | 0.070 | -9.704 | 0.000 | 0.000 |
| ICVF-UNC | GeneDosage (1 = del, 2 = con, 3 dup) | | -0.601 | 0.076 | -7.892 | 0.000 | 0.000 |
| ICVF-PCR | GeneDosage (1 = del, 2 = con, 3 dup) | | -0.760 | 0.080 | -9.474 | 0.000 | 0.000 |
| ICVF-PLIC | GeneDosage (1 = del, 2 = con, 3 dup) | | -0.586 | 0.083 | -7.061 | 0.000 | 0.000 |
| ICVF-PTR | GeneDosage (1 = del, 2 = con, 3 dup) | | -0.797 | 0.079 | -10.039 | 0.000 | 0.000 |
| ICVF-RLIC | GeneDosage (1 = del, 2 = con, 3 dup) | | -0.756 | 0.072 | -10.571 | 0.000 | 0.000 |
| ICVF-SCC | GeneDosage (1 = del, 2 = con, 3 dup) | | -0.738 | 0.085 | -8.680 | 0.000 | 0.000 |
| ICVF-SCR | GeneDosage (1 = del, 2 = con, 3 dup) | | -0.756 | 0.079 | -9.618 | 0.000 | 0.000 |
| ICVF-SFO | GeneDosage (1 = del, 2 = con, 3 dup) | | -0.668 | 0.080 | -8.314 | 0.000 | 0.000 |
| ICVF-SLF | GeneDosage (1 = del, 2 = con, 3 dup) | | -0.759 | 0.073 | -10.400 | 0.000 | 0.000 |
| ICVF-SS | GeneDosage (1 = del, 2 = con, 3 dup) | | -0.761 | 0.075 | -10.194 | 0.000 | 0.000 |
| ICVF-TAP | GeneDosage (1 = del, 2 = con, 3 dup) | | -0.737 | 0.092 | -8.042 | 0.000 | 0.000 |
| ISO-ACR | GeneDosage (1 = del, 2 = con, 3 dup) | | -0.412 | 0.095 | -4.341 | 0.000 | 0.000 |
| ISO-ALIC | GeneDosage (1 = del, 2 = con, 3 dup) | | -0.102 | 0.119 | -0.862 | 0.391 | 0.479 |
| ISO-BCC | GeneDosage (1 = del, 2 = con, 3 dup) | | -0.146 | 0.111 | -1.319 | 0.190 | 0.267 |
| ISO-CC | GeneDosage (1 = del, 2 = con, 3 dup) | | -0.010 | 0.119 | -0.085 | 0.932 | 0.955 |
| ISO-CGC | GeneDosage (1 = del, 2 = con, 3 dup) | | -0.193 | 0.102 | -1.897 | 0.061 | 0.104 |
| ISO-CGH | GeneDosage (1 = del, 2 = con, 3 dup) | | -0.147 | 0.101 | -1.455 | 0.149 | 0.224 |
| ISO-CR | GeneDosage (1 = del, 2 = con, 3 dup) | | -0.448 | 0.092 | -4.856 | 0.000 | 0.000 |
| ISO-CST | GeneDosage (1 = del, 2 = con, 3 dup) | | 0.203 | 0.108 | 1.885 | 0.063 | 0.106 |
| ISO-EC | GeneDosage (1 = del, 2 = con, 3 dup) | | -0.037 | 0.094 | -0.390 | 0.697 | 0.778 |
| ISO-FX | GeneDosage (1 = del, 2 = con, 3 dup) | | -0.025 | 0.130 | -0.194 | 0.846 | 0.903 |
| ISO-FXST | GeneDosage (1 = del, 2 = con, 3 dup) | | -0.149 | 0.112 | -1.325 | 0.188 | 0.267 |
| ISO-GCC | GeneDosage (1 = del, 2 = con, 3 dup) | | -0.075 | 0.117 | -0.639 | 0.524 | 0.617 |
| ISO-IC | GeneDosage (1 = del, 2 = con, 3 dup) | | -0.160 | 0.099 | -1.612 | 0.111 | 0.174 |
| ISO-UNC | GeneDosage (1 = del, 2 = con, 3 dup) | | -0.150 | 0.109 | -1.378 | 0.171 | 0.251 |
| ISO-PCR | GeneDosage (1 = del, 2 = con, 3 dup) | | -0.460 | 0.093 | -4.939 | 0.000 | 0.000 |
| ISO-PLIC | GeneDosage (1 = del, 2 = con, 3 dup) | | -0.074 | 0.110 | -0.668 | 0.505 | 0.599 |
| ISO-PTR | GeneDosage (1 = del, 2 = con, 3 dup) | | -0.478 | 0.096 | -4.974 | 0.000 | 0.000 |
| ISO-RLIC | GeneDosage (1 = del, 2 = con, 3 dup) | | -0.238 | 0.091 | -2.620 | 0.010 | 0.021 |
| ISO-SCC | GeneDosage (1 = del, 2 = con, 3 dup) | | 0.166 | 0.126 | 1.316 | 0.192 | 0.267 |
| ISO-SCR | GeneDosage (1 = del, 2 = con, 3 dup) | | -0.424 | 0.097 | -4.395 | 0.000 | 0.000 |
| ISO-SFO | GeneDosage (1 = del, 2 = con, 3 dup) | | -0.432 | 0.101 | -4.267 | 0.000 | 0.000 |
| ISO-SLF | GeneDosage (1 = del, 2 = con, 3 dup) | | -0.444 | 0.089 | -5.003 | 0.000 | 0.000 |
| ISO-SS | GeneDosage (1 = del, 2 = con, 3 dup) | | -0.356 | 0.089 | -3.980 | 0.000 | 0.000 |
| ISO-TAP | GeneDosage (1 = del, 2 = con, 3 dup) | | -0.408 | 0.107 | -3.807 | 0.000 | 0.001 |
| ODI-ACR | GeneDosage (1 = del, 2 = con, 3 dup) | | -0.052 | 0.117 | -0.441 | 0.660 | 0.752 |
| ODI-ALIC | GeneDosage (1 = del, 2 = con, 3 dup) | | 0.559 | 0.098 | 5.675 | 0.000 | 0.000 |
| ODI-BCC | GeneDosage (1 = del, 2 = con, 3 dup) | | 0.185 | 0.116 | 1.597 | 0.114 | 0.177 |
| ODI-CC | GeneDosage (1 = del, 2 = con, 3 dup) | | 0.036 | 0.118 | 0.310 | 0.757 | 0.823 |
| ODI-CGC | GeneDosage (1 = del, 2 = con, 3 dup) | | 0.303 | 0.118 | 2.577 | 0.012 | 0.023 |
| ODI-CGH | GeneDosage (1 = del, 2 = con, 3 dup) | | -0.333 | 0.116 | -2.884 | 0.005 | 0.010 |
| ODI-CR | GeneDosage (1 = del, 2 = con, 3 dup) | | -0.118 | 0.116 | -1.016 | 0.312 | 0.402 |
| ODI-CST | GeneDosage (1 = del, 2 = con, 3 dup) | | 0.418 | 0.107 | 3.907 | 0.000 | 0.000 |
| ODI-EC | GeneDosage (1 = del, 2 = con, 3 dup) | | -0.059 | 0.117 | -0.503 | 0.616 | 0.708 |
| ODI-FX | GeneDosage (1 = del, 2 = con, 3 dup) | | 0.209 | 0.125 | 1.667 | 0.099 | 0.157 |
| ODI-FXST | GeneDosage (1 = del, 2 = con, 3 dup) | | -0.227 | 0.110 | -2.068 | 0.041 | 0.073 |
| ODI-GCC | GeneDosage (1 = del, 2 = con, 3 dup) | | -0.046 | 0.125 | -0.365 | 0.716 | 0.790 |
| ODI-IC | GeneDosage (1 = del, 2 = con, 3 dup) | | 0.384 | 0.105 | 3.670 | 0.000 | 0.001 |
| ODI-UNC | GeneDosage (1 = del, 2 = con, 3 dup) | | 0.314 | 0.123 | 2.543 | 0.013 | 0.025 |
| ODI-PCR | GeneDosage (1 = del, 2 = con, 3 dup) | | -0.335 | 0.115 | -2.899 | 0.005 | 0.010 |
| ODI-PLIC | GeneDosage (1 = del, 2 = con, 3 dup) | | 0.520 | 0.109 | 4.762 | 0.000 | 0.000 |
| ODI-PTR | GeneDosage (1 = del, 2 = con, 3 dup) | | -0.458 | 0.106 | -4.325 | 0.000 | 0.000 |
| ODI-RLIC | GeneDosage (1 = del, 2 = con, 3 dup) | | -0.189 | 0.110 | -1.717 | 0.089 | 0.146 |
| ODI-SCC | GeneDosage (1 = del, 2 = con, 3 dup) | | -0.085 | 0.122 | -0.700 | 0.486 | 0.586 |
| ODI-SCR | GeneDosage (1 = del, 2 = con, 3 dup) | | -0.049 | 0.124 | -0.399 | 0.691 | 0.776 |
| ODI-SFO | GeneDosage (1 = del, 2 = con, 3 dup) | | -0.153 | 0.120 | -1.271 | 0.207 | 0.284 |
| ODI-SLF | GeneDosage (1 = del, 2 = con, 3 dup) | | -0.402 | 0.113 | -3.548 | 0.001 | 0.001 |
| ODI-SS | GeneDosage (1 = del, 2 = con, 3 dup) | | -0.471 | 0.113 | -4.177 | 0.000 | 0.000 |
| ODI-TAP | GeneDosage (1 = del, 2 = con, 3 dup) | | 0.113 | 0.131 | 0.867 | 0.388 | 0.479 |

| sTable 6. Sensitivity analysis - Group differences across individuals scanned at the same scanner site, adjusted for white matter volume | | | | | | |
| --- | --- | --- | --- | --- | --- | --- |
| Dependent variable | **Analysis** | **Std.B.** | **SE** | **T-value** | **P-value** | **pFDR** |
| ICVF | Control (Ref) vs 22q11.2Deletion | 0.804 | 0.142 | 5.677 | 0.000 | 0.000 |
| ISO | Control (Ref) vs 22q11.2Deletion | 0.250 | 0.132 | 1.903 | 0.060 | 0.097 |
| ODI | Control (Ref) vs 22q11.2Deletion | 0.160 | 0.171 | 0.936 | 0.351 | 0.437 |
| ICVF-ACR | Control (Ref) vs 22q11.2Deletion | 1.044 | 0.165 | 6.340 | 0.000 | 0.000 |
| ICVF-ALIC | Control (Ref) vs 22q11.2Deletion | 0.629 | 0.121 | 5.174 | 0.000 | 0.000 |
| ICVF-BCC | Control (Ref) vs 22q11.2Deletion | 1.142 | 0.178 | 6.422 | 0.000 | 0.000 |
| ICVF-CC | Control (Ref) vs 22q11.2Deletion | 1.133 | 0.180 | 6.287 | 0.000 | 0.000 |
| ICVF-CGC | Control (Ref) vs 22q11.2Deletion | 0.740 | 0.149 | 4.961 | 0.000 | 0.000 |
| ICVF-CGH | Control (Ref) vs 22q11.2Deletion | 0.354 | 0.164 | 2.159 | 0.033 | 0.059 |
| ICVF-CR | Control (Ref) vs 22q11.2Deletion | 1.013 | 0.173 | 5.860 | 0.000 | 0.000 |
| ICVF-CST | Control (Ref) vs 22q11.2Deletion | 0.473 | 0.186 | 2.546 | 0.012 | 0.024 |
| ICVF-EC | Control (Ref) vs 22q11.2Deletion | 0.630 | 0.134 | 4.719 | 0.000 | 0.000 |
| ICVF-FX | Control (Ref) vs 22q11.2Deletion | 0.286 | 0.207 | 1.381 | 0.170 | 0.238 |
| ICVF-FXST | Control (Ref) vs 22q11.2Deletion | 0.810 | 0.157 | 5.172 | 0.000 | 0.000 |
| ICVF-GCC | Control (Ref) vs 22q11.2Deletion | 1.130 | 0.172 | 6.560 | 0.000 | 0.000 |
| ICVF-IC | Control (Ref) vs 22q11.2Deletion | 0.864 | 0.141 | 6.116 | 0.000 | 0.000 |
| ICVF-UNC | Control (Ref) vs 22q11.2Deletion | 0.876 | 0.139 | 6.282 | 0.000 | 0.000 |
| ICVF-PCR | Control (Ref) vs 22q11.2Deletion | 1.058 | 0.183 | 5.795 | 0.000 | 0.000 |
| ICVF-PLIC | Control (Ref) vs 22q11.2Deletion | 0.737 | 0.161 | 4.588 | 0.000 | 0.000 |
| ICVF-PTR | Control (Ref) vs 22q11.2Deletion | 1.041 | 0.189 | 5.508 | 0.000 | 0.000 |
| ICVF-RLIC | Control (Ref) vs 22q11.2Deletion | 0.957 | 0.178 | 5.372 | 0.000 | 0.000 |
| ICVF-SCC | Control (Ref) vs 22q11.2Deletion | 0.984 | 0.201 | 4.905 | 0.000 | 0.000 |
| ICVF-SCR | Control (Ref) vs 22q11.2Deletion | 0.900 | 0.179 | 5.013 | 0.000 | 0.000 |
| ICVF-SFO | Control (Ref) vs 22q11.2Deletion | 0.619 | 0.158 | 3.925 | 0.000 | 0.000 |
| ICVF-SLF | Control (Ref) vs 22q11.2Deletion | 0.975 | 0.167 | 5.854 | 0.000 | 0.000 |
| ICVF-SS | Control (Ref) vs 22q11.2Deletion | 1.079 | 0.172 | 6.264 | 0.000 | 0.000 |
| ICVF-TAP | Control (Ref) vs 22q11.2Deletion | 0.933 | 0.205 | 4.561 | 0.000 | 0.000 |
| ISO-ACR | Control (Ref) vs 22q11.2Deletion | 0.386 | 0.177 | 2.176 | 0.032 | 0.057 |
| ISO-ALIC | Control (Ref) vs 22q11.2Deletion | 0.070 | 0.199 | 0.353 | 0.725 | 0.794 |
| ISO-BCC | Control (Ref) vs 22q11.2Deletion | -0.066 | 0.201 | -0.330 | 0.742 | 0.809 |
| ISO-CC | Control (Ref) vs 22q11.2Deletion | -0.076 | 0.195 | -0.389 | 0.698 | 0.773 |
| ISO-CGC | Control (Ref) vs 22q11.2Deletion | 0.052 | 0.175 | 0.296 | 0.768 | 0.831 |
| ISO-CGH | Control (Ref) vs 22q11.2Deletion | 0.372 | 0.183 | 2.030 | 0.045 | 0.076 |
| ISO-CR | Control (Ref) vs 22q11.2Deletion | 0.340 | 0.168 | 2.022 | 0.046 | 0.076 |
| ISO-CST | Control (Ref) vs 22q11.2Deletion | 0.024 | 0.187 | 0.130 | 0.896 | 0.934 |
| ISO-EC | Control (Ref) vs 22q11.2Deletion | 0.067 | 0.167 | 0.404 | 0.687 | 0.763 |
| ISO-FX | Control (Ref) vs 22q11.2Deletion | 0.562 | 0.205 | 2.737 | 0.007 | 0.015 |
| ISO-FXST | Control (Ref) vs 22q11.2Deletion | -0.321 | 0.206 | -1.556 | 0.123 | 0.179 |
| ISO-GCC | Control (Ref) vs 22q11.2Deletion | 0.100 | 0.204 | 0.491 | 0.624 | 0.701 |
| ISO-IC | Control (Ref) vs 22q11.2Deletion | 0.004 | 0.165 | 0.023 | 0.982 | 0.982 |
| ISO-UNC | Control (Ref) vs 22q11.2Deletion | 0.077 | 0.186 | 0.413 | 0.681 | 0.759 |
| ISO-PCR | Control (Ref) vs 22q11.2Deletion | 0.369 | 0.163 | 2.263 | 0.026 | 0.048 |
| ISO-PLIC | Control (Ref) vs 22q11.2Deletion | -0.185 | 0.183 | -1.011 | 0.314 | 0.396 |
| ISO-PTR | Control (Ref) vs 22q11.2Deletion | 0.403 | 0.172 | 2.349 | 0.021 | 0.039 |
| ISO-RLIC | Control (Ref) vs 22q11.2Deletion | 0.117 | 0.166 | 0.707 | 0.481 | 0.562 |
| ISO-SCC | Control (Ref) vs 22q11.2Deletion | -0.193 | 0.176 | -1.096 | 0.275 | 0.351 |
| ISO-SCR | Control (Ref) vs 22q11.2Deletion | 0.160 | 0.176 | 0.911 | 0.364 | 0.452 |
| ISO-SFO | Control (Ref) vs 22q11.2Deletion | 0.127 | 0.204 | 0.622 | 0.535 | 0.620 |
| ISO-SLF | Control (Ref) vs 22q11.2Deletion | 0.251 | 0.143 | 1.756 | 0.082 | 0.124 |
| ISO-SS | Control (Ref) vs 22q11.2Deletion | 0.212 | 0.180 | 1.178 | 0.241 | 0.313 |
| ISO-TAP | Control (Ref) vs 22q11.2Deletion | 0.377 | 0.198 | 1.910 | 0.059 | 0.096 |
| ODI-ACR | Control (Ref) vs 22q11.2Deletion | 0.244 | 0.198 | 1.234 | 0.220 | 0.294 |
| ODI-ALIC | Control (Ref) vs 22q11.2Deletion | -0.308 | 0.194 | -1.588 | 0.115 | 0.170 |
| ODI-BCC | Control (Ref) vs 22q11.2Deletion | 0.337 | 0.185 | 1.829 | 0.070 | 0.112 |
| ODI-CC | Control (Ref) vs 22q11.2Deletion | 0.322 | 0.182 | 1.771 | 0.079 | 0.123 |
| ODI-CGC | Control (Ref) vs 22q11.2Deletion | -0.124 | 0.219 | -0.568 | 0.571 | 0.659 |
| ODI-CGH | Control (Ref) vs 22q11.2Deletion | 0.430 | 0.213 | 2.015 | 0.046 | 0.077 |
| ODI-CR | Control (Ref) vs 22q11.2Deletion | 0.151 | 0.193 | 0.781 | 0.436 | 0.517 |
| ODI-CST | Control (Ref) vs 22q11.2Deletion | -0.295 | 0.215 | -1.370 | 0.174 | 0.242 |
| ODI-EC | Control (Ref) vs 22q11.2Deletion | 0.447 | 0.184 | 2.427 | 0.017 | 0.032 |
| ODI-FX | Control (Ref) vs 22q11.2Deletion | -0.045 | 0.224 | -0.202 | 0.840 | 0.891 |
| ODI-FXST | Control (Ref) vs 22q11.2Deletion | 0.432 | 0.198 | 2.176 | 0.032 | 0.057 |
| ODI-GCC | Control (Ref) vs 22q11.2Deletion | 0.358 | 0.204 | 1.755 | 0.082 | 0.124 |
| ODI-IC | Control (Ref) vs 22q11.2Deletion | -0.319 | 0.181 | -1.761 | 0.081 | 0.124 |
| ODI-UNC | Control (Ref) vs 22q11.2Deletion | -0.119 | 0.214 | -0.555 | 0.580 | 0.664 |
| ODI-PCR | Control (Ref) vs 22q11.2Deletion | 0.361 | 0.198 | 1.823 | 0.071 | 0.112 |
| ODI-PLIC | Control (Ref) vs 22q11.2Deletion | -0.500 | 0.183 | -2.740 | 0.007 | 0.015 |
| ODI-PTR | Control (Ref) vs 22q11.2Deletion | 0.766 | 0.180 | 4.247 | 0.000 | 0.000 |
| ODI-RLIC | Control (Ref) vs 22q11.2Deletion | 0.048 | 0.203 | 0.237 | 0.813 | 0.865 |
| ODI-SCC | Control (Ref) vs 22q11.2Deletion | 0.150 | 0.199 | 0.753 | 0.453 | 0.535 |
| ODI-SCR | Control (Ref) vs 22q11.2Deletion | -0.140 | 0.203 | -0.686 | 0.494 | 0.575 |
| ODI-SFO | Control (Ref) vs 22q11.2Deletion | 0.523 | 0.186 | 2.817 | 0.006 | 0.013 |
| ODI-SLF | Control (Ref) vs 22q11.2Deletion | 0.880 | 0.194 | 4.539 | 0.000 | 0.000 |
| ODI-SS | Control (Ref) vs 22q11.2Deletion | 0.535 | 0.212 | 2.518 | 0.013 | 0.026 |
| ODI-TAP | Control (Ref) vs 22q11.2Deletion | -0.128 | 0.230 | -0.558 | 0.578 | 0.664 |
| ICVF | Control (Ref) vs 22q11.2Duplication | -0.922 | 0.171 | -5.395 | 0.000 | 0.000 |
| ISO | Control (Ref) vs 22q11.2Duplication | -0.495 | 0.188 | -2.636 | 0.010 | 0.021 |
| ODI | Control (Ref) vs 22q11.2Duplication | 0.306 | 0.233 | 1.311 | 0.194 | 0.262 |
| ICVF-ACR | Control (Ref) vs 22q11.2Duplication | -1.052 | 0.220 | -4.775 | 0.000 | 0.000 |
| ICVF-ALIC | Control (Ref) vs 22q11.2Duplication | -0.618 | 0.154 | -4.006 | 0.000 | 0.000 |
| ICVF-BCC | Control (Ref) vs 22q11.2Duplication | -1.395 | 0.210 | -6.643 | 0.000 | 0.000 |
| ICVF-CC | Control (Ref) vs 22q11.2Duplication | -1.381 | 0.213 | -6.482 | 0.000 | 0.000 |
| ICVF-CGC | Control (Ref) vs 22q11.2Duplication | -0.965 | 0.179 | -5.390 | 0.000 | 0.000 |
| ICVF-CGH | Control (Ref) vs 22q11.2Duplication | -0.741 | 0.181 | -4.093 | 0.000 | 0.000 |
| ICVF-CR | Control (Ref) vs 22q11.2Duplication | -1.104 | 0.211 | -5.230 | 0.000 | 0.000 |
| ICVF-CST | Control (Ref) vs 22q11.2Duplication | -0.551 | 0.221 | -2.487 | 0.015 | 0.029 |
| ICVF-EC | Control (Ref) vs 22q11.2Duplication | -0.698 | 0.181 | -3.863 | 0.000 | 0.001 |
| ICVF-FX | Control (Ref) vs 22q11.2Duplication | -0.692 | 0.247 | -2.799 | 0.007 | 0.014 |
| ICVF-FXST | Control (Ref) vs 22q11.2Duplication | -0.668 | 0.174 | -3.827 | 0.000 | 0.001 |
| ICVF-GCC | Control (Ref) vs 22q11.2Duplication | -1.207 | 0.231 | -5.219 | 0.000 | 0.000 |
| ICVF-IC | Control (Ref) vs 22q11.2Duplication | -0.879 | 0.170 | -5.170 | 0.000 | 0.000 |
| ICVF-UNC | Control (Ref) vs 22q11.2Duplication | -0.528 | 0.168 | -3.150 | 0.002 | 0.006 |
| ICVF-PCR | Control (Ref) vs 22q11.2Duplication | -1.144 | 0.209 | -5.460 | 0.000 | 0.000 |
| ICVF-PLIC | Control (Ref) vs 22q11.2Duplication | -0.820 | 0.179 | -4.567 | 0.000 | 0.000 |
| ICVF-PTR | Control (Ref) vs 22q11.2Duplication | -1.164 | 0.212 | -5.503 | 0.000 | 0.000 |
| ICVF-RLIC | Control (Ref) vs 22q11.2Duplication | -1.038 | 0.191 | -5.427 | 0.000 | 0.000 |
| ICVF-SCC | Control (Ref) vs 22q11.2Duplication | -1.230 | 0.230 | -5.345 | 0.000 | 0.000 |
| ICVF-SCR | Control (Ref) vs 22q11.2Duplication | -1.107 | 0.208 | -5.333 | 0.000 | 0.000 |
| ICVF-SFO | Control (Ref) vs 22q11.2Duplication | -0.809 | 0.202 | -4.017 | 0.000 | 0.000 |
| ICVF-SLF | Control (Ref) vs 22q11.2Duplication | -1.125 | 0.189 | -5.963 | 0.000 | 0.000 |
| ICVF-SS | Control (Ref) vs 22q11.2Duplication | -0.933 | 0.211 | -4.415 | 0.000 | 0.000 |
| ICVF-TAP | Control (Ref) vs 22q11.2Duplication | -1.065 | 0.239 | -4.462 | 0.000 | 0.000 |
| ISO-ACR | Control (Ref) vs 22q11.2Duplication | -0.450 | 0.236 | -1.907 | 0.061 | 0.098 |
| ISO-ALIC | Control (Ref) vs 22q11.2Duplication | 0.049 | 0.275 | 0.176 | 0.860 | 0.909 |
| ISO-BCC | Control (Ref) vs 22q11.2Duplication | -0.294 | 0.264 | -1.114 | 0.269 | 0.345 |
| ISO-CC | Control (Ref) vs 22q11.2Duplication | -0.197 | 0.278 | -0.710 | 0.480 | 0.562 |
| ISO-CGC | Control (Ref) vs 22q11.2Duplication | -0.298 | 0.252 | -1.180 | 0.242 | 0.313 |
| ISO-CGH | Control (Ref) vs 22q11.2Duplication | 0.031 | 0.244 | 0.126 | 0.900 | 0.934 |
| ISO-CR | Control (Ref) vs 22q11.2Duplication | -0.601 | 0.234 | -2.567 | 0.013 | 0.024 |
| ISO-CST | Control (Ref) vs 22q11.2Duplication | 0.469 | 0.239 | 1.965 | 0.054 | 0.088 |
| ISO-EC | Control (Ref) vs 22q11.2Duplication | -0.252 | 0.191 | -1.314 | 0.193 | 0.262 |
| ISO-FX | Control (Ref) vs 22q11.2Duplication | 0.506 | 0.279 | 1.815 | 0.074 | 0.116 |
| ISO-FXST | Control (Ref) vs 22q11.2Duplication | -0.665 | 0.244 | -2.725 | 0.008 | 0.017 |
| ISO-GCC | Control (Ref) vs 22q11.2Duplication | -0.343 | 0.260 | -1.320 | 0.191 | 0.261 |
| ISO-IC | Control (Ref) vs 22q11.2Duplication | -0.209 | 0.248 | -0.844 | 0.402 | 0.488 |
| ISO-UNC | Control (Ref) vs 22q11.2Duplication | -0.395 | 0.246 | -1.606 | 0.113 | 0.168 |
| ISO-PCR | Control (Ref) vs 22q11.2Duplication | -0.670 | 0.233 | -2.879 | 0.005 | 0.012 |
| ISO-PLIC | Control (Ref) vs 22q11.2Duplication | -0.189 | 0.254 | -0.744 | 0.459 | 0.541 |
| ISO-PTR | Control (Ref) vs 22q11.2Duplication | -0.765 | 0.240 | -3.188 | 0.002 | 0.005 |
| ISO-RLIC | Control (Ref) vs 22q11.2Duplication | -0.370 | 0.234 | -1.584 | 0.118 | 0.173 |
| ISO-SCC | Control (Ref) vs 22q11.2Duplication | 0.090 | 0.305 | 0.297 | 0.768 | 0.831 |
| ISO-SCR | Control (Ref) vs 22q11.2Duplication | -0.661 | 0.232 | -2.854 | 0.006 | 0.013 |
| ISO-SFO | Control (Ref) vs 22q11.2Duplication | -0.605 | 0.265 | -2.284 | 0.026 | 0.048 |
| ISO-SLF | Control (Ref) vs 22q11.2Duplication | -0.691 | 0.216 | -3.202 | 0.002 | 0.005 |
| ISO-SS | Control (Ref) vs 22q11.2Duplication | -0.471 | 0.218 | -2.166 | 0.034 | 0.060 |
| ISO-TAP | Control (Ref) vs 22q11.2Duplication | -0.540 | 0.263 | -2.052 | 0.044 | 0.075 |
| ODI-ACR | Control (Ref) vs 22q11.2Duplication | 0.025 | 0.273 | 0.093 | 0.926 | 0.948 |
| ODI-ALIC | Control (Ref) vs 22q11.2Duplication | 0.656 | 0.242 | 2.708 | 0.009 | 0.017 |
| ODI-BCC | Control (Ref) vs 22q11.2Duplication | 0.250 | 0.266 | 0.940 | 0.351 | 0.437 |
| ODI-CC | Control (Ref) vs 22q11.2Duplication | -0.034 | 0.268 | -0.129 | 0.898 | 0.934 |
| ODI-CGC | Control (Ref) vs 22q11.2Duplication | 0.411 | 0.270 | 1.523 | 0.132 | 0.190 |
| ODI-CGH | Control (Ref) vs 22q11.2Duplication | -0.325 | 0.267 | -1.217 | 0.228 | 0.304 |
| ODI-CR | Control (Ref) vs 22q11.2Duplication | -0.307 | 0.258 | -1.188 | 0.239 | 0.313 |
| ODI-CST | Control (Ref) vs 22q11.2Duplication | 0.329 | 0.272 | 1.209 | 0.231 | 0.307 |
| ODI-EC | Control (Ref) vs 22q11.2Duplication | 0.537 | 0.261 | 2.054 | 0.044 | 0.075 |
| ODI-FX | Control (Ref) vs 22q11.2Duplication | -0.044 | 0.255 | -0.173 | 0.863 | 0.909 |
| ODI-FXST | Control (Ref) vs 22q11.2Duplication | -0.019 | 0.254 | -0.076 | 0.940 | 0.952 |
| ODI-GCC | Control (Ref) vs 22q11.2Duplication | -0.117 | 0.271 | -0.433 | 0.666 | 0.746 |
| ODI-IC | Control (Ref) vs 22q11.2Duplication | 0.361 | 0.236 | 1.525 | 0.132 | 0.190 |
| ODI-UNC | Control (Ref) vs 22q11.2Duplication | 0.748 | 0.286 | 2.618 | 0.011 | 0.021 |
| ODI-PCR | Control (Ref) vs 22q11.2Duplication | -0.545 | 0.262 | -2.079 | 0.041 | 0.071 |
| ODI-PLIC | Control (Ref) vs 22q11.2Duplication | 0.409 | 0.235 | 1.738 | 0.087 | 0.131 |
| ODI-PTR | Control (Ref) vs 22q11.2Duplication | 0.031 | 0.267 | 0.115 | 0.909 | 0.937 |
| ODI-RLIC | Control (Ref) vs 22q11.2Duplication | -0.331 | 0.255 | -1.297 | 0.199 | 0.268 |
| ODI-SCC | Control (Ref) vs 22q11.2Duplication | -0.222 | 0.273 | -0.813 | 0.419 | 0.507 |
| ODI-SCR | Control (Ref) vs 22q11.2Duplication | -0.523 | 0.250 | -2.091 | 0.040 | 0.070 |
| ODI-SFO | Control (Ref) vs 22q11.2Duplication | 0.040 | 0.269 | 0.150 | 0.881 | 0.924 |
| ODI-SLF | Control (Ref) vs 22q11.2Duplication | -0.013 | 0.285 | -0.045 | 0.964 | 0.974 |
| ODI-SS | Control (Ref) vs 22q11.2Duplication | -0.501 | 0.283 | -1.768 | 0.082 | 0.124 |
| ODI-TAP | Control (Ref) vs 22q11.2Duplication | 0.279 | 0.293 | 0.952 | 0.345 | 0.432 |
| ICVF | 22q11.2Deletion (Ref) vs 22q11.2Duplication | -1.428 | 0.142 | -10.070 | 0.000 | 0.000 |
| ISO | 22q11.2Deletion (Ref) vs 22q11.2Duplication | -0.790 | 0.161 | -4.913 | 0.000 | 0.000 |
| ODI | 22q11.2Deletion (Ref) vs 22q11.2Duplication | 0.319 | 0.209 | 1.526 | 0.130 | 0.189 |
| ICVF-ACR | 22q11.2Deletion (Ref) vs 22q11.2Duplication | -1.579 | 0.158 | -9.990 | 0.000 | 0.000 |
| ICVF-ALIC | 22q11.2Deletion (Ref) vs 22q11.2Duplication | -1.254 | 0.122 | -10.301 | 0.000 | 0.000 |
| ICVF-BCC | 22q11.2Deletion (Ref) vs 22q11.2Duplication | -1.652 | 0.150 | -11.014 | 0.000 | 0.000 |
| ICVF-CC | 22q11.2Deletion (Ref) vs 22q11.2Duplication | -1.631 | 0.153 | -10.644 | 0.000 | 0.000 |
| ICVF-CGC | 22q11.2Deletion (Ref) vs 22q11.2Duplication | -1.452 | 0.150 | -9.657 | 0.000 | 0.000 |
| ICVF-CGH | 22q11.2Deletion (Ref) vs 22q11.2Duplication | -1.055 | 0.174 | -6.053 | 0.000 | 0.000 |
| ICVF-CR | 22q11.2Deletion (Ref) vs 22q11.2Duplication | -1.565 | 0.162 | -9.690 | 0.000 | 0.000 |
| ICVF-CST | 22q11.2Deletion (Ref) vs 22q11.2Duplication | -0.909 | 0.190 | -4.781 | 0.000 | 0.000 |
| ICVF-EC | 22q11.2Deletion (Ref) vs 22q11.2Duplication | -1.290 | 0.132 | -9.745 | 0.000 | 0.000 |
| ICVF-FX | 22q11.2Deletion (Ref) vs 22q11.2Duplication | -0.890 | 0.217 | -4.098 | 0.000 | 0.000 |
| ICVF-FXST | 22q11.2Deletion (Ref) vs 22q11.2Duplication | -1.322 | 0.149 | -8.863 | 0.000 | 0.000 |
| ICVF-GCC | 22q11.2Deletion (Ref) vs 22q11.2Duplication | -1.596 | 0.155 | -10.300 | 0.000 | 0.000 |
| ICVF-IC | 22q11.2Deletion (Ref) vs 22q11.2Duplication | -1.437 | 0.140 | -10.296 | 0.000 | 0.000 |
| ICVF-UNC | 22q11.2Deletion (Ref) vs 22q11.2Duplication | -1.315 | 0.145 | -9.056 | 0.000 | 0.000 |
| ICVF-PCR | 22q11.2Deletion (Ref) vs 22q11.2Duplication | -1.529 | 0.168 | -9.127 | 0.000 | 0.000 |
| ICVF-PLIC | 22q11.2Deletion (Ref) vs 22q11.2Duplication | -1.231 | 0.166 | -7.400 | 0.000 | 0.000 |
| ICVF-PTR | 22q11.2Deletion (Ref) vs 22q11.2Duplication | -1.595 | 0.166 | -9.602 | 0.000 | 0.000 |
| ICVF-RLIC | 22q11.2Deletion (Ref) vs 22q11.2Duplication | -1.561 | 0.145 | -10.728 | 0.000 | 0.000 |
| ICVF-SCC | 22q11.2Deletion (Ref) vs 22q11.2Duplication | -1.516 | 0.176 | -8.603 | 0.000 | 0.000 |
| ICVF-SCR | 22q11.2Deletion (Ref) vs 22q11.2Duplication | -1.557 | 0.154 | -10.109 | 0.000 | 0.000 |
| ICVF-SFO | 22q11.2Deletion (Ref) vs 22q11.2Duplication | -1.389 | 0.165 | -8.431 | 0.000 | 0.000 |
| ICVF-SLF | 22q11.2Deletion (Ref) vs 22q11.2Duplication | -1.538 | 0.152 | -10.142 | 0.000 | 0.000 |
| ICVF-SS | 22q11.2Deletion (Ref) vs 22q11.2Duplication | -1.599 | 0.150 | -10.629 | 0.000 | 0.000 |
| ICVF-TAP | 22q11.2Deletion (Ref) vs 22q11.2Duplication | -1.479 | 0.190 | -7.797 | 0.000 | 0.000 |
| ISO-ACR | 22q11.2Deletion (Ref) vs 22q11.2Duplication | -0.872 | 0.193 | -4.513 | 0.000 | 0.000 |
| ISO-ALIC | 22q11.2Deletion (Ref) vs 22q11.2Duplication | -0.321 | 0.241 | -1.331 | 0.187 | 0.258 |
| ISO-BCC | 22q11.2Deletion (Ref) vs 22q11.2Duplication | -0.301 | 0.229 | -1.315 | 0.192 | 0.261 |
| ISO-CC | 22q11.2Deletion (Ref) vs 22q11.2Duplication | -0.091 | 0.245 | -0.370 | 0.712 | 0.783 |
| ISO-CGC | 22q11.2Deletion (Ref) vs 22q11.2Duplication | -0.453 | 0.206 | -2.195 | 0.031 | 0.056 |
| ISO-CGH | 22q11.2Deletion (Ref) vs 22q11.2Duplication | -0.443 | 0.196 | -2.256 | 0.026 | 0.049 |
| ISO-CR | 22q11.2Deletion (Ref) vs 22q11.2Duplication | -0.926 | 0.191 | -4.855 | 0.000 | 0.000 |
| ISO-CST | 22q11.2Deletion (Ref) vs 22q11.2Duplication | 0.353 | 0.219 | 1.611 | 0.111 | 0.165 |
| ISO-EC | 22q11.2Deletion (Ref) vs 22q11.2Duplication | -0.162 | 0.192 | -0.843 | 0.402 | 0.488 |
| ISO-FX | 22q11.2Deletion (Ref) vs 22q11.2Duplication | 0.007 | 0.268 | 0.025 | 0.980 | 0.982 |
| ISO-FXST | 22q11.2Deletion (Ref) vs 22q11.2Duplication | -0.272 | 0.231 | -1.182 | 0.240 | 0.313 |
| ISO-GCC | 22q11.2Deletion (Ref) vs 22q11.2Duplication | -0.273 | 0.233 | -1.174 | 0.243 | 0.313 |
| ISO-IC | 22q11.2Deletion (Ref) vs 22q11.2Duplication | -0.404 | 0.202 | -1.996 | 0.049 | 0.081 |
| ISO-UNC | 22q11.2Deletion (Ref) vs 22q11.2Duplication | -0.394 | 0.215 | -1.828 | 0.071 | 0.112 |
| ISO-PCR | 22q11.2Deletion (Ref) vs 22q11.2Duplication | -0.945 | 0.193 | -4.899 | 0.000 | 0.000 |
| ISO-PLIC | 22q11.2Deletion (Ref) vs 22q11.2Duplication | -0.179 | 0.228 | -0.785 | 0.435 | 0.517 |
| ISO-PTR | 22q11.2Deletion (Ref) vs 22q11.2Duplication | -0.993 | 0.199 | -4.987 | 0.000 | 0.000 |
| ISO-RLIC | 22q11.2Deletion (Ref) vs 22q11.2Duplication | -0.568 | 0.183 | -3.099 | 0.003 | 0.006 |
| ISO-SCC | 22q11.2Deletion (Ref) vs 22q11.2Duplication | 0.271 | 0.261 | 1.038 | 0.302 | 0.382 |
| ISO-SCR | 22q11.2Deletion (Ref) vs 22q11.2Duplication | -0.855 | 0.200 | -4.279 | 0.000 | 0.000 |
| ISO-SFO | 22q11.2Deletion (Ref) vs 22q11.2Duplication | -0.928 | 0.206 | -4.496 | 0.000 | 0.000 |
| ISO-SLF | 22q11.2Deletion (Ref) vs 22q11.2Duplication | -0.876 | 0.183 | -4.777 | 0.000 | 0.000 |
| ISO-SS | 22q11.2Deletion (Ref) vs 22q11.2Duplication | -0.856 | 0.173 | -4.949 | 0.000 | 0.000 |
| ISO-TAP | 22q11.2Deletion (Ref) vs 22q11.2Duplication | -0.760 | 0.222 | -3.429 | 0.001 | 0.002 |
| ODI-ACR | 22q11.2Deletion (Ref) vs 22q11.2Duplication | -0.064 | 0.243 | -0.265 | 0.791 | 0.847 |
| ODI-ALIC | 22q11.2Deletion (Ref) vs 22q11.2Duplication | 1.038 | 0.200 | 5.192 | 0.000 | 0.000 |
| ODI-BCC | 22q11.2Deletion (Ref) vs 22q11.2Duplication | 0.353 | 0.241 | 1.460 | 0.148 | 0.208 |
| ODI-CC | 22q11.2Deletion (Ref) vs 22q11.2Duplication | 0.064 | 0.246 | 0.262 | 0.794 | 0.847 |
| ODI-CGC | 22q11.2Deletion (Ref) vs 22q11.2Duplication | 0.642 | 0.245 | 2.624 | 0.010 | 0.020 |
| ODI-CGH | 22q11.2Deletion (Ref) vs 22q11.2Duplication | -0.640 | 0.240 | -2.671 | 0.009 | 0.018 |
| ODI-CR | 22q11.2Deletion (Ref) vs 22q11.2Duplication | -0.193 | 0.240 | -0.802 | 0.424 | 0.509 |
| ODI-CST | 22q11.2Deletion (Ref) vs 22q11.2Duplication | 0.856 | 0.222 | 3.854 | 0.000 | 0.001 |
| ODI-EC | 22q11.2Deletion (Ref) vs 22q11.2Duplication | 0.019 | 0.230 | 0.081 | 0.936 | 0.951 |
| ODI-FX | 22q11.2Deletion (Ref) vs 22q11.2Duplication | 0.525 | 0.254 | 2.067 | 0.042 | 0.071 |
| ODI-FXST | 22q11.2Deletion (Ref) vs 22q11.2Duplication | -0.476 | 0.228 | -2.092 | 0.039 | 0.069 |
| ODI-GCC | 22q11.2Deletion (Ref) vs 22q11.2Duplication | -0.136 | 0.259 | -0.524 | 0.601 | 0.678 |
| ODI-IC | 22q11.2Deletion (Ref) vs 22q11.2Duplication | 0.690 | 0.213 | 3.237 | 0.002 | 0.004 |
| ODI-UNC | 22q11.2Deletion (Ref) vs 22q11.2Duplication | 0.716 | 0.257 | 2.792 | 0.006 | 0.014 |
| ODI-PCR | 22q11.2Deletion (Ref) vs 22q11.2Duplication | -0.654 | 0.240 | -2.727 | 0.008 | 0.016 |
| ODI-PLIC | 22q11.2Deletion (Ref) vs 22q11.2Duplication | 0.943 | 0.222 | 4.244 | 0.000 | 0.000 |
| ODI-PTR | 22q11.2Deletion (Ref) vs 22q11.2Duplication | -0.855 | 0.219 | -3.908 | 0.000 | 0.000 |
| ODI-RLIC | 22q11.2Deletion (Ref) vs 22q11.2Duplication | -0.407 | 0.229 | -1.779 | 0.079 | 0.122 |
| ODI-SCC | 22q11.2Deletion (Ref) vs 22q11.2Duplication | -0.135 | 0.254 | -0.534 | 0.595 | 0.676 |
| ODI-SCR | 22q11.2Deletion (Ref) vs 22q11.2Duplication | -0.029 | 0.259 | -0.111 | 0.912 | 0.937 |
| ODI-SFO | 22q11.2Deletion (Ref) vs 22q11.2Duplication | -0.366 | 0.248 | -1.475 | 0.144 | 0.204 |
| ODI-SLF | 22q11.2Deletion (Ref) vs 22q11.2Duplication | -0.739 | 0.234 | -3.158 | 0.002 | 0.005 |
| ODI-SS | 22q11.2Deletion (Ref) vs 22q11.2Duplication | -0.928 | 0.235 | -3.951 | 0.000 | 0.000 |
| ODI-TAP | 22q11.2Deletion (Ref) vs 22q11.2Duplication | 0.237 | 0.273 | 0.868 | 0.387 | 0.476 |
| ICVF | GeneDosage (1 = del, 2 = con, 3 dup) | -0.714 | 0.071 | -10.070 | 0.000 | 0.000 |
| ISO | GeneDosage (1 = del, 2 = con, 3 dup) | -0.395 | 0.080 | -4.913 | 0.000 | 0.000 |
| ODI | GeneDosage (1 = del, 2 = con, 3 dup) | 0.160 | 0.105 | 1.526 | 0.130 | 0.189 |
| ICVF-ACR | GeneDosage (1 = del, 2 = con, 3 dup) | -0.790 | 0.079 | -9.990 | 0.000 | 0.000 |
| ICVF-ALIC | GeneDosage (1 = del, 2 = con, 3 dup) | -0.627 | 0.061 | -10.301 | 0.000 | 0.000 |
| ICVF-BCC | GeneDosage (1 = del, 2 = con, 3 dup) | -0.826 | 0.075 | -11.014 | 0.000 | 0.000 |
| ICVF-CC | GeneDosage (1 = del, 2 = con, 3 dup) | -0.816 | 0.077 | -10.644 | 0.000 | 0.000 |
| ICVF-CGC | GeneDosage (1 = del, 2 = con, 3 dup) | -0.726 | 0.075 | -9.657 | 0.000 | 0.000 |
| ICVF-CGH | GeneDosage (1 = del, 2 = con, 3 dup) | -0.527 | 0.087 | -6.053 | 0.000 | 0.000 |
| ICVF-CR | GeneDosage (1 = del, 2 = con, 3 dup) | -0.783 | 0.081 | -9.690 | 0.000 | 0.000 |
| ICVF-CST | GeneDosage (1 = del, 2 = con, 3 dup) | -0.454 | 0.095 | -4.781 | 0.000 | 0.000 |
| ICVF-EC | GeneDosage (1 = del, 2 = con, 3 dup) | -0.645 | 0.066 | -9.745 | 0.000 | 0.000 |
| ICVF-FX | GeneDosage (1 = del, 2 = con, 3 dup) | -0.445 | 0.109 | -4.098 | 0.000 | 0.000 |
| ICVF-FXST | GeneDosage (1 = del, 2 = con, 3 dup) | -0.661 | 0.075 | -8.863 | 0.000 | 0.000 |
| ICVF-GCC | GeneDosage (1 = del, 2 = con, 3 dup) | -0.798 | 0.077 | -10.300 | 0.000 | 0.000 |
| ICVF-IC | GeneDosage (1 = del, 2 = con, 3 dup) | -0.718 | 0.070 | -10.296 | 0.000 | 0.000 |
| ICVF-UNC | GeneDosage (1 = del, 2 = con, 3 dup) | -0.658 | 0.073 | -9.056 | 0.000 | 0.000 |
| ICVF-PCR | GeneDosage (1 = del, 2 = con, 3 dup) | -0.765 | 0.084 | -9.127 | 0.000 | 0.000 |
| ICVF-PLIC | GeneDosage (1 = del, 2 = con, 3 dup) | -0.616 | 0.083 | -7.400 | 0.000 | 0.000 |
| ICVF-PTR | GeneDosage (1 = del, 2 = con, 3 dup) | -0.798 | 0.083 | -9.602 | 0.000 | 0.000 |
| ICVF-RLIC | GeneDosage (1 = del, 2 = con, 3 dup) | -0.780 | 0.073 | -10.728 | 0.000 | 0.000 |
| ICVF-SCC | GeneDosage (1 = del, 2 = con, 3 dup) | -0.758 | 0.088 | -8.603 | 0.000 | 0.000 |
| ICVF-SCR | GeneDosage (1 = del, 2 = con, 3 dup) | -0.779 | 0.077 | -10.109 | 0.000 | 0.000 |
| ICVF-SFO | GeneDosage (1 = del, 2 = con, 3 dup) | -0.695 | 0.082 | -8.431 | 0.000 | 0.000 |
| ICVF-SLF | GeneDosage (1 = del, 2 = con, 3 dup) | -0.769 | 0.076 | -10.142 | 0.000 | 0.000 |
| ICVF-SS | GeneDosage (1 = del, 2 = con, 3 dup) | -0.799 | 0.075 | -10.629 | 0.000 | 0.000 |
| ICVF-TAP | GeneDosage (1 = del, 2 = con, 3 dup) | -0.739 | 0.095 | -7.797 | 0.000 | 0.000 |
| ISO-ACR | GeneDosage (1 = del, 2 = con, 3 dup) | -0.436 | 0.097 | -4.513 | 0.000 | 0.000 |
| ISO-ALIC | GeneDosage (1 = del, 2 = con, 3 dup) | -0.160 | 0.120 | -1.331 | 0.187 | 0.258 |
| ISO-BCC | GeneDosage (1 = del, 2 = con, 3 dup) | -0.150 | 0.114 | -1.315 | 0.192 | 0.261 |
| ISO-CC | GeneDosage (1 = del, 2 = con, 3 dup) | -0.045 | 0.123 | -0.370 | 0.712 | 0.783 |
| ISO-CGC | GeneDosage (1 = del, 2 = con, 3 dup) | -0.226 | 0.103 | -2.195 | 0.031 | 0.056 |
| ISO-CGH | GeneDosage (1 = del, 2 = con, 3 dup) | -0.221 | 0.098 | -2.256 | 0.026 | 0.049 |
| ISO-CR | GeneDosage (1 = del, 2 = con, 3 dup) | -0.463 | 0.095 | -4.855 | 0.000 | 0.000 |
| ISO-CST | GeneDosage (1 = del, 2 = con, 3 dup) | 0.177 | 0.110 | 1.611 | 0.111 | 0.165 |
| ISO-EC | GeneDosage (1 = del, 2 = con, 3 dup) | -0.081 | 0.096 | -0.843 | 0.402 | 0.488 |
| ISO-FX | GeneDosage (1 = del, 2 = con, 3 dup) | 0.003 | 0.134 | 0.025 | 0.980 | 0.982 |
| ISO-FXST | GeneDosage (1 = del, 2 = con, 3 dup) | -0.136 | 0.115 | -1.182 | 0.240 | 0.313 |
| ISO-GCC | GeneDosage (1 = del, 2 = con, 3 dup) | -0.137 | 0.116 | -1.174 | 0.243 | 0.313 |
| ISO-IC | GeneDosage (1 = del, 2 = con, 3 dup) | -0.202 | 0.101 | -1.996 | 0.049 | 0.081 |
| ISO-UNC | GeneDosage (1 = del, 2 = con, 3 dup) | -0.197 | 0.108 | -1.828 | 0.071 | 0.112 |
| ISO-PCR | GeneDosage (1 = del, 2 = con, 3 dup) | -0.473 | 0.096 | -4.899 | 0.000 | 0.000 |
| ISO-PLIC | GeneDosage (1 = del, 2 = con, 3 dup) | -0.089 | 0.114 | -0.785 | 0.435 | 0.517 |
| ISO-PTR | GeneDosage (1 = del, 2 = con, 3 dup) | -0.497 | 0.100 | -4.987 | 0.000 | 0.000 |
| ISO-RLIC | GeneDosage (1 = del, 2 = con, 3 dup) | -0.284 | 0.092 | -3.099 | 0.003 | 0.006 |
| ISO-SCC | GeneDosage (1 = del, 2 = con, 3 dup) | 0.135 | 0.130 | 1.038 | 0.302 | 0.382 |
| ISO-SCR | GeneDosage (1 = del, 2 = con, 3 dup) | -0.427 | 0.100 | -4.279 | 0.000 | 0.000 |
| ISO-SFO | GeneDosage (1 = del, 2 = con, 3 dup) | -0.464 | 0.103 | -4.496 | 0.000 | 0.000 |
| ISO-SLF | GeneDosage (1 = del, 2 = con, 3 dup) | -0.438 | 0.092 | -4.777 | 0.000 | 0.000 |
| ISO-SS | GeneDosage (1 = del, 2 = con, 3 dup) | -0.428 | 0.086 | -4.949 | 0.000 | 0.000 |
| ISO-TAP | GeneDosage (1 = del, 2 = con, 3 dup) | -0.380 | 0.111 | -3.429 | 0.001 | 0.002 |
| ODI-ACR | GeneDosage (1 = del, 2 = con, 3 dup) | -0.032 | 0.121 | -0.265 | 0.791 | 0.847 |
| ODI-ALIC | GeneDosage (1 = del, 2 = con, 3 dup) | 0.519 | 0.100 | 5.192 | 0.000 | 0.000 |
| ODI-BCC | GeneDosage (1 = del, 2 = con, 3 dup) | 0.176 | 0.121 | 1.460 | 0.148 | 0.208 |
| ODI-CC | GeneDosage (1 = del, 2 = con, 3 dup) | 0.032 | 0.123 | 0.262 | 0.794 | 0.847 |
| ODI-CGC | GeneDosage (1 = del, 2 = con, 3 dup) | 0.321 | 0.122 | 2.624 | 0.010 | 0.020 |
| ODI-CGH | GeneDosage (1 = del, 2 = con, 3 dup) | -0.320 | 0.120 | -2.671 | 0.009 | 0.018 |
| ODI-CR | GeneDosage (1 = del, 2 = con, 3 dup) | -0.096 | 0.120 | -0.802 | 0.424 | 0.509 |
| ODI-CST | GeneDosage (1 = del, 2 = con, 3 dup) | 0.428 | 0.111 | 3.854 | 0.000 | 0.001 |
| ODI-EC | GeneDosage (1 = del, 2 = con, 3 dup) | 0.009 | 0.115 | 0.081 | 0.936 | 0.951 |
| ODI-FX | GeneDosage (1 = del, 2 = con, 3 dup) | 0.263 | 0.127 | 2.067 | 0.042 | 0.071 |
| ODI-FXST | GeneDosage (1 = del, 2 = con, 3 dup) | -0.238 | 0.114 | -2.092 | 0.039 | 0.069 |
| ODI-GCC | GeneDosage (1 = del, 2 = con, 3 dup) | -0.068 | 0.130 | -0.524 | 0.601 | 0.678 |
| ODI-IC | GeneDosage (1 = del, 2 = con, 3 dup) | 0.345 | 0.107 | 3.237 | 0.002 | 0.004 |
| ODI-UNC | GeneDosage (1 = del, 2 = con, 3 dup) | 0.358 | 0.128 | 2.792 | 0.006 | 0.014 |
| ODI-PCR | GeneDosage (1 = del, 2 = con, 3 dup) | -0.327 | 0.120 | -2.727 | 0.008 | 0.016 |
| ODI-PLIC | GeneDosage (1 = del, 2 = con, 3 dup) | 0.471 | 0.111 | 4.244 | 0.000 | 0.000 |
| ODI-PTR | GeneDosage (1 = del, 2 = con, 3 dup) | -0.428 | 0.109 | -3.908 | 0.000 | 0.000 |
| ODI-RLIC | GeneDosage (1 = del, 2 = con, 3 dup) | -0.203 | 0.114 | -1.779 | 0.079 | 0.122 |
| ODI-SCC | GeneDosage (1 = del, 2 = con, 3 dup) | -0.068 | 0.127 | -0.534 | 0.595 | 0.676 |
| ODI-SCR | GeneDosage (1 = del, 2 = con, 3 dup) | -0.014 | 0.129 | -0.111 | 0.912 | 0.937 |
| ODI-SFO | GeneDosage (1 = del, 2 = con, 3 dup) | -0.183 | 0.124 | -1.475 | 0.144 | 0.204 |
| ODI-SLF | GeneDosage (1 = del, 2 = con, 3 dup) | -0.370 | 0.117 | -3.158 | 0.002 | 0.005 |
| ODI-SS | GeneDosage (1 = del, 2 = con, 3 dup) | -0.464 | 0.117 | -3.951 | 0.000 | 0.000 |
| ODI-TAP | GeneDosage (1 = del, 2 = con, 3 dup) | 0.119 | 0.137 | 0.868 | 0.387 | 0.476 |

| sTable 7. Sensitivity analysis - Group differences across individuals scanned at the same scanner site, adjusted for estimated intracranial volume | | | | | | |
| --- | --- | --- | --- | --- | --- | --- |
| Dependent variable | **Analysis** | **Std.B.** | **SE** | **T-value** | **P-value** | **pFDR** |
| ICVF | Control (Ref) vs 22q11.2Deletion | 0.769 | 0.141 | 5.454 | 0.000 | 0.000 |
| ISO | Control (Ref) vs 22q11.2Deletion | 0.221 | 0.130 | 1.702 | 0.092 | 0.142 |
| ODI | Control (Ref) vs 22q11.2Deletion | 0.178 | 0.168 | 1.056 | 0.293 | 0.378 |
| ICVF-ACR | Control (Ref) vs 22q11.2Deletion | 1.006 | 0.162 | 6.209 | 0.000 | 0.000 |
| ICVF-ALIC | Control (Ref) vs 22q11.2Deletion | 0.627 | 0.117 | 5.350 | 0.000 | 0.000 |
| ICVF-BCC | Control (Ref) vs 22q11.2Deletion | 1.118 | 0.175 | 6.378 | 0.000 | 0.000 |
| ICVF-CC | Control (Ref) vs 22q11.2Deletion | 1.099 | 0.178 | 6.185 | 0.000 | 0.000 |
| ICVF-CGC | Control (Ref) vs 22q11.2Deletion | 0.710 | 0.147 | 4.829 | 0.000 | 0.000 |
| ICVF-CGH | Control (Ref) vs 22q11.2Deletion | 0.344 | 0.158 | 2.172 | 0.032 | 0.056 |
| ICVF-CR | Control (Ref) vs 22q11.2Deletion | 0.978 | 0.170 | 5.758 | 0.000 | 0.000 |
| ICVF-CST | Control (Ref) vs 22q11.2Deletion | 0.423 | 0.184 | 2.299 | 0.023 | 0.043 |
| ICVF-EC | Control (Ref) vs 22q11.2Deletion | 0.584 | 0.134 | 4.343 | 0.000 | 0.000 |
| ICVF-FX | Control (Ref) vs 22q11.2Deletion | 0.317 | 0.194 | 1.632 | 0.105 | 0.158 |
| ICVF-FXST | Control (Ref) vs 22q11.2Deletion | 0.776 | 0.155 | 4.996 | 0.000 | 0.000 |
| ICVF-GCC | Control (Ref) vs 22q11.2Deletion | 1.131 | 0.170 | 6.651 | 0.000 | 0.000 |
| ICVF-IC | Control (Ref) vs 22q11.2Deletion | 0.848 | 0.139 | 6.117 | 0.000 | 0.000 |
| ICVF-UNC | Control (Ref) vs 22q11.2Deletion | 0.812 | 0.140 | 5.795 | 0.000 | 0.000 |
| ICVF-PCR | Control (Ref) vs 22q11.2Deletion | 1.034 | 0.179 | 5.770 | 0.000 | 0.000 |
| ICVF-PLIC | Control (Ref) vs 22q11.2Deletion | 0.729 | 0.157 | 4.652 | 0.000 | 0.000 |
| ICVF-PTR | Control (Ref) vs 22q11.2Deletion | 1.028 | 0.186 | 5.536 | 0.000 | 0.000 |
| ICVF-RLIC | Control (Ref) vs 22q11.2Deletion | 0.928 | 0.177 | 5.253 | 0.000 | 0.000 |
| ICVF-SCC | Control (Ref) vs 22q11.2Deletion | 0.917 | 0.200 | 4.586 | 0.000 | 0.000 |
| ICVF-SCR | Control (Ref) vs 22q11.2Deletion | 0.868 | 0.177 | 4.914 | 0.000 | 0.000 |
| ICVF-SFO | Control (Ref) vs 22q11.2Deletion | 0.604 | 0.156 | 3.878 | 0.000 | 0.000 |
| ICVF-SLF | Control (Ref) vs 22q11.2Deletion | 0.938 | 0.165 | 5.687 | 0.000 | 0.000 |
| ICVF-SS | Control (Ref) vs 22q11.2Deletion | 1.035 | 0.172 | 6.033 | 0.000 | 0.000 |
| ICVF-TAP | Control (Ref) vs 22q11.2Deletion | 0.986 | 0.201 | 4.910 | 0.000 | 0.000 |
| ISO-ACR | Control (Ref) vs 22q11.2Deletion | 0.366 | 0.174 | 2.103 | 0.038 | 0.065 |
| ISO-ALIC | Control (Ref) vs 22q11.2Deletion | 0.039 | 0.196 | 0.200 | 0.842 | 0.902 |
| ISO-BCC | Control (Ref) vs 22q11.2Deletion | -0.066 | 0.198 | -0.334 | 0.739 | 0.809 |
| ISO-CC | Control (Ref) vs 22q11.2Deletion | -0.097 | 0.193 | -0.501 | 0.617 | 0.715 |
| ISO-CGC | Control (Ref) vs 22q11.2Deletion | 0.058 | 0.172 | 0.335 | 0.739 | 0.809 |
| ISO-CGH | Control (Ref) vs 22q11.2Deletion | 0.317 | 0.185 | 1.716 | 0.089 | 0.139 |
| ISO-CR | Control (Ref) vs 22q11.2Deletion | 0.331 | 0.165 | 2.005 | 0.047 | 0.078 |
| ISO-CST | Control (Ref) vs 22q11.2Deletion | 0.027 | 0.183 | 0.148 | 0.882 | 0.932 |
| ISO-EC | Control (Ref) vs 22q11.2Deletion | 0.005 | 0.168 | 0.030 | 0.976 | 0.989 |
| ISO-FX | Control (Ref) vs 22q11.2Deletion | 0.596 | 0.203 | 2.937 | 0.004 | 0.009 |
| ISO-FXST | Control (Ref) vs 22q11.2Deletion | -0.268 | 0.204 | -1.318 | 0.190 | 0.261 |
| ISO-GCC | Control (Ref) vs 22q11.2Deletion | 0.031 | 0.203 | 0.152 | 0.880 | 0.932 |
| ISO-IC | Control (Ref) vs 22q11.2Deletion | 0.004 | 0.161 | 0.024 | 0.981 | 0.989 |
| ISO-UNC | Control (Ref) vs 22q11.2Deletion | 0.046 | 0.186 | 0.247 | 0.805 | 0.878 |
| ISO-PCR | Control (Ref) vs 22q11.2Deletion | 0.348 | 0.161 | 2.166 | 0.032 | 0.056 |
| ISO-PLIC | Control (Ref) vs 22q11.2Deletion | -0.174 | 0.180 | -0.968 | 0.335 | 0.420 |
| ISO-PTR | Control (Ref) vs 22q11.2Deletion | 0.423 | 0.169 | 2.509 | 0.014 | 0.026 |
| ISO-RLIC | Control (Ref) vs 22q11.2Deletion | 0.124 | 0.161 | 0.770 | 0.443 | 0.529 |
| ISO-SCC | Control (Ref) vs 22q11.2Deletion | -0.186 | 0.173 | -1.078 | 0.283 | 0.368 |
| ISO-SCR | Control (Ref) vs 22q11.2Deletion | 0.172 | 0.173 | 0.995 | 0.322 | 0.412 |
| ISO-SFO | Control (Ref) vs 22q11.2Deletion | 0.135 | 0.201 | 0.671 | 0.504 | 0.600 |
| ISO-SLF | Control (Ref) vs 22q11.2Deletion | 0.256 | 0.140 | 1.828 | 0.070 | 0.114 |
| ISO-SS | Control (Ref) vs 22q11.2Deletion | 0.174 | 0.178 | 0.975 | 0.332 | 0.420 |
| ISO-TAP | Control (Ref) vs 22q11.2Deletion | 0.394 | 0.196 | 2.011 | 0.047 | 0.077 |
| ODI-ACR | Control (Ref) vs 22q11.2Deletion | 0.210 | 0.194 | 1.080 | 0.282 | 0.368 |
| ODI-ALIC | Control (Ref) vs 22q11.2Deletion | -0.324 | 0.192 | -1.688 | 0.094 | 0.145 |
| ODI-BCC | Control (Ref) vs 22q11.2Deletion | 0.263 | 0.186 | 1.414 | 0.160 | 0.226 |
| ODI-CC | Control (Ref) vs 22q11.2Deletion | 0.252 | 0.182 | 1.383 | 0.169 | 0.237 |
| ODI-CGC | Control (Ref) vs 22q11.2Deletion | -0.076 | 0.216 | -0.351 | 0.726 | 0.809 |
| ODI-CGH | Control (Ref) vs 22q11.2Deletion | 0.443 | 0.209 | 2.118 | 0.036 | 0.063 |
| ODI-CR | Control (Ref) vs 22q11.2Deletion | 0.082 | 0.189 | 0.433 | 0.666 | 0.768 |
| ODI-CST | Control (Ref) vs 22q11.2Deletion | -0.319 | 0.213 | -1.493 | 0.138 | 0.200 |
| ODI-EC | Control (Ref) vs 22q11.2Deletion | 0.520 | 0.188 | 2.764 | 0.007 | 0.014 |
| ODI-FX | Control (Ref) vs 22q11.2Deletion | -0.038 | 0.221 | -0.171 | 0.865 | 0.920 |
| ODI-FXST | Control (Ref) vs 22q11.2Deletion | 0.518 | 0.191 | 2.704 | 0.008 | 0.016 |
| ODI-GCC | Control (Ref) vs 22q11.2Deletion | 0.273 | 0.204 | 1.338 | 0.184 | 0.253 |
| ODI-IC | Control (Ref) vs 22q11.2Deletion | -0.366 | 0.181 | -2.025 | 0.045 | 0.075 |
| ODI-UNC | Control (Ref) vs 22q11.2Deletion | -0.046 | 0.213 | -0.214 | 0.831 | 0.896 |
| ODI-PCR | Control (Ref) vs 22q11.2Deletion | 0.253 | 0.198 | 1.277 | 0.204 | 0.278 |
| ODI-PLIC | Control (Ref) vs 22q11.2Deletion | -0.562 | 0.182 | -3.086 | 0.003 | 0.006 |
| ODI-PTR | Control (Ref) vs 22q11.2Deletion | 0.791 | 0.179 | 4.426 | 0.000 | 0.000 |
| ODI-RLIC | Control (Ref) vs 22q11.2Deletion | 0.016 | 0.201 | 0.077 | 0.938 | 0.981 |
| ODI-SCC | Control (Ref) vs 22q11.2Deletion | 0.123 | 0.196 | 0.625 | 0.533 | 0.630 |
| ODI-SCR | Control (Ref) vs 22q11.2Deletion | -0.215 | 0.200 | -1.072 | 0.286 | 0.370 |
| ODI-SFO | Control (Ref) vs 22q11.2Deletion | 0.517 | 0.183 | 2.825 | 0.006 | 0.012 |
| ODI-SLF | Control (Ref) vs 22q11.2Deletion | 0.875 | 0.191 | 4.575 | 0.000 | 0.000 |
| ODI-SS | Control (Ref) vs 22q11.2Deletion | 0.548 | 0.209 | 2.616 | 0.010 | 0.020 |
| ODI-TAP | Control (Ref) vs 22q11.2Deletion | -0.090 | 0.226 | -0.396 | 0.693 | 0.787 |
| ICVF | Control (Ref) vs 22q11.2Duplication | -0.932 | 0.168 | -5.543 | 0.000 | 0.000 |
| ISO | Control (Ref) vs 22q11.2Duplication | -0.494 | 0.187 | -2.647 | 0.010 | 0.020 |
| ODI | Control (Ref) vs 22q11.2Duplication | 0.350 | 0.232 | 1.506 | 0.137 | 0.198 |
| ICVF-ACR | Control (Ref) vs 22q11.2Duplication | -1.053 | 0.212 | -4.971 | 0.000 | 0.000 |
| ICVF-ALIC | Control (Ref) vs 22q11.2Duplication | -0.626 | 0.153 | -4.102 | 0.000 | 0.000 |
| ICVF-BCC | Control (Ref) vs 22q11.2Duplication | -1.378 | 0.198 | -6.951 | 0.000 | 0.000 |
| ICVF-CC | Control (Ref) vs 22q11.2Duplication | -1.357 | 0.200 | -6.801 | 0.000 | 0.000 |
| ICVF-CGC | Control (Ref) vs 22q11.2Duplication | -0.967 | 0.176 | -5.506 | 0.000 | 0.000 |
| ICVF-CGH | Control (Ref) vs 22q11.2Duplication | -0.770 | 0.186 | -4.140 | 0.000 | 0.000 |
| ICVF-CR | Control (Ref) vs 22q11.2Duplication | -1.101 | 0.204 | -5.384 | 0.000 | 0.000 |
| ICVF-CST | Control (Ref) vs 22q11.2Duplication | -0.593 | 0.214 | -2.764 | 0.007 | 0.015 |
| ICVF-EC | Control (Ref) vs 22q11.2Duplication | -0.721 | 0.185 | -3.902 | 0.000 | 0.001 |
| ICVF-FX | Control (Ref) vs 22q11.2Duplication | -0.745 | 0.254 | -2.931 | 0.005 | 0.010 |
| ICVF-FXST | Control (Ref) vs 22q11.2Duplication | -0.696 | 0.182 | -3.831 | 0.000 | 0.001 |
| ICVF-GCC | Control (Ref) vs 22q11.2Duplication | -1.154 | 0.215 | -5.361 | 0.000 | 0.000 |
| ICVF-IC | Control (Ref) vs 22q11.2Duplication | -0.884 | 0.169 | -5.239 | 0.000 | 0.000 |
| ICVF-UNC | Control (Ref) vs 22q11.2Duplication | -0.560 | 0.176 | -3.178 | 0.002 | 0.005 |
| ICVF-PCR | Control (Ref) vs 22q11.2Duplication | -1.130 | 0.202 | -5.594 | 0.000 | 0.000 |
| ICVF-PLIC | Control (Ref) vs 22q11.2Duplication | -0.836 | 0.176 | -4.751 | 0.000 | 0.000 |
| ICVF-PTR | Control (Ref) vs 22q11.2Duplication | -1.166 | 0.210 | -5.559 | 0.000 | 0.000 |
| ICVF-RLIC | Control (Ref) vs 22q11.2Duplication | -1.039 | 0.190 | -5.458 | 0.000 | 0.000 |
| ICVF-SCC | Control (Ref) vs 22q11.2Duplication | -1.204 | 0.222 | -5.416 | 0.000 | 0.000 |
| ICVF-SCR | Control (Ref) vs 22q11.2Duplication | -1.105 | 0.204 | -5.417 | 0.000 | 0.000 |
| ICVF-SFO | Control (Ref) vs 22q11.2Duplication | -0.823 | 0.205 | -4.019 | 0.000 | 0.000 |
| ICVF-SLF | Control (Ref) vs 22q11.2Duplication | -1.122 | 0.183 | -6.134 | 0.000 | 0.000 |
| ICVF-SS | Control (Ref) vs 22q11.2Duplication | -0.948 | 0.208 | -4.559 | 0.000 | 0.000 |
| ICVF-TAP | Control (Ref) vs 22q11.2Duplication | -1.056 | 0.236 | -4.469 | 0.000 | 0.000 |
| ISO-ACR | Control (Ref) vs 22q11.2Duplication | -0.414 | 0.237 | -1.748 | 0.085 | 0.134 |
| ISO-ALIC | Control (Ref) vs 22q11.2Duplication | 0.049 | 0.269 | 0.182 | 0.856 | 0.914 |
| ISO-BCC | Control (Ref) vs 22q11.2Duplication | -0.234 | 0.269 | -0.869 | 0.388 | 0.477 |
| ISO-CC | Control (Ref) vs 22q11.2Duplication | -0.184 | 0.275 | -0.669 | 0.506 | 0.600 |
| ISO-CGC | Control (Ref) vs 22q11.2Duplication | -0.289 | 0.250 | -1.158 | 0.251 | 0.329 |
| ISO-CGH | Control (Ref) vs 22q11.2Duplication | -0.004 | 0.244 | -0.017 | 0.986 | 0.989 |
| ISO-CR | Control (Ref) vs 22q11.2Duplication | -0.579 | 0.235 | -2.465 | 0.016 | 0.031 |
| ISO-CST | Control (Ref) vs 22q11.2Duplication | 0.456 | 0.233 | 1.954 | 0.055 | 0.090 |
| ISO-EC | Control (Ref) vs 22q11.2Duplication | -0.267 | 0.186 | -1.433 | 0.156 | 0.221 |
| ISO-FX | Control (Ref) vs 22q11.2Duplication | 0.561 | 0.268 | 2.092 | 0.040 | 0.068 |
| ISO-FXST | Control (Ref) vs 22q11.2Duplication | -0.598 | 0.242 | -2.468 | 0.016 | 0.031 |
| ISO-GCC | Control (Ref) vs 22q11.2Duplication | -0.314 | 0.256 | -1.223 | 0.226 | 0.299 |
| ISO-IC | Control (Ref) vs 22q11.2Duplication | -0.223 | 0.245 | -0.910 | 0.366 | 0.452 |
| ISO-UNC | Control (Ref) vs 22q11.2Duplication | -0.378 | 0.243 | -1.552 | 0.125 | 0.183 |
| ISO-PCR | Control (Ref) vs 22q11.2Duplication | -0.658 | 0.232 | -2.843 | 0.006 | 0.013 |
| ISO-PLIC | Control (Ref) vs 22q11.2Duplication | -0.215 | 0.252 | -0.853 | 0.397 | 0.482 |
| ISO-PTR | Control (Ref) vs 22q11.2Duplication | -0.761 | 0.236 | -3.226 | 0.002 | 0.004 |
| ISO-RLIC | Control (Ref) vs 22q11.2Duplication | -0.374 | 0.229 | -1.631 | 0.108 | 0.161 |
| ISO-SCC | Control (Ref) vs 22q11.2Duplication | 0.130 | 0.306 | 0.424 | 0.673 | 0.774 |
| ISO-SCR | Control (Ref) vs 22q11.2Duplication | -0.649 | 0.230 | -2.819 | 0.006 | 0.013 |
| ISO-SFO | Control (Ref) vs 22q11.2Duplication | -0.624 | 0.264 | -2.364 | 0.021 | 0.039 |
| ISO-SLF | Control (Ref) vs 22q11.2Duplication | -0.672 | 0.217 | -3.094 | 0.003 | 0.006 |
| ISO-SS | Control (Ref) vs 22q11.2Duplication | -0.486 | 0.213 | -2.284 | 0.026 | 0.046 |
| ISO-TAP | Control (Ref) vs 22q11.2Duplication | -0.474 | 0.266 | -1.778 | 0.080 | 0.127 |
| ODI-ACR | Control (Ref) vs 22q11.2Duplication | 0.023 | 0.268 | 0.087 | 0.931 | 0.977 |
| ODI-ALIC | Control (Ref) vs 22q11.2Duplication | 0.661 | 0.238 | 2.776 | 0.007 | 0.015 |
| ODI-BCC | Control (Ref) vs 22q11.2Duplication | 0.260 | 0.262 | 0.993 | 0.324 | 0.414 |
| ODI-CC | Control (Ref) vs 22q11.2Duplication | -0.026 | 0.261 | -0.100 | 0.921 | 0.969 |
| ODI-CGC | Control (Ref) vs 22q11.2Duplication | 0.406 | 0.258 | 1.574 | 0.120 | 0.177 |
| ODI-CGH | Control (Ref) vs 22q11.2Duplication | -0.327 | 0.262 | -1.246 | 0.217 | 0.295 |
| ODI-CR | Control (Ref) vs 22q11.2Duplication | -0.293 | 0.250 | -1.173 | 0.245 | 0.322 |
| ODI-CST | Control (Ref) vs 22q11.2Duplication | 0.318 | 0.266 | 1.194 | 0.237 | 0.313 |
| ODI-EC | Control (Ref) vs 22q11.2Duplication | 0.560 | 0.256 | 2.190 | 0.032 | 0.056 |
| ODI-FX | Control (Ref) vs 22q11.2Duplication | 0.013 | 0.263 | 0.049 | 0.961 | 0.988 |
| ODI-FXST | Control (Ref) vs 22q11.2Duplication | -0.056 | 0.236 | -0.236 | 0.814 | 0.885 |
| ODI-GCC | Control (Ref) vs 22q11.2Duplication | -0.099 | 0.260 | -0.382 | 0.703 | 0.790 |
| ODI-IC | Control (Ref) vs 22q11.2Duplication | 0.322 | 0.234 | 1.376 | 0.173 | 0.242 |
| ODI-UNC | Control (Ref) vs 22q11.2Duplication | 0.734 | 0.278 | 2.641 | 0.010 | 0.020 |
| ODI-PCR | Control (Ref) vs 22q11.2Duplication | -0.536 | 0.253 | -2.117 | 0.038 | 0.065 |
| ODI-PLIC | Control (Ref) vs 22q11.2Duplication | 0.346 | 0.235 | 1.470 | 0.146 | 0.210 |
| ODI-PTR | Control (Ref) vs 22q11.2Duplication | 0.011 | 0.267 | 0.040 | 0.968 | 0.988 |
| ODI-RLIC | Control (Ref) vs 22q11.2Duplication | -0.383 | 0.253 | -1.510 | 0.136 | 0.198 |
| ODI-SCC | Control (Ref) vs 22q11.2Duplication | -0.221 | 0.266 | -0.829 | 0.410 | 0.496 |
| ODI-SCR | Control (Ref) vs 22q11.2Duplication | -0.488 | 0.238 | -2.048 | 0.044 | 0.075 |
| ODI-SFO | Control (Ref) vs 22q11.2Duplication | 0.017 | 0.259 | 0.066 | 0.948 | 0.987 |
| ODI-SLF | Control (Ref) vs 22q11.2Duplication | 0.014 | 0.282 | 0.049 | 0.961 | 0.988 |
| ODI-SS | Control (Ref) vs 22q11.2Duplication | -0.513 | 0.280 | -1.832 | 0.071 | 0.115 |
| ODI-TAP | Control (Ref) vs 22q11.2Duplication | 0.284 | 0.291 | 0.979 | 0.331 | 0.420 |
| ICVF | 22q11.2Deletion (Ref) vs 22q11.2Duplication | -1.428 | 0.141 | -10.133 | 0.000 | 0.000 |
| ISO | 22q11.2Deletion (Ref) vs 22q11.2Duplication | -0.795 | 0.161 | -4.936 | 0.000 | 0.000 |
| ODI | 22q11.2Deletion (Ref) vs 22q11.2Duplication | 0.355 | 0.207 | 1.717 | 0.089 | 0.139 |
| ICVF-ACR | 22q11.2Deletion (Ref) vs 22q11.2Duplication | -1.571 | 0.157 | -9.986 | 0.000 | 0.000 |
| ICVF-ALIC | 22q11.2Deletion (Ref) vs 22q11.2Duplication | -1.266 | 0.121 | -10.476 | 0.000 | 0.000 |
| ICVF-BCC | 22q11.2Deletion (Ref) vs 22q11.2Duplication | -1.665 | 0.149 | -11.145 | 0.000 | 0.000 |
| ICVF-CC | 22q11.2Deletion (Ref) vs 22q11.2Duplication | -1.637 | 0.153 | -10.727 | 0.000 | 0.000 |
| ICVF-CGC | 22q11.2Deletion (Ref) vs 22q11.2Duplication | -1.455 | 0.149 | -9.754 | 0.000 | 0.000 |
| ICVF-CGH | 22q11.2Deletion (Ref) vs 22q11.2Duplication | -1.070 | 0.169 | -6.332 | 0.000 | 0.000 |
| ICVF-CR | 22q11.2Deletion (Ref) vs 22q11.2Duplication | -1.568 | 0.160 | -9.803 | 0.000 | 0.000 |
| ICVF-CST | 22q11.2Deletion (Ref) vs 22q11.2Duplication | -0.930 | 0.188 | -4.934 | 0.000 | 0.000 |
| ICVF-EC | 22q11.2Deletion (Ref) vs 22q11.2Duplication | -1.276 | 0.135 | -9.464 | 0.000 | 0.000 |
| ICVF-FX | 22q11.2Deletion (Ref) vs 22q11.2Duplication | -0.917 | 0.209 | -4.393 | 0.000 | 0.000 |
| ICVF-FXST | 22q11.2Deletion (Ref) vs 22q11.2Duplication | -1.329 | 0.148 | -9.000 | 0.000 | 0.000 |
| ICVF-GCC | 22q11.2Deletion (Ref) vs 22q11.2Duplication | -1.603 | 0.156 | -10.299 | 0.000 | 0.000 |
| ICVF-IC | 22q11.2Deletion (Ref) vs 22q11.2Duplication | -1.450 | 0.139 | -10.459 | 0.000 | 0.000 |
| ICVF-UNC | 22q11.2Deletion (Ref) vs 22q11.2Duplication | -1.309 | 0.149 | -8.802 | 0.000 | 0.000 |
| ICVF-PCR | 22q11.2Deletion (Ref) vs 22q11.2Duplication | -1.551 | 0.165 | -9.412 | 0.000 | 0.000 |
| ICVF-PLIC | 22q11.2Deletion (Ref) vs 22q11.2Duplication | -1.247 | 0.166 | -7.516 | 0.000 | 0.000 |
| ICVF-PTR | 22q11.2Deletion (Ref) vs 22q11.2Duplication | -1.616 | 0.164 | -9.883 | 0.000 | 0.000 |
| ICVF-RLIC | 22q11.2Deletion (Ref) vs 22q11.2Duplication | -1.574 | 0.144 | -10.912 | 0.000 | 0.000 |
| ICVF-SCC | 22q11.2Deletion (Ref) vs 22q11.2Duplication | -1.509 | 0.175 | -8.619 | 0.000 | 0.000 |
| ICVF-SCR | 22q11.2Deletion (Ref) vs 22q11.2Duplication | -1.568 | 0.153 | -10.251 | 0.000 | 0.000 |
| ICVF-SFO | 22q11.2Deletion (Ref) vs 22q11.2Duplication | -1.395 | 0.165 | -8.480 | 0.000 | 0.000 |
| ICVF-SLF | 22q11.2Deletion (Ref) vs 22q11.2Duplication | -1.552 | 0.149 | -10.395 | 0.000 | 0.000 |
| ICVF-SS | 22q11.2Deletion (Ref) vs 22q11.2Duplication | -1.599 | 0.150 | -10.687 | 0.000 | 0.000 |
| ICVF-TAP | 22q11.2Deletion (Ref) vs 22q11.2Duplication | -1.537 | 0.187 | -8.221 | 0.000 | 0.000 |
| ISO-ACR | 22q11.2Deletion (Ref) vs 22q11.2Duplication | -0.860 | 0.197 | -4.370 | 0.000 | 0.000 |
| ISO-ALIC | 22q11.2Deletion (Ref) vs 22q11.2Duplication | -0.297 | 0.243 | -1.220 | 0.226 | 0.299 |
| ISO-BCC | 22q11.2Deletion (Ref) vs 22q11.2Duplication | -0.312 | 0.230 | -1.359 | 0.177 | 0.245 |
| ISO-CC | 22q11.2Deletion (Ref) vs 22q11.2Duplication | -0.094 | 0.247 | -0.382 | 0.703 | 0.790 |
| ISO-CGC | 22q11.2Deletion (Ref) vs 22q11.2Duplication | -0.458 | 0.208 | -2.205 | 0.030 | 0.053 |
| ISO-CGH | 22q11.2Deletion (Ref) vs 22q11.2Duplication | -0.457 | 0.196 | -2.337 | 0.022 | 0.040 |
| ISO-CR | 22q11.2Deletion (Ref) vs 22q11.2Duplication | -0.934 | 0.191 | -4.883 | 0.000 | 0.000 |
| ISO-CST | 22q11.2Deletion (Ref) vs 22q11.2Duplication | 0.363 | 0.221 | 1.642 | 0.104 | 0.157 |
| ISO-EC | 22q11.2Deletion (Ref) vs 22q11.2Duplication | -0.100 | 0.196 | -0.511 | 0.611 | 0.710 |
| ISO-FX | 22q11.2Deletion (Ref) vs 22q11.2Duplication | -0.057 | 0.269 | -0.211 | 0.833 | 0.896 |
| ISO-FXST | 22q11.2Deletion (Ref) vs 22q11.2Duplication | -0.287 | 0.232 | -1.236 | 0.219 | 0.295 |
| ISO-GCC | 22q11.2Deletion (Ref) vs 22q11.2Duplication | -0.232 | 0.240 | -0.967 | 0.336 | 0.420 |
| ISO-IC | 22q11.2Deletion (Ref) vs 22q11.2Duplication | -0.410 | 0.202 | -2.031 | 0.045 | 0.075 |
| ISO-UNC | 22q11.2Deletion (Ref) vs 22q11.2Duplication | -0.395 | 0.218 | -1.810 | 0.074 | 0.117 |
| ISO-PCR | 22q11.2Deletion (Ref) vs 22q11.2Duplication | -0.957 | 0.193 | -4.956 | 0.000 | 0.000 |
| ISO-PLIC | 22q11.2Deletion (Ref) vs 22q11.2Duplication | -0.179 | 0.229 | -0.782 | 0.436 | 0.523 |
| ISO-PTR | 22q11.2Deletion (Ref) vs 22q11.2Duplication | -1.027 | 0.196 | -5.231 | 0.000 | 0.000 |
| ISO-RLIC | 22q11.2Deletion (Ref) vs 22q11.2Duplication | -0.593 | 0.180 | -3.301 | 0.001 | 0.003 |
| ISO-SCC | 22q11.2Deletion (Ref) vs 22q11.2Duplication | 0.245 | 0.261 | 0.936 | 0.352 | 0.436 |
| ISO-SCR | 22q11.2Deletion (Ref) vs 22q11.2Duplication | -0.876 | 0.200 | -4.374 | 0.000 | 0.000 |
| ISO-SFO | 22q11.2Deletion (Ref) vs 22q11.2Duplication | -0.920 | 0.208 | -4.423 | 0.000 | 0.000 |
| ISO-SLF | 22q11.2Deletion (Ref) vs 22q11.2Duplication | -0.897 | 0.184 | -4.872 | 0.000 | 0.000 |
| ISO-SS | 22q11.2Deletion (Ref) vs 22q11.2Duplication | -0.864 | 0.170 | -5.072 | 0.000 | 0.000 |
| ISO-TAP | 22q11.2Deletion (Ref) vs 22q11.2Duplication | -0.739 | 0.223 | -3.320 | 0.001 | 0.003 |
| ODI-ACR | 22q11.2Deletion (Ref) vs 22q11.2Duplication | -0.008 | 0.240 | -0.035 | 0.972 | 0.988 |
| ODI-ALIC | 22q11.2Deletion (Ref) vs 22q11.2Duplication | 1.069 | 0.203 | 5.264 | 0.000 | 0.000 |
| ODI-BCC | 22q11.2Deletion (Ref) vs 22q11.2Duplication | 0.400 | 0.242 | 1.658 | 0.101 | 0.153 |
| ODI-CC | 22q11.2Deletion (Ref) vs 22q11.2Duplication | 0.146 | 0.244 | 0.598 | 0.551 | 0.646 |
| ODI-CGC | 22q11.2Deletion (Ref) vs 22q11.2Duplication | 0.637 | 0.245 | 2.604 | 0.011 | 0.021 |
| ODI-CGH | 22q11.2Deletion (Ref) vs 22q11.2Duplication | -0.643 | 0.240 | -2.677 | 0.009 | 0.018 |
| ODI-CR | 22q11.2Deletion (Ref) vs 22q11.2Duplication | -0.079 | 0.235 | -0.335 | 0.738 | 0.809 |
| ODI-CST | 22q11.2Deletion (Ref) vs 22q11.2Duplication | 0.888 | 0.221 | 4.019 | 0.000 | 0.000 |
| ODI-EC | 22q11.2Deletion (Ref) vs 22q11.2Duplication | 0.013 | 0.234 | 0.055 | 0.956 | 0.988 |
| ODI-FX | 22q11.2Deletion (Ref) vs 22q11.2Duplication | 0.557 | 0.251 | 2.215 | 0.029 | 0.052 |
| ODI-FXST | 22q11.2Deletion (Ref) vs 22q11.2Duplication | -0.520 | 0.225 | -2.314 | 0.023 | 0.042 |
| ODI-GCC | 22q11.2Deletion (Ref) vs 22q11.2Duplication | -0.004 | 0.259 | -0.014 | 0.989 | 0.989 |
| ODI-IC | 22q11.2Deletion (Ref) vs 22q11.2Duplication | 0.750 | 0.217 | 3.456 | 0.001 | 0.002 |
| ODI-UNC | 22q11.2Deletion (Ref) vs 22q11.2Duplication | 0.692 | 0.257 | 2.690 | 0.008 | 0.017 |
| ODI-PCR | 22q11.2Deletion (Ref) vs 22q11.2Duplication | -0.539 | 0.229 | -2.359 | 0.020 | 0.039 |
| ODI-PLIC | 22q11.2Deletion (Ref) vs 22q11.2Duplication | 1.020 | 0.227 | 4.499 | 0.000 | 0.000 |
| ODI-PTR | 22q11.2Deletion (Ref) vs 22q11.2Duplication | -0.852 | 0.219 | -3.892 | 0.000 | 0.000 |
| ODI-RLIC | 22q11.2Deletion (Ref) vs 22q11.2Duplication | -0.331 | 0.229 | -1.447 | 0.151 | 0.215 |
| ODI-SCC | 22q11.2Deletion (Ref) vs 22q11.2Duplication | -0.087 | 0.251 | -0.346 | 0.730 | 0.809 |
| ODI-SCR | 22q11.2Deletion (Ref) vs 22q11.2Duplication | 0.100 | 0.249 | 0.403 | 0.688 | 0.785 |
| ODI-SFO | 22q11.2Deletion (Ref) vs 22q11.2Duplication | -0.389 | 0.247 | -1.573 | 0.119 | 0.176 |
| ODI-SLF | 22q11.2Deletion (Ref) vs 22q11.2Duplication | -0.681 | 0.229 | -2.971 | 0.004 | 0.008 |
| ODI-SS | 22q11.2Deletion (Ref) vs 22q11.2Duplication | -0.877 | 0.233 | -3.771 | 0.000 | 0.001 |
| ODI-TAP | 22q11.2Deletion (Ref) vs 22q11.2Duplication | 0.233 | 0.271 | 0.860 | 0.392 | 0.478 |
| ICVF | GeneDosage (1 = del, 2 = con, 3 dup) | -0.714 | 0.070 | -10.133 | 0.000 | 0.000 |
| ISO | GeneDosage (1 = del, 2 = con, 3 dup) | -0.397 | 0.080 | -4.936 | 0.000 | 0.000 |
| ODI | GeneDosage (1 = del, 2 = con, 3 dup) | 0.178 | 0.103 | 1.717 | 0.089 | 0.139 |
| ICVF-ACR | GeneDosage (1 = del, 2 = con, 3 dup) | -0.786 | 0.079 | -9.986 | 0.000 | 0.000 |
| ICVF-ALIC | GeneDosage (1 = del, 2 = con, 3 dup) | -0.633 | 0.060 | -10.476 | 0.000 | 0.000 |
| ICVF-BCC | GeneDosage (1 = del, 2 = con, 3 dup) | -0.833 | 0.075 | -11.145 | 0.000 | 0.000 |
| ICVF-CC | GeneDosage (1 = del, 2 = con, 3 dup) | -0.819 | 0.076 | -10.727 | 0.000 | 0.000 |
| ICVF-CGC | GeneDosage (1 = del, 2 = con, 3 dup) | -0.727 | 0.075 | -9.754 | 0.000 | 0.000 |
| ICVF-CGH | GeneDosage (1 = del, 2 = con, 3 dup) | -0.535 | 0.085 | -6.332 | 0.000 | 0.000 |
| ICVF-CR | GeneDosage (1 = del, 2 = con, 3 dup) | -0.784 | 0.080 | -9.803 | 0.000 | 0.000 |
| ICVF-CST | GeneDosage (1 = del, 2 = con, 3 dup) | -0.465 | 0.094 | -4.934 | 0.000 | 0.000 |
| ICVF-EC | GeneDosage (1 = del, 2 = con, 3 dup) | -0.638 | 0.067 | -9.464 | 0.000 | 0.000 |
| ICVF-FX | GeneDosage (1 = del, 2 = con, 3 dup) | -0.458 | 0.104 | -4.393 | 0.000 | 0.000 |
| ICVF-FXST | GeneDosage (1 = del, 2 = con, 3 dup) | -0.665 | 0.074 | -9.000 | 0.000 | 0.000 |
| ICVF-GCC | GeneDosage (1 = del, 2 = con, 3 dup) | -0.802 | 0.078 | -10.299 | 0.000 | 0.000 |
| ICVF-IC | GeneDosage (1 = del, 2 = con, 3 dup) | -0.725 | 0.069 | -10.459 | 0.000 | 0.000 |
| ICVF-UNC | GeneDosage (1 = del, 2 = con, 3 dup) | -0.654 | 0.074 | -8.802 | 0.000 | 0.000 |
| ICVF-PCR | GeneDosage (1 = del, 2 = con, 3 dup) | -0.775 | 0.082 | -9.412 | 0.000 | 0.000 |
| ICVF-PLIC | GeneDosage (1 = del, 2 = con, 3 dup) | -0.624 | 0.083 | -7.516 | 0.000 | 0.000 |
| ICVF-PTR | GeneDosage (1 = del, 2 = con, 3 dup) | -0.808 | 0.082 | -9.883 | 0.000 | 0.000 |
| ICVF-RLIC | GeneDosage (1 = del, 2 = con, 3 dup) | -0.787 | 0.072 | -10.912 | 0.000 | 0.000 |
| ICVF-SCC | GeneDosage (1 = del, 2 = con, 3 dup) | -0.755 | 0.088 | -8.619 | 0.000 | 0.000 |
| ICVF-SCR | GeneDosage (1 = del, 2 = con, 3 dup) | -0.784 | 0.076 | -10.251 | 0.000 | 0.000 |
| ICVF-SFO | GeneDosage (1 = del, 2 = con, 3 dup) | -0.698 | 0.082 | -8.480 | 0.000 | 0.000 |
| ICVF-SLF | GeneDosage (1 = del, 2 = con, 3 dup) | -0.776 | 0.075 | -10.395 | 0.000 | 0.000 |
| ICVF-SS | GeneDosage (1 = del, 2 = con, 3 dup) | -0.799 | 0.075 | -10.687 | 0.000 | 0.000 |
| ICVF-TAP | GeneDosage (1 = del, 2 = con, 3 dup) | -0.768 | 0.093 | -8.221 | 0.000 | 0.000 |
| ISO-ACR | GeneDosage (1 = del, 2 = con, 3 dup) | -0.430 | 0.098 | -4.370 | 0.000 | 0.000 |
| ISO-ALIC | GeneDosage (1 = del, 2 = con, 3 dup) | -0.148 | 0.122 | -1.220 | 0.226 | 0.299 |
| ISO-BCC | GeneDosage (1 = del, 2 = con, 3 dup) | -0.156 | 0.115 | -1.359 | 0.177 | 0.245 |
| ISO-CC | GeneDosage (1 = del, 2 = con, 3 dup) | -0.047 | 0.123 | -0.382 | 0.703 | 0.790 |
| ISO-CGC | GeneDosage (1 = del, 2 = con, 3 dup) | -0.229 | 0.104 | -2.205 | 0.030 | 0.053 |
| ISO-CGH | GeneDosage (1 = del, 2 = con, 3 dup) | -0.229 | 0.098 | -2.337 | 0.022 | 0.040 |
| ISO-CR | GeneDosage (1 = del, 2 = con, 3 dup) | -0.467 | 0.096 | -4.883 | 0.000 | 0.000 |
| ISO-CST | GeneDosage (1 = del, 2 = con, 3 dup) | 0.181 | 0.110 | 1.642 | 0.104 | 0.157 |
| ISO-EC | GeneDosage (1 = del, 2 = con, 3 dup) | -0.050 | 0.098 | -0.511 | 0.611 | 0.710 |
| ISO-FX | GeneDosage (1 = del, 2 = con, 3 dup) | -0.028 | 0.135 | -0.211 | 0.833 | 0.896 |
| ISO-FXST | GeneDosage (1 = del, 2 = con, 3 dup) | -0.143 | 0.116 | -1.236 | 0.219 | 0.295 |
| ISO-GCC | GeneDosage (1 = del, 2 = con, 3 dup) | -0.116 | 0.120 | -0.967 | 0.336 | 0.420 |
| ISO-IC | GeneDosage (1 = del, 2 = con, 3 dup) | -0.205 | 0.101 | -2.032 | 0.045 | 0.075 |
| ISO-UNC | GeneDosage (1 = del, 2 = con, 3 dup) | -0.197 | 0.109 | -1.810 | 0.074 | 0.117 |
| ISO-PCR | GeneDosage (1 = del, 2 = con, 3 dup) | -0.479 | 0.097 | -4.956 | 0.000 | 0.000 |
| ISO-PLIC | GeneDosage (1 = del, 2 = con, 3 dup) | -0.089 | 0.114 | -0.782 | 0.436 | 0.523 |
| ISO-PTR | GeneDosage (1 = del, 2 = con, 3 dup) | -0.513 | 0.098 | -5.231 | 0.000 | 0.000 |
| ISO-RLIC | GeneDosage (1 = del, 2 = con, 3 dup) | -0.296 | 0.090 | -3.301 | 0.001 | 0.003 |
| ISO-SCC | GeneDosage (1 = del, 2 = con, 3 dup) | 0.122 | 0.131 | 0.936 | 0.352 | 0.436 |
| ISO-SCR | GeneDosage (1 = del, 2 = con, 3 dup) | -0.438 | 0.100 | -4.374 | 0.000 | 0.000 |
| ISO-SFO | GeneDosage (1 = del, 2 = con, 3 dup) | -0.460 | 0.104 | -4.423 | 0.000 | 0.000 |
| ISO-SLF | GeneDosage (1 = del, 2 = con, 3 dup) | -0.449 | 0.092 | -4.872 | 0.000 | 0.000 |
| ISO-SS | GeneDosage (1 = del, 2 = con, 3 dup) | -0.432 | 0.085 | -5.072 | 0.000 | 0.000 |
| ISO-TAP | GeneDosage (1 = del, 2 = con, 3 dup) | -0.370 | 0.111 | -3.320 | 0.001 | 0.003 |
| ODI-ACR | GeneDosage (1 = del, 2 = con, 3 dup) | -0.004 | 0.120 | -0.035 | 0.972 | 0.988 |
| ODI-ALIC | GeneDosage (1 = del, 2 = con, 3 dup) | 0.535 | 0.102 | 5.264 | 0.000 | 0.000 |
| ODI-BCC | GeneDosage (1 = del, 2 = con, 3 dup) | 0.200 | 0.121 | 1.658 | 0.101 | 0.153 |
| ODI-CC | GeneDosage (1 = del, 2 = con, 3 dup) | 0.073 | 0.122 | 0.598 | 0.551 | 0.646 |
| ODI-CGC | GeneDosage (1 = del, 2 = con, 3 dup) | 0.319 | 0.122 | 2.604 | 0.011 | 0.021 |
| ODI-CGH | GeneDosage (1 = del, 2 = con, 3 dup) | -0.322 | 0.120 | -2.677 | 0.009 | 0.018 |
| ODI-CR | GeneDosage (1 = del, 2 = con, 3 dup) | -0.039 | 0.118 | -0.335 | 0.738 | 0.809 |
| ODI-CST | GeneDosage (1 = del, 2 = con, 3 dup) | 0.444 | 0.110 | 4.019 | 0.000 | 0.000 |
| ODI-EC | GeneDosage (1 = del, 2 = con, 3 dup) | 0.006 | 0.117 | 0.055 | 0.956 | 0.988 |
| ODI-FX | GeneDosage (1 = del, 2 = con, 3 dup) | 0.278 | 0.126 | 2.215 | 0.029 | 0.052 |
| ODI-FXST | GeneDosage (1 = del, 2 = con, 3 dup) | -0.260 | 0.112 | -2.314 | 0.023 | 0.042 |
| ODI-GCC | GeneDosage (1 = del, 2 = con, 3 dup) | -0.002 | 0.130 | -0.014 | 0.989 | 0.989 |
| ODI-IC | GeneDosage (1 = del, 2 = con, 3 dup) | 0.375 | 0.109 | 3.456 | 0.001 | 0.002 |
| ODI-UNC | GeneDosage (1 = del, 2 = con, 3 dup) | 0.346 | 0.129 | 2.690 | 0.008 | 0.017 |
| ODI-PCR | GeneDosage (1 = del, 2 = con, 3 dup) | -0.270 | 0.114 | -2.359 | 0.020 | 0.039 |
| ODI-PLIC | GeneDosage (1 = del, 2 = con, 3 dup) | 0.510 | 0.113 | 4.499 | 0.000 | 0.000 |
| ODI-PTR | GeneDosage (1 = del, 2 = con, 3 dup) | -0.426 | 0.109 | -3.892 | 0.000 | 0.000 |
| ODI-RLIC | GeneDosage (1 = del, 2 = con, 3 dup) | -0.165 | 0.114 | -1.447 | 0.151 | 0.215 |
| ODI-SCC | GeneDosage (1 = del, 2 = con, 3 dup) | -0.043 | 0.125 | -0.346 | 0.730 | 0.809 |
| ODI-SCR | GeneDosage (1 = del, 2 = con, 3 dup) | 0.050 | 0.124 | 0.403 | 0.688 | 0.785 |
| ODI-SFO | GeneDosage (1 = del, 2 = con, 3 dup) | -0.195 | 0.124 | -1.573 | 0.119 | 0.176 |
| ODI-SLF | GeneDosage (1 = del, 2 = con, 3 dup) | -0.340 | 0.115 | -2.971 | 0.004 | 0.008 |
| ODI-SS | GeneDosage (1 = del, 2 = con, 3 dup) | -0.439 | 0.116 | -3.771 | 0.000 | 0.001 |
| ODI-TAP | GeneDosage (1 = del, 2 = con, 3 dup) | 0.117 | 0.136 | 0.860 | 0.392 | 0.478 |

| sTable 8. Sensitivity analysis - Group differences across individuals scanned at the same scanner site, adjusted for cerebrospinal fluid volume | | | | | | |
| --- | --- | --- | --- | --- | --- | --- |
| Dependent variable | **Analysis** | **Std.B.** | **SE** | **T-value** | **P-value** | **pFDR** |
| ICVF | Control (Ref) vs 22q11.2Deletion | 0.657 | 0.138 | 4.764 | 0.000 | 0.000 |
| ISO | Control (Ref) vs 22q11.2Deletion | 0.101 | 0.130 | 0.773 | 0.441 | 0.557 |
| ODI | Control (Ref) vs 22q11.2Deletion | 0.223 | 0.171 | 1.304 | 0.195 | 0.284 |
| ICVF-ACR | Control (Ref) vs 22q11.2Deletion | 1.021 | 0.160 | 6.365 | 0.000 | 0.000 |
| ICVF-ALIC | Control (Ref) vs 22q11.2Deletion | 0.471 | 0.115 | 4.094 | 0.000 | 0.000 |
| ICVF-BCC | Control (Ref) vs 22q11.2Deletion | 1.021 | 0.168 | 6.090 | 0.000 | 0.000 |
| ICVF-CC | Control (Ref) vs 22q11.2Deletion | 1.015 | 0.172 | 5.886 | 0.000 | 0.000 |
| ICVF-CGC | Control (Ref) vs 22q11.2Deletion | 0.536 | 0.151 | 3.545 | 0.001 | 0.001 |
| ICVF-CGH | Control (Ref) vs 22q11.2Deletion | 0.085 | 0.157 | 0.539 | 0.591 | 0.686 |
| ICVF-CR | Control (Ref) vs 22q11.2Deletion | 0.984 | 0.168 | 5.841 | 0.000 | 0.000 |
| ICVF-CST | Control (Ref) vs 22q11.2Deletion | 0.106 | 0.193 | 0.548 | 0.585 | 0.684 |
| ICVF-EC | Control (Ref) vs 22q11.2Deletion | 0.531 | 0.136 | 3.907 | 0.000 | 0.000 |
| ICVF-FX | Control (Ref) vs 22q11.2Deletion | 0.002 | 0.202 | 0.010 | 0.992 | 0.992 |
| ICVF-FXST | Control (Ref) vs 22q11.2Deletion | 0.547 | 0.151 | 3.622 | 0.000 | 0.001 |
| ICVF-GCC | Control (Ref) vs 22q11.2Deletion | 1.114 | 0.166 | 6.707 | 0.000 | 0.000 |
| ICVF-IC | Control (Ref) vs 22q11.2Deletion | 0.693 | 0.137 | 5.064 | 0.000 | 0.000 |
| ICVF-UNC | Control (Ref) vs 22q11.2Deletion | 0.736 | 0.145 | 5.063 | 0.000 | 0.000 |
| ICVF-PCR | Control (Ref) vs 22q11.2Deletion | 0.997 | 0.177 | 5.632 | 0.000 | 0.000 |
| ICVF-PLIC | Control (Ref) vs 22q11.2Deletion | 0.574 | 0.159 | 3.613 | 0.000 | 0.001 |
| ICVF-PTR | Control (Ref) vs 22q11.2Deletion | 1.001 | 0.181 | 5.526 | 0.000 | 0.000 |
| ICVF-RLIC | Control (Ref) vs 22q11.2Deletion | 0.842 | 0.174 | 4.826 | 0.000 | 0.000 |
| ICVF-SCC | Control (Ref) vs 22q11.2Deletion | 0.755 | 0.198 | 3.819 | 0.000 | 0.001 |
| ICVF-SCR | Control (Ref) vs 22q11.2Deletion | 0.851 | 0.175 | 4.873 | 0.000 | 0.000 |
| ICVF-SFO | Control (Ref) vs 22q11.2Deletion | 0.531 | 0.154 | 3.459 | 0.001 | 0.002 |
| ICVF-SLF | Control (Ref) vs 22q11.2Deletion | 0.861 | 0.163 | 5.277 | 0.000 | 0.000 |
| ICVF-SS | Control (Ref) vs 22q11.2Deletion | 0.939 | 0.168 | 5.583 | 0.000 | 0.000 |
| ICVF-TAP | Control (Ref) vs 22q11.2Deletion | 0.955 | 0.199 | 4.803 | 0.000 | 0.000 |
| ISO-ACR | Control (Ref) vs 22q11.2Deletion | 0.377 | 0.176 | 2.142 | 0.034 | 0.064 |
| ISO-ALIC | Control (Ref) vs 22q11.2Deletion | 0.056 | 0.196 | 0.283 | 0.777 | 0.836 |
| ISO-BCC | Control (Ref) vs 22q11.2Deletion | 0.003 | 0.198 | 0.016 | 0.987 | 0.991 |
| ISO-CC | Control (Ref) vs 22q11.2Deletion | -0.085 | 0.192 | -0.442 | 0.659 | 0.741 |
| ISO-CGC | Control (Ref) vs 22q11.2Deletion | -0.017 | 0.174 | -0.098 | 0.922 | 0.957 |
| ISO-CGH | Control (Ref) vs 22q11.2Deletion | 0.092 | 0.180 | 0.510 | 0.611 | 0.697 |
| ISO-CR | Control (Ref) vs 22q11.2Deletion | 0.302 | 0.166 | 1.813 | 0.073 | 0.127 |
| ISO-CST | Control (Ref) vs 22q11.2Deletion | -0.056 | 0.186 | -0.299 | 0.766 | 0.831 |
| ISO-EC | Control (Ref) vs 22q11.2Deletion | -0.122 | 0.167 | -0.729 | 0.468 | 0.575 |
| ISO-FX | Control (Ref) vs 22q11.2Deletion | 0.509 | 0.199 | 2.564 | 0.012 | 0.023 |
| ISO-FXST | Control (Ref) vs 22q11.2Deletion | -0.285 | 0.205 | -1.390 | 0.167 | 0.251 |
| ISO-GCC | Control (Ref) vs 22q11.2Deletion | 0.029 | 0.204 | 0.140 | 0.889 | 0.942 |
| ISO-IC | Control (Ref) vs 22q11.2Deletion | -0.077 | 0.162 | -0.478 | 0.634 | 0.715 |
| ISO-UNC | Control (Ref) vs 22q11.2Deletion | -0.126 | 0.188 | -0.671 | 0.504 | 0.612 |
| ISO-PCR | Control (Ref) vs 22q11.2Deletion | 0.262 | 0.160 | 1.640 | 0.104 | 0.169 |
| ISO-PLIC | Control (Ref) vs 22q11.2Deletion | -0.162 | 0.181 | -0.894 | 0.373 | 0.482 |
| ISO-PTR | Control (Ref) vs 22q11.2Deletion | 0.320 | 0.166 | 1.926 | 0.057 | 0.101 |
| ISO-RLIC | Control (Ref) vs 22q11.2Deletion | -0.085 | 0.159 | -0.538 | 0.592 | 0.686 |
| ISO-SCC | Control (Ref) vs 22q11.2Deletion | -0.222 | 0.174 | -1.277 | 0.204 | 0.296 |
| ISO-SCR | Control (Ref) vs 22q11.2Deletion | 0.114 | 0.173 | 0.659 | 0.512 | 0.615 |
| ISO-SFO | Control (Ref) vs 22q11.2Deletion | 0.132 | 0.200 | 0.657 | 0.512 | 0.615 |
| ISO-SLF | Control (Ref) vs 22q11.2Deletion | 0.155 | 0.140 | 1.103 | 0.272 | 0.371 |
| ISO-SS | Control (Ref) vs 22q11.2Deletion | -0.009 | 0.177 | -0.049 | 0.961 | 0.981 |
| ISO-TAP | Control (Ref) vs 22q11.2Deletion | 0.343 | 0.191 | 1.794 | 0.076 | 0.131 |
| ODI-ACR | Control (Ref) vs 22q11.2Deletion | 0.225 | 0.194 | 1.160 | 0.249 | 0.344 |
| ODI-ALIC | Control (Ref) vs 22q11.2Deletion | -0.332 | 0.191 | -1.735 | 0.086 | 0.147 |
| ODI-BCC | Control (Ref) vs 22q11.2Deletion | 0.122 | 0.185 | 0.664 | 0.508 | 0.615 |
| ODI-CC | Control (Ref) vs 22q11.2Deletion | 0.148 | 0.181 | 0.820 | 0.414 | 0.529 |
| ODI-CGC | Control (Ref) vs 22q11.2Deletion | -0.118 | 0.215 | -0.546 | 0.586 | 0.684 |
| ODI-CGH | Control (Ref) vs 22q11.2Deletion | 0.321 | 0.209 | 1.540 | 0.126 | 0.200 |
| ODI-CR | Control (Ref) vs 22q11.2Deletion | 0.145 | 0.189 | 0.767 | 0.445 | 0.558 |
| ODI-CST | Control (Ref) vs 22q11.2Deletion | -0.476 | 0.211 | -2.262 | 0.026 | 0.049 |
| ODI-EC | Control (Ref) vs 22q11.2Deletion | 0.627 | 0.192 | 3.271 | 0.001 | 0.003 |
| ODI-FX | Control (Ref) vs 22q11.2Deletion | 0.023 | 0.221 | 0.106 | 0.916 | 0.957 |
| ODI-FXST | Control (Ref) vs 22q11.2Deletion | 0.303 | 0.189 | 1.605 | 0.111 | 0.181 |
| ODI-GCC | Control (Ref) vs 22q11.2Deletion | 0.197 | 0.201 | 0.980 | 0.329 | 0.433 |
| ODI-IC | Control (Ref) vs 22q11.2Deletion | -0.354 | 0.179 | -1.978 | 0.050 | 0.092 |
| ODI-UNC | Control (Ref) vs 22q11.2Deletion | -0.079 | 0.210 | -0.374 | 0.709 | 0.791 |
| ODI-PCR | Control (Ref) vs 22q11.2Deletion | 0.290 | 0.196 | 1.485 | 0.140 | 0.219 |
| ODI-PLIC | Control (Ref) vs 22q11.2Deletion | -0.538 | 0.180 | -2.988 | 0.003 | 0.008 |
| ODI-PTR | Control (Ref) vs 22q11.2Deletion | 0.734 | 0.176 | 4.180 | 0.000 | 0.000 |
| ODI-RLIC | Control (Ref) vs 22q11.2Deletion | 0.052 | 0.198 | 0.264 | 0.792 | 0.843 |
| ODI-SCC | Control (Ref) vs 22q11.2Deletion | 0.094 | 0.196 | 0.481 | 0.631 | 0.715 |
| ODI-SCR | Control (Ref) vs 22q11.2Deletion | -0.099 | 0.195 | -0.507 | 0.613 | 0.697 |
| ODI-SFO | Control (Ref) vs 22q11.2Deletion | 0.361 | 0.179 | 2.016 | 0.046 | 0.085 |
| ODI-SLF | Control (Ref) vs 22q11.2Deletion | 0.922 | 0.191 | 4.833 | 0.000 | 0.000 |
| ODI-SS | Control (Ref) vs 22q11.2Deletion | 0.510 | 0.208 | 2.448 | 0.016 | 0.032 |
| ODI-TAP | Control (Ref) vs 22q11.2Deletion | -0.115 | 0.224 | -0.515 | 0.608 | 0.696 |
| ICVF | Control (Ref) vs 22q11.2Duplication | -0.926 | 0.170 | -5.453 | 0.000 | 0.000 |
| ISO | Control (Ref) vs 22q11.2Duplication | -0.500 | 0.186 | -2.691 | 0.009 | 0.018 |
| ODI | Control (Ref) vs 22q11.2Duplication | 0.353 | 0.233 | 1.516 | 0.134 | 0.211 |
| ICVF-ACR | Control (Ref) vs 22q11.2Duplication | -1.029 | 0.217 | -4.740 | 0.000 | 0.000 |
| ICVF-ALIC | Control (Ref) vs 22q11.2Duplication | -0.625 | 0.154 | -4.060 | 0.000 | 0.000 |
| ICVF-BCC | Control (Ref) vs 22q11.2Duplication | -1.359 | 0.206 | -6.604 | 0.000 | 0.000 |
| ICVF-CC | Control (Ref) vs 22q11.2Duplication | -1.337 | 0.209 | -6.384 | 0.000 | 0.000 |
| ICVF-CGC | Control (Ref) vs 22q11.2Duplication | -0.959 | 0.177 | -5.412 | 0.000 | 0.000 |
| ICVF-CGH | Control (Ref) vs 22q11.2Duplication | -0.793 | 0.185 | -4.277 | 0.000 | 0.000 |
| ICVF-CR | Control (Ref) vs 22q11.2Duplication | -1.093 | 0.209 | -5.231 | 0.000 | 0.000 |
| ICVF-CST | Control (Ref) vs 22q11.2Duplication | -0.614 | 0.227 | -2.708 | 0.009 | 0.018 |
| ICVF-EC | Control (Ref) vs 22q11.2Duplication | -0.720 | 0.185 | -3.902 | 0.000 | 0.001 |
| ICVF-FX | Control (Ref) vs 22q11.2Duplication | -0.753 | 0.248 | -3.033 | 0.003 | 0.008 |
| ICVF-FXST | Control (Ref) vs 22q11.2Duplication | -0.695 | 0.183 | -3.806 | 0.000 | 0.001 |
| ICVF-GCC | Control (Ref) vs 22q11.2Duplication | -1.129 | 0.229 | -4.937 | 0.000 | 0.000 |
| ICVF-IC | Control (Ref) vs 22q11.2Duplication | -0.880 | 0.170 | -5.190 | 0.000 | 0.000 |
| ICVF-UNC | Control (Ref) vs 22q11.2Duplication | -0.564 | 0.176 | -3.207 | 0.002 | 0.005 |
| ICVF-PCR | Control (Ref) vs 22q11.2Duplication | -1.127 | 0.210 | -5.364 | 0.000 | 0.000 |
| ICVF-PLIC | Control (Ref) vs 22q11.2Duplication | -0.841 | 0.176 | -4.777 | 0.000 | 0.000 |
| ICVF-PTR | Control (Ref) vs 22q11.2Duplication | -1.175 | 0.210 | -5.583 | 0.000 | 0.000 |
| ICVF-RLIC | Control (Ref) vs 22q11.2Duplication | -1.034 | 0.190 | -5.442 | 0.000 | 0.000 |
| ICVF-SCC | Control (Ref) vs 22q11.2Duplication | -1.201 | 0.229 | -5.240 | 0.000 | 0.000 |
| ICVF-SCR | Control (Ref) vs 22q11.2Duplication | -1.103 | 0.206 | -5.361 | 0.000 | 0.000 |
| ICVF-SFO | Control (Ref) vs 22q11.2Duplication | -0.822 | 0.205 | -4.008 | 0.000 | 0.000 |
| ICVF-SLF | Control (Ref) vs 22q11.2Duplication | -1.116 | 0.188 | -5.953 | 0.000 | 0.000 |
| ICVF-SS | Control (Ref) vs 22q11.2Duplication | -0.943 | 0.210 | -4.492 | 0.000 | 0.000 |
| ICVF-TAP | Control (Ref) vs 22q11.2Duplication | -1.081 | 0.236 | -4.579 | 0.000 | 0.000 |
| ISO-ACR | Control (Ref) vs 22q11.2Duplication | -0.410 | 0.240 | -1.707 | 0.092 | 0.156 |
| ISO-ALIC | Control (Ref) vs 22q11.2Duplication | 0.025 | 0.273 | 0.093 | 0.926 | 0.958 |
| ISO-BCC | Control (Ref) vs 22q11.2Duplication | -0.236 | 0.268 | -0.880 | 0.382 | 0.492 |
| ISO-CC | Control (Ref) vs 22q11.2Duplication | -0.180 | 0.276 | -0.651 | 0.517 | 0.618 |
| ISO-CGC | Control (Ref) vs 22q11.2Duplication | -0.313 | 0.250 | -1.252 | 0.215 | 0.309 |
| ISO-CGH | Control (Ref) vs 22q11.2Duplication | -0.030 | 0.248 | -0.123 | 0.903 | 0.947 |
| ISO-CR | Control (Ref) vs 22q11.2Duplication | -0.578 | 0.235 | -2.455 | 0.017 | 0.033 |
| ISO-CST | Control (Ref) vs 22q11.2Duplication | 0.447 | 0.235 | 1.902 | 0.062 | 0.109 |
| ISO-EC | Control (Ref) vs 22q11.2Duplication | -0.270 | 0.185 | -1.460 | 0.149 | 0.229 |
| ISO-FX | Control (Ref) vs 22q11.2Duplication | 0.540 | 0.243 | 2.226 | 0.029 | 0.055 |
| ISO-FXST | Control (Ref) vs 22q11.2Duplication | -0.596 | 0.243 | -2.456 | 0.017 | 0.033 |
| ISO-GCC | Control (Ref) vs 22q11.2Duplication | -0.310 | 0.255 | -1.214 | 0.229 | 0.327 |
| ISO-IC | Control (Ref) vs 22q11.2Duplication | -0.239 | 0.246 | -0.970 | 0.336 | 0.440 |
| ISO-UNC | Control (Ref) vs 22q11.2Duplication | -0.376 | 0.241 | -1.564 | 0.123 | 0.196 |
| ISO-PCR | Control (Ref) vs 22q11.2Duplication | -0.653 | 0.233 | -2.805 | 0.007 | 0.014 |
| ISO-PLIC | Control (Ref) vs 22q11.2Duplication | -0.229 | 0.254 | -0.903 | 0.370 | 0.481 |
| ISO-PTR | Control (Ref) vs 22q11.2Duplication | -0.768 | 0.233 | -3.297 | 0.002 | 0.004 |
| ISO-RLIC | Control (Ref) vs 22q11.2Duplication | -0.386 | 0.232 | -1.668 | 0.100 | 0.164 |
| ISO-SCC | Control (Ref) vs 22q11.2Duplication | 0.130 | 0.303 | 0.429 | 0.669 | 0.749 |
| ISO-SCR | Control (Ref) vs 22q11.2Duplication | -0.653 | 0.230 | -2.839 | 0.006 | 0.013 |
| ISO-SFO | Control (Ref) vs 22q11.2Duplication | -0.632 | 0.264 | -2.391 | 0.020 | 0.038 |
| ISO-SLF | Control (Ref) vs 22q11.2Duplication | -0.671 | 0.217 | -3.091 | 0.003 | 0.007 |
| ISO-SS | Control (Ref) vs 22q11.2Duplication | -0.508 | 0.211 | -2.407 | 0.019 | 0.037 |
| ISO-TAP | Control (Ref) vs 22q11.2Duplication | -0.474 | 0.267 | -1.771 | 0.081 | 0.140 |
| ODI-ACR | Control (Ref) vs 22q11.2Duplication | 0.027 | 0.268 | 0.100 | 0.921 | 0.957 |
| ODI-ALIC | Control (Ref) vs 22q11.2Duplication | 0.663 | 0.237 | 2.792 | 0.007 | 0.014 |
| ODI-BCC | Control (Ref) vs 22q11.2Duplication | 0.259 | 0.260 | 0.995 | 0.323 | 0.427 |
| ODI-CC | Control (Ref) vs 22q11.2Duplication | -0.019 | 0.264 | -0.071 | 0.944 | 0.973 |
| ODI-CGC | Control (Ref) vs 22q11.2Duplication | 0.395 | 0.266 | 1.488 | 0.141 | 0.220 |
| ODI-CGH | Control (Ref) vs 22q11.2Duplication | -0.325 | 0.260 | -1.252 | 0.215 | 0.309 |
| ODI-CR | Control (Ref) vs 22q11.2Duplication | -0.279 | 0.251 | -1.115 | 0.269 | 0.368 |
| ODI-CST | Control (Ref) vs 22q11.2Duplication | 0.310 | 0.267 | 1.159 | 0.251 | 0.345 |
| ODI-EC | Control (Ref) vs 22q11.2Duplication | 0.551 | 0.257 | 2.141 | 0.036 | 0.066 |
| ODI-FX | Control (Ref) vs 22q11.2Duplication | 0.016 | 0.263 | 0.063 | 0.950 | 0.976 |
| ODI-FXST | Control (Ref) vs 22q11.2Duplication | -0.084 | 0.241 | -0.348 | 0.729 | 0.804 |
| ODI-GCC | Control (Ref) vs 22q11.2Duplication | -0.084 | 0.265 | -0.317 | 0.752 | 0.821 |
| ODI-IC | Control (Ref) vs 22q11.2Duplication | 0.320 | 0.232 | 1.380 | 0.172 | 0.257 |
| ODI-UNC | Control (Ref) vs 22q11.2Duplication | 0.711 | 0.279 | 2.550 | 0.013 | 0.026 |
| ODI-PCR | Control (Ref) vs 22q11.2Duplication | -0.521 | 0.257 | -2.027 | 0.047 | 0.085 |
| ODI-PLIC | Control (Ref) vs 22q11.2Duplication | 0.343 | 0.232 | 1.477 | 0.144 | 0.223 |
| ODI-PTR | Control (Ref) vs 22q11.2Duplication | 0.007 | 0.270 | 0.025 | 0.980 | 0.990 |
| ODI-RLIC | Control (Ref) vs 22q11.2Duplication | -0.391 | 0.252 | -1.548 | 0.126 | 0.200 |
| ODI-SCC | Control (Ref) vs 22q11.2Duplication | -0.225 | 0.270 | -0.833 | 0.408 | 0.523 |
| ODI-SCR | Control (Ref) vs 22q11.2Duplication | -0.465 | 0.237 | -1.959 | 0.054 | 0.098 |
| ODI-SFO | Control (Ref) vs 22q11.2Duplication | -0.005 | 0.254 | -0.018 | 0.986 | 0.991 |
| ODI-SLF | Control (Ref) vs 22q11.2Duplication | 0.016 | 0.279 | 0.057 | 0.954 | 0.977 |
| ODI-SS | Control (Ref) vs 22q11.2Duplication | -0.523 | 0.282 | -1.856 | 0.068 | 0.119 |
| ODI-TAP | Control (Ref) vs 22q11.2Duplication | 0.270 | 0.290 | 0.930 | 0.355 | 0.464 |
| ICVF | 22q11.2Deletion (Ref) vs 22q11.2Duplication | -1.332 | 0.135 | -9.881 | 0.000 | 0.000 |
| ISO | 22q11.2Deletion (Ref) vs 22q11.2Duplication | -0.662 | 0.161 | -4.119 | 0.000 | 0.000 |
| ODI | 22q11.2Deletion (Ref) vs 22q11.2Duplication | 0.227 | 0.212 | 1.071 | 0.287 | 0.384 |
| ICVF-ACR | 22q11.2Deletion (Ref) vs 22q11.2Duplication | -1.566 | 0.141 | -11.071 | 0.000 | 0.000 |
| ICVF-ALIC | 22q11.2Deletion (Ref) vs 22q11.2Duplication | -1.164 | 0.123 | -9.460 | 0.000 | 0.000 |
| ICVF-BCC | 22q11.2Deletion (Ref) vs 22q11.2Duplication | -1.653 | 0.144 | -11.453 | 0.000 | 0.000 |
| ICVF-CC | 22q11.2Deletion (Ref) vs 22q11.2Duplication | -1.636 | 0.149 | -10.996 | 0.000 | 0.000 |
| ICVF-CGC | 22q11.2Deletion (Ref) vs 22q11.2Duplication | -1.338 | 0.150 | -8.933 | 0.000 | 0.000 |
| ICVF-CGH | 22q11.2Deletion (Ref) vs 22q11.2Duplication | -0.847 | 0.173 | -4.902 | 0.000 | 0.000 |
| ICVF-CR | 22q11.2Deletion (Ref) vs 22q11.2Duplication | -1.537 | 0.154 | -9.964 | 0.000 | 0.000 |
| ICVF-CST | 22q11.2Deletion (Ref) vs 22q11.2Duplication | -0.666 | 0.198 | -3.356 | 0.001 | 0.003 |
| ICVF-EC | 22q11.2Deletion (Ref) vs 22q11.2Duplication | -1.150 | 0.140 | -8.235 | 0.000 | 0.000 |
| ICVF-FX | 22q11.2Deletion (Ref) vs 22q11.2Duplication | -0.647 | 0.227 | -2.855 | 0.005 | 0.012 |
| ICVF-FXST | 22q11.2Deletion (Ref) vs 22q11.2Duplication | -1.141 | 0.150 | -7.632 | 0.000 | 0.000 |
| ICVF-GCC | 22q11.2Deletion (Ref) vs 22q11.2Duplication | -1.646 | 0.153 | -10.725 | 0.000 | 0.000 |
| ICVF-IC | 22q11.2Deletion (Ref) vs 22q11.2Duplication | -1.325 | 0.141 | -9.409 | 0.000 | 0.000 |
| ICVF-UNC | 22q11.2Deletion (Ref) vs 22q11.2Duplication | -1.171 | 0.157 | -7.458 | 0.000 | 0.000 |
| ICVF-PCR | 22q11.2Deletion (Ref) vs 22q11.2Duplication | -1.494 | 0.158 | -9.454 | 0.000 | 0.000 |
| ICVF-PLIC | 22q11.2Deletion (Ref) vs 22q11.2Duplication | -1.130 | 0.170 | -6.632 | 0.000 | 0.000 |
| ICVF-PTR | 22q11.2Deletion (Ref) vs 22q11.2Duplication | -1.566 | 0.155 | -10.102 | 0.000 | 0.000 |
| ICVF-RLIC | 22q11.2Deletion (Ref) vs 22q11.2Duplication | -1.477 | 0.141 | -10.464 | 0.000 | 0.000 |
| ICVF-SCC | 22q11.2Deletion (Ref) vs 22q11.2Duplication | -1.454 | 0.171 | -8.488 | 0.000 | 0.000 |
| ICVF-SCR | 22q11.2Deletion (Ref) vs 22q11.2Duplication | -1.515 | 0.148 | -10.255 | 0.000 | 0.000 |
| ICVF-SFO | 22q11.2Deletion (Ref) vs 22q11.2Duplication | -1.304 | 0.161 | -8.120 | 0.000 | 0.000 |
| ICVF-SLF | 22q11.2Deletion (Ref) vs 22q11.2Duplication | -1.499 | 0.145 | -10.316 | 0.000 | 0.000 |
| ICVF-SS | 22q11.2Deletion (Ref) vs 22q11.2Duplication | -1.480 | 0.144 | -10.291 | 0.000 | 0.000 |
| ICVF-TAP | 22q11.2Deletion (Ref) vs 22q11.2Duplication | -1.454 | 0.182 | -7.972 | 0.000 | 0.000 |
| ISO-ACR | 22q11.2Deletion (Ref) vs 22q11.2Duplication | -0.802 | 0.196 | -4.096 | 0.000 | 0.000 |
| ISO-ALIC | 22q11.2Deletion (Ref) vs 22q11.2Duplication | -0.179 | 0.242 | -0.740 | 0.461 | 0.569 |
| ISO-BCC | 22q11.2Deletion (Ref) vs 22q11.2Duplication | -0.270 | 0.229 | -1.178 | 0.242 | 0.342 |
| ISO-CC | 22q11.2Deletion (Ref) vs 22q11.2Duplication | 0.006 | 0.245 | 0.026 | 0.979 | 0.990 |
| ISO-CGC | 22q11.2Deletion (Ref) vs 22q11.2Duplication | -0.353 | 0.210 | -1.685 | 0.095 | 0.159 |
| ISO-CGH | 22q11.2Deletion (Ref) vs 22q11.2Duplication | -0.237 | 0.202 | -1.173 | 0.244 | 0.342 |
| ISO-CR | 22q11.2Deletion (Ref) vs 22q11.2Duplication | -0.858 | 0.190 | -4.525 | 0.000 | 0.000 |
| ISO-CST | 22q11.2Deletion (Ref) vs 22q11.2Duplication | 0.385 | 0.222 | 1.733 | 0.086 | 0.147 |
| ISO-EC | 22q11.2Deletion (Ref) vs 22q11.2Duplication | -0.051 | 0.192 | -0.266 | 0.791 | 0.843 |
| ISO-FX | 22q11.2Deletion (Ref) vs 22q11.2Duplication | 0.031 | 0.255 | 0.123 | 0.903 | 0.947 |
| ISO-FXST | 22q11.2Deletion (Ref) vs 22q11.2Duplication | -0.248 | 0.231 | -1.073 | 0.286 | 0.384 |
| ISO-GCC | 22q11.2Deletion (Ref) vs 22q11.2Duplication | -0.153 | 0.241 | -0.636 | 0.526 | 0.624 |
| ISO-IC | 22q11.2Deletion (Ref) vs 22q11.2Duplication | -0.265 | 0.201 | -1.321 | 0.190 | 0.280 |
| ISO-UNC | 22q11.2Deletion (Ref) vs 22q11.2Duplication | -0.296 | 0.225 | -1.315 | 0.192 | 0.280 |
| ISO-PCR | 22q11.2Deletion (Ref) vs 22q11.2Duplication | -0.873 | 0.189 | -4.606 | 0.000 | 0.000 |
| ISO-PLIC | 22q11.2Deletion (Ref) vs 22q11.2Duplication | -0.126 | 0.227 | -0.556 | 0.580 | 0.682 |
| ISO-PTR | 22q11.2Deletion (Ref) vs 22q11.2Duplication | -0.893 | 0.190 | -4.692 | 0.000 | 0.000 |
| ISO-RLIC | 22q11.2Deletion (Ref) vs 22q11.2Duplication | -0.396 | 0.177 | -2.230 | 0.028 | 0.053 |
| ISO-SCC | 22q11.2Deletion (Ref) vs 22q11.2Duplication | 0.368 | 0.259 | 1.422 | 0.158 | 0.239 |
| ISO-SCR | 22q11.2Deletion (Ref) vs 22q11.2Duplication | -0.807 | 0.199 | -4.062 | 0.000 | 0.000 |
| ISO-SFO | 22q11.2Deletion (Ref) vs 22q11.2Duplication | -0.833 | 0.209 | -3.994 | 0.000 | 0.000 |
| ISO-SLF | 22q11.2Deletion (Ref) vs 22q11.2Duplication | -0.846 | 0.182 | -4.651 | 0.000 | 0.000 |
| ISO-SS | 22q11.2Deletion (Ref) vs 22q11.2Duplication | -0.644 | 0.172 | -3.749 | 0.000 | 0.001 |
| ISO-TAP | 22q11.2Deletion (Ref) vs 22q11.2Duplication | -0.614 | 0.222 | -2.766 | 0.007 | 0.014 |
| ODI-ACR | 22q11.2Deletion (Ref) vs 22q11.2Duplication | -0.070 | 0.238 | -0.293 | 0.770 | 0.831 |
| ODI-ALIC | 22q11.2Deletion (Ref) vs 22q11.2Duplication | 1.071 | 0.200 | 5.362 | 0.000 | 0.000 |
| ODI-BCC | 22q11.2Deletion (Ref) vs 22q11.2Duplication | 0.393 | 0.236 | 1.665 | 0.099 | 0.164 |
| ODI-CC | 22q11.2Deletion (Ref) vs 22q11.2Duplication | 0.079 | 0.240 | 0.329 | 0.743 | 0.814 |
| ODI-CGC | 22q11.2Deletion (Ref) vs 22q11.2Duplication | 0.633 | 0.240 | 2.641 | 0.010 | 0.020 |
| ODI-CGH | 22q11.2Deletion (Ref) vs 22q11.2Duplication | -0.686 | 0.236 | -2.904 | 0.005 | 0.010 |
| ODI-CR | 22q11.2Deletion (Ref) vs 22q11.2Duplication | -0.274 | 0.234 | -1.167 | 0.246 | 0.342 |
| ODI-CST | 22q11.2Deletion (Ref) vs 22q11.2Duplication | 0.815 | 0.218 | 3.736 | 0.000 | 0.001 |
| ODI-EC | 22q11.2Deletion (Ref) vs 22q11.2Duplication | -0.127 | 0.240 | -0.528 | 0.599 | 0.688 |
| ODI-FX | 22q11.2Deletion (Ref) vs 22q11.2Duplication | 0.367 | 0.253 | 1.448 | 0.151 | 0.230 |
| ODI-FXST | 22q11.2Deletion (Ref) vs 22q11.2Duplication | -0.342 | 0.214 | -1.601 | 0.113 | 0.181 |
| ODI-GCC | 22q11.2Deletion (Ref) vs 22q11.2Duplication | -0.093 | 0.255 | -0.366 | 0.715 | 0.792 |
| ODI-IC | 22q11.2Deletion (Ref) vs 22q11.2Duplication | 0.698 | 0.209 | 3.343 | 0.001 | 0.003 |
| ODI-UNC | 22q11.2Deletion (Ref) vs 22q11.2Duplication | 0.696 | 0.247 | 2.818 | 0.006 | 0.013 |
| ODI-PCR | 22q11.2Deletion (Ref) vs 22q11.2Duplication | -0.746 | 0.225 | -3.311 | 0.001 | 0.003 |
| ODI-PLIC | 22q11.2Deletion (Ref) vs 22q11.2Duplication | 0.972 | 0.216 | 4.499 | 0.000 | 0.000 |
| ODI-PTR | 22q11.2Deletion (Ref) vs 22q11.2Duplication | -0.891 | 0.217 | -4.113 | 0.000 | 0.000 |
| ODI-RLIC | 22q11.2Deletion (Ref) vs 22q11.2Duplication | -0.427 | 0.222 | -1.927 | 0.057 | 0.101 |
| ODI-SCC | 22q11.2Deletion (Ref) vs 22q11.2Duplication | -0.171 | 0.248 | -0.691 | 0.491 | 0.599 |
| ODI-SCR | 22q11.2Deletion (Ref) vs 22q11.2Duplication | -0.182 | 0.240 | -0.759 | 0.450 | 0.560 |
| ODI-SFO | 22q11.2Deletion (Ref) vs 22q11.2Duplication | -0.239 | 0.240 | -0.994 | 0.323 | 0.427 |
| ODI-SLF | 22q11.2Deletion (Ref) vs 22q11.2Duplication | -0.810 | 0.232 | -3.500 | 0.001 | 0.002 |
| ODI-SS | 22q11.2Deletion (Ref) vs 22q11.2Duplication | -0.952 | 0.230 | -4.136 | 0.000 | 0.000 |
| ODI-TAP | 22q11.2Deletion (Ref) vs 22q11.2Duplication | 0.207 | 0.264 | 0.784 | 0.435 | 0.551 |
| ICVF | GeneDosage (1 = del, 2 = con, 3 dup) | -0.666 | 0.067 | -9.881 | 0.000 | 0.000 |
| ISO | GeneDosage (1 = del, 2 = con, 3 dup) | -0.331 | 0.080 | -4.119 | 0.000 | 0.000 |
| ODI | GeneDosage (1 = del, 2 = con, 3 dup) | 0.113 | 0.106 | 1.071 | 0.287 | 0.384 |
| ICVF-ACR | GeneDosage (1 = del, 2 = con, 3 dup) | -0.783 | 0.071 | -11.071 | 0.000 | 0.000 |
| ICVF-ALIC | GeneDosage (1 = del, 2 = con, 3 dup) | -0.582 | 0.062 | -9.460 | 0.000 | 0.000 |
| ICVF-BCC | GeneDosage (1 = del, 2 = con, 3 dup) | -0.827 | 0.072 | -11.453 | 0.000 | 0.000 |
| ICVF-CC | GeneDosage (1 = del, 2 = con, 3 dup) | -0.818 | 0.074 | -10.996 | 0.000 | 0.000 |
| ICVF-CGC | GeneDosage (1 = del, 2 = con, 3 dup) | -0.669 | 0.075 | -8.933 | 0.000 | 0.000 |
| ICVF-CGH | GeneDosage (1 = del, 2 = con, 3 dup) | -0.424 | 0.086 | -4.902 | 0.000 | 0.000 |
| ICVF-CR | GeneDosage (1 = del, 2 = con, 3 dup) | -0.769 | 0.077 | -9.964 | 0.000 | 0.000 |
| ICVF-CST | GeneDosage (1 = del, 2 = con, 3 dup) | -0.333 | 0.099 | -3.356 | 0.001 | 0.003 |
| ICVF-EC | GeneDosage (1 = del, 2 = con, 3 dup) | -0.575 | 0.070 | -8.235 | 0.000 | 0.000 |
| ICVF-FX | GeneDosage (1 = del, 2 = con, 3 dup) | -0.324 | 0.113 | -2.855 | 0.005 | 0.012 |
| ICVF-FXST | GeneDosage (1 = del, 2 = con, 3 dup) | -0.571 | 0.075 | -7.632 | 0.000 | 0.000 |
| ICVF-GCC | GeneDosage (1 = del, 2 = con, 3 dup) | -0.823 | 0.077 | -10.725 | 0.000 | 0.000 |
| ICVF-IC | GeneDosage (1 = del, 2 = con, 3 dup) | -0.663 | 0.070 | -9.409 | 0.000 | 0.000 |
| ICVF-UNC | GeneDosage (1 = del, 2 = con, 3 dup) | -0.586 | 0.079 | -7.458 | 0.000 | 0.000 |
| ICVF-PCR | GeneDosage (1 = del, 2 = con, 3 dup) | -0.747 | 0.079 | -9.454 | 0.000 | 0.000 |
| ICVF-PLIC | GeneDosage (1 = del, 2 = con, 3 dup) | -0.565 | 0.085 | -6.632 | 0.000 | 0.000 |
| ICVF-PTR | GeneDosage (1 = del, 2 = con, 3 dup) | -0.783 | 0.078 | -10.102 | 0.000 | 0.000 |
| ICVF-RLIC | GeneDosage (1 = del, 2 = con, 3 dup) | -0.739 | 0.071 | -10.464 | 0.000 | 0.000 |
| ICVF-SCC | GeneDosage (1 = del, 2 = con, 3 dup) | -0.727 | 0.086 | -8.488 | 0.000 | 0.000 |
| ICVF-SCR | GeneDosage (1 = del, 2 = con, 3 dup) | -0.758 | 0.074 | -10.255 | 0.000 | 0.000 |
| ICVF-SFO | GeneDosage (1 = del, 2 = con, 3 dup) | -0.652 | 0.080 | -8.120 | 0.000 | 0.000 |
| ICVF-SLF | GeneDosage (1 = del, 2 = con, 3 dup) | -0.749 | 0.073 | -10.316 | 0.000 | 0.000 |
| ICVF-SS | GeneDosage (1 = del, 2 = con, 3 dup) | -0.740 | 0.072 | -10.290 | 0.000 | 0.000 |
| ICVF-TAP | GeneDosage (1 = del, 2 = con, 3 dup) | -0.727 | 0.091 | -7.972 | 0.000 | 0.000 |
| ISO-ACR | GeneDosage (1 = del, 2 = con, 3 dup) | -0.401 | 0.098 | -4.095 | 0.000 | 0.000 |
| ISO-ALIC | GeneDosage (1 = del, 2 = con, 3 dup) | -0.090 | 0.121 | -0.740 | 0.461 | 0.569 |
| ISO-BCC | GeneDosage (1 = del, 2 = con, 3 dup) | -0.135 | 0.114 | -1.178 | 0.242 | 0.342 |
| ISO-CC | GeneDosage (1 = del, 2 = con, 3 dup) | 0.003 | 0.123 | 0.026 | 0.979 | 0.990 |
| ISO-CGC | GeneDosage (1 = del, 2 = con, 3 dup) | -0.177 | 0.105 | -1.685 | 0.095 | 0.159 |
| ISO-CGH | GeneDosage (1 = del, 2 = con, 3 dup) | -0.119 | 0.101 | -1.173 | 0.244 | 0.342 |
| ISO-CR | GeneDosage (1 = del, 2 = con, 3 dup) | -0.429 | 0.095 | -4.525 | 0.000 | 0.000 |
| ISO-CST | GeneDosage (1 = del, 2 = con, 3 dup) | 0.193 | 0.111 | 1.733 | 0.086 | 0.147 |
| ISO-EC | GeneDosage (1 = del, 2 = con, 3 dup) | -0.026 | 0.096 | -0.266 | 0.791 | 0.843 |
| ISO-FX | GeneDosage (1 = del, 2 = con, 3 dup) | 0.016 | 0.127 | 0.123 | 0.903 | 0.947 |
| ISO-FXST | GeneDosage (1 = del, 2 = con, 3 dup) | -0.124 | 0.115 | -1.073 | 0.286 | 0.384 |
| ISO-GCC | GeneDosage (1 = del, 2 = con, 3 dup) | -0.077 | 0.120 | -0.636 | 0.526 | 0.624 |
| ISO-IC | GeneDosage (1 = del, 2 = con, 3 dup) | -0.132 | 0.100 | -1.321 | 0.190 | 0.280 |
| ISO-UNC | GeneDosage (1 = del, 2 = con, 3 dup) | -0.148 | 0.113 | -1.318 | 0.191 | 0.280 |
| ISO-PCR | GeneDosage (1 = del, 2 = con, 3 dup) | -0.436 | 0.095 | -4.606 | 0.000 | 0.000 |
| ISO-PLIC | GeneDosage (1 = del, 2 = con, 3 dup) | -0.063 | 0.114 | -0.556 | 0.580 | 0.682 |
| ISO-PTR | GeneDosage (1 = del, 2 = con, 3 dup) | -0.446 | 0.095 | -4.692 | 0.000 | 0.000 |
| ISO-RLIC | GeneDosage (1 = del, 2 = con, 3 dup) | -0.198 | 0.089 | -2.230 | 0.028 | 0.053 |
| ISO-SCC | GeneDosage (1 = del, 2 = con, 3 dup) | 0.184 | 0.129 | 1.422 | 0.158 | 0.239 |
| ISO-SCR | GeneDosage (1 = del, 2 = con, 3 dup) | -0.403 | 0.099 | -4.062 | 0.000 | 0.000 |
| ISO-SFO | GeneDosage (1 = del, 2 = con, 3 dup) | -0.417 | 0.104 | -3.994 | 0.000 | 0.000 |
| ISO-SLF | GeneDosage (1 = del, 2 = con, 3 dup) | -0.423 | 0.091 | -4.654 | 0.000 | 0.000 |
| ISO-SS | GeneDosage (1 = del, 2 = con, 3 dup) | -0.322 | 0.086 | -3.749 | 0.000 | 0.001 |
| ISO-TAP | GeneDosage (1 = del, 2 = con, 3 dup) | -0.307 | 0.111 | -2.766 | 0.007 | 0.014 |
| ODI-ACR | GeneDosage (1 = del, 2 = con, 3 dup) | -0.035 | 0.119 | -0.293 | 0.770 | 0.831 |
| ODI-ALIC | GeneDosage (1 = del, 2 = con, 3 dup) | 0.536 | 0.100 | 5.362 | 0.000 | 0.000 |
| ODI-BCC | GeneDosage (1 = del, 2 = con, 3 dup) | 0.197 | 0.118 | 1.665 | 0.099 | 0.164 |
| ODI-CC | GeneDosage (1 = del, 2 = con, 3 dup) | 0.040 | 0.120 | 0.329 | 0.743 | 0.814 |
| ODI-CGC | GeneDosage (1 = del, 2 = con, 3 dup) | 0.316 | 0.120 | 2.642 | 0.010 | 0.020 |
| ODI-CGH | GeneDosage (1 = del, 2 = con, 3 dup) | -0.343 | 0.118 | -2.904 | 0.005 | 0.010 |
| ODI-CR | GeneDosage (1 = del, 2 = con, 3 dup) | -0.137 | 0.117 | -1.167 | 0.246 | 0.342 |
| ODI-CST | GeneDosage (1 = del, 2 = con, 3 dup) | 0.407 | 0.109 | 3.736 | 0.000 | 0.001 |
| ODI-EC | GeneDosage (1 = del, 2 = con, 3 dup) | -0.063 | 0.120 | -0.528 | 0.599 | 0.688 |
| ODI-FX | GeneDosage (1 = del, 2 = con, 3 dup) | 0.183 | 0.127 | 1.448 | 0.151 | 0.230 |
| ODI-FXST | GeneDosage (1 = del, 2 = con, 3 dup) | -0.171 | 0.107 | -1.601 | 0.113 | 0.181 |
| ODI-GCC | GeneDosage (1 = del, 2 = con, 3 dup) | -0.047 | 0.128 | -0.366 | 0.715 | 0.792 |
| ODI-IC | GeneDosage (1 = del, 2 = con, 3 dup) | 0.349 | 0.104 | 3.343 | 0.001 | 0.003 |
| ODI-UNC | GeneDosage (1 = del, 2 = con, 3 dup) | 0.348 | 0.123 | 2.818 | 0.006 | 0.013 |
| ODI-PCR | GeneDosage (1 = del, 2 = con, 3 dup) | -0.373 | 0.113 | -3.311 | 0.001 | 0.003 |
| ODI-PLIC | GeneDosage (1 = del, 2 = con, 3 dup) | 0.486 | 0.108 | 4.499 | 0.000 | 0.000 |
| ODI-PTR | GeneDosage (1 = del, 2 = con, 3 dup) | -0.446 | 0.108 | -4.113 | 0.000 | 0.000 |
| ODI-RLIC | GeneDosage (1 = del, 2 = con, 3 dup) | -0.214 | 0.111 | -1.927 | 0.057 | 0.101 |
| ODI-SCC | GeneDosage (1 = del, 2 = con, 3 dup) | -0.086 | 0.124 | -0.691 | 0.491 | 0.599 |
| ODI-SCR | GeneDosage (1 = del, 2 = con, 3 dup) | -0.091 | 0.120 | -0.759 | 0.450 | 0.560 |
| ODI-SFO | GeneDosage (1 = del, 2 = con, 3 dup) | -0.119 | 0.120 | -0.994 | 0.323 | 0.427 |
| ODI-SLF | GeneDosage (1 = del, 2 = con, 3 dup) | -0.405 | 0.116 | -3.500 | 0.001 | 0.002 |
| ODI-SS | GeneDosage (1 = del, 2 = con, 3 dup) | -0.476 | 0.115 | -4.136 | 0.000 | 0.000 |
| ODI-TAP | GeneDosage (1 = del, 2 = con, 3 dup) | 0.103 | 0.132 | 0.784 | 0.435 | 0.551 |

| sTable 9. Age effect on the NODDI measures for the control group | | | | | | |
| --- | --- | --- | --- | --- | --- | --- |
| Dependent variable | **Group** | **Edf** | **Ref.df** | **F** | **P-value** | **pFDR** |
| ICVF.combat | Control | 5.083 | 5.083 | 187.115 | 0.000 | 0.000 |
| ISO.combat | Control | 4.945 | 4.945 | 402.623 | 0.000 | 0.000 |
| ODI.combat | Control | 4.661 | 4.661 | 233.516 | 0.000 | 0.000 |
| ICVF.ACR.combat | Control | 4.332 | 4.332 | 81.846 | 0.000 | 0.000 |
| ICVF.ALIC.combat | Control | 4.700 | 4.700 | 313.189 | 0.000 | 0.000 |
| ICVF.BCC.combat | Control | 4.125 | 4.125 | 23.067 | 0.000 | 0.000 |
| ICVF.CC.combat | Control | 4.185 | 4.185 | 23.987 | 0.000 | 0.000 |
| ICVF.CGC.combat | Control | 4.658 | 4.658 | 102.146 | 0.000 | 0.000 |
| ICVF.CGH.combat | Control | 4.381 | 4.381 | 209.642 | 0.000 | 0.000 |
| ICVF.CR.combat | Control | 4.554 | 4.554 | 82.411 | 0.000 | 0.000 |
| ICVF.CST.combat | Control | 5.178 | 5.178 | 65.999 | 0.000 | 0.000 |
| ICVF.EC.combat | Control | 4.782 | 4.782 | 161.321 | 0.000 | 0.000 |
| ICVF.FX.combat | Control | 3.244 | 3.244 | 45.601 | 0.000 | 0.000 |
| ICVF.FXST.combat | Control | 4.555 | 4.555 | 128.783 | 0.000 | 0.000 |
| ICVF.GCC.combat | Control | 4.016 | 4.016 | 13.069 | 0.000 | 0.000 |
| ICVF.IC.combat | Control | 4.785 | 4.785 | 172.386 | 0.000 | 0.000 |
| ICVF.UNC.combat | Control | 4.706 | 4.706 | 178.963 | 0.000 | 0.000 |
| ICVF.PCR.combat | Control | 4.825 | 4.825 | 64.879 | 0.000 | 0.000 |
| ICVF.PLIC.combat | Control | 4.673 | 4.673 | 150.544 | 0.000 | 0.000 |
| ICVF.PTR.combat | Control | 4.541 | 4.541 | 34.723 | 0.000 | 0.000 |
| ICVF.RLIC.combat | Control | 4.643 | 4.643 | 73.145 | 0.000 | 0.000 |
| ICVF.SCC.combat | Control | 4.327 | 4.327 | 28.920 | 0.000 | 0.000 |
| ICVF.SCR.combat | Control | 4.622 | 4.622 | 76.302 | 0.000 | 0.000 |
| ICVF.SFO.combat | Control | 4.670 | 4.670 | 137.968 | 0.000 | 0.000 |
| ICVF.SLF.combat | Control | 4.769 | 4.769 | 79.917 | 0.000 | 0.000 |
| ICVF.SS.combat | Control | 4.326 | 4.326 | 61.178 | 0.000 | 0.000 |
| ICVF.TAP.combat | Control | 4.393 | 4.393 | 33.582 | 0.000 | 0.000 |
| ISO.ACR.combat | Control | 4.499 | 4.499 | 154.903 | 0.000 | 0.000 |
| ISO.ALIC.combat | Control | 5.380 | 5.380 | 81.515 | 0.000 | 0.000 |
| ISO.BCC.combat | Control | 3.490 | 3.490 | 177.260 | 0.000 | 0.000 |
| ISO.CC.combat | Control | 4.224 | 4.224 | 162.608 | 0.000 | 0.000 |
| ISO.CGC.combat | Control | 3.918 | 3.918 | 152.540 | 0.000 | 0.000 |
| ISO.CGH.combat | Control | 3.633 | 3.633 | 166.949 | 0.000 | 0.000 |
| ISO.CR.combat | Control | 4.725 | 4.725 | 196.275 | 0.000 | 0.000 |
| ISO.CST.combat | Control | 5.668 | 5.668 | 41.362 | 0.000 | 0.000 |
| ISO.EC.combat | Control | 4.297 | 4.297 | 167.037 | 0.000 | 0.000 |
| ISO.FX.combat | Control | 1.737 | 1.737 | 196.978 | 0.000 | 0.000 |
| ISO.FXST.combat | Control | 3.763 | 3.763 | 84.140 | 0.000 | 0.000 |
| ISO.GCC.combat | Control | 4.067 | 4.067 | 85.162 | 0.000 | 0.000 |
| ISO.IC.combat | Control | 5.731 | 5.731 | 151.658 | 0.000 | 0.000 |
| ISO.UNC.combat | Control | 2.992 | 2.992 | 159.862 | 0.000 | 0.000 |
| ISO.PCR.combat | Control | 4.706 | 4.706 | 207.322 | 0.000 | 0.000 |
| ISO.PLIC.combat | Control | 5.719 | 5.719 | 44.784 | 0.000 | 0.000 |
| ISO.PTR.combat | Control | 3.961 | 3.961 | 119.601 | 0.000 | 0.000 |
| ISO.RLIC.combat | Control | 4.874 | 4.874 | 200.577 | 0.000 | 0.000 |
| ISO.SCC.combat | Control | 4.860 | 4.860 | 121.538 | 0.000 | 0.000 |
| ISO.SCR.combat | Control | 4.711 | 4.711 | 182.589 | 0.000 | 0.000 |
| ISO.SFO.combat | Control | 5.355 | 5.355 | 85.186 | 0.000 | 0.000 |
| ISO.SLF.combat | Control | 4.022 | 4.022 | 215.168 | 0.000 | 0.000 |
| ISO.SS.combat | Control | 4.653 | 4.653 | 140.296 | 0.000 | 0.000 |
| ISO.TAP.combat | Control | 3.736 | 3.736 | 64.179 | 0.000 | 0.000 |
| ODI.ACR.combat | Control | 3.072 | 3.072 | 112.501 | 0.000 | 0.000 |
| ODI.ALIC.combat | Control | 1.000 | 1.000 | 468.145 | 0.000 | 0.000 |
| ODI.BCC.combat | Control | 2.287 | 2.287 | 104.619 | 0.000 | 0.000 |
| ODI.CC.combat | Control | 2.945 | 2.945 | 92.214 | 0.000 | 0.000 |
| ODI.CGC.combat | Control | 3.028 | 3.028 | 4.654 | 0.003 | 0.003 |
| ODI.CGH.combat | Control | 3.500 | 3.500 | 50.195 | 0.000 | 0.000 |
| ODI.CR.combat | Control | 2.886 | 2.886 | 128.781 | 0.000 | 0.000 |
| ODI.CST.combat | Control | 1.000 | 1.000 | 74.309 | 0.000 | 0.000 |
| ODI.EC.combat | Control | 1.000 | 1.000 | 229.620 | 0.000 | 0.000 |
| ODI.FX.combat | Control | 1.690 | 1.690 | 4.221 | 0.012 | 0.012 |
| ODI.FXST.combat | Control | 3.722 | 3.722 | 82.805 | 0.000 | 0.000 |
| ODI.GCC.combat | Control | 2.875 | 2.875 | 70.328 | 0.000 | 0.000 |
| ODI.IC.combat | Control | 2.741 | 2.741 | 220.170 | 0.000 | 0.000 |
| ODI.UNC.combat | Control | 1.568 | 1.568 | 92.070 | 0.000 | 0.000 |
| ODI.PCR.combat | Control | 3.084 | 3.084 | 70.154 | 0.000 | 0.000 |
| ODI.PLIC.combat | Control | 3.620 | 3.620 | 118.132 | 0.000 | 0.000 |
| ODI.PTR.combat | Control | 2.069 | 2.069 | 103.185 | 0.000 | 0.000 |
| ODI.RLIC.combat | Control | 2.614 | 2.614 | 109.292 | 0.000 | 0.000 |
| ODI.SCC.combat | Control | 3.448 | 3.448 | 49.998 | 0.000 | 0.000 |
| ODI.SCR.combat | Control | 1.715 | 1.715 | 125.508 | 0.000 | 0.000 |
| ODI.SFO.combat | Control | 1.000 | 1.000 | 346.009 | 0.000 | 0.000 |
| ODI.SLF.combat | Control | 1.000 | 1.000 | 126.664 | 0.000 | 0.000 |
| ODI.SS.combat | Control | 1.000 | 1.000 | 88.650 | 0.000 | 0.000 |
| ODI.TAP.combat | Control | 2.436 | 2.436 | 1.764 | 0.239 | 0.239 |

| sTable 10. Age*22q11.2 CNV interaction effect on NODDI measures. | | | | | | |
| --- | --- | --- | --- | --- | --- | --- |
| Dependent variable | **Group** | **Edf** | **Ref.df** | **F** | **P-value** | **pFDR** |
| ICVF.combat | 22qDeletion | 1.000 | 1.000 | 0.108 | 0.742 | 0.800 |
| ICVF.combat | 22qDuplication | 2.160 | 2.160 | 3.477 | 0.027 | 0.229 |
| ISO.combat | 22qDeletion | 1.521 | 1.521 | 2.054 | 0.255 | 0.516 |
| ISO.combat | 22qDuplication | 1.000 | 1.000 | 8.052 | 0.005 | 0.135 |
| ODI.combat | 22qDeletion | 1.000 | 1.000 | 0.268 | 0.605 | 0.766 |
| ODI.combat | 22qDuplication | 1.000 | 1.000 | 5.170 | 0.023 | 0.229 |
| ICVF.ACR.combat | 22qDeletion | 1.847 | 1.847 | 0.788 | 0.487 | 0.689 |
| ICVF.ACR.combat | 22qDuplication | 2.348 | 2.348 | 3.035 | 0.037 | 0.229 |
| ICVF.ALIC.combat | 22qDeletion | 1.404 | 1.404 | 1.220 | 0.415 | 0.676 |
| ICVF.ALIC.combat | 22qDuplication | 2.706 | 2.706 | 3.145 | 0.033 | 0.229 |
| ICVF.BCC.combat | 22qDeletion | 1.463 | 1.463 | 0.225 | 0.608 | 0.766 |
| ICVF.BCC.combat | 22qDuplication | 2.122 | 2.122 | 1.568 | 0.225 | 0.478 |
| ICVF.CC.combat | 22qDeletion | 1.597 | 1.597 | 0.324 | 0.554 | 0.741 |
| ICVF.CC.combat | 22qDuplication | 2.015 | 2.015 | 1.466 | 0.234 | 0.487 |
| ICVF.CGC.combat | 22qDeletion | 1.000 | 1.000 | 0.021 | 0.884 | 0.915 |
| ICVF.CGC.combat | 22qDuplication | 1.474 | 1.474 | 1.340 | 0.168 | 0.394 |
| ICVF.CGH.combat | 22qDeletion | 1.000 | 1.000 | 1.389 | 0.239 | 0.491 |
| ICVF.CGH.combat | 22qDuplication | 2.368 | 2.368 | 2.290 | 0.080 | 0.272 |
| ICVF.CR.combat | 22qDeletion | 1.411 | 1.411 | 0.391 | 0.740 | 0.800 |
| ICVF.CR.combat | 22qDuplication | 2.266 | 2.266 | 2.886 | 0.047 | 0.265 |
| ICVF.CST.combat | 22qDeletion | 1.000 | 1.000 | 0.006 | 0.938 | 0.958 |
| ICVF.CST.combat | 22qDuplication | 1.000 | 1.000 | 3.236 | 0.072 | 0.265 |
| ICVF.EC.combat | 22qDeletion | 1.000 | 1.000 | 1.082 | 0.298 | 0.547 |
| ICVF.EC.combat | 22qDuplication | 2.107 | 2.107 | 3.587 | 0.033 | 0.229 |
| ICVF.FX.combat | 22qDeletion | 1.000 | 1.000 | 0.104 | 0.747 | 0.800 |
| ICVF.FX.combat | 22qDuplication | 3.770 | 3.770 | 0.763 | 0.461 | 0.676 |
| ICVF.FXST.combat | 22qDeletion | 1.000 | 1.000 | 0.001 | 0.980 | 0.980 |
| ICVF.FXST.combat | 22qDuplication | 2.175 | 2.175 | 2.568 | 0.099 | 0.317 |
| ICVF.GCC.combat | 22qDeletion | 1.490 | 1.490 | 0.187 | 0.729 | 0.800 |
| ICVF.GCC.combat | 22qDuplication | 2.394 | 2.394 | 1.877 | 0.156 | 0.379 |
| ICVF.IC.combat | 22qDeletion | 1.155 | 1.155 | 0.281 | 0.728 | 0.800 |
| ICVF.IC.combat | 22qDuplication | 2.216 | 2.216 | 3.266 | 0.036 | 0.229 |
| ICVF.UNC.combat | 22qDeletion | 1.000 | 1.000 | 3.456 | 0.063 | 0.265 |
| ICVF.UNC.combat | 22qDuplication | 1.093 | 1.093 | 1.924 | 0.144 | 0.376 |
| ICVF.PCR.combat | 22qDeletion | 1.000 | 1.000 | 0.001 | 0.974 | 0.980 |
| ICVF.PCR.combat | 22qDuplication | 2.167 | 2.167 | 2.398 | 0.085 | 0.279 |
| ICVF.PLIC.combat | 22qDeletion | 1.000 | 1.000 | 0.493 | 0.483 | 0.689 |
| ICVF.PLIC.combat | 22qDuplication | 2.105 | 2.105 | 2.601 | 0.066 | 0.265 |
| ICVF.PTR.combat | 22qDeletion | 1.000 | 1.000 | 0.158 | 0.691 | 0.800 |
| ICVF.PTR.combat | 22qDuplication | 2.037 | 2.037 | 1.657 | 0.200 | 0.455 |
| ICVF.RLIC.combat | 22qDeletion | 1.000 | 1.000 | 0.127 | 0.722 | 0.800 |
| ICVF.RLIC.combat | 22qDuplication | 1.383 | 1.383 | 2.609 | 0.061 | 0.265 |
| ICVF.SCC.combat | 22qDeletion | 1.411 | 1.411 | 0.489 | 0.422 | 0.676 |
| ICVF.SCC.combat | 22qDuplication | 1.000 | 1.000 | 2.027 | 0.155 | 0.379 |
| ICVF.SCR.combat | 22qDeletion | 1.000 | 1.000 | 0.129 | 0.719 | 0.800 |
| ICVF.SCR.combat | 22qDuplication | 2.035 | 2.035 | 2.432 | 0.085 | 0.279 |
| ICVF.SFO.combat | 22qDeletion | 1.000 | 1.000 | 0.584 | 0.445 | 0.676 |
| ICVF.SFO.combat | 22qDuplication | 3.029 | 3.029 | 3.433 | 0.016 | 0.210 |
| ICVF.SLF.combat | 22qDeletion | 1.000 | 1.000 | 0.004 | 0.950 | 0.963 |
| ICVF.SLF.combat | 22qDuplication | 1.743 | 1.743 | 3.022 | 0.032 | 0.229 |
| ICVF.SS.combat | 22qDeletion | 1.000 | 1.000 | 0.399 | 0.528 | 0.719 |
| ICVF.SS.combat | 22qDuplication | 2.002 | 2.002 | 1.889 | 0.152 | 0.379 |
| ICVF.TAP.combat | 22qDeletion | 1.000 | 1.000 | 0.107 | 0.744 | 0.800 |
| ICVF.TAP.combat | 22qDuplication | 2.319 | 2.319 | 2.254 | 0.133 | 0.376 |
| ISO.ACR.combat | 22qDeletion | 1.323 | 1.323 | 6.140 | 0.019 | 0.216 |
| ISO.ACR.combat | 22qDuplication | 1.000 | 1.000 | 9.736 | 0.002 | 0.093 |
| ISO.ALIC.combat | 22qDeletion | 1.000 | 1.000 | 2.624 | 0.106 | 0.329 |
| ISO.ALIC.combat | 22qDuplication | 1.000 | 1.000 | 7.277 | 0.007 | 0.135 |
| ISO.BCC.combat | 22qDeletion | 1.676 | 1.676 | 2.326 | 0.226 | 0.478 |
| ISO.BCC.combat | 22qDuplication | 1.000 | 1.000 | 7.167 | 0.008 | 0.135 |
| ISO.CC.combat | 22qDeletion | 1.000 | 1.000 | 2.222 | 0.136 | 0.376 |
| ISO.CC.combat | 22qDuplication | 1.000 | 1.000 | 10.463 | 0.001 | 0.093 |
| ISO.CGC.combat | 22qDeletion | 1.103 | 1.103 | 1.658 | 0.221 | 0.478 |
| ISO.CGC.combat | 22qDuplication | 1.000 | 1.000 | 0.559 | 0.455 | 0.676 |
| ISO.CGH.combat | 22qDeletion | 1.970 | 1.970 | 1.289 | 0.300 | 0.547 |
| ISO.CGH.combat | 22qDuplication | 1.000 | 1.000 | 3.563 | 0.059 | 0.265 |
| ISO.CR.combat | 22qDeletion | 1.524 | 1.524 | 4.143 | 0.073 | 0.265 |
| ISO.CR.combat | 22qDuplication | 1.000 | 1.000 | 9.057 | 0.003 | 0.101 |
| ISO.CST.combat | 22qDeletion | 2.152 | 2.152 | 1.982 | 0.140 | 0.376 |
| ISO.CST.combat | 22qDuplication | 1.000 | 1.000 | 0.227 | 0.634 | 0.786 |
| ISO.EC.combat | 22qDeletion | 2.299 | 2.299 | 1.332 | 0.404 | 0.676 |
| ISO.EC.combat | 22qDuplication | 1.109 | 1.109 | 2.747 | 0.108 | 0.329 |
| ISO.FX.combat | 22qDeletion | 1.000 | 1.000 | 0.201 | 0.654 | 0.794 |
| ISO.FX.combat | 22qDuplication | 4.849 | 4.849 | 3.183 | 0.027 | 0.229 |
| ISO.FXST.combat | 22qDeletion | 2.238 | 2.238 | 2.930 | 0.064 | 0.265 |
| ISO.FXST.combat | 22qDuplication | 1.000 | 1.000 | 0.342 | 0.559 | 0.741 |
| ISO.GCC.combat | 22qDeletion | 1.000 | 1.000 | 4.426 | 0.036 | 0.229 |
| ISO.GCC.combat | 22qDuplication | 1.000 | 1.000 | 5.908 | 0.015 | 0.210 |
| ISO.IC.combat | 22qDeletion | 1.697 | 1.697 | 0.343 | 0.714 | 0.800 |
| ISO.IC.combat | 22qDuplication | 1.000 | 1.000 | 3.722 | 0.054 | 0.265 |
| ISO.UNC.combat | 22qDeletion | 1.000 | 1.000 | 0.585 | 0.444 | 0.676 |
| ISO.UNC.combat | 22qDuplication | 1.000 | 1.000 | 1.952 | 0.163 | 0.387 |
| ISO.PCR.combat | 22qDeletion | 1.629 | 1.629 | 4.179 | 0.074 | 0.265 |
| ISO.PCR.combat | 22qDuplication | 1.445 | 1.445 | 7.233 | 0.008 | 0.135 |
| ISO.PLIC.combat | 22qDeletion | 1.000 | 1.000 | 2.458 | 0.117 | 0.352 |
| ISO.PLIC.combat | 22qDuplication | 2.083 | 2.083 | 1.177 | 0.288 | 0.547 |
| ISO.PTR.combat | 22qDeletion | 1.000 | 1.000 | 0.130 | 0.719 | 0.800 |
| ISO.PTR.combat | 22qDuplication | 1.961 | 1.961 | 4.178 | 0.028 | 0.229 |
| ISO.RLIC.combat | 22qDeletion | 1.940 | 1.940 | 0.618 | 0.590 | 0.758 |
| ISO.RLIC.combat | 22qDuplication | 1.000 | 1.000 | 3.423 | 0.065 | 0.265 |
| ISO.SCC.combat | 22qDeletion | 1.000 | 1.000 | 0.149 | 0.699 | 0.800 |
| ISO.SCC.combat | 22qDuplication | 3.690 | 3.690 | 5.899 | 0.001 | 0.093 |
| ISO.SCR.combat | 22qDeletion | 1.477 | 1.477 | 1.231 | 0.435 | 0.676 |
| ISO.SCR.combat | 22qDuplication | 1.494 | 1.494 | 2.472 | 0.060 | 0.265 |
| ISO.SFO.combat | 22qDeletion | 1.562 | 1.562 | 1.831 | 0.303 | 0.547 |
| ISO.SFO.combat | 22qDuplication | 1.000 | 1.000 | 2.123 | 0.145 | 0.376 |
| ISO.SLF.combat | 22qDeletion | 1.000 | 1.000 | 1.139 | 0.286 | 0.547 |
| ISO.SLF.combat | 22qDuplication | 1.000 | 1.000 | 3.127 | 0.077 | 0.270 |
| ISO.SS.combat | 22qDeletion | 1.000 | 1.000 | 1.473 | 0.225 | 0.478 |
| ISO.SS.combat | 22qDuplication | 1.000 | 1.000 | 1.238 | 0.266 | 0.525 |
| ISO.TAP.combat | 22qDeletion | 1.000 | 1.000 | 0.334 | 0.563 | 0.741 |
| ISO.TAP.combat | 22qDuplication | 2.138 | 2.138 | 3.067 | 0.038 | 0.229 |
| ODI.ACR.combat | 22qDeletion | 1.000 | 1.000 | 0.245 | 0.621 | 0.776 |
| ODI.ACR.combat | 22qDuplication | 1.725 | 1.725 | 1.599 | 0.322 | 0.568 |
| ODI.ALIC.combat | 22qDeletion | 1.000 | 1.000 | 1.281 | 0.258 | 0.516 |
| ODI.ALIC.combat | 22qDuplication | 1.000 | 1.000 | 3.222 | 0.073 | 0.265 |
| ODI.BCC.combat | 22qDeletion | 2.248 | 2.248 | 3.479 | 0.017 | 0.210 |
| ODI.BCC.combat | 22qDuplication | 1.000 | 1.000 | 2.159 | 0.142 | 0.376 |
| ODI.CC.combat | 22qDeletion | 1.812 | 1.812 | 4.353 | 0.058 | 0.265 |
| ODI.CC.combat | 22qDuplication | 1.000 | 1.000 | 0.664 | 0.415 | 0.676 |
| ODI.CGC.combat | 22qDeletion | 1.000 | 1.000 | 0.443 | 0.506 | 0.702 |
| ODI.CGC.combat | 22qDuplication | 1.838 | 1.838 | 1.051 | 0.425 | 0.676 |
| ODI.CGH.combat | 22qDeletion | 1.000 | 1.000 | 0.537 | 0.464 | 0.676 |
| ODI.CGH.combat | 22qDuplication | 1.000 | 1.000 | 0.012 | 0.912 | 0.937 |
| ODI.CR.combat | 22qDeletion | 1.000 | 1.000 | 0.916 | 0.339 | 0.584 |
| ODI.CR.combat | 22qDuplication | 1.000 | 1.000 | 1.555 | 0.213 | 0.476 |
| ODI.CST.combat | 22qDeletion | 1.000 | 1.000 | 0.540 | 0.462 | 0.676 |
| ODI.CST.combat | 22qDuplication | 1.000 | 1.000 | 0.204 | 0.652 | 0.794 |
| ODI.EC.combat | 22qDeletion | 1.000 | 1.000 | 0.164 | 0.685 | 0.800 |
| ODI.EC.combat | 22qDuplication | 1.000 | 1.000 | 1.097 | 0.295 | 0.547 |
| ODI.FX.combat | 22qDeletion | 1.000 | 1.000 | 2.090 | 0.149 | 0.378 |
| ODI.FX.combat | 22qDuplication | 2.382 | 2.382 | 1.245 | 0.458 | 0.676 |
| ODI.FXST.combat | 22qDeletion | 1.329 | 1.329 | 0.705 | 0.583 | 0.758 |
| ODI.FXST.combat | 22qDuplication | 1.000 | 1.000 | 0.519 | 0.471 | 0.680 |
| ODI.GCC.combat | 22qDeletion | 1.000 | 1.000 | 0.054 | 0.817 | 0.869 |
| ODI.GCC.combat | 22qDuplication | 1.000 | 1.000 | 0.982 | 0.322 | 0.568 |
| ODI.IC.combat | 22qDeletion | 1.000 | 1.000 | 0.040 | 0.841 | 0.882 |
| ODI.IC.combat | 22qDuplication | 1.172 | 1.172 | 0.883 | 0.421 | 0.676 |
| ODI.UNC.combat | 22qDeletion | 1.000 | 1.000 | 2.242 | 0.135 | 0.376 |
| ODI.UNC.combat | 22qDuplication | 1.000 | 1.000 | 2.171 | 0.141 | 0.376 |
| ODI.PCR.combat | 22qDeletion | 1.000 | 1.000 | 1.867 | 0.172 | 0.397 |
| ODI.PCR.combat | 22qDuplication | 1.000 | 1.000 | 3.836 | 0.050 | 0.265 |
| ODI.PLIC.combat | 22qDeletion | 1.000 | 1.000 | 0.035 | 0.851 | 0.886 |
| ODI.PLIC.combat | 22qDuplication | 1.716 | 1.716 | 0.953 | 0.492 | 0.690 |
| ODI.PTR.combat | 22qDeletion | 1.000 | 1.000 | 4.601 | 0.032 | 0.229 |
| ODI.PTR.combat | 22qDuplication | 1.000 | 1.000 | 0.334 | 0.563 | 0.741 |
| ODI.RLIC.combat | 22qDeletion | 1.000 | 1.000 | 0.427 | 0.514 | 0.707 |
| ODI.RLIC.combat | 22qDuplication | 1.000 | 1.000 | 0.172 | 0.678 | 0.800 |
| ODI.SCC.combat | 22qDeletion | 2.781 | 2.781 | 3.703 | 0.008 | 0.135 |
| ODI.SCC.combat | 22qDuplication | 1.000 | 1.000 | 0.289 | 0.591 | 0.758 |
| ODI.SCR.combat | 22qDeletion | 1.000 | 1.000 | 1.100 | 0.295 | 0.547 |
| ODI.SCR.combat | 22qDuplication | 1.000 | 1.000 | 0.177 | 0.674 | 0.800 |
| ODI.SFO.combat | 22qDeletion | 2.026 | 2.026 | 2.704 | 0.067 | 0.265 |
| ODI.SFO.combat | 22qDuplication | 1.000 | 1.000 | 0.947 | 0.331 | 0.577 |
| ODI.SLF.combat | 22qDeletion | 1.000 | 1.000 | 0.142 | 0.706 | 0.800 |
| ODI.SLF.combat | 22qDuplication | 1.000 | 1.000 | 3.295 | 0.070 | 0.265 |
| ODI.SS.combat | 22qDeletion | 1.833 | 1.833 | 0.695 | 0.436 | 0.676 |
| ODI.SS.combat | 22qDuplication | 1.000 | 1.000 | 0.198 | 0.656 | 0.794 |
| ODI.TAP.combat | 22qDeletion | 1.000 | 1.000 | 0.610 | 0.435 | 0.676 |
| ODI.TAP.combat | 22qDuplication | 1.051 | 1.051 | 0.053 | 0.837 | 0.882 |

| sTable 11. Group comparisons by binned age groups. | | | | | | | |
| --- | --- | --- | --- | --- | --- | --- | --- |
| Dependent Variable | **Analysis** | **Std.B.** | **S.E.** | **T-value** | **P-value** | **pFDR** | **Age group** |
| ICVF.combat | Control (Ref) vs 22q11.2Deletion | 0.555 | 0.145 | 3.840 | 0.000 | 0.001 | 7-15years |
| ISO.combat | Control (Ref) vs 22q11.2Deletion | 0.349 | 0.133 | 2.618 | 0.009 | 0.021 | 7-15years |
| ODI.combat | Control (Ref) vs 22q11.2Deletion | 0.385 | 0.157 | 2.453 | 0.015 | 0.032 | 7-15years |
| ICVF.ACR.combat | Control (Ref) vs 22q11.2Deletion | 0.848 | 0.159 | 5.319 | 0.000 | 0.000 | 7-15years |
| ICVF.ALIC.combat | Control (Ref) vs 22q11.2Deletion | 0.689 | 0.148 | 4.650 | 0.000 | 0.000 | 7-15years |
| ICVF.BCC.combat | Control (Ref) vs 22q11.2Deletion | 0.741 | 0.164 | 4.523 | 0.000 | 0.000 | 7-15years |
| ICVF.CC.combat | Control (Ref) vs 22q11.2Deletion | 0.747 | 0.165 | 4.517 | 0.000 | 0.000 | 7-15years |
| ICVF.CGC.combat | Control (Ref) vs 22q11.2Deletion | 0.424 | 0.151 | 2.811 | 0.005 | 0.013 | 7-15years |
| ICVF.CGH.combat | Control (Ref) vs 22q11.2Deletion | 0.117 | 0.155 | 0.758 | 0.449 | 0.550 | 7-15years |
| ICVF.CR.combat | Control (Ref) vs 22q11.2Deletion | 0.799 | 0.158 | 5.072 | 0.000 | 0.000 | 7-15years |
| ICVF.CST.combat | Control (Ref) vs 22q11.2Deletion | 0.207 | 0.148 | 1.398 | 0.163 | 0.244 | 7-15years |
| ICVF.EC.combat | Control (Ref) vs 22q11.2Deletion | 0.317 | 0.150 | 2.109 | 0.035 | 0.067 | 7-15years |
| ICVF.FX.combat | Control (Ref) vs 22q11.2Deletion | 0.190 | 0.166 | 1.147 | 0.252 | 0.352 | 7-15years |
| ICVF.FXST.combat | Control (Ref) vs 22q11.2Deletion | 0.506 | 0.158 | 3.194 | 0.001 | 0.004 | 7-15years |
| ICVF.GCC.combat | Control (Ref) vs 22q11.2Deletion | 0.787 | 0.166 | 4.733 | 0.000 | 0.000 | 7-15years |
| ICVF.IC.combat | Control (Ref) vs 22q11.2Deletion | 0.702 | 0.152 | 4.611 | 0.000 | 0.000 | 7-15years |
| ICVF.UNC.combat | Control (Ref) vs 22q11.2Deletion | 0.576 | 0.138 | 4.174 | 0.000 | 0.000 | 7-15years |
| ICVF.PCR.combat | Control (Ref) vs 22q11.2Deletion | 0.778 | 0.156 | 4.985 | 0.000 | 0.000 | 7-15years |
| ICVF.PLIC.combat | Control (Ref) vs 22q11.2Deletion | 0.663 | 0.153 | 4.327 | 0.000 | 0.000 | 7-15years |
| ICVF.PTR.combat | Control (Ref) vs 22q11.2Deletion | 0.683 | 0.155 | 4.410 | 0.000 | 0.000 | 7-15years |
| ICVF.RLIC.combat | Control (Ref) vs 22q11.2Deletion | 0.652 | 0.155 | 4.204 | 0.000 | 0.000 | 7-15years |
| ICVF.SCC.combat | Control (Ref) vs 22q11.2Deletion | 0.558 | 0.164 | 3.401 | 0.001 | 0.002 | 7-15years |
| ICVF.SCR.combat | Control (Ref) vs 22q11.2Deletion | 0.664 | 0.157 | 4.238 | 0.000 | 0.000 | 7-15years |
| ICVF.SFO.combat | Control (Ref) vs 22q11.2Deletion | 0.553 | 0.155 | 3.560 | 0.000 | 0.002 | 7-15years |
| ICVF.SLF.combat | Control (Ref) vs 22q11.2Deletion | 0.637 | 0.153 | 4.171 | 0.000 | 0.000 | 7-15years |
| ICVF.SS.combat | Control (Ref) vs 22q11.2Deletion | 0.667 | 0.154 | 4.321 | 0.000 | 0.000 | 7-15years |
| ICVF.TAP.combat | Control (Ref) vs 22q11.2Deletion | 0.756 | 0.163 | 4.636 | 0.000 | 0.000 | 7-15years |
| ISO.ACR.combat | Control (Ref) vs 22q11.2Deletion | 0.497 | 0.142 | 3.488 | 0.001 | 0.002 | 7-15years |
| ISO.ALIC.combat | Control (Ref) vs 22q11.2Deletion | 0.162 | 0.154 | 1.051 | 0.294 | 0.394 | 7-15years |
| ISO.BCC.combat | Control (Ref) vs 22q11.2Deletion | 0.177 | 0.157 | 1.127 | 0.260 | 0.360 | 7-15years |
| ISO.CC.combat | Control (Ref) vs 22q11.2Deletion | 0.100 | 0.149 | 0.672 | 0.502 | 0.598 | 7-15years |
| ISO.CGC.combat | Control (Ref) vs 22q11.2Deletion | 0.136 | 0.144 | 0.948 | 0.344 | 0.450 | 7-15years |
| ISO.CGH.combat | Control (Ref) vs 22q11.2Deletion | 0.265 | 0.156 | 1.691 | 0.091 | 0.153 | 7-15years |
| ISO.CR.combat | Control (Ref) vs 22q11.2Deletion | 0.473 | 0.139 | 3.395 | 0.001 | 0.002 | 7-15years |
| ISO.CST.combat | Control (Ref) vs 22q11.2Deletion | -0.206 | 0.145 | -1.420 | 0.156 | 0.237 | 7-15years |
| ISO.EC.combat | Control (Ref) vs 22q11.2Deletion | 0.245 | 0.146 | 1.679 | 0.094 | 0.156 | 7-15years |
| ISO.FX.combat | Control (Ref) vs 22q11.2Deletion | 0.573 | 0.165 | 3.484 | 0.001 | 0.002 | 7-15years |
| ISO.FXST.combat | Control (Ref) vs 22q11.2Deletion | -0.084 | 0.152 | -0.548 | 0.584 | 0.674 | 7-15years |
| ISO.GCC.combat | Control (Ref) vs 22q11.2Deletion | 0.249 | 0.148 | 1.691 | 0.092 | 0.153 | 7-15years |
| ISO.IC.combat | Control (Ref) vs 22q11.2Deletion | 0.058 | 0.145 | 0.404 | 0.687 | 0.760 | 7-15years |
| ISO.UNC.combat | Control (Ref) vs 22q11.2Deletion | -0.066 | 0.160 | -0.415 | 0.678 | 0.754 | 7-15years |
| ISO.PCR.combat | Control (Ref) vs 22q11.2Deletion | 0.453 | 0.143 | 3.161 | 0.002 | 0.005 | 7-15years |
| ISO.PLIC.combat | Control (Ref) vs 22q11.2Deletion | -0.233 | 0.148 | -1.575 | 0.116 | 0.185 | 7-15years |
| ISO.PTR.combat | Control (Ref) vs 22q11.2Deletion | 0.237 | 0.148 | 1.603 | 0.110 | 0.177 | 7-15years |
| ISO.RLIC.combat | Control (Ref) vs 22q11.2Deletion | 0.217 | 0.150 | 1.453 | 0.147 | 0.226 | 7-15years |
| ISO.SCC.combat | Control (Ref) vs 22q11.2Deletion | -0.152 | 0.141 | -1.083 | 0.280 | 0.379 | 7-15years |
| ISO.SCR.combat | Control (Ref) vs 22q11.2Deletion | 0.287 | 0.151 | 1.908 | 0.057 | 0.102 | 7-15years |
| ISO.SFO.combat | Control (Ref) vs 22q11.2Deletion | 0.338 | 0.152 | 2.220 | 0.027 | 0.052 | 7-15years |
| ISO.SLF.combat | Control (Ref) vs 22q11.2Deletion | 0.320 | 0.145 | 2.203 | 0.028 | 0.054 | 7-15years |
| ISO.SS.combat | Control (Ref) vs 22q11.2Deletion | 0.215 | 0.153 | 1.409 | 0.159 | 0.240 | 7-15years |
| ISO.TAP.combat | Control (Ref) vs 22q11.2Deletion | 0.359 | 0.162 | 2.225 | 0.027 | 0.052 | 7-15years |
| ODI.ACR.combat | Control (Ref) vs 22q11.2Deletion | 0.312 | 0.169 | 1.846 | 0.066 | 0.116 | 7-15years |
| ODI.ALIC.combat | Control (Ref) vs 22q11.2Deletion | -0.143 | 0.169 | -0.845 | 0.399 | 0.496 | 7-15years |
| ODI.BCC.combat | Control (Ref) vs 22q11.2Deletion | -0.147 | 0.171 | -0.859 | 0.391 | 0.493 | 7-15years |
| ODI.CC.combat | Control (Ref) vs 22q11.2Deletion | -0.116 | 0.171 | -0.679 | 0.497 | 0.595 | 7-15years |
| ODI.CGC.combat | Control (Ref) vs 22q11.2Deletion | 0.107 | 0.170 | 0.632 | 0.528 | 0.619 | 7-15years |
| ODI.CGH.combat | Control (Ref) vs 22q11.2Deletion | 0.380 | 0.165 | 2.305 | 0.022 | 0.044 | 7-15years |
| ODI.CR.combat | Control (Ref) vs 22q11.2Deletion | 0.077 | 0.172 | 0.446 | 0.656 | 0.743 | 7-15years |
| ODI.CST.combat | Control (Ref) vs 22q11.2Deletion | -0.297 | 0.165 | -1.798 | 0.073 | 0.126 | 7-15years |
| ODI.EC.combat | Control (Ref) vs 22q11.2Deletion | 0.542 | 0.169 | 3.216 | 0.001 | 0.004 | 7-15years |
| ODI.FX.combat | Control (Ref) vs 22q11.2Deletion | -0.167 | 0.172 | -0.969 | 0.333 | 0.440 | 7-15years |
| ODI.FXST.combat | Control (Ref) vs 22q11.2Deletion | 0.473 | 0.160 | 2.950 | 0.003 | 0.009 | 7-15years |
| ODI.GCC.combat | Control (Ref) vs 22q11.2Deletion | 0.136 | 0.170 | 0.800 | 0.424 | 0.524 | 7-15years |
| ODI.IC.combat | Control (Ref) vs 22q11.2Deletion | -0.256 | 0.169 | -1.522 | 0.129 | 0.201 | 7-15years |
| ODI.UNC.combat | Control (Ref) vs 22q11.2Deletion | -0.222 | 0.156 | -1.427 | 0.154 | 0.235 | 7-15years |
| ODI.PCR.combat | Control (Ref) vs 22q11.2Deletion | 0.165 | 0.172 | 0.962 | 0.337 | 0.443 | 7-15years |
| ODI.PLIC.combat | Control (Ref) vs 22q11.2Deletion | -0.446 | 0.167 | -2.679 | 0.008 | 0.018 | 7-15years |
| ODI.PTR.combat | Control (Ref) vs 22q11.2Deletion | 0.532 | 0.165 | 3.220 | 0.001 | 0.004 | 7-15years |
| ODI.RLIC.combat | Control (Ref) vs 22q11.2Deletion | -0.011 | 0.170 | -0.066 | 0.947 | 0.967 | 7-15years |
| ODI.SCC.combat | Control (Ref) vs 22q11.2Deletion | -0.261 | 0.170 | -1.537 | 0.125 | 0.196 | 7-15years |
| ODI.SCR.combat | Control (Ref) vs 22q11.2Deletion | -0.339 | 0.174 | -1.949 | 0.052 | 0.094 | 7-15years |
| ODI.SFO.combat | Control (Ref) vs 22q11.2Deletion | 0.223 | 0.169 | 1.320 | 0.187 | 0.274 | 7-15years |
| ODI.SLF.combat | Control (Ref) vs 22q11.2Deletion | 0.775 | 0.167 | 4.629 | 0.000 | 0.000 | 7-15years |
| ODI.SS.combat | Control (Ref) vs 22q11.2Deletion | 0.450 | 0.166 | 2.712 | 0.007 | 0.017 | 7-15years |
| ODI.TAP.combat | Control (Ref) vs 22q11.2Deletion | -0.096 | 0.174 | -0.554 | 0.580 | 0.672 | 7-15years |
| ICVF.combat | Control (Ref) vs 22q11.2Duplication | -0.926 | 0.169 | -5.487 | 0.000 | 0.000 | 7-15years |
| ISO.combat | Control (Ref) vs 22q11.2Duplication | -0.254 | 0.160 | -1.593 | 0.112 | 0.179 | 7-15years |
| ODI.combat | Control (Ref) vs 22q11.2Duplication | 0.726 | 0.189 | 3.837 | 0.000 | 0.001 | 7-15years |
| ICVF.ACR.combat | Control (Ref) vs 22q11.2Duplication | -0.725 | 0.191 | -3.796 | 0.000 | 0.001 | 7-15years |
| ICVF.ALIC.combat | Control (Ref) vs 22q11.2Duplication | -0.448 | 0.168 | -2.667 | 0.008 | 0.019 | 7-15years |
| ICVF.BCC.combat | Control (Ref) vs 22q11.2Duplication | -0.914 | 0.189 | -4.841 | 0.000 | 0.000 | 7-15years |
| ICVF.CC.combat | Control (Ref) vs 22q11.2Duplication | -0.930 | 0.191 | -4.880 | 0.000 | 0.000 | 7-15years |
| ICVF.CGC.combat | Control (Ref) vs 22q11.2Duplication | -0.941 | 0.174 | -5.393 | 0.000 | 0.000 | 7-15years |
| ICVF.CGH.combat | Control (Ref) vs 22q11.2Duplication | -0.465 | 0.181 | -2.567 | 0.011 | 0.024 | 7-15years |
| ICVF.CR.combat | Control (Ref) vs 22q11.2Duplication | -0.875 | 0.185 | -4.730 | 0.000 | 0.000 | 7-15years |
| ICVF.CST.combat | Control (Ref) vs 22q11.2Duplication | -0.624 | 0.178 | -3.496 | 0.001 | 0.002 | 7-15years |
| ICVF.EC.combat | Control (Ref) vs 22q11.2Duplication | -0.686 | 0.174 | -3.934 | 0.000 | 0.000 | 7-15years |
| ICVF.FX.combat | Control (Ref) vs 22q11.2Duplication | -0.566 | 0.193 | -2.926 | 0.004 | 0.010 | 7-15years |
| ICVF.FXST.combat | Control (Ref) vs 22q11.2Duplication | -0.445 | 0.188 | -2.370 | 0.018 | 0.038 | 7-15years |
| ICVF.GCC.combat | Control (Ref) vs 22q11.2Duplication | -0.634 | 0.189 | -3.366 | 0.001 | 0.003 | 7-15years |
| ICVF.IC.combat | Control (Ref) vs 22q11.2Duplication | -0.704 | 0.179 | -3.937 | 0.000 | 0.000 | 7-15years |
| ICVF.UNC.combat | Control (Ref) vs 22q11.2Duplication | -0.587 | 0.182 | -3.233 | 0.001 | 0.004 | 7-15years |
| ICVF.PCR.combat | Control (Ref) vs 22q11.2Duplication | -0.896 | 0.182 | -4.923 | 0.000 | 0.000 | 7-15years |
| ICVF.PLIC.combat | Control (Ref) vs 22q11.2Duplication | -0.659 | 0.181 | -3.647 | 0.000 | 0.001 | 7-15years |
| ICVF.PTR.combat | Control (Ref) vs 22q11.2Duplication | -0.880 | 0.183 | -4.822 | 0.000 | 0.000 | 7-15years |
| ICVF.RLIC.combat | Control (Ref) vs 22q11.2Duplication | -0.842 | 0.186 | -4.533 | 0.000 | 0.000 | 7-15years |
| ICVF.SCC.combat | Control (Ref) vs 22q11.2Duplication | -0.992 | 0.187 | -5.302 | 0.000 | 0.000 | 7-15years |
| ICVF.SCR.combat | Control (Ref) vs 22q11.2Duplication | -0.953 | 0.179 | -5.308 | 0.000 | 0.000 | 7-15years |
| ICVF.SFO.combat | Control (Ref) vs 22q11.2Duplication | -0.464 | 0.182 | -2.549 | 0.011 | 0.025 | 7-15years |
| ICVF.SLF.combat | Control (Ref) vs 22q11.2Duplication | -1.177 | 0.174 | -6.753 | 0.000 | 0.000 | 7-15years |
| ICVF.SS.combat | Control (Ref) vs 22q11.2Duplication | -0.659 | 0.189 | -3.479 | 0.001 | 0.002 | 7-15years |
| ICVF.TAP.combat | Control (Ref) vs 22q11.2Duplication | -0.531 | 0.194 | -2.731 | 0.007 | 0.017 | 7-15years |
| ISO.ACR.combat | Control (Ref) vs 22q11.2Duplication | -0.035 | 0.166 | -0.211 | 0.833 | 0.883 | 7-15years |
| ISO.ALIC.combat | Control (Ref) vs 22q11.2Duplication | 0.266 | 0.175 | 1.514 | 0.131 | 0.203 | 7-15years |
| ISO.BCC.combat | Control (Ref) vs 22q11.2Duplication | 0.033 | 0.173 | 0.191 | 0.849 | 0.896 | 7-15years |
| ISO.CC.combat | Control (Ref) vs 22q11.2Duplication | 0.154 | 0.167 | 0.922 | 0.357 | 0.466 | 7-15years |
| ISO.CGC.combat | Control (Ref) vs 22q11.2Duplication | -0.125 | 0.175 | -0.712 | 0.477 | 0.577 | 7-15years |
| ISO.CGH.combat | Control (Ref) vs 22q11.2Duplication | 0.170 | 0.193 | 0.880 | 0.379 | 0.483 | 7-15years |
| ISO.CR.combat | Control (Ref) vs 22q11.2Duplication | -0.183 | 0.166 | -1.107 | 0.269 | 0.366 | 7-15years |
| ISO.CST.combat | Control (Ref) vs 22q11.2Duplication | 0.252 | 0.168 | 1.500 | 0.134 | 0.208 | 7-15years |
| ISO.EC.combat | Control (Ref) vs 22q11.2Duplication | 0.296 | 0.163 | 1.816 | 0.070 | 0.122 | 7-15years |
| ISO.FX.combat | Control (Ref) vs 22q11.2Duplication | 0.500 | 0.200 | 2.502 | 0.013 | 0.028 | 7-15years |
| ISO.FXST.combat | Control (Ref) vs 22q11.2Duplication | -0.595 | 0.167 | -3.557 | 0.000 | 0.002 | 7-15years |
| ISO.GCC.combat | Control (Ref) vs 22q11.2Duplication | 0.019 | 0.170 | 0.113 | 0.910 | 0.942 | 7-15years |
| ISO.IC.combat | Control (Ref) vs 22q11.2Duplication | -0.002 | 0.168 | -0.014 | 0.989 | 0.992 | 7-15years |
| ISO.UNC.combat | Control (Ref) vs 22q11.2Duplication | -0.217 | 0.176 | -1.231 | 0.219 | 0.312 | 7-15years |
| ISO.PCR.combat | Control (Ref) vs 22q11.2Duplication | -0.046 | 0.171 | -0.271 | 0.787 | 0.843 | 7-15years |
| ISO.PLIC.combat | Control (Ref) vs 22q11.2Duplication | -0.183 | 0.175 | -1.050 | 0.294 | 0.394 | 7-15years |
| ISO.PTR.combat | Control (Ref) vs 22q11.2Duplication | -0.220 | 0.169 | -1.300 | 0.194 | 0.283 | 7-15years |
| ISO.RLIC.combat | Control (Ref) vs 22q11.2Duplication | -0.067 | 0.179 | -0.372 | 0.710 | 0.783 | 7-15years |
| ISO.SCC.combat | Control (Ref) vs 22q11.2Duplication | 0.420 | 0.175 | 2.402 | 0.017 | 0.036 | 7-15years |
| ISO.SCR.combat | Control (Ref) vs 22q11.2Duplication | -0.446 | 0.166 | -2.687 | 0.007 | 0.018 | 7-15years |
| ISO.SFO.combat | Control (Ref) vs 22q11.2Duplication | -0.180 | 0.168 | -1.071 | 0.285 | 0.385 | 7-15years |
| ISO.SLF.combat | Control (Ref) vs 22q11.2Duplication | -0.417 | 0.181 | -2.309 | 0.021 | 0.044 | 7-15years |
| ISO.SS.combat | Control (Ref) vs 22q11.2Duplication | -0.214 | 0.185 | -1.159 | 0.247 | 0.346 | 7-15years |
| ISO.TAP.combat | Control (Ref) vs 22q11.2Duplication | -0.024 | 0.196 | -0.124 | 0.901 | 0.936 | 7-15years |
| ODI.ACR.combat | Control (Ref) vs 22q11.2Duplication | 0.397 | 0.196 | 2.027 | 0.043 | 0.081 | 7-15years |
| ODI.ALIC.combat | Control (Ref) vs 22q11.2Duplication | 0.866 | 0.190 | 4.546 | 0.000 | 0.000 | 7-15years |
| ODI.BCC.combat | Control (Ref) vs 22q11.2Duplication | 0.586 | 0.194 | 3.017 | 0.003 | 0.008 | 7-15years |
| ODI.CC.combat | Control (Ref) vs 22q11.2Duplication | 0.318 | 0.196 | 1.623 | 0.105 | 0.171 | 7-15years |
| ODI.CGC.combat | Control (Ref) vs 22q11.2Duplication | 0.554 | 0.192 | 2.891 | 0.004 | 0.011 | 7-15years |
| ODI.CGH.combat | Control (Ref) vs 22q11.2Duplication | -0.167 | 0.191 | -0.876 | 0.382 | 0.483 | 7-15years |
| ODI.CR.combat | Control (Ref) vs 22q11.2Duplication | 0.120 | 0.200 | 0.598 | 0.550 | 0.642 | 7-15years |
| ODI.CST.combat | Control (Ref) vs 22q11.2Duplication | 0.482 | 0.192 | 2.506 | 0.013 | 0.028 | 7-15years |
| ODI.EC.combat | Control (Ref) vs 22q11.2Duplication | 0.521 | 0.198 | 2.626 | 0.009 | 0.021 | 7-15years |
| ODI.FX.combat | Control (Ref) vs 22q11.2Duplication | 0.306 | 0.199 | 1.540 | 0.124 | 0.196 | 7-15years |
| ODI.FXST.combat | Control (Ref) vs 22q11.2Duplication | -0.213 | 0.190 | -1.120 | 0.263 | 0.362 | 7-15years |
| ODI.GCC.combat | Control (Ref) vs 22q11.2Duplication | 0.277 | 0.194 | 1.428 | 0.154 | 0.235 | 7-15years |
| ODI.IC.combat | Control (Ref) vs 22q11.2Duplication | 0.587 | 0.194 | 3.022 | 0.003 | 0.008 | 7-15years |
| ODI.UNC.combat | Control (Ref) vs 22q11.2Duplication | 0.540 | 0.201 | 2.694 | 0.007 | 0.018 | 7-15years |
| ODI.PCR.combat | Control (Ref) vs 22q11.2Duplication | -0.090 | 0.203 | -0.444 | 0.657 | 0.743 | 7-15years |
| ODI.PLIC.combat | Control (Ref) vs 22q11.2Duplication | 0.642 | 0.194 | 3.318 | 0.001 | 0.003 | 7-15years |
| ODI.PTR.combat | Control (Ref) vs 22q11.2Duplication | 0.099 | 0.198 | 0.501 | 0.617 | 0.709 | 7-15years |
| ODI.RLIC.combat | Control (Ref) vs 22q11.2Duplication | -0.143 | 0.199 | -0.719 | 0.472 | 0.574 | 7-15years |
| ODI.SCC.combat | Control (Ref) vs 22q11.2Duplication | -0.098 | 0.200 | -0.489 | 0.625 | 0.716 | 7-15years |
| ODI.SCR.combat | Control (Ref) vs 22q11.2Duplication | -0.184 | 0.204 | -0.901 | 0.368 | 0.474 | 7-15years |
| ODI.SFO.combat | Control (Ref) vs 22q11.2Duplication | 0.244 | 0.198 | 1.231 | 0.219 | 0.312 | 7-15years |
| ODI.SLF.combat | Control (Ref) vs 22q11.2Duplication | 0.266 | 0.201 | 1.325 | 0.186 | 0.273 | 7-15years |
| ODI.SS.combat | Control (Ref) vs 22q11.2Duplication | -0.224 | 0.198 | -1.134 | 0.257 | 0.358 | 7-15years |
| ODI.TAP.combat | Control (Ref) vs 22q11.2Duplication | 0.067 | 0.205 | 0.325 | 0.746 | 0.808 | 7-15years |
| ICVF.combat | 22q11.2Deletion (Ref) vs 22q11.2Duplication | -0.968 | 0.183 | -5.285 | 0.000 | 0.000 | 7-15years |
| ISO.combat | 22q11.2Deletion (Ref) vs 22q11.2Duplication | -0.414 | 0.248 | -1.669 | 0.104 | 0.170 | 7-15years |
| ODI.combat | 22q11.2Deletion (Ref) vs 22q11.2Duplication | 0.451 | 0.247 | 1.828 | 0.076 | 0.131 | 7-15years |
| ICVF.ACR.combat | 22q11.2Deletion (Ref) vs 22q11.2Duplication | -0.987 | 0.191 | -5.157 | 0.000 | 0.000 | 7-15years |
| ICVF.ALIC.combat | 22q11.2Deletion (Ref) vs 22q11.2Duplication | -0.911 | 0.180 | -5.054 | 0.000 | 0.000 | 7-15years |
| ICVF.BCC.combat | 22q11.2Deletion (Ref) vs 22q11.2Duplication | -0.997 | 0.200 | -4.995 | 0.000 | 0.000 | 7-15years |
| ICVF.CC.combat | 22q11.2Deletion (Ref) vs 22q11.2Duplication | -1.003 | 0.201 | -5.002 | 0.000 | 0.000 | 7-15years |
| ICVF.CGC.combat | 22q11.2Deletion (Ref) vs 22q11.2Duplication | -0.994 | 0.195 | -5.088 | 0.000 | 0.000 | 7-15years |
| ICVF.CGH.combat | 22q11.2Deletion (Ref) vs 22q11.2Duplication | -0.673 | 0.227 | -2.959 | 0.006 | 0.014 | 7-15years |
| ICVF.CR.combat | 22q11.2Deletion (Ref) vs 22q11.2Duplication | -0.992 | 0.187 | -5.308 | 0.000 | 0.000 | 7-15years |
| ICVF.CST.combat | 22q11.2Deletion (Ref) vs 22q11.2Duplication | -0.790 | 0.198 | -3.996 | 0.000 | 0.001 | 7-15years |
| ICVF.EC.combat | 22q11.2Deletion (Ref) vs 22q11.2Duplication | -0.890 | 0.164 | -5.412 | 0.000 | 0.000 | 7-15years |
| ICVF.FX.combat | 22q11.2Deletion (Ref) vs 22q11.2Duplication | -0.560 | 0.231 | -2.420 | 0.021 | 0.043 | 7-15years |
| ICVF.FXST.combat | 22q11.2Deletion (Ref) vs 22q11.2Duplication | -0.916 | 0.217 | -4.215 | 0.000 | 0.001 | 7-15years |
| ICVF.GCC.combat | 22q11.2Deletion (Ref) vs 22q11.2Duplication | -0.965 | 0.199 | -4.837 | 0.000 | 0.000 | 7-15years |
| ICVF.IC.combat | 22q11.2Deletion (Ref) vs 22q11.2Duplication | -0.954 | 0.172 | -5.549 | 0.000 | 0.000 | 7-15years |
| ICVF.UNC.combat | 22q11.2Deletion (Ref) vs 22q11.2Duplication | -0.746 | 0.196 | -3.810 | 0.001 | 0.002 | 7-15years |
| ICVF.PCR.combat | 22q11.2Deletion (Ref) vs 22q11.2Duplication | -0.992 | 0.186 | -5.331 | 0.000 | 0.000 | 7-15years |
| ICVF.PLIC.combat | 22q11.2Deletion (Ref) vs 22q11.2Duplication | -0.885 | 0.172 | -5.150 | 0.000 | 0.000 | 7-15years |
| ICVF.PTR.combat | 22q11.2Deletion (Ref) vs 22q11.2Duplication | -0.947 | 0.186 | -5.100 | 0.000 | 0.000 | 7-15years |
| ICVF.RLIC.combat | 22q11.2Deletion (Ref) vs 22q11.2Duplication | -1.005 | 0.168 | -5.973 | 0.000 | 0.000 | 7-15years |
| ICVF.SCC.combat | 22q11.2Deletion (Ref) vs 22q11.2Duplication | -0.910 | 0.210 | -4.334 | 0.000 | 0.001 | 7-15years |
| ICVF.SCR.combat | 22q11.2Deletion (Ref) vs 22q11.2Duplication | -0.964 | 0.188 | -5.137 | 0.000 | 0.000 | 7-15years |
| ICVF.SFO.combat | 22q11.2Deletion (Ref) vs 22q11.2Duplication | -0.811 | 0.227 | -3.570 | 0.001 | 0.004 | 7-15years |
| ICVF.SLF.combat | 22q11.2Deletion (Ref) vs 22q11.2Duplication | -1.015 | 0.174 | -5.822 | 0.000 | 0.000 | 7-15years |
| ICVF.SS.combat | 22q11.2Deletion (Ref) vs 22q11.2Duplication | -0.963 | 0.181 | -5.330 | 0.000 | 0.000 | 7-15years |
| ICVF.TAP.combat | 22q11.2Deletion (Ref) vs 22q11.2Duplication | -0.839 | 0.215 | -3.904 | 0.000 | 0.002 | 7-15years |
| ISO.ACR.combat | 22q11.2Deletion (Ref) vs 22q11.2Duplication | -0.490 | 0.241 | -2.033 | 0.050 | 0.092 | 7-15years |
| ISO.ALIC.combat | 22q11.2Deletion (Ref) vs 22q11.2Duplication | 0.056 | 0.261 | 0.215 | 0.831 | 0.883 | 7-15years |
| ISO.BCC.combat | 22q11.2Deletion (Ref) vs 22q11.2Duplication | -0.014 | 0.263 | -0.054 | 0.957 | 0.974 | 7-15years |
| ISO.CC.combat | 22q11.2Deletion (Ref) vs 22q11.2Duplication | 0.216 | 0.269 | 0.803 | 0.428 | 0.526 | 7-15years |
| ISO.CGC.combat | 22q11.2Deletion (Ref) vs 22q11.2Duplication | -0.207 | 0.256 | -0.808 | 0.424 | 0.524 | 7-15years |
| ISO.CGH.combat | 22q11.2Deletion (Ref) vs 22q11.2Duplication | -0.092 | 0.252 | -0.366 | 0.717 | 0.785 | 7-15years |
| ISO.CR.combat | 22q11.2Deletion (Ref) vs 22q11.2Duplication | -0.524 | 0.242 | -2.164 | 0.038 | 0.070 | 7-15years |
| ISO.CST.combat | 22q11.2Deletion (Ref) vs 22q11.2Duplication | 0.439 | 0.233 | 1.887 | 0.068 | 0.119 | 7-15years |
| ISO.EC.combat | 22q11.2Deletion (Ref) vs 22q11.2Duplication | 0.282 | 0.249 | 1.133 | 0.265 | 0.363 | 7-15years |
| ISO.FX.combat | 22q11.2Deletion (Ref) vs 22q11.2Duplication | 0.022 | 0.273 | 0.081 | 0.936 | 0.961 | 7-15years |
| ISO.FXST.combat | 22q11.2Deletion (Ref) vs 22q11.2Duplication | -0.263 | 0.251 | -1.050 | 0.301 | 0.402 | 7-15years |
| ISO.GCC.combat | 22q11.2Deletion (Ref) vs 22q11.2Duplication | -0.112 | 0.251 | -0.446 | 0.658 | 0.743 | 7-15years |
| ISO.IC.combat | 22q11.2Deletion (Ref) vs 22q11.2Duplication | -0.083 | 0.248 | -0.336 | 0.739 | 0.804 | 7-15years |
| ISO.UNC.combat | 22q11.2Deletion (Ref) vs 22q11.2Duplication | -0.092 | 0.255 | -0.361 | 0.721 | 0.786 | 7-15years |
| ISO.PCR.combat | 22q11.2Deletion (Ref) vs 22q11.2Duplication | -0.454 | 0.258 | -1.761 | 0.087 | 0.148 | 7-15years |
| ISO.PLIC.combat | 22q11.2Deletion (Ref) vs 22q11.2Duplication | -0.010 | 0.245 | -0.039 | 0.969 | 0.982 | 7-15years |
| ISO.PTR.combat | 22q11.2Deletion (Ref) vs 22q11.2Duplication | -0.369 | 0.266 | -1.385 | 0.175 | 0.260 | 7-15years |
| ISO.RLIC.combat | 22q11.2Deletion (Ref) vs 22q11.2Duplication | -0.321 | 0.249 | -1.287 | 0.207 | 0.298 | 7-15years |
| ISO.SCC.combat | 22q11.2Deletion (Ref) vs 22q11.2Duplication | 0.530 | 0.267 | 1.988 | 0.055 | 0.099 | 7-15years |
| ISO.SCR.combat | 22q11.2Deletion (Ref) vs 22q11.2Duplication | -0.559 | 0.233 | -2.402 | 0.022 | 0.044 | 7-15years |
| ISO.SFO.combat | 22q11.2Deletion (Ref) vs 22q11.2Duplication | -0.517 | 0.207 | -2.494 | 0.018 | 0.038 | 7-15years |
| ISO.SLF.combat | 22q11.2Deletion (Ref) vs 22q11.2Duplication | -0.565 | 0.243 | -2.324 | 0.026 | 0.052 | 7-15years |
| ISO.SS.combat | 22q11.2Deletion (Ref) vs 22q11.2Duplication | -0.584 | 0.231 | -2.527 | 0.016 | 0.035 | 7-15years |
| ISO.TAP.combat | 22q11.2Deletion (Ref) vs 22q11.2Duplication | -0.227 | 0.252 | -0.901 | 0.374 | 0.479 | 7-15years |
| ODI.ACR.combat | 22q11.2Deletion (Ref) vs 22q11.2Duplication | 0.052 | 0.273 | 0.189 | 0.851 | 0.896 | 7-15years |
| ODI.ALIC.combat | 22q11.2Deletion (Ref) vs 22q11.2Duplication | 0.653 | 0.213 | 3.065 | 0.004 | 0.011 | 7-15years |
| ODI.BCC.combat | 22q11.2Deletion (Ref) vs 22q11.2Duplication | 0.509 | 0.254 | 2.003 | 0.053 | 0.096 | 7-15years |
| ODI.CC.combat | 22q11.2Deletion (Ref) vs 22q11.2Duplication | 0.328 | 0.269 | 1.218 | 0.232 | 0.326 | 7-15years |
| ODI.CGC.combat | 22q11.2Deletion (Ref) vs 22q11.2Duplication | 0.325 | 0.249 | 1.304 | 0.201 | 0.291 | 7-15years |
| ODI.CGH.combat | 22q11.2Deletion (Ref) vs 22q11.2Duplication | -0.557 | 0.269 | -2.074 | 0.046 | 0.085 | 7-15years |
| ODI.CR.combat | 22q11.2Deletion (Ref) vs 22q11.2Duplication | 0.044 | 0.281 | 0.157 | 0.876 | 0.915 | 7-15years |
| ODI.CST.combat | 22q11.2Deletion (Ref) vs 22q11.2Duplication | 0.578 | 0.242 | 2.388 | 0.023 | 0.045 | 7-15years |
| ODI.EC.combat | 22q11.2Deletion (Ref) vs 22q11.2Duplication | 0.004 | 0.292 | 0.014 | 0.989 | 0.992 | 7-15years |
| ODI.FX.combat | 22q11.2Deletion (Ref) vs 22q11.2Duplication | 0.595 | 0.260 | 2.292 | 0.028 | 0.054 | 7-15years |
| ODI.FXST.combat | 22q11.2Deletion (Ref) vs 22q11.2Duplication | -0.615 | 0.239 | -2.571 | 0.015 | 0.032 | 7-15years |
| ODI.GCC.combat | 22q11.2Deletion (Ref) vs 22q11.2Duplication | 0.117 | 0.265 | 0.441 | 0.662 | 0.744 | 7-15years |
| ODI.IC.combat | 22q11.2Deletion (Ref) vs 22q11.2Duplication | 0.617 | 0.226 | 2.733 | 0.010 | 0.023 | 7-15years |
| ODI.UNC.combat | 22q11.2Deletion (Ref) vs 22q11.2Duplication | 0.652 | 0.234 | 2.784 | 0.009 | 0.021 | 7-15years |
| ODI.PCR.combat | 22q11.2Deletion (Ref) vs 22q11.2Duplication | -0.188 | 0.285 | -0.661 | 0.513 | 0.606 | 7-15years |
| ODI.PLIC.combat | 22q11.2Deletion (Ref) vs 22q11.2Duplication | 0.790 | 0.216 | 3.664 | 0.001 | 0.003 | 7-15years |
| ODI.PTR.combat | 22q11.2Deletion (Ref) vs 22q11.2Duplication | -0.420 | 0.251 | -1.676 | 0.103 | 0.169 | 7-15years |
| ODI.RLIC.combat | 22q11.2Deletion (Ref) vs 22q11.2Duplication | -0.200 | 0.287 | -0.699 | 0.489 | 0.587 | 7-15years |
| ODI.SCC.combat | 22q11.2Deletion (Ref) vs 22q11.2Duplication | 0.121 | 0.283 | 0.426 | 0.673 | 0.750 | 7-15years |
| ODI.SCR.combat | 22q11.2Deletion (Ref) vs 22q11.2Duplication | 0.166 | 0.289 | 0.574 | 0.570 | 0.663 | 7-15years |
| ODI.SFO.combat | 22q11.2Deletion (Ref) vs 22q11.2Duplication | -0.022 | 0.280 | -0.078 | 0.939 | 0.961 | 7-15years |
| ODI.SLF.combat | 22q11.2Deletion (Ref) vs 22q11.2Duplication | -0.446 | 0.264 | -1.692 | 0.100 | 0.165 | 7-15years |
| ODI.SS.combat | 22q11.2Deletion (Ref) vs 22q11.2Duplication | -0.714 | 0.248 | -2.874 | 0.007 | 0.017 | 7-15years |
| ODI.TAP.combat | 22q11.2Deletion (Ref) vs 22q11.2Duplication | 0.243 | 0.267 | 0.912 | 0.368 | 0.474 | 7-15years |
| ICVF.combat | GeneDosage (1 = del, 2 = con, 3 dup) | -0.988 | 0.149 | -6.611 | 0.000 | 0.000 | 7-15years |
| ISO.combat | GeneDosage (1 = del, 2 = con, 3 dup) | -0.409 | 0.146 | -2.808 | 0.005 | 0.013 | 7-15years |
| ODI.combat | GeneDosage (1 = del, 2 = con, 3 dup) | 0.157 | 0.172 | 0.912 | 0.362 | 0.470 | 7-15years |
| ICVF.ACR.combat | GeneDosage (1 = del, 2 = con, 3 dup) | -1.097 | 0.166 | -6.621 | 0.000 | 0.000 | 7-15years |
| ICVF.ALIC.combat | GeneDosage (1 = del, 2 = con, 3 dup) | -0.815 | 0.149 | -5.468 | 0.000 | 0.000 | 7-15years |
| ICVF.BCC.combat | GeneDosage (1 = del, 2 = con, 3 dup) | -1.111 | 0.168 | -6.618 | 0.000 | 0.000 | 7-15years |
| ICVF.CC.combat | GeneDosage (1 = del, 2 = con, 3 dup) | -1.123 | 0.169 | -6.639 | 0.000 | 0.000 | 7-15years |
| ICVF.CGC.combat | GeneDosage (1 = del, 2 = con, 3 dup) | -0.892 | 0.160 | -5.579 | 0.000 | 0.000 | 7-15years |
| ICVF.CGH.combat | GeneDosage (1 = del, 2 = con, 3 dup) | -0.379 | 0.164 | -2.320 | 0.021 | 0.043 | 7-15years |
| ICVF.CR.combat | GeneDosage (1 = del, 2 = con, 3 dup) | -1.134 | 0.162 | -6.988 | 0.000 | 0.000 | 7-15years |
| ICVF.CST.combat | GeneDosage (1 = del, 2 = con, 3 dup) | -0.557 | 0.155 | -3.587 | 0.000 | 0.001 | 7-15years |
| ICVF.EC.combat | GeneDosage (1 = del, 2 = con, 3 dup) | -0.672 | 0.155 | -4.327 | 0.000 | 0.000 | 7-15years |
| ICVF.FX.combat | GeneDosage (1 = del, 2 = con, 3 dup) | -0.484 | 0.172 | -2.811 | 0.005 | 0.013 | 7-15years |
| ICVF.FXST.combat | GeneDosage (1 = del, 2 = con, 3 dup) | -0.683 | 0.168 | -4.074 | 0.000 | 0.000 | 7-15years |
| ICVF.GCC.combat | GeneDosage (1 = del, 2 = con, 3 dup) | -0.999 | 0.169 | -5.909 | 0.000 | 0.000 | 7-15years |
| ICVF.IC.combat | GeneDosage (1 = del, 2 = con, 3 dup) | -0.973 | 0.157 | -6.193 | 0.000 | 0.000 | 7-15years |
| ICVF.UNC.combat | GeneDosage (1 = del, 2 = con, 3 dup) | -0.854 | 0.143 | -5.991 | 0.000 | 0.000 | 7-15years |
| ICVF.PCR.combat | GeneDosage (1 = del, 2 = con, 3 dup) | -1.129 | 0.161 | -7.020 | 0.000 | 0.000 | 7-15years |
| ICVF.PLIC.combat | GeneDosage (1 = del, 2 = con, 3 dup) | -0.915 | 0.159 | -5.773 | 0.000 | 0.000 | 7-15years |
| ICVF.PTR.combat | GeneDosage (1 = del, 2 = con, 3 dup) | -1.049 | 0.160 | -6.548 | 0.000 | 0.000 | 7-15years |
| ICVF.RLIC.combat | GeneDosage (1 = del, 2 = con, 3 dup) | -1.011 | 0.162 | -6.260 | 0.000 | 0.000 | 7-15years |
| ICVF.SCC.combat | GeneDosage (1 = del, 2 = con, 3 dup) | -1.009 | 0.168 | -6.001 | 0.000 | 0.000 | 7-15years |
| ICVF.SCR.combat | GeneDosage (1 = del, 2 = con, 3 dup) | -1.071 | 0.161 | -6.656 | 0.000 | 0.000 | 7-15years |
| ICVF.SFO.combat | GeneDosage (1 = del, 2 = con, 3 dup) | -0.720 | 0.162 | -4.439 | 0.000 | 0.000 | 7-15years |
| ICVF.SLF.combat | GeneDosage (1 = del, 2 = con, 3 dup) | -1.177 | 0.156 | -7.547 | 0.000 | 0.000 | 7-15years |
| ICVF.SS.combat | GeneDosage (1 = del, 2 = con, 3 dup) | -0.929 | 0.162 | -5.720 | 0.000 | 0.000 | 7-15years |
| ICVF.TAP.combat | GeneDosage (1 = del, 2 = con, 3 dup) | -0.915 | 0.171 | -5.350 | 0.000 | 0.000 | 7-15years |
| ISO.ACR.combat | GeneDosage (1 = del, 2 = con, 3 dup) | -0.422 | 0.150 | -2.813 | 0.005 | 0.013 | 7-15years |
| ISO.ALIC.combat | GeneDosage (1 = del, 2 = con, 3 dup) | 0.029 | 0.162 | 0.180 | 0.858 | 0.899 | 7-15years |
| ISO.BCC.combat | GeneDosage (1 = del, 2 = con, 3 dup) | -0.110 | 0.165 | -0.666 | 0.506 | 0.599 | 7-15years |
| ISO.CC.combat | GeneDosage (1 = del, 2 = con, 3 dup) | 0.039 | 0.159 | 0.243 | 0.808 | 0.863 | 7-15years |
| ISO.CGC.combat | GeneDosage (1 = del, 2 = con, 3 dup) | -0.190 | 0.154 | -1.239 | 0.216 | 0.310 | 7-15years |
| ISO.CGH.combat | GeneDosage (1 = del, 2 = con, 3 dup) | -0.116 | 0.166 | -0.697 | 0.486 | 0.586 | 7-15years |
| ISO.CR.combat | GeneDosage (1 = del, 2 = con, 3 dup) | -0.462 | 0.153 | -3.020 | 0.003 | 0.008 | 7-15years |
| ISO.CST.combat | GeneDosage (1 = del, 2 = con, 3 dup) | 0.321 | 0.152 | 2.113 | 0.035 | 0.067 | 7-15years |
| ISO.EC.combat | GeneDosage (1 = del, 2 = con, 3 dup) | 0.000 | 0.157 | 0.003 | 0.998 | 0.998 | 7-15years |
| ISO.FX.combat | GeneDosage (1 = del, 2 = con, 3 dup) | -0.152 | 0.178 | -0.852 | 0.394 | 0.493 | 7-15years |
| ISO.FXST.combat | GeneDosage (1 = del, 2 = con, 3 dup) | -0.275 | 0.160 | -1.722 | 0.086 | 0.146 | 7-15years |
| ISO.GCC.combat | GeneDosage (1 = del, 2 = con, 3 dup) | -0.189 | 0.155 | -1.217 | 0.224 | 0.317 | 7-15years |
| ISO.IC.combat | GeneDosage (1 = del, 2 = con, 3 dup) | -0.046 | 0.153 | -0.303 | 0.762 | 0.822 | 7-15years |
| ISO.UNC.combat | GeneDosage (1 = del, 2 = con, 3 dup) | -0.062 | 0.170 | -0.366 | 0.714 | 0.785 | 7-15years |
| ISO.PCR.combat | GeneDosage (1 = del, 2 = con, 3 dup) | -0.375 | 0.158 | -2.372 | 0.018 | 0.038 | 7-15years |
| ISO.PLIC.combat | GeneDosage (1 = del, 2 = con, 3 dup) | 0.073 | 0.157 | 0.465 | 0.642 | 0.733 | 7-15years |
| ISO.PTR.combat | GeneDosage (1 = del, 2 = con, 3 dup) | -0.300 | 0.161 | -1.868 | 0.062 | 0.111 | 7-15years |
| ISO.RLIC.combat | GeneDosage (1 = del, 2 = con, 3 dup) | -0.222 | 0.160 | -1.385 | 0.167 | 0.249 | 7-15years |
| ISO.SCC.combat | GeneDosage (1 = del, 2 = con, 3 dup) | 0.345 | 0.150 | 2.297 | 0.022 | 0.044 | 7-15years |
| ISO.SCR.combat | GeneDosage (1 = del, 2 = con, 3 dup) | -0.502 | 0.155 | -3.243 | 0.001 | 0.004 | 7-15years |
| ISO.SFO.combat | GeneDosage (1 = del, 2 = con, 3 dup) | -0.390 | 0.158 | -2.475 | 0.014 | 0.030 | 7-15years |
| ISO.SLF.combat | GeneDosage (1 = del, 2 = con, 3 dup) | -0.520 | 0.159 | -3.273 | 0.001 | 0.004 | 7-15years |
| ISO.SS.combat | GeneDosage (1 = del, 2 = con, 3 dup) | -0.317 | 0.163 | -1.951 | 0.052 | 0.094 | 7-15years |
| ISO.TAP.combat | GeneDosage (1 = del, 2 = con, 3 dup) | -0.295 | 0.174 | -1.700 | 0.090 | 0.151 | 7-15years |
| ODI.ACR.combat | GeneDosage (1 = del, 2 = con, 3 dup) | -0.019 | 0.180 | -0.104 | 0.917 | 0.945 | 7-15years |
| ODI.ALIC.combat | GeneDosage (1 = del, 2 = con, 3 dup) | 0.625 | 0.173 | 3.601 | 0.000 | 0.001 | 7-15years |
| ODI.BCC.combat | GeneDosage (1 = del, 2 = con, 3 dup) | 0.458 | 0.177 | 2.581 | 0.010 | 0.023 | 7-15years |
| ODI.CC.combat | GeneDosage (1 = del, 2 = con, 3 dup) | 0.277 | 0.179 | 1.548 | 0.122 | 0.194 | 7-15years |
| ODI.CGC.combat | GeneDosage (1 = del, 2 = con, 3 dup) | 0.238 | 0.178 | 1.333 | 0.183 | 0.271 | 7-15years |
| ODI.CGH.combat | GeneDosage (1 = del, 2 = con, 3 dup) | -0.418 | 0.175 | -2.396 | 0.017 | 0.036 | 7-15years |
| ODI.CR.combat | GeneDosage (1 = del, 2 = con, 3 dup) | 0.006 | 0.182 | 0.033 | 0.974 | 0.984 | 7-15years |
| ODI.CST.combat | GeneDosage (1 = del, 2 = con, 3 dup) | 0.526 | 0.175 | 3.007 | 0.003 | 0.008 | 7-15years |
| ODI.EC.combat | GeneDosage (1 = del, 2 = con, 3 dup) | -0.131 | 0.182 | -0.720 | 0.472 | 0.574 | 7-15years |
| ODI.FX.combat | GeneDosage (1 = del, 2 = con, 3 dup) | 0.323 | 0.181 | 1.788 | 0.074 | 0.128 | 7-15years |
| ODI.FXST.combat | GeneDosage (1 = del, 2 = con, 3 dup) | -0.498 | 0.172 | -2.893 | 0.004 | 0.011 | 7-15years |
| ODI.GCC.combat | GeneDosage (1 = del, 2 = con, 3 dup) | 0.052 | 0.178 | 0.295 | 0.768 | 0.826 | 7-15years |
| ODI.IC.combat | GeneDosage (1 = del, 2 = con, 3 dup) | 0.549 | 0.176 | 3.127 | 0.002 | 0.006 | 7-15years |
| ODI.UNC.combat | GeneDosage (1 = del, 2 = con, 3 dup) | 0.528 | 0.170 | 3.107 | 0.002 | 0.006 | 7-15years |
| ODI.PCR.combat | GeneDosage (1 = del, 2 = con, 3 dup) | -0.185 | 0.183 | -1.015 | 0.311 | 0.412 | 7-15years |
| ODI.PLIC.combat | GeneDosage (1 = del, 2 = con, 3 dup) | 0.732 | 0.173 | 4.229 | 0.000 | 0.000 | 7-15years |
| ODI.PTR.combat | GeneDosage (1 = del, 2 = con, 3 dup) | -0.375 | 0.177 | -2.114 | 0.035 | 0.067 | 7-15years |
| ODI.RLIC.combat | GeneDosage (1 = del, 2 = con, 3 dup) | -0.077 | 0.182 | -0.426 | 0.670 | 0.750 | 7-15years |
| ODI.SCC.combat | GeneDosage (1 = del, 2 = con, 3 dup) | 0.154 | 0.181 | 0.854 | 0.394 | 0.493 | 7-15years |
| ODI.SCR.combat | GeneDosage (1 = del, 2 = con, 3 dup) | 0.162 | 0.185 | 0.877 | 0.381 | 0.483 | 7-15years |
| ODI.SFO.combat | GeneDosage (1 = del, 2 = con, 3 dup) | -0.027 | 0.179 | -0.153 | 0.879 | 0.915 | 7-15years |
| ODI.SLF.combat | GeneDosage (1 = del, 2 = con, 3 dup) | -0.472 | 0.180 | -2.625 | 0.009 | 0.021 | 7-15years |
| ODI.SS.combat | GeneDosage (1 = del, 2 = con, 3 dup) | -0.499 | 0.177 | -2.818 | 0.005 | 0.013 | 7-15years |
| ODI.TAP.combat | GeneDosage (1 = del, 2 = con, 3 dup) | 0.118 | 0.185 | 0.640 | 0.523 | 0.615 | 7-15years |
| ICVF.combat | Control (Ref) vs 22q11.2Deletion | 0.812 | 0.164 | 4.941 | 0.000 | 0.000 | 15-23years |
| ISO.combat | Control (Ref) vs 22q11.2Deletion | 0.093 | 0.152 | 0.614 | 0.540 | 0.647 | 15-23years |
| ODI.combat | Control (Ref) vs 22q11.2Deletion | 0.502 | 0.164 | 3.055 | 0.003 | 0.008 | 15-23years |
| ICVF.ACR.combat | Control (Ref) vs 22q11.2Deletion | 0.949 | 0.165 | 5.751 | 0.000 | 0.000 | 15-23years |
| ICVF.ALIC.combat | Control (Ref) vs 22q11.2Deletion | 0.514 | 0.164 | 3.142 | 0.002 | 0.006 | 15-23years |
| ICVF.BCC.combat | Control (Ref) vs 22q11.2Deletion | 0.903 | 0.170 | 5.320 | 0.000 | 0.000 | 15-23years |
| ICVF.CC.combat | Control (Ref) vs 22q11.2Deletion | 0.872 | 0.169 | 5.158 | 0.000 | 0.000 | 15-23years |
| ICVF.CGC.combat | Control (Ref) vs 22q11.2Deletion | 0.480 | 0.171 | 2.800 | 0.006 | 0.016 | 15-23years |
| ICVF.CGH.combat | Control (Ref) vs 22q11.2Deletion | 0.193 | 0.168 | 1.149 | 0.252 | 0.379 | 15-23years |
| ICVF.CR.combat | Control (Ref) vs 22q11.2Deletion | 1.026 | 0.164 | 6.240 | 0.000 | 0.000 | 15-23years |
| ICVF.CST.combat | Control (Ref) vs 22q11.2Deletion | 0.277 | 0.146 | 1.895 | 0.059 | 0.120 | 15-23years |
| ICVF.EC.combat | Control (Ref) vs 22q11.2Deletion | 0.504 | 0.174 | 2.904 | 0.004 | 0.012 | 15-23years |
| ICVF.FX.combat | Control (Ref) vs 22q11.2Deletion | -0.031 | 0.178 | -0.176 | 0.861 | 0.900 | 15-23years |
| ICVF.FXST.combat | Control (Ref) vs 22q11.2Deletion | 0.540 | 0.173 | 3.120 | 0.002 | 0.007 | 15-23years |
| ICVF.GCC.combat | Control (Ref) vs 22q11.2Deletion | 0.790 | 0.171 | 4.612 | 0.000 | 0.000 | 15-23years |
| ICVF.IC.combat | Control (Ref) vs 22q11.2Deletion | 0.631 | 0.146 | 4.313 | 0.000 | 0.000 | 15-23years |
| ICVF.UNC.combat | Control (Ref) vs 22q11.2Deletion | 0.911 | 0.167 | 5.465 | 0.000 | 0.000 | 15-23years |
| ICVF.PCR.combat | Control (Ref) vs 22q11.2Deletion | 1.107 | 0.163 | 6.808 | 0.000 | 0.000 | 15-23years |
| ICVF.PLIC.combat | Control (Ref) vs 22q11.2Deletion | 0.551 | 0.151 | 3.643 | 0.000 | 0.001 | 15-23years |
| ICVF.PTR.combat | Control (Ref) vs 22q11.2Deletion | 1.045 | 0.169 | 6.174 | 0.000 | 0.000 | 15-23years |
| ICVF.RLIC.combat | Control (Ref) vs 22q11.2Deletion | 0.684 | 0.171 | 4.006 | 0.000 | 0.000 | 15-23years |
| ICVF.SCC.combat | Control (Ref) vs 22q11.2Deletion | 0.739 | 0.174 | 4.240 | 0.000 | 0.000 | 15-23years |
| ICVF.SCR.combat | Control (Ref) vs 22q11.2Deletion | 0.961 | 0.167 | 5.750 | 0.000 | 0.000 | 15-23years |
| ICVF.SFO.combat | Control (Ref) vs 22q11.2Deletion | 0.508 | 0.178 | 2.855 | 0.005 | 0.014 | 15-23years |
| ICVF.SLF.combat | Control (Ref) vs 22q11.2Deletion | 0.883 | 0.168 | 5.248 | 0.000 | 0.000 | 15-23years |
| ICVF.SS.combat | Control (Ref) vs 22q11.2Deletion | 0.836 | 0.171 | 4.892 | 0.000 | 0.000 | 15-23years |
| ICVF.TAP.combat | Control (Ref) vs 22q11.2Deletion | 0.812 | 0.173 | 4.700 | 0.000 | 0.000 | 15-23years |
| ISO.ACR.combat | Control (Ref) vs 22q11.2Deletion | 0.212 | 0.160 | 1.324 | 0.187 | 0.302 | 15-23years |
| ISO.ALIC.combat | Control (Ref) vs 22q11.2Deletion | 0.047 | 0.179 | 0.265 | 0.791 | 0.851 | 15-23years |
| ISO.BCC.combat | Control (Ref) vs 22q11.2Deletion | -0.138 | 0.180 | -0.765 | 0.445 | 0.573 | 15-23years |
| ISO.CC.combat | Control (Ref) vs 22q11.2Deletion | -0.130 | 0.181 | -0.717 | 0.474 | 0.595 | 15-23years |
| ISO.CGC.combat | Control (Ref) vs 22q11.2Deletion | 0.014 | 0.146 | 0.094 | 0.925 | 0.951 | 15-23years |
| ISO.CGH.combat | Control (Ref) vs 22q11.2Deletion | -0.087 | 0.152 | -0.575 | 0.566 | 0.671 | 15-23years |
| ISO.CR.combat | Control (Ref) vs 22q11.2Deletion | 0.191 | 0.159 | 1.196 | 0.233 | 0.358 | 15-23years |
| ISO.CST.combat | Control (Ref) vs 22q11.2Deletion | 0.220 | 0.183 | 1.203 | 0.230 | 0.358 | 15-23years |
| ISO.EC.combat | Control (Ref) vs 22q11.2Deletion | -0.208 | 0.186 | -1.117 | 0.265 | 0.392 | 15-23years |
| ISO.FX.combat | Control (Ref) vs 22q11.2Deletion | 0.495 | 0.184 | 2.686 | 0.008 | 0.021 | 15-23years |
| ISO.FXST.combat | Control (Ref) vs 22q11.2Deletion | -0.405 | 0.147 | -2.753 | 0.006 | 0.018 | 15-23years |
| ISO.GCC.combat | Control (Ref) vs 22q11.2Deletion | 0.024 | 0.180 | 0.132 | 0.895 | 0.926 | 15-23years |
| ISO.IC.combat | Control (Ref) vs 22q11.2Deletion | -0.151 | 0.169 | -0.891 | 0.374 | 0.503 | 15-23years |
| ISO.UNC.combat | Control (Ref) vs 22q11.2Deletion | 0.107 | 0.161 | 0.664 | 0.508 | 0.619 | 15-23years |
| ISO.PCR.combat | Control (Ref) vs 22q11.2Deletion | 0.285 | 0.157 | 1.813 | 0.071 | 0.137 | 15-23years |
| ISO.PLIC.combat | Control (Ref) vs 22q11.2Deletion | -0.162 | 0.169 | -0.959 | 0.338 | 0.472 | 15-23years |
| ISO.PTR.combat | Control (Ref) vs 22q11.2Deletion | 0.355 | 0.178 | 1.994 | 0.047 | 0.101 | 15-23years |
| ISO.RLIC.combat | Control (Ref) vs 22q11.2Deletion | -0.241 | 0.162 | -1.486 | 0.139 | 0.235 | 15-23years |
| ISO.SCC.combat | Control (Ref) vs 22q11.2Deletion | -0.248 | 0.183 | -1.356 | 0.176 | 0.288 | 15-23years |
| ISO.SCR.combat | Control (Ref) vs 22q11.2Deletion | 0.068 | 0.170 | 0.402 | 0.688 | 0.787 | 15-23years |
| ISO.SFO.combat | Control (Ref) vs 22q11.2Deletion | 0.020 | 0.177 | 0.115 | 0.908 | 0.936 | 15-23years |
| ISO.SLF.combat | Control (Ref) vs 22q11.2Deletion | 0.125 | 0.160 | 0.784 | 0.434 | 0.565 | 15-23years |
| ISO.SS.combat | Control (Ref) vs 22q11.2Deletion | -0.051 | 0.184 | -0.281 | 0.779 | 0.844 | 15-23years |
| ISO.TAP.combat | Control (Ref) vs 22q11.2Deletion | 0.297 | 0.183 | 1.625 | 0.106 | 0.193 | 15-23years |
| ODI.ACR.combat | Control (Ref) vs 22q11.2Deletion | 0.454 | 0.180 | 2.526 | 0.012 | 0.031 | 15-23years |
| ODI.ALIC.combat | Control (Ref) vs 22q11.2Deletion | -0.329 | 0.180 | -1.823 | 0.070 | 0.136 | 15-23years |
| ODI.BCC.combat | Control (Ref) vs 22q11.2Deletion | 0.224 | 0.185 | 1.214 | 0.226 | 0.353 | 15-23years |
| ODI.CC.combat | Control (Ref) vs 22q11.2Deletion | 0.299 | 0.186 | 1.608 | 0.109 | 0.198 | 15-23years |
| ODI.CGC.combat | Control (Ref) vs 22q11.2Deletion | -0.126 | 0.191 | -0.661 | 0.509 | 0.619 | 15-23years |
| ODI.CGH.combat | Control (Ref) vs 22q11.2Deletion | 0.482 | 0.175 | 2.751 | 0.006 | 0.018 | 15-23years |
| ODI.CR.combat | Control (Ref) vs 22q11.2Deletion | 0.348 | 0.181 | 1.917 | 0.056 | 0.116 | 15-23years |
| ODI.CST.combat | Control (Ref) vs 22q11.2Deletion | -0.070 | 0.176 | -0.399 | 0.690 | 0.787 | 15-23years |
| ODI.EC.combat | Control (Ref) vs 22q11.2Deletion | 0.725 | 0.166 | 4.358 | 0.000 | 0.000 | 15-23years |
| ODI.FX.combat | Control (Ref) vs 22q11.2Deletion | 0.294 | 0.189 | 1.556 | 0.121 | 0.214 | 15-23years |
| ODI.FXST.combat | Control (Ref) vs 22q11.2Deletion | 0.260 | 0.173 | 1.502 | 0.135 | 0.231 | 15-23years |
| ODI.GCC.combat | Control (Ref) vs 22q11.2Deletion | 0.522 | 0.183 | 2.853 | 0.005 | 0.014 | 15-23years |
| ODI.IC.combat | Control (Ref) vs 22q11.2Deletion | -0.362 | 0.182 | -1.984 | 0.048 | 0.103 | 15-23years |
| ODI.UNC.combat | Control (Ref) vs 22q11.2Deletion | -0.045 | 0.192 | -0.233 | 0.816 | 0.868 | 15-23years |
| ODI.PCR.combat | Control (Ref) vs 22q11.2Deletion | 0.196 | 0.182 | 1.077 | 0.283 | 0.410 | 15-23years |
| ODI.PLIC.combat | Control (Ref) vs 22q11.2Deletion | -0.500 | 0.187 | -2.674 | 0.008 | 0.022 | 15-23years |
| ODI.PTR.combat | Control (Ref) vs 22q11.2Deletion | 0.643 | 0.180 | 3.575 | 0.000 | 0.002 | 15-23years |
| ODI.RLIC.combat | Control (Ref) vs 22q11.2Deletion | -0.006 | 0.185 | -0.035 | 0.972 | 0.986 | 15-23years |
| ODI.SCC.combat | Control (Ref) vs 22q11.2Deletion | 0.052 | 0.190 | 0.275 | 0.783 | 0.845 | 15-23years |
| ODI.SCR.combat | Control (Ref) vs 22q11.2Deletion | 0.121 | 0.187 | 0.646 | 0.519 | 0.628 | 15-23years |
| ODI.SFO.combat | Control (Ref) vs 22q11.2Deletion | 0.525 | 0.179 | 2.940 | 0.004 | 0.011 | 15-23years |
| ODI.SLF.combat | Control (Ref) vs 22q11.2Deletion | 0.867 | 0.172 | 5.039 | 0.000 | 0.000 | 15-23years |
| ODI.SS.combat | Control (Ref) vs 22q11.2Deletion | 0.336 | 0.185 | 1.813 | 0.071 | 0.137 | 15-23years |
| ODI.TAP.combat | Control (Ref) vs 22q11.2Deletion | -0.258 | 0.188 | -1.371 | 0.172 | 0.283 | 15-23years |
| ICVF.combat | Control (Ref) vs 22q11.2Duplication | -1.373 | 0.274 | -5.006 | 0.000 | 0.000 | 15-23years |
| ISO.combat | Control (Ref) vs 22q11.2Duplication | -0.607 | 0.290 | -2.095 | 0.037 | 0.084 | 15-23years |
| ODI.combat | Control (Ref) vs 22q11.2Duplication | 0.470 | 0.307 | 1.531 | 0.127 | 0.222 | 15-23years |
| ICVF.ACR.combat | Control (Ref) vs 22q11.2Duplication | -1.302 | 0.284 | -4.592 | 0.000 | 0.000 | 15-23years |
| ICVF.ALIC.combat | Control (Ref) vs 22q11.2Duplication | -0.930 | 0.259 | -3.597 | 0.000 | 0.001 | 15-23years |
| ICVF.BCC.combat | Control (Ref) vs 22q11.2Duplication | -1.416 | 0.271 | -5.223 | 0.000 | 0.000 | 15-23years |
| ICVF.CC.combat | Control (Ref) vs 22q11.2Duplication | -1.400 | 0.273 | -5.133 | 0.000 | 0.000 | 15-23years |
| ICVF.CGC.combat | Control (Ref) vs 22q11.2Duplication | -0.932 | 0.257 | -3.618 | 0.000 | 0.001 | 15-23years |
| ICVF.CGH.combat | Control (Ref) vs 22q11.2Duplication | -0.955 | 0.284 | -3.357 | 0.001 | 0.003 | 15-23years |
| ICVF.CR.combat | Control (Ref) vs 22q11.2Duplication | -1.439 | 0.283 | -5.089 | 0.000 | 0.000 | 15-23years |
| ICVF.CST.combat | Control (Ref) vs 22q11.2Duplication | -0.343 | 0.268 | -1.280 | 0.202 | 0.322 | 15-23years |
| ICVF.EC.combat | Control (Ref) vs 22q11.2Duplication | -1.061 | 0.287 | -3.703 | 0.000 | 0.001 | 15-23years |
| ICVF.FX.combat | Control (Ref) vs 22q11.2Duplication | -0.606 | 0.272 | -2.224 | 0.027 | 0.063 | 15-23years |
| ICVF.FXST.combat | Control (Ref) vs 22q11.2Duplication | -1.062 | 0.297 | -3.575 | 0.000 | 0.002 | 15-23years |
| ICVF.GCC.combat | Control (Ref) vs 22q11.2Duplication | -1.168 | 0.283 | -4.131 | 0.000 | 0.000 | 15-23years |
| ICVF.IC.combat | Control (Ref) vs 22q11.2Duplication | -1.066 | 0.269 | -3.967 | 0.000 | 0.000 | 15-23years |
| ICVF.UNC.combat | Control (Ref) vs 22q11.2Duplication | -0.862 | 0.304 | -2.831 | 0.005 | 0.015 | 15-23years |
| ICVF.PCR.combat | Control (Ref) vs 22q11.2Duplication | -1.465 | 0.291 | -5.033 | 0.000 | 0.000 | 15-23years |
| ICVF.PLIC.combat | Control (Ref) vs 22q11.2Duplication | -0.943 | 0.275 | -3.429 | 0.001 | 0.003 | 15-23years |
| ICVF.PTR.combat | Control (Ref) vs 22q11.2Duplication | -1.494 | 0.292 | -5.121 | 0.000 | 0.000 | 15-23years |
| ICVF.RLIC.combat | Control (Ref) vs 22q11.2Duplication | -1.138 | 0.281 | -4.053 | 0.000 | 0.000 | 15-23years |
| ICVF.SCC.combat | Control (Ref) vs 22q11.2Duplication | -1.367 | 0.281 | -4.867 | 0.000 | 0.000 | 15-23years |
| ICVF.SCR.combat | Control (Ref) vs 22q11.2Duplication | -1.437 | 0.280 | -5.134 | 0.000 | 0.000 | 15-23years |
| ICVF.SFO.combat | Control (Ref) vs 22q11.2Duplication | -1.194 | 0.268 | -4.459 | 0.000 | 0.000 | 15-23years |
| ICVF.SLF.combat | Control (Ref) vs 22q11.2Duplication | -1.563 | 0.278 | -5.620 | 0.000 | 0.000 | 15-23years |
| ICVF.SS.combat | Control (Ref) vs 22q11.2Duplication | -1.295 | 0.296 | -4.379 | 0.000 | 0.000 | 15-23years |
| ICVF.TAP.combat | Control (Ref) vs 22q11.2Duplication | -1.301 | 0.299 | -4.355 | 0.000 | 0.000 | 15-23years |
| ISO.ACR.combat | Control (Ref) vs 22q11.2Duplication | -0.276 | 0.273 | -1.009 | 0.314 | 0.451 | 15-23years |
| ISO.ALIC.combat | Control (Ref) vs 22q11.2Duplication | -0.302 | 0.314 | -0.961 | 0.337 | 0.472 | 15-23years |
| ISO.BCC.combat | Control (Ref) vs 22q11.2Duplication | -0.212 | 0.312 | -0.678 | 0.499 | 0.615 | 15-23years |
| ISO.CC.combat | Control (Ref) vs 22q11.2Duplication | -0.218 | 0.314 | -0.694 | 0.488 | 0.608 | 15-23years |
| ISO.CGC.combat | Control (Ref) vs 22q11.2Duplication | -0.454 | 0.266 | -1.708 | 0.089 | 0.168 | 15-23years |
| ISO.CGH.combat | Control (Ref) vs 22q11.2Duplication | -0.066 | 0.267 | -0.249 | 0.804 | 0.858 | 15-23years |
| ISO.CR.combat | Control (Ref) vs 22q11.2Duplication | -0.471 | 0.294 | -1.601 | 0.111 | 0.200 | 15-23years |
| ISO.CST.combat | Control (Ref) vs 22q11.2Duplication | 0.151 | 0.291 | 0.519 | 0.605 | 0.706 | 15-23years |
| ISO.EC.combat | Control (Ref) vs 22q11.2Duplication | -0.291 | 0.313 | -0.928 | 0.354 | 0.488 | 15-23years |
| ISO.FX.combat | Control (Ref) vs 22q11.2Duplication | 0.658 | 0.317 | 2.076 | 0.039 | 0.087 | 15-23years |
| ISO.FXST.combat | Control (Ref) vs 22q11.2Duplication | -0.343 | 0.279 | -1.231 | 0.220 | 0.347 | 15-23years |
| ISO.GCC.combat | Control (Ref) vs 22q11.2Duplication | -0.044 | 0.307 | -0.142 | 0.887 | 0.921 | 15-23years |
| ISO.IC.combat | Control (Ref) vs 22q11.2Duplication | -0.488 | 0.311 | -1.571 | 0.118 | 0.210 | 15-23years |
| ISO.UNC.combat | Control (Ref) vs 22q11.2Duplication | -0.191 | 0.322 | -0.594 | 0.553 | 0.661 | 15-23years |
| ISO.PCR.combat | Control (Ref) vs 22q11.2Duplication | -0.777 | 0.304 | -2.555 | 0.011 | 0.030 | 15-23years |
| ISO.PLIC.combat | Control (Ref) vs 22q11.2Duplication | -0.377 | 0.314 | -1.200 | 0.231 | 0.358 | 15-23years |
| ISO.PTR.combat | Control (Ref) vs 22q11.2Duplication | -0.622 | 0.319 | -1.951 | 0.052 | 0.109 | 15-23years |
| ISO.RLIC.combat | Control (Ref) vs 22q11.2Duplication | -0.467 | 0.302 | -1.547 | 0.123 | 0.216 | 15-23years |
| ISO.SCC.combat | Control (Ref) vs 22q11.2Duplication | -0.314 | 0.316 | -0.994 | 0.321 | 0.457 | 15-23years |
| ISO.SCR.combat | Control (Ref) vs 22q11.2Duplication | -0.524 | 0.308 | -1.700 | 0.091 | 0.170 | 15-23years |
| ISO.SFO.combat | Control (Ref) vs 22q11.2Duplication | -0.668 | 0.263 | -2.545 | 0.012 | 0.030 | 15-23years |
| ISO.SLF.combat | Control (Ref) vs 22q11.2Duplication | -0.480 | 0.308 | -1.560 | 0.120 | 0.214 | 15-23years |
| ISO.SS.combat | Control (Ref) vs 22q11.2Duplication | -0.598 | 0.305 | -1.960 | 0.051 | 0.108 | 15-23years |
| ISO.TAP.combat | Control (Ref) vs 22q11.2Duplication | -0.514 | 0.316 | -1.626 | 0.105 | 0.193 | 15-23years |
| ODI.ACR.combat | Control (Ref) vs 22q11.2Duplication | 0.058 | 0.306 | 0.189 | 0.850 | 0.892 | 15-23years |
| ODI.ALIC.combat | Control (Ref) vs 22q11.2Duplication | 0.769 | 0.280 | 2.743 | 0.007 | 0.018 | 15-23years |
| ODI.BCC.combat | Control (Ref) vs 22q11.2Duplication | 0.490 | 0.280 | 1.752 | 0.081 | 0.155 | 15-23years |
| ODI.CC.combat | Control (Ref) vs 22q11.2Duplication | 0.113 | 0.298 | 0.379 | 0.705 | 0.800 | 15-23years |
| ODI.CGC.combat | Control (Ref) vs 22q11.2Duplication | 0.169 | 0.302 | 0.560 | 0.576 | 0.681 | 15-23years |
| ODI.CGH.combat | Control (Ref) vs 22q11.2Duplication | -0.214 | 0.308 | -0.694 | 0.488 | 0.608 | 15-23years |
| ODI.CR.combat | Control (Ref) vs 22q11.2Duplication | -0.148 | 0.305 | -0.484 | 0.629 | 0.728 | 15-23years |
| ODI.CST.combat | Control (Ref) vs 22q11.2Duplication | 0.390 | 0.300 | 1.299 | 0.195 | 0.314 | 15-23years |
| ODI.EC.combat | Control (Ref) vs 22q11.2Duplication | 0.594 | 0.301 | 1.977 | 0.049 | 0.104 | 15-23years |
| ODI.FX.combat | Control (Ref) vs 22q11.2Duplication | 0.085 | 0.271 | 0.313 | 0.755 | 0.837 | 15-23years |
| ODI.FXST.combat | Control (Ref) vs 22q11.2Duplication | 0.112 | 0.297 | 0.376 | 0.707 | 0.800 | 15-23years |
| ODI.GCC.combat | Control (Ref) vs 22q11.2Duplication | -0.002 | 0.302 | -0.007 | 0.995 | 0.995 | 15-23years |
| ODI.IC.combat | Control (Ref) vs 22q11.2Duplication | 0.088 | 0.287 | 0.306 | 0.760 | 0.837 | 15-23years |
| ODI.UNC.combat | Control (Ref) vs 22q11.2Duplication | 0.736 | 0.319 | 2.307 | 0.022 | 0.052 | 15-23years |
| ODI.PCR.combat | Control (Ref) vs 22q11.2Duplication | -0.364 | 0.315 | -1.155 | 0.249 | 0.378 | 15-23years |
| ODI.PLIC.combat | Control (Ref) vs 22q11.2Duplication | 0.026 | 0.294 | 0.090 | 0.929 | 0.951 | 15-23years |
| ODI.PTR.combat | Control (Ref) vs 22q11.2Duplication | 0.022 | 0.318 | 0.070 | 0.944 | 0.964 | 15-23years |
| ODI.RLIC.combat | Control (Ref) vs 22q11.2Duplication | -0.633 | 0.300 | -2.111 | 0.036 | 0.082 | 15-23years |
| ODI.SCC.combat | Control (Ref) vs 22q11.2Duplication | -0.285 | 0.311 | -0.918 | 0.360 | 0.493 | 15-23years |
| ODI.SCR.combat | Control (Ref) vs 22q11.2Duplication | -0.249 | 0.301 | -0.826 | 0.410 | 0.539 | 15-23years |
| ODI.SFO.combat | Control (Ref) vs 22q11.2Duplication | 0.003 | 0.316 | 0.010 | 0.992 | 0.995 | 15-23years |
| ODI.SLF.combat | Control (Ref) vs 22q11.2Duplication | -0.154 | 0.310 | -0.496 | 0.621 | 0.722 | 15-23years |
| ODI.SS.combat | Control (Ref) vs 22q11.2Duplication | -0.454 | 0.314 | -1.445 | 0.150 | 0.251 | 15-23years |
| ODI.TAP.combat | Control (Ref) vs 22q11.2Duplication | 0.228 | 0.316 | 0.722 | 0.471 | 0.595 | 15-23years |
| ICVF.combat | 22q11.2Deletion (Ref) vs 22q11.2Duplication | -1.455 | 0.188 | -7.727 | 0.000 | 0.000 | 15-23years |
| ISO.combat | 22q11.2Deletion (Ref) vs 22q11.2Duplication | -0.705 | 0.269 | -2.620 | 0.014 | 0.037 | 15-23years |
| ODI.combat | 22q11.2Deletion (Ref) vs 22q11.2Duplication | 0.216 | 0.369 | 0.584 | 0.564 | 0.671 | 15-23years |
| ICVF.ACR.combat | 22q11.2Deletion (Ref) vs 22q11.2Duplication | -1.465 | 0.178 | -8.238 | 0.000 | 0.000 | 15-23years |
| ICVF.ALIC.combat | 22q11.2Deletion (Ref) vs 22q11.2Duplication | -1.505 | 0.218 | -6.901 | 0.000 | 0.000 | 15-23years |
| ICVF.BCC.combat | 22q11.2Deletion (Ref) vs 22q11.2Duplication | -1.404 | 0.204 | -6.897 | 0.000 | 0.000 | 15-23years |
| ICVF.CC.combat | 22q11.2Deletion (Ref) vs 22q11.2Duplication | -1.388 | 0.219 | -6.345 | 0.000 | 0.000 | 15-23years |
| ICVF.CGC.combat | 22q11.2Deletion (Ref) vs 22q11.2Duplication | -1.350 | 0.270 | -5.002 | 0.000 | 0.000 | 15-23years |
| ICVF.CGH.combat | 22q11.2Deletion (Ref) vs 22q11.2Duplication | -1.158 | 0.329 | -3.520 | 0.002 | 0.005 | 15-23years |
| ICVF.CR.combat | 22q11.2Deletion (Ref) vs 22q11.2Duplication | -1.461 | 0.190 | -7.702 | 0.000 | 0.000 | 15-23years |
| ICVF.CST.combat | 22q11.2Deletion (Ref) vs 22q11.2Duplication | -0.934 | 0.294 | -3.171 | 0.004 | 0.012 | 15-23years |
| ICVF.EC.combat | 22q11.2Deletion (Ref) vs 22q11.2Duplication | -1.530 | 0.221 | -6.939 | 0.000 | 0.000 | 15-23years |
| ICVF.FX.combat | 22q11.2Deletion (Ref) vs 22q11.2Duplication | -0.805 | 0.402 | -2.003 | 0.056 | 0.116 | 15-23years |
| ICVF.FXST.combat | 22q11.2Deletion (Ref) vs 22q11.2Duplication | -1.669 | 0.183 | -9.110 | 0.000 | 0.000 | 15-23years |
| ICVF.GCC.combat | 22q11.2Deletion (Ref) vs 22q11.2Duplication | -1.303 | 0.253 | -5.160 | 0.000 | 0.000 | 15-23years |
| ICVF.IC.combat | 22q11.2Deletion (Ref) vs 22q11.2Duplication | -1.422 | 0.232 | -6.137 | 0.000 | 0.000 | 15-23years |
| ICVF.UNC.combat | 22q11.2Deletion (Ref) vs 22q11.2Duplication | -1.411 | 0.222 | -6.360 | 0.000 | 0.000 | 15-23years |
| ICVF.PCR.combat | 22q11.2Deletion (Ref) vs 22q11.2Duplication | -1.381 | 0.217 | -6.353 | 0.000 | 0.000 | 15-23years |
| ICVF.PLIC.combat | 22q11.2Deletion (Ref) vs 22q11.2Duplication | -0.984 | 0.292 | -3.368 | 0.002 | 0.008 | 15-23years |
| ICVF.PTR.combat | 22q11.2Deletion (Ref) vs 22q11.2Duplication | -1.540 | 0.179 | -8.614 | 0.000 | 0.000 | 15-23years |
| ICVF.RLIC.combat | 22q11.2Deletion (Ref) vs 22q11.2Duplication | -1.506 | 0.228 | -6.609 | 0.000 | 0.000 | 15-23years |
| ICVF.SCC.combat | 22q11.2Deletion (Ref) vs 22q11.2Duplication | -1.344 | 0.247 | -5.449 | 0.000 | 0.000 | 15-23years |
| ICVF.SCR.combat | 22q11.2Deletion (Ref) vs 22q11.2Duplication | -1.442 | 0.203 | -7.100 | 0.000 | 0.000 | 15-23years |
| ICVF.SFO.combat | 22q11.2Deletion (Ref) vs 22q11.2Duplication | -1.469 | 0.222 | -6.608 | 0.000 | 0.000 | 15-23years |
| ICVF.SLF.combat | 22q11.2Deletion (Ref) vs 22q11.2Duplication | -1.321 | 0.217 | -6.077 | 0.000 | 0.000 | 15-23years |
| ICVF.SS.combat | 22q11.2Deletion (Ref) vs 22q11.2Duplication | -1.521 | 0.190 | -8.015 | 0.000 | 0.000 | 15-23years |
| ICVF.TAP.combat | 22q11.2Deletion (Ref) vs 22q11.2Duplication | -1.449 | 0.260 | -5.577 | 0.000 | 0.000 | 15-23years |
| ISO.ACR.combat | 22q11.2Deletion (Ref) vs 22q11.2Duplication | -0.534 | 0.306 | -1.747 | 0.092 | 0.172 | 15-23years |
| ISO.ALIC.combat | 22q11.2Deletion (Ref) vs 22q11.2Duplication | -0.402 | 0.437 | -0.920 | 0.366 | 0.497 | 15-23years |
| ISO.BCC.combat | 22q11.2Deletion (Ref) vs 22q11.2Duplication | -0.369 | 0.303 | -1.220 | 0.234 | 0.358 | 15-23years |
| ISO.CC.combat | 22q11.2Deletion (Ref) vs 22q11.2Duplication | -0.283 | 0.337 | -0.838 | 0.409 | 0.539 | 15-23years |
| ISO.CGC.combat | 22q11.2Deletion (Ref) vs 22q11.2Duplication | -0.530 | 0.300 | -1.770 | 0.088 | 0.168 | 15-23years |
| ISO.CGH.combat | 22q11.2Deletion (Ref) vs 22q11.2Duplication | -0.097 | 0.373 | -0.261 | 0.796 | 0.853 | 15-23years |
| ISO.CR.combat | 22q11.2Deletion (Ref) vs 22q11.2Duplication | -0.715 | 0.284 | -2.519 | 0.018 | 0.045 | 15-23years |
| ISO.CST.combat | 22q11.2Deletion (Ref) vs 22q11.2Duplication | -0.005 | 0.426 | -0.012 | 0.991 | 0.995 | 15-23years |
| ISO.EC.combat | 22q11.2Deletion (Ref) vs 22q11.2Duplication | -0.383 | 0.412 | -0.929 | 0.362 | 0.493 | 15-23years |
| ISO.FX.combat | 22q11.2Deletion (Ref) vs 22q11.2Duplication | 0.010 | 0.452 | 0.021 | 0.983 | 0.993 | 15-23years |
| ISO.FXST.combat | 22q11.2Deletion (Ref) vs 22q11.2Duplication | 0.259 | 0.332 | 0.779 | 0.443 | 0.573 | 15-23years |
| ISO.GCC.combat | 22q11.2Deletion (Ref) vs 22q11.2Duplication | -0.126 | 0.379 | -0.333 | 0.742 | 0.830 | 15-23years |
| ISO.IC.combat | 22q11.2Deletion (Ref) vs 22q11.2Duplication | -0.361 | 0.373 | -0.966 | 0.343 | 0.476 | 15-23years |
| ISO.UNC.combat | 22q11.2Deletion (Ref) vs 22q11.2Duplication | -0.367 | 0.369 | -0.994 | 0.329 | 0.466 | 15-23years |
| ISO.PCR.combat | 22q11.2Deletion (Ref) vs 22q11.2Duplication | -0.901 | 0.277 | -3.247 | 0.003 | 0.010 | 15-23years |
| ISO.PLIC.combat | 22q11.2Deletion (Ref) vs 22q11.2Duplication | -0.123 | 0.374 | -0.329 | 0.745 | 0.830 | 15-23years |
| ISO.PTR.combat | 22q11.2Deletion (Ref) vs 22q11.2Duplication | -1.145 | 0.300 | -3.811 | 0.001 | 0.003 | 15-23years |
| ISO.RLIC.combat | 22q11.2Deletion (Ref) vs 22q11.2Duplication | -0.497 | 0.324 | -1.536 | 0.137 | 0.233 | 15-23years |
| ISO.SCC.combat | 22q11.2Deletion (Ref) vs 22q11.2Duplication | -0.138 | 0.395 | -0.350 | 0.730 | 0.820 | 15-23years |
| ISO.SCR.combat | 22q11.2Deletion (Ref) vs 22q11.2Duplication | -0.752 | 0.277 | -2.719 | 0.012 | 0.030 | 15-23years |
| ISO.SFO.combat | 22q11.2Deletion (Ref) vs 22q11.2Duplication | -0.699 | 0.329 | -2.124 | 0.043 | 0.095 | 15-23years |
| ISO.SLF.combat | 22q11.2Deletion (Ref) vs 22q11.2Duplication | -0.593 | 0.284 | -2.089 | 0.047 | 0.101 | 15-23years |
| ISO.SS.combat | 22q11.2Deletion (Ref) vs 22q11.2Duplication | -0.991 | 0.308 | -3.220 | 0.003 | 0.011 | 15-23years |
| ISO.TAP.combat | 22q11.2Deletion (Ref) vs 22q11.2Duplication | -0.716 | 0.373 | -1.922 | 0.066 | 0.130 | 15-23years |
| ODI.ACR.combat | 22q11.2Deletion (Ref) vs 22q11.2Duplication | -0.288 | 0.382 | -0.753 | 0.458 | 0.585 | 15-23years |
| ODI.ALIC.combat | 22q11.2Deletion (Ref) vs 22q11.2Duplication | 0.872 | 0.362 | 2.405 | 0.024 | 0.055 | 15-23years |
| ODI.BCC.combat | 22q11.2Deletion (Ref) vs 22q11.2Duplication | 0.160 | 0.373 | 0.428 | 0.672 | 0.773 | 15-23years |
| ODI.CC.combat | 22q11.2Deletion (Ref) vs 22q11.2Duplication | -0.121 | 0.394 | -0.306 | 0.762 | 0.837 | 15-23years |
| ODI.CGC.combat | 22q11.2Deletion (Ref) vs 22q11.2Duplication | 0.230 | 0.415 | 0.554 | 0.584 | 0.688 | 15-23years |
| ODI.CGH.combat | 22q11.2Deletion (Ref) vs 22q11.2Duplication | -0.504 | 0.403 | -1.251 | 0.222 | 0.349 | 15-23years |
| ODI.CR.combat | 22q11.2Deletion (Ref) vs 22q11.2Duplication | -0.283 | 0.372 | -0.759 | 0.455 | 0.583 | 15-23years |
| ODI.CST.combat | 22q11.2Deletion (Ref) vs 22q11.2Duplication | 0.363 | 0.382 | 0.952 | 0.350 | 0.484 | 15-23years |
| ODI.EC.combat | 22q11.2Deletion (Ref) vs 22q11.2Duplication | 0.148 | 0.394 | 0.377 | 0.709 | 0.800 | 15-23years |
| ODI.FX.combat | 22q11.2Deletion (Ref) vs 22q11.2Duplication | 0.098 | 0.423 | 0.232 | 0.818 | 0.868 | 15-23years |
| ODI.FXST.combat | 22q11.2Deletion (Ref) vs 22q11.2Duplication | -0.314 | 0.395 | -0.793 | 0.435 | 0.565 | 15-23years |
| ODI.GCC.combat | 22q11.2Deletion (Ref) vs 22q11.2Duplication | -0.322 | 0.376 | -0.855 | 0.401 | 0.532 | 15-23years |
| ODI.IC.combat | 22q11.2Deletion (Ref) vs 22q11.2Duplication | 0.293 | 0.404 | 0.726 | 0.474 | 0.595 | 15-23years |
| ODI.UNC.combat | 22q11.2Deletion (Ref) vs 22q11.2Duplication | 0.890 | 0.384 | 2.320 | 0.028 | 0.065 | 15-23years |
| ODI.PCR.combat | 22q11.2Deletion (Ref) vs 22q11.2Duplication | -0.309 | 0.388 | -0.796 | 0.433 | 0.565 | 15-23years |
| ODI.PLIC.combat | 22q11.2Deletion (Ref) vs 22q11.2Duplication | 0.269 | 0.386 | 0.698 | 0.492 | 0.610 | 15-23years |
| ODI.PTR.combat | 22q11.2Deletion (Ref) vs 22q11.2Duplication | -0.722 | 0.374 | -1.928 | 0.065 | 0.130 | 15-23years |
| ODI.RLIC.combat | 22q11.2Deletion (Ref) vs 22q11.2Duplication | -0.849 | 0.362 | -2.345 | 0.027 | 0.063 | 15-23years |
| ODI.SCC.combat | 22q11.2Deletion (Ref) vs 22q11.2Duplication | -0.271 | 0.397 | -0.682 | 0.501 | 0.616 | 15-23years |
| ODI.SCR.combat | 22q11.2Deletion (Ref) vs 22q11.2Duplication | -0.228 | 0.415 | -0.549 | 0.588 | 0.689 | 15-23years |
| ODI.SFO.combat | 22q11.2Deletion (Ref) vs 22q11.2Duplication | -0.416 | 0.406 | -1.024 | 0.315 | 0.451 | 15-23years |
| ODI.SLF.combat | 22q11.2Deletion (Ref) vs 22q11.2Duplication | -0.866 | 0.332 | -2.606 | 0.015 | 0.037 | 15-23years |
| ODI.SS.combat | 22q11.2Deletion (Ref) vs 22q11.2Duplication | -0.796 | 0.376 | -2.115 | 0.044 | 0.096 | 15-23years |
| ODI.TAP.combat | 22q11.2Deletion (Ref) vs 22q11.2Duplication | 0.551 | 0.407 | 1.354 | 0.187 | 0.302 | 15-23years |
| ICVF.combat | GeneDosage (1 = del, 2 = con, 3 dup) | -1.277 | 0.185 | -6.899 | 0.000 | 0.000 | 15-23years |
| ISO.combat | GeneDosage (1 = del, 2 = con, 3 dup) | -0.337 | 0.178 | -1.889 | 0.060 | 0.121 | 15-23years |
| ODI.combat | GeneDosage (1 = del, 2 = con, 3 dup) | -0.304 | 0.208 | -1.465 | 0.144 | 0.243 | 15-23years |
| ICVF.ACR.combat | GeneDosage (1 = del, 2 = con, 3 dup) | -1.392 | 0.186 | -7.464 | 0.000 | 0.000 | 15-23years |
| ICVF.ALIC.combat | GeneDosage (1 = del, 2 = con, 3 dup) | -0.879 | 0.185 | -4.754 | 0.000 | 0.000 | 15-23years |
| ICVF.BCC.combat | GeneDosage (1 = del, 2 = con, 3 dup) | -1.369 | 0.186 | -7.351 | 0.000 | 0.000 | 15-23years |
| ICVF.CC.combat | GeneDosage (1 = del, 2 = con, 3 dup) | -1.337 | 0.187 | -7.144 | 0.000 | 0.000 | 15-23years |
| ICVF.CGC.combat | GeneDosage (1 = del, 2 = con, 3 dup) | -0.838 | 0.193 | -4.345 | 0.000 | 0.000 | 15-23years |
| ICVF.CGH.combat | GeneDosage (1 = del, 2 = con, 3 dup) | -0.545 | 0.200 | -2.720 | 0.007 | 0.019 | 15-23years |
| ICVF.CR.combat | GeneDosage (1 = del, 2 = con, 3 dup) | -1.490 | 0.184 | -8.104 | 0.000 | 0.000 | 15-23years |
| ICVF.CST.combat | GeneDosage (1 = del, 2 = con, 3 dup) | -0.429 | 0.172 | -2.491 | 0.013 | 0.034 | 15-23years |
| ICVF.EC.combat | GeneDosage (1 = del, 2 = con, 3 dup) | -0.905 | 0.201 | -4.494 | 0.000 | 0.000 | 15-23years |
| ICVF.FX.combat | GeneDosage (1 = del, 2 = con, 3 dup) | -0.213 | 0.212 | -1.005 | 0.316 | 0.451 | 15-23years |
| ICVF.FXST.combat | GeneDosage (1 = del, 2 = con, 3 dup) | -0.940 | 0.204 | -4.619 | 0.000 | 0.000 | 15-23years |
| ICVF.GCC.combat | GeneDosage (1 = del, 2 = con, 3 dup) | -1.193 | 0.194 | -6.145 | 0.000 | 0.000 | 15-23years |
| ICVF.IC.combat | GeneDosage (1 = del, 2 = con, 3 dup) | -1.036 | 0.173 | -5.976 | 0.000 | 0.000 | 15-23years |
| ICVF.UNC.combat | GeneDosage (1 = del, 2 = con, 3 dup) | -1.224 | 0.195 | -6.265 | 0.000 | 0.000 | 15-23years |
| ICVF.PCR.combat | GeneDosage (1 = del, 2 = con, 3 dup) | -1.555 | 0.183 | -8.504 | 0.000 | 0.000 | 15-23years |
| ICVF.PLIC.combat | GeneDosage (1 = del, 2 = con, 3 dup) | -0.898 | 0.173 | -5.182 | 0.000 | 0.000 | 15-23years |
| ICVF.PTR.combat | GeneDosage (1 = del, 2 = con, 3 dup) | -1.528 | 0.189 | -8.073 | 0.000 | 0.000 | 15-23years |
| ICVF.RLIC.combat | GeneDosage (1 = del, 2 = con, 3 dup) | -1.108 | 0.196 | -5.646 | 0.000 | 0.000 | 15-23years |
| ICVF.SCC.combat | GeneDosage (1 = del, 2 = con, 3 dup) | -1.210 | 0.195 | -6.192 | 0.000 | 0.000 | 15-23years |
| ICVF.SCR.combat | GeneDosage (1 = del, 2 = con, 3 dup) | -1.434 | 0.186 | -7.710 | 0.000 | 0.000 | 15-23years |
| ICVF.SFO.combat | GeneDosage (1 = del, 2 = con, 3 dup) | -0.947 | 0.199 | -4.761 | 0.000 | 0.000 | 15-23years |
| ICVF.SLF.combat | GeneDosage (1 = del, 2 = con, 3 dup) | -1.382 | 0.186 | -7.420 | 0.000 | 0.000 | 15-23years |
| ICVF.SS.combat | GeneDosage (1 = del, 2 = con, 3 dup) | -1.289 | 0.195 | -6.601 | 0.000 | 0.000 | 15-23years |
| ICVF.TAP.combat | GeneDosage (1 = del, 2 = con, 3 dup) | -1.257 | 0.199 | -6.316 | 0.000 | 0.000 | 15-23years |
| ISO.ACR.combat | GeneDosage (1 = del, 2 = con, 3 dup) | -0.336 | 0.183 | -1.831 | 0.068 | 0.134 | 15-23years |
| ISO.ALIC.combat | GeneDosage (1 = del, 2 = con, 3 dup) | -0.196 | 0.218 | -0.898 | 0.370 | 0.500 | 15-23years |
| ISO.BCC.combat | GeneDosage (1 = del, 2 = con, 3 dup) | 0.062 | 0.217 | 0.284 | 0.776 | 0.844 | 15-23years |
| ISO.CC.combat | GeneDosage (1 = del, 2 = con, 3 dup) | 0.048 | 0.218 | 0.221 | 0.826 | 0.872 | 15-23years |
| ISO.CGC.combat | GeneDosage (1 = del, 2 = con, 3 dup) | -0.202 | 0.171 | -1.185 | 0.237 | 0.361 | 15-23years |
| ISO.CGH.combat | GeneDosage (1 = del, 2 = con, 3 dup) | 0.054 | 0.186 | 0.293 | 0.770 | 0.843 | 15-23years |
| ISO.CR.combat | GeneDosage (1 = del, 2 = con, 3 dup) | -0.387 | 0.186 | -2.081 | 0.038 | 0.086 | 15-23years |
| ISO.CST.combat | GeneDosage (1 = del, 2 = con, 3 dup) | -0.154 | 0.214 | -0.720 | 0.472 | 0.595 | 15-23years |
| ISO.EC.combat | GeneDosage (1 = del, 2 = con, 3 dup) | 0.103 | 0.224 | 0.459 | 0.647 | 0.747 | 15-23years |
| ISO.FX.combat | GeneDosage (1 = del, 2 = con, 3 dup) | -0.253 | 0.225 | -1.126 | 0.261 | 0.388 | 15-23years |
| ISO.FXST.combat | GeneDosage (1 = del, 2 = con, 3 dup) | 0.297 | 0.196 | 1.517 | 0.131 | 0.226 | 15-23years |
| ISO.GCC.combat | GeneDosage (1 = del, 2 = con, 3 dup) | -0.043 | 0.214 | -0.199 | 0.843 | 0.887 | 15-23years |
| ISO.IC.combat | GeneDosage (1 = del, 2 = con, 3 dup) | -0.061 | 0.209 | -0.290 | 0.772 | 0.843 | 15-23years |
| ISO.UNC.combat | GeneDosage (1 = del, 2 = con, 3 dup) | -0.233 | 0.189 | -1.231 | 0.219 | 0.347 | 15-23years |
| ISO.PCR.combat | GeneDosage (1 = del, 2 = con, 3 dup) | -0.588 | 0.181 | -3.246 | 0.001 | 0.005 | 15-23years |
| ISO.PLIC.combat | GeneDosage (1 = del, 2 = con, 3 dup) | -0.009 | 0.206 | -0.044 | 0.965 | 0.981 | 15-23years |
| ISO.PTR.combat | GeneDosage (1 = del, 2 = con, 3 dup) | -0.589 | 0.212 | -2.775 | 0.006 | 0.017 | 15-23years |
| ISO.RLIC.combat | GeneDosage (1 = del, 2 = con, 3 dup) | 0.061 | 0.200 | 0.305 | 0.761 | 0.837 | 15-23years |
| ISO.SCC.combat | GeneDosage (1 = del, 2 = con, 3 dup) | 0.137 | 0.221 | 0.620 | 0.536 | 0.646 | 15-23years |
| ISO.SCR.combat | GeneDosage (1 = del, 2 = con, 3 dup) | -0.278 | 0.203 | -1.367 | 0.173 | 0.283 | 15-23years |
| ISO.SFO.combat | GeneDosage (1 = del, 2 = con, 3 dup) | -0.282 | 0.204 | -1.382 | 0.168 | 0.279 | 15-23years |
| ISO.SLF.combat | GeneDosage (1 = del, 2 = con, 3 dup) | -0.316 | 0.189 | -1.670 | 0.096 | 0.178 | 15-23years |
| ISO.SS.combat | GeneDosage (1 = del, 2 = con, 3 dup) | -0.192 | 0.219 | -0.877 | 0.381 | 0.511 | 15-23years |
| ISO.TAP.combat | GeneDosage (1 = del, 2 = con, 3 dup) | -0.500 | 0.218 | -2.298 | 0.022 | 0.053 | 15-23years |
| ODI.ACR.combat | GeneDosage (1 = del, 2 = con, 3 dup) | -0.442 | 0.214 | -2.062 | 0.040 | 0.089 | 15-23years |
| ODI.ALIC.combat | GeneDosage (1 = del, 2 = con, 3 dup) | 0.626 | 0.209 | 3.002 | 0.003 | 0.009 | 15-23years |
| ODI.BCC.combat | GeneDosage (1 = del, 2 = con, 3 dup) | -0.036 | 0.217 | -0.168 | 0.866 | 0.903 | 15-23years |
| ODI.CC.combat | GeneDosage (1 = del, 2 = con, 3 dup) | -0.248 | 0.219 | -1.131 | 0.259 | 0.387 | 15-23years |
| ODI.CGC.combat | GeneDosage (1 = del, 2 = con, 3 dup) | 0.194 | 0.223 | 0.870 | 0.385 | 0.514 | 15-23years |
| ODI.CGH.combat | GeneDosage (1 = del, 2 = con, 3 dup) | -0.566 | 0.210 | -2.698 | 0.007 | 0.020 | 15-23years |
| ODI.CR.combat | GeneDosage (1 = del, 2 = con, 3 dup) | -0.408 | 0.214 | -1.905 | 0.058 | 0.118 | 15-23years |
| ODI.CST.combat | GeneDosage (1 = del, 2 = con, 3 dup) | 0.228 | 0.209 | 1.089 | 0.277 | 0.406 | 15-23years |
| ODI.EC.combat | GeneDosage (1 = del, 2 = con, 3 dup) | -0.496 | 0.210 | -2.364 | 0.019 | 0.046 | 15-23years |
| ODI.FX.combat | GeneDosage (1 = del, 2 = con, 3 dup) | -0.235 | 0.213 | -1.101 | 0.272 | 0.400 | 15-23years |
| ODI.FXST.combat | GeneDosage (1 = del, 2 = con, 3 dup) | -0.227 | 0.209 | -1.083 | 0.280 | 0.408 | 15-23years |
| ODI.GCC.combat | GeneDosage (1 = del, 2 = con, 3 dup) | -0.508 | 0.216 | -2.358 | 0.019 | 0.046 | 15-23years |
| ODI.IC.combat | GeneDosage (1 = del, 2 = con, 3 dup) | 0.391 | 0.213 | 1.833 | 0.068 | 0.134 | 15-23years |
| ODI.UNC.combat | GeneDosage (1 = del, 2 = con, 3 dup) | 0.324 | 0.230 | 1.412 | 0.159 | 0.266 | 15-23years |
| ODI.PCR.combat | GeneDosage (1 = del, 2 = con, 3 dup) | -0.328 | 0.217 | -1.513 | 0.132 | 0.227 | 15-23years |
| ODI.PLIC.combat | GeneDosage (1 = del, 2 = con, 3 dup) | 0.504 | 0.217 | 2.322 | 0.021 | 0.051 | 15-23years |
| ODI.PTR.combat | GeneDosage (1 = del, 2 = con, 3 dup) | -0.666 | 0.217 | -3.068 | 0.002 | 0.008 | 15-23years |
| ODI.RLIC.combat | GeneDosage (1 = del, 2 = con, 3 dup) | -0.250 | 0.220 | -1.137 | 0.256 | 0.385 | 15-23years |
| ODI.SCC.combat | GeneDosage (1 = del, 2 = con, 3 dup) | -0.150 | 0.225 | -0.668 | 0.505 | 0.618 | 15-23years |
| ODI.SCR.combat | GeneDosage (1 = del, 2 = con, 3 dup) | -0.213 | 0.220 | -0.971 | 0.333 | 0.468 | 15-23years |
| ODI.SFO.combat | GeneDosage (1 = del, 2 = con, 3 dup) | -0.546 | 0.216 | -2.524 | 0.012 | 0.031 | 15-23years |
| ODI.SLF.combat | GeneDosage (1 = del, 2 = con, 3 dup) | -0.949 | 0.207 | -4.583 | 0.000 | 0.000 | 15-23years |
| ODI.SS.combat | GeneDosage (1 = del, 2 = con, 3 dup) | -0.523 | 0.221 | -2.372 | 0.018 | 0.045 | 15-23years |
| ODI.TAP.combat | GeneDosage (1 = del, 2 = con, 3 dup) | 0.358 | 0.225 | 1.592 | 0.113 | 0.202 | 15-23years |
| ICVF.combat | Control (Ref) vs 22q11.2Deletion | 0.616 | 0.190 | 3.244 | 0.001 | 0.007 | >23years |
| ISO.combat | Control (Ref) vs 22q11.2Deletion | -0.263 | 0.191 | -1.372 | 0.171 | 0.287 | >23years |
| ODI.combat | Control (Ref) vs 22q11.2Deletion | 0.225 | 0.221 | 1.017 | 0.310 | 0.467 | >23years |
| ICVF.ACR.combat | Control (Ref) vs 22q11.2Deletion | 0.623 | 0.187 | 3.332 | 0.001 | 0.005 | >23years |
| ICVF.ALIC.combat | Control (Ref) vs 22q11.2Deletion | 0.323 | 0.176 | 1.833 | 0.068 | 0.146 | >23years |
| ICVF.BCC.combat | Control (Ref) vs 22q11.2Deletion | 0.792 | 0.182 | 4.352 | 0.000 | 0.000 | >23years |
| ICVF.CC.combat | Control (Ref) vs 22q11.2Deletion | 0.772 | 0.183 | 4.225 | 0.000 | 0.000 | >23years |
| ICVF.CGC.combat | Control (Ref) vs 22q11.2Deletion | 0.438 | 0.187 | 2.338 | 0.020 | 0.056 | >23years |
| ICVF.CGH.combat | Control (Ref) vs 22q11.2Deletion | 0.110 | 0.185 | 0.591 | 0.555 | 0.674 | >23years |
| ICVF.CR.combat | Control (Ref) vs 22q11.2Deletion | 0.693 | 0.186 | 3.715 | 0.000 | 0.002 | >23years |
| ICVF.CST.combat | Control (Ref) vs 22q11.2Deletion | 0.141 | 0.186 | 0.757 | 0.450 | 0.584 | >23years |
| ICVF.EC.combat | Control (Ref) vs 22q11.2Deletion | 0.489 | 0.191 | 2.563 | 0.011 | 0.037 | >23years |
| ICVF.FX.combat | Control (Ref) vs 22q11.2Deletion | 0.183 | 0.191 | 0.960 | 0.338 | 0.499 | >23years |
| ICVF.FXST.combat | Control (Ref) vs 22q11.2Deletion | 0.581 | 0.188 | 3.093 | 0.002 | 0.011 | >23years |
| ICVF.GCC.combat | Control (Ref) vs 22q11.2Deletion | 0.667 | 0.183 | 3.637 | 0.000 | 0.002 | >23years |
| ICVF.IC.combat | Control (Ref) vs 22q11.2Deletion | 0.525 | 0.180 | 2.918 | 0.004 | 0.017 | >23years |
| ICVF.UNC.combat | Control (Ref) vs 22q11.2Deletion | 0.828 | 0.204 | 4.059 | 0.000 | 0.001 | >23years |
| ICVF.PCR.combat | Control (Ref) vs 22q11.2Deletion | 0.840 | 0.183 | 4.582 | 0.000 | 0.000 | >23years |
| ICVF.PLIC.combat | Control (Ref) vs 22q11.2Deletion | 0.392 | 0.180 | 2.185 | 0.030 | 0.074 | >23years |
| ICVF.PTR.combat | Control (Ref) vs 22q11.2Deletion | 0.752 | 0.184 | 4.098 | 0.000 | 0.001 | >23years |
| ICVF.RLIC.combat | Control (Ref) vs 22q11.2Deletion | 0.709 | 0.188 | 3.779 | 0.000 | 0.001 | >23years |
| ICVF.SCC.combat | Control (Ref) vs 22q11.2Deletion | 0.729 | 0.187 | 3.897 | 0.000 | 0.001 | >23years |
| ICVF.SCR.combat | Control (Ref) vs 22q11.2Deletion | 0.631 | 0.189 | 3.334 | 0.001 | 0.005 | >23years |
| ICVF.SFO.combat | Control (Ref) vs 22q11.2Deletion | 0.373 | 0.193 | 1.934 | 0.054 | 0.121 | >23years |
| ICVF.SLF.combat | Control (Ref) vs 22q11.2Deletion | 0.678 | 0.188 | 3.602 | 0.000 | 0.002 | >23years |
| ICVF.SS.combat | Control (Ref) vs 22q11.2Deletion | 0.790 | 0.183 | 4.309 | 0.000 | 0.000 | >23years |
| ICVF.TAP.combat | Control (Ref) vs 22q11.2Deletion | 0.889 | 0.176 | 5.066 | 0.000 | 0.000 | >23years |
| ISO.ACR.combat | Control (Ref) vs 22q11.2Deletion | -0.313 | 0.196 | -1.596 | 0.112 | 0.213 | >23years |
| ISO.ALIC.combat | Control (Ref) vs 22q11.2Deletion | -0.355 | 0.222 | -1.600 | 0.111 | 0.213 | >23years |
| ISO.BCC.combat | Control (Ref) vs 22q11.2Deletion | -0.345 | 0.192 | -1.792 | 0.074 | 0.158 | >23years |
| ISO.CC.combat | Control (Ref) vs 22q11.2Deletion | -0.468 | 0.193 | -2.419 | 0.016 | 0.049 | >23years |
| ISO.CGC.combat | Control (Ref) vs 22q11.2Deletion | -0.300 | 0.185 | -1.619 | 0.107 | 0.209 | >23years |
| ISO.CGH.combat | Control (Ref) vs 22q11.2Deletion | 0.044 | 0.177 | 0.247 | 0.805 | 0.864 | >23years |
| ISO.CR.combat | Control (Ref) vs 22q11.2Deletion | -0.253 | 0.194 | -1.308 | 0.192 | 0.317 | >23years |
| ISO.CST.combat | Control (Ref) vs 22q11.2Deletion | -0.165 | 0.225 | -0.733 | 0.464 | 0.597 | >23years |
| ISO.EC.combat | Control (Ref) vs 22q11.2Deletion | -0.115 | 0.193 | -0.596 | 0.552 | 0.673 | >23years |
| ISO.FX.combat | Control (Ref) vs 22q11.2Deletion | 0.338 | 0.192 | 1.760 | 0.079 | 0.166 | >23years |
| ISO.FXST.combat | Control (Ref) vs 22q11.2Deletion | -0.506 | 0.170 | -2.971 | 0.003 | 0.015 | >23years |
| ISO.GCC.combat | Control (Ref) vs 22q11.2Deletion | -0.450 | 0.195 | -2.302 | 0.022 | 0.060 | >23years |
| ISO.IC.combat | Control (Ref) vs 22q11.2Deletion | -0.094 | 0.190 | -0.493 | 0.622 | 0.726 | >23years |
| ISO.UNC.combat | Control (Ref) vs 22q11.2Deletion | -0.281 | 0.180 | -1.559 | 0.120 | 0.221 | >23years |
| ISO.PCR.combat | Control (Ref) vs 22q11.2Deletion | -0.178 | 0.195 | -0.915 | 0.361 | 0.516 | >23years |
| ISO.PLIC.combat | Control (Ref) vs 22q11.2Deletion | -0.051 | 0.194 | -0.262 | 0.794 | 0.859 | >23years |
| ISO.PTR.combat | Control (Ref) vs 22q11.2Deletion | 0.026 | 0.196 | 0.133 | 0.894 | 0.919 | >23years |
| ISO.RLIC.combat | Control (Ref) vs 22q11.2Deletion | 0.005 | 0.191 | 0.024 | 0.981 | 0.988 | >23years |
| ISO.SCC.combat | Control (Ref) vs 22q11.2Deletion | -0.406 | 0.194 | -2.091 | 0.037 | 0.089 | >23years |
| ISO.SCR.combat | Control (Ref) vs 22q11.2Deletion | -0.135 | 0.190 | -0.713 | 0.476 | 0.605 | >23years |
| ISO.SFO.combat | Control (Ref) vs 22q11.2Deletion | -0.079 | 0.216 | -0.364 | 0.716 | 0.795 | >23years |
| ISO.SLF.combat | Control (Ref) vs 22q11.2Deletion | -0.177 | 0.190 | -0.928 | 0.354 | 0.515 | >23years |
| ISO.SS.combat | Control (Ref) vs 22q11.2Deletion | -0.161 | 0.194 | -0.832 | 0.406 | 0.549 | >23years |
| ISO.TAP.combat | Control (Ref) vs 22q11.2Deletion | 0.102 | 0.216 | 0.473 | 0.637 | 0.738 | >23years |
| ODI.ACR.combat | Control (Ref) vs 22q11.2Deletion | 0.142 | 0.223 | 0.639 | 0.523 | 0.651 | >23years |
| ODI.ALIC.combat | Control (Ref) vs 22q11.2Deletion | -0.506 | 0.222 | -2.276 | 0.024 | 0.061 | >23years |
| ODI.BCC.combat | Control (Ref) vs 22q11.2Deletion | 0.610 | 0.231 | 2.644 | 0.009 | 0.033 | >23years |
| ODI.CC.combat | Control (Ref) vs 22q11.2Deletion | 0.531 | 0.228 | 2.330 | 0.021 | 0.057 | >23years |
| ODI.CGC.combat | Control (Ref) vs 22q11.2Deletion | 0.054 | 0.231 | 0.233 | 0.816 | 0.864 | >23years |
| ODI.CGH.combat | Control (Ref) vs 22q11.2Deletion | 0.568 | 0.228 | 2.495 | 0.013 | 0.043 | >23years |
| ODI.CR.combat | Control (Ref) vs 22q11.2Deletion | 0.133 | 0.228 | 0.581 | 0.562 | 0.677 | >23years |
| ODI.CST.combat | Control (Ref) vs 22q11.2Deletion | -0.383 | 0.189 | -2.022 | 0.044 | 0.102 | >23years |
| ODI.EC.combat | Control (Ref) vs 22q11.2Deletion | 0.549 | 0.185 | 2.966 | 0.003 | 0.015 | >23years |
| ODI.FX.combat | Control (Ref) vs 22q11.2Deletion | 0.211 | 0.187 | 1.126 | 0.261 | 0.406 | >23years |
| ODI.FXST.combat | Control (Ref) vs 22q11.2Deletion | 0.347 | 0.220 | 1.573 | 0.117 | 0.220 | >23years |
| ODI.GCC.combat | Control (Ref) vs 22q11.2Deletion | 0.102 | 0.220 | 0.463 | 0.644 | 0.743 | >23years |
| ODI.IC.combat | Control (Ref) vs 22q11.2Deletion | -0.309 | 0.182 | -1.701 | 0.090 | 0.183 | >23years |
| ODI.UNC.combat | Control (Ref) vs 22q11.2Deletion | 0.276 | 0.194 | 1.424 | 0.155 | 0.270 | >23years |
| ODI.PCR.combat | Control (Ref) vs 22q11.2Deletion | 0.400 | 0.195 | 2.051 | 0.041 | 0.097 | >23years |
| ODI.PLIC.combat | Control (Ref) vs 22q11.2Deletion | -0.415 | 0.181 | -2.292 | 0.023 | 0.060 | >23years |
| ODI.PTR.combat | Control (Ref) vs 22q11.2Deletion | 0.813 | 0.204 | 3.980 | 0.000 | 0.001 | >23years |
| ODI.RLIC.combat | Control (Ref) vs 22q11.2Deletion | 0.157 | 0.216 | 0.730 | 0.466 | 0.597 | >23years |
| ODI.SCC.combat | Control (Ref) vs 22q11.2Deletion | 0.524 | 0.220 | 2.383 | 0.018 | 0.053 | >23years |
| ODI.SCR.combat | Control (Ref) vs 22q11.2Deletion | -0.124 | 0.229 | -0.539 | 0.591 | 0.703 | >23years |
| ODI.SFO.combat | Control (Ref) vs 22q11.2Deletion | 0.564 | 0.228 | 2.480 | 0.014 | 0.044 | >23years |
| ODI.SLF.combat | Control (Ref) vs 22q11.2Deletion | 0.519 | 0.194 | 2.678 | 0.008 | 0.031 | >23years |
| ODI.SS.combat | Control (Ref) vs 22q11.2Deletion | 0.403 | 0.232 | 1.739 | 0.083 | 0.170 | >23years |
| ODI.TAP.combat | Control (Ref) vs 22q11.2Deletion | 0.174 | 0.212 | 0.818 | 0.414 | 0.555 | >23years |
| ICVF.combat | Control (Ref) vs 22q11.2Duplication | -0.489 | 0.263 | -1.857 | 0.064 | 0.141 | >23years |
| ISO.combat | Control (Ref) vs 22q11.2Duplication | -1.002 | 0.253 | -3.967 | 0.000 | 0.001 | >23years |
| ODI.combat | Control (Ref) vs 22q11.2Duplication | 0.073 | 0.246 | 0.297 | 0.766 | 0.842 | >23years |
| ICVF.ACR.combat | Control (Ref) vs 22q11.2Duplication | -0.440 | 0.265 | -1.663 | 0.098 | 0.194 | >23years |
| ICVF.ALIC.combat | Control (Ref) vs 22q11.2Duplication | -0.195 | 0.232 | -0.840 | 0.402 | 0.548 | >23years |
| ICVF.BCC.combat | Control (Ref) vs 22q11.2Duplication | -0.815 | 0.260 | -3.133 | 0.002 | 0.010 | >23years |
| ICVF.CC.combat | Control (Ref) vs 22q11.2Duplication | -0.749 | 0.261 | -2.869 | 0.004 | 0.019 | >23years |
| ICVF.CGC.combat | Control (Ref) vs 22q11.2Duplication | -0.452 | 0.259 | -1.746 | 0.082 | 0.169 | >23years |
| ICVF.CGH.combat | Control (Ref) vs 22q11.2Duplication | -0.313 | 0.249 | -1.254 | 0.211 | 0.340 | >23years |
| ICVF.CR.combat | Control (Ref) vs 22q11.2Duplication | -0.544 | 0.264 | -2.064 | 0.040 | 0.095 | >23years |
| ICVF.CST.combat | Control (Ref) vs 22q11.2Duplication | -0.058 | 0.251 | -0.230 | 0.818 | 0.864 | >23years |
| ICVF.EC.combat | Control (Ref) vs 22q11.2Duplication | -0.064 | 0.262 | -0.246 | 0.806 | 0.864 | >23years |
| ICVF.FX.combat | Control (Ref) vs 22q11.2Duplication | -0.220 | 0.263 | -0.835 | 0.404 | 0.549 | >23years |
| ICVF.FXST.combat | Control (Ref) vs 22q11.2Duplication | 0.016 | 0.260 | 0.060 | 0.952 | 0.968 | >23years |
| ICVF.GCC.combat | Control (Ref) vs 22q11.2Duplication | -0.676 | 0.261 | -2.592 | 0.010 | 0.036 | >23years |
| ICVF.IC.combat | Control (Ref) vs 22q11.2Duplication | -0.209 | 0.247 | -0.848 | 0.397 | 0.546 | >23years |
| ICVF.UNC.combat | Control (Ref) vs 22q11.2Duplication | -0.231 | 0.266 | -0.869 | 0.386 | 0.535 | >23years |
| ICVF.PCR.combat | Control (Ref) vs 22q11.2Duplication | -0.641 | 0.264 | -2.426 | 0.016 | 0.048 | >23years |
| ICVF.PLIC.combat | Control (Ref) vs 22q11.2Duplication | -0.197 | 0.251 | -0.785 | 0.433 | 0.571 | >23years |
| ICVF.PTR.combat | Control (Ref) vs 22q11.2Duplication | -0.641 | 0.264 | -2.434 | 0.016 | 0.048 | >23years |
| ICVF.RLIC.combat | Control (Ref) vs 22q11.2Duplication | -0.241 | 0.262 | -0.920 | 0.359 | 0.515 | >23years |
| ICVF.SCC.combat | Control (Ref) vs 22q11.2Duplication | -0.607 | 0.265 | -2.295 | 0.023 | 0.060 | >23years |
| ICVF.SCR.combat | Control (Ref) vs 22q11.2Duplication | -0.569 | 0.263 | -2.168 | 0.031 | 0.076 | >23years |
| ICVF.SFO.combat | Control (Ref) vs 22q11.2Duplication | -0.384 | 0.254 | -1.516 | 0.131 | 0.235 | >23years |
| ICVF.SLF.combat | Control (Ref) vs 22q11.2Duplication | -0.583 | 0.265 | -2.201 | 0.029 | 0.073 | >23years |
| ICVF.SS.combat | Control (Ref) vs 22q11.2Duplication | -0.386 | 0.267 | -1.443 | 0.150 | 0.264 | >23years |
| ICVF.TAP.combat | Control (Ref) vs 22q11.2Duplication | -0.580 | 0.260 | -2.231 | 0.027 | 0.068 | >23years |
| ISO.ACR.combat | Control (Ref) vs 22q11.2Duplication | -0.962 | 0.258 | -3.727 | 0.000 | 0.002 | >23years |
| ISO.ALIC.combat | Control (Ref) vs 22q11.2Duplication | -0.568 | 0.242 | -2.352 | 0.019 | 0.056 | >23years |
| ISO.BCC.combat | Control (Ref) vs 22q11.2Duplication | -0.689 | 0.261 | -2.644 | 0.009 | 0.033 | >23years |
| ISO.CC.combat | Control (Ref) vs 22q11.2Duplication | -0.680 | 0.263 | -2.589 | 0.010 | 0.036 | >23years |
| ISO.CGC.combat | Control (Ref) vs 22q11.2Duplication | -0.364 | 0.257 | -1.419 | 0.157 | 0.271 | >23years |
| ISO.CGH.combat | Control (Ref) vs 22q11.2Duplication | -0.279 | 0.240 | -1.163 | 0.246 | 0.384 | >23years |
| ISO.CR.combat | Control (Ref) vs 22q11.2Duplication | -1.108 | 0.253 | -4.381 | 0.000 | 0.000 | >23years |
| ISO.CST.combat | Control (Ref) vs 22q11.2Duplication | 0.182 | 0.229 | 0.796 | 0.427 | 0.566 | >23years |
| ISO.EC.combat | Control (Ref) vs 22q11.2Duplication | -0.234 | 0.253 | -0.923 | 0.357 | 0.515 | >23years |
| ISO.FX.combat | Control (Ref) vs 22q11.2Duplication | 0.134 | 0.261 | 0.512 | 0.609 | 0.714 | >23years |
| ISO.FXST.combat | Control (Ref) vs 22q11.2Duplication | -0.646 | 0.249 | -2.593 | 0.010 | 0.036 | >23years |
| ISO.GCC.combat | Control (Ref) vs 22q11.2Duplication | -0.610 | 0.265 | -2.303 | 0.022 | 0.060 | >23years |
| ISO.IC.combat | Control (Ref) vs 22q11.2Duplication | -0.573 | 0.236 | -2.430 | 0.016 | 0.048 | >23years |
| ISO.UNC.combat | Control (Ref) vs 22q11.2Duplication | -0.423 | 0.228 | -1.855 | 0.065 | 0.141 | >23years |
| ISO.PCR.combat | Control (Ref) vs 22q11.2Duplication | -0.979 | 0.254 | -3.851 | 0.000 | 0.001 | >23years |
| ISO.PLIC.combat | Control (Ref) vs 22q11.2Duplication | -0.224 | 0.250 | -0.896 | 0.371 | 0.523 | >23years |
| ISO.PTR.combat | Control (Ref) vs 22q11.2Duplication | -0.722 | 0.263 | -2.750 | 0.006 | 0.026 | >23years |
| ISO.RLIC.combat | Control (Ref) vs 22q11.2Duplication | -0.571 | 0.223 | -2.558 | 0.011 | 0.037 | >23years |
| ISO.SCC.combat | Control (Ref) vs 22q11.2Duplication | -0.416 | 0.268 | -1.554 | 0.121 | 0.221 | >23years |
| ISO.SCR.combat | Control (Ref) vs 22q11.2Duplication | -1.152 | 0.251 | -4.590 | 0.000 | 0.000 | >23years |
| ISO.SFO.combat | Control (Ref) vs 22q11.2Duplication | -0.657 | 0.254 | -2.589 | 0.010 | 0.036 | >23years |
| ISO.SLF.combat | Control (Ref) vs 22q11.2Duplication | -1.032 | 0.249 | -4.153 | 0.000 | 0.000 | >23years |
| ISO.SS.combat | Control (Ref) vs 22q11.2Duplication | -0.489 | 0.252 | -1.938 | 0.054 | 0.121 | >23years |
| ISO.TAP.combat | Control (Ref) vs 22q11.2Duplication | -0.639 | 0.265 | -2.410 | 0.017 | 0.049 | >23years |
| ODI.ACR.combat | Control (Ref) vs 22q11.2Duplication | -0.104 | 0.265 | -0.392 | 0.695 | 0.775 | >23years |
| ODI.ALIC.combat | Control (Ref) vs 22q11.2Duplication | 0.257 | 0.254 | 1.013 | 0.312 | 0.468 | >23years |
| ODI.BCC.combat | Control (Ref) vs 22q11.2Duplication | 0.127 | 0.260 | 0.488 | 0.626 | 0.728 | >23years |
| ODI.CC.combat | Control (Ref) vs 22q11.2Duplication | 0.017 | 0.260 | 0.067 | 0.947 | 0.966 | >23years |
| ODI.CGC.combat | Control (Ref) vs 22q11.2Duplication | 0.363 | 0.252 | 1.437 | 0.152 | 0.265 | >23years |
| ODI.CGH.combat | Control (Ref) vs 22q11.2Duplication | -0.151 | 0.262 | -0.576 | 0.565 | 0.678 | >23years |
| ODI.CR.combat | Control (Ref) vs 22q11.2Duplication | -0.344 | 0.261 | -1.320 | 0.188 | 0.311 | >23years |
| ODI.CST.combat | Control (Ref) vs 22q11.2Duplication | 0.375 | 0.260 | 1.445 | 0.150 | 0.264 | >23years |
| ODI.EC.combat | Control (Ref) vs 22q11.2Duplication | 0.129 | 0.249 | 0.519 | 0.604 | 0.714 | >23years |
| ODI.FX.combat | Control (Ref) vs 22q11.2Duplication | 0.268 | 0.246 | 1.091 | 0.276 | 0.425 | >23years |
| ODI.FXST.combat | Control (Ref) vs 22q11.2Duplication | 0.154 | 0.245 | 0.630 | 0.529 | 0.655 | >23years |
| ODI.GCC.combat | Control (Ref) vs 22q11.2Duplication | -0.158 | 0.260 | -0.607 | 0.545 | 0.667 | >23years |
| ODI.IC.combat | Control (Ref) vs 22q11.2Duplication | 0.266 | 0.248 | 1.070 | 0.285 | 0.435 | >23years |
| ODI.UNC.combat | Control (Ref) vs 22q11.2Duplication | 0.092 | 0.260 | 0.354 | 0.723 | 0.801 | >23years |
| ODI.PCR.combat | Control (Ref) vs 22q11.2Duplication | -0.675 | 0.260 | -2.599 | 0.010 | 0.036 | >23years |
| ODI.PLIC.combat | Control (Ref) vs 22q11.2Duplication | 0.386 | 0.240 | 1.605 | 0.110 | 0.213 | >23years |
| ODI.PTR.combat | Control (Ref) vs 22q11.2Duplication | -0.122 | 0.268 | -0.454 | 0.650 | 0.744 | >23years |
| ODI.RLIC.combat | Control (Ref) vs 22q11.2Duplication | 0.014 | 0.257 | 0.054 | 0.957 | 0.970 | >23years |
| ODI.SCC.combat | Control (Ref) vs 22q11.2Duplication | 0.062 | 0.264 | 0.234 | 0.815 | 0.864 | >23years |
| ODI.SCR.combat | Control (Ref) vs 22q11.2Duplication | -0.318 | 0.262 | -1.213 | 0.226 | 0.359 | >23years |
| ODI.SFO.combat | Control (Ref) vs 22q11.2Duplication | -0.050 | 0.256 | -0.195 | 0.846 | 0.879 | >23years |
| ODI.SLF.combat | Control (Ref) vs 22q11.2Duplication | -0.229 | 0.266 | -0.859 | 0.391 | 0.541 | >23years |
| ODI.SS.combat | Control (Ref) vs 22q11.2Duplication | -0.120 | 0.264 | -0.454 | 0.650 | 0.744 | >23years |
| ODI.TAP.combat | Control (Ref) vs 22q11.2Duplication | 0.119 | 0.269 | 0.442 | 0.659 | 0.748 | >23years |
| ICVF.combat | 22q11.2Deletion (Ref) vs 22q11.2Duplication | -1.235 | 0.250 | -4.935 | 0.000 | 0.000 | >23years |
| ISO.combat | 22q11.2Deletion (Ref) vs 22q11.2Duplication | -1.070 | 0.334 | -3.207 | 0.003 | 0.015 | >23years |
| ODI.combat | 22q11.2Deletion (Ref) vs 22q11.2Duplication | -0.052 | 0.370 | -0.141 | 0.889 | 0.916 | >23years |
| ICVF.ACR.combat | 22q11.2Deletion (Ref) vs 22q11.2Duplication | -1.279 | 0.245 | -5.228 | 0.000 | 0.000 | >23years |
| ICVF.ALIC.combat | 22q11.2Deletion (Ref) vs 22q11.2Duplication | -0.739 | 0.328 | -2.255 | 0.031 | 0.076 | >23years |
| ICVF.BCC.combat | 22q11.2Deletion (Ref) vs 22q11.2Duplication | -1.396 | 0.208 | -6.717 | 0.000 | 0.000 | >23years |
| ICVF.CC.combat | 22q11.2Deletion (Ref) vs 22q11.2Duplication | -1.401 | 0.218 | -6.427 | 0.000 | 0.000 | >23years |
| ICVF.CGC.combat | 22q11.2Deletion (Ref) vs 22q11.2Duplication | -1.234 | 0.249 | -4.962 | 0.000 | 0.000 | >23years |
| ICVF.CGH.combat | 22q11.2Deletion (Ref) vs 22q11.2Duplication | -0.590 | 0.343 | -1.720 | 0.095 | 0.191 | >23years |
| ICVF.CR.combat | 22q11.2Deletion (Ref) vs 22q11.2Duplication | -1.275 | 0.248 | -5.138 | 0.000 | 0.000 | >23years |
| ICVF.CST.combat | 22q11.2Deletion (Ref) vs 22q11.2Duplication | -0.353 | 0.416 | -0.850 | 0.402 | 0.548 | >23years |
| ICVF.EC.combat | 22q11.2Deletion (Ref) vs 22q11.2Duplication | -0.953 | 0.263 | -3.618 | 0.001 | 0.006 | >23years |
| ICVF.FX.combat | 22q11.2Deletion (Ref) vs 22q11.2Duplication | -0.299 | 0.338 | -0.883 | 0.384 | 0.535 | >23years |
| ICVF.FXST.combat | 22q11.2Deletion (Ref) vs 22q11.2Duplication | -0.634 | 0.345 | -1.836 | 0.076 | 0.161 | >23years |
| ICVF.GCC.combat | 22q11.2Deletion (Ref) vs 22q11.2Duplication | -1.386 | 0.215 | -6.448 | 0.000 | 0.000 | >23years |
| ICVF.IC.combat | 22q11.2Deletion (Ref) vs 22q11.2Duplication | -0.959 | 0.316 | -3.031 | 0.005 | 0.021 | >23years |
| ICVF.UNC.combat | 22q11.2Deletion (Ref) vs 22q11.2Duplication | -1.429 | 0.224 | -6.383 | 0.000 | 0.000 | >23years |
| ICVF.PCR.combat | 22q11.2Deletion (Ref) vs 22q11.2Duplication | -1.274 | 0.256 | -4.974 | 0.000 | 0.000 | >23years |
| ICVF.PLIC.combat | 22q11.2Deletion (Ref) vs 22q11.2Duplication | -0.829 | 0.337 | -2.462 | 0.020 | 0.056 | >23years |
| ICVF.PTR.combat | 22q11.2Deletion (Ref) vs 22q11.2Duplication | -1.356 | 0.255 | -5.320 | 0.000 | 0.000 | >23years |
| ICVF.RLIC.combat | 22q11.2Deletion (Ref) vs 22q11.2Duplication | -1.152 | 0.285 | -4.035 | 0.000 | 0.002 | >23years |
| ICVF.SCC.combat | 22q11.2Deletion (Ref) vs 22q11.2Duplication | -1.284 | 0.284 | -4.527 | 0.000 | 0.001 | >23years |
| ICVF.SCR.combat | 22q11.2Deletion (Ref) vs 22q11.2Duplication | -1.285 | 0.257 | -4.997 | 0.000 | 0.000 | >23years |
| ICVF.SFO.combat | 22q11.2Deletion (Ref) vs 22q11.2Duplication | -0.942 | 0.310 | -3.035 | 0.005 | 0.021 | >23years |
| ICVF.SLF.combat | 22q11.2Deletion (Ref) vs 22q11.2Duplication | -1.358 | 0.213 | -6.361 | 0.000 | 0.000 | >23years |
| ICVF.SS.combat | 22q11.2Deletion (Ref) vs 22q11.2Duplication | -1.176 | 0.262 | -4.491 | 0.000 | 0.001 | >23years |
| ICVF.TAP.combat | 22q11.2Deletion (Ref) vs 22q11.2Duplication | -1.204 | 0.316 | -3.807 | 0.001 | 0.004 | >23years |
| ISO.ACR.combat | 22q11.2Deletion (Ref) vs 22q11.2Duplication | -0.869 | 0.352 | -2.467 | 0.019 | 0.056 | >23years |
| ISO.ALIC.combat | 22q11.2Deletion (Ref) vs 22q11.2Duplication | -0.221 | 0.334 | -0.660 | 0.514 | 0.644 | >23years |
| ISO.BCC.combat | 22q11.2Deletion (Ref) vs 22q11.2Duplication | -0.478 | 0.429 | -1.113 | 0.274 | 0.424 | >23years |
| ISO.CC.combat | 22q11.2Deletion (Ref) vs 22q11.2Duplication | -0.300 | 0.435 | -0.689 | 0.496 | 0.625 | >23years |
| ISO.CGC.combat | 22q11.2Deletion (Ref) vs 22q11.2Duplication | -0.178 | 0.419 | -0.426 | 0.673 | 0.758 | >23years |
| ISO.CGH.combat | 22q11.2Deletion (Ref) vs 22q11.2Duplication | -0.577 | 0.362 | -1.596 | 0.121 | 0.221 | >23years |
| ISO.CR.combat | 22q11.2Deletion (Ref) vs 22q11.2Duplication | -0.986 | 0.350 | -2.819 | 0.008 | 0.033 | >23years |
| ISO.CST.combat | 22q11.2Deletion (Ref) vs 22q11.2Duplication | 0.540 | 0.313 | 1.724 | 0.096 | 0.191 | >23years |
| ISO.EC.combat | 22q11.2Deletion (Ref) vs 22q11.2Duplication | -0.330 | 0.341 | -0.968 | 0.340 | 0.501 | >23years |
| ISO.FX.combat | 22q11.2Deletion (Ref) vs 22q11.2Duplication | -0.365 | 0.395 | -0.924 | 0.363 | 0.516 | >23years |
| ISO.FXST.combat | 22q11.2Deletion (Ref) vs 22q11.2Duplication | -0.146 | 0.354 | -0.412 | 0.683 | 0.765 | >23years |
| ISO.GCC.combat | 22q11.2Deletion (Ref) vs 22q11.2Duplication | -0.335 | 0.423 | -0.792 | 0.434 | 0.571 | >23years |
| ISO.IC.combat | 22q11.2Deletion (Ref) vs 22q11.2Duplication | -0.506 | 0.350 | -1.445 | 0.159 | 0.272 | >23years |
| ISO.UNC.combat | 22q11.2Deletion (Ref) vs 22q11.2Duplication | -0.307 | 0.414 | -0.742 | 0.464 | 0.597 | >23years |
| ISO.PCR.combat | 22q11.2Deletion (Ref) vs 22q11.2Duplication | -1.009 | 0.339 | -2.973 | 0.006 | 0.023 | >23years |
| ISO.PLIC.combat | 22q11.2Deletion (Ref) vs 22q11.2Duplication | -0.272 | 0.413 | -0.659 | 0.515 | 0.644 | >23years |
| ISO.PTR.combat | 22q11.2Deletion (Ref) vs 22q11.2Duplication | -1.130 | 0.299 | -3.778 | 0.001 | 0.004 | >23years |
| ISO.RLIC.combat | 22q11.2Deletion (Ref) vs 22q11.2Duplication | -0.699 | 0.341 | -2.049 | 0.049 | 0.112 | >23years |
| ISO.SCC.combat | 22q11.2Deletion (Ref) vs 22q11.2Duplication | 0.007 | 0.391 | 0.017 | 0.987 | 0.990 | >23years |
| ISO.SCR.combat | 22q11.2Deletion (Ref) vs 22q11.2Duplication | -0.981 | 0.365 | -2.688 | 0.011 | 0.038 | >23years |
| ISO.SFO.combat | 22q11.2Deletion (Ref) vs 22q11.2Duplication | -0.703 | 0.363 | -1.940 | 0.062 | 0.137 | >23years |
| ISO.SLF.combat | 22q11.2Deletion (Ref) vs 22q11.2Duplication | -1.136 | 0.297 | -3.820 | 0.001 | 0.004 | >23years |
| ISO.SS.combat | 22q11.2Deletion (Ref) vs 22q11.2Duplication | -0.473 | 0.328 | -1.439 | 0.161 | 0.274 | >23years |
| ISO.TAP.combat | 22q11.2Deletion (Ref) vs 22q11.2Duplication | -0.779 | 0.316 | -2.465 | 0.020 | 0.056 | >23years |
| ODI.ACR.combat | 22q11.2Deletion (Ref) vs 22q11.2Duplication | -0.425 | 0.391 | -1.089 | 0.285 | 0.435 | >23years |
| ODI.ALIC.combat | 22q11.2Deletion (Ref) vs 22q11.2Duplication | 0.767 | 0.335 | 2.291 | 0.029 | 0.073 | >23years |
| ODI.BCC.combat | 22q11.2Deletion (Ref) vs 22q11.2Duplication | -0.310 | 0.392 | -0.789 | 0.436 | 0.571 | >23years |
| ODI.CC.combat | 22q11.2Deletion (Ref) vs 22q11.2Duplication | -0.375 | 0.401 | -0.935 | 0.357 | 0.515 | >23years |
| ODI.CGC.combat | 22q11.2Deletion (Ref) vs 22q11.2Duplication | 0.418 | 0.388 | 1.077 | 0.290 | 0.439 | >23years |
| ODI.CGH.combat | 22q11.2Deletion (Ref) vs 22q11.2Duplication | -0.615 | 0.382 | -1.611 | 0.117 | 0.220 | >23years |
| ODI.CR.combat | 22q11.2Deletion (Ref) vs 22q11.2Duplication | -0.497 | 0.388 | -1.279 | 0.210 | 0.340 | >23years |
| ODI.CST.combat | 22q11.2Deletion (Ref) vs 22q11.2Duplication | 0.626 | 0.342 | 1.830 | 0.077 | 0.161 | >23years |
| ODI.EC.combat | 22q11.2Deletion (Ref) vs 22q11.2Duplication | -0.495 | 0.387 | -1.279 | 0.210 | 0.340 | >23years |
| ODI.FX.combat | 22q11.2Deletion (Ref) vs 22q11.2Duplication | 0.062 | 0.389 | 0.160 | 0.874 | 0.904 | >23years |
| ODI.FXST.combat | 22q11.2Deletion (Ref) vs 22q11.2Duplication | -0.098 | 0.412 | -0.238 | 0.814 | 0.864 | >23years |
| ODI.GCC.combat | 22q11.2Deletion (Ref) vs 22q11.2Duplication | -0.263 | 0.414 | -0.635 | 0.530 | 0.655 | >23years |
| ODI.IC.combat | 22q11.2Deletion (Ref) vs 22q11.2Duplication | 0.504 | 0.369 | 1.365 | 0.182 | 0.303 | >23years |
| ODI.UNC.combat | 22q11.2Deletion (Ref) vs 22q11.2Duplication | -0.090 | 0.443 | -0.204 | 0.840 | 0.879 | >23years |
| ODI.PCR.combat | 22q11.2Deletion (Ref) vs 22q11.2Duplication | -1.065 | 0.335 | -3.184 | 0.003 | 0.015 | >23years |
| ODI.PLIC.combat | 22q11.2Deletion (Ref) vs 22q11.2Duplication | 0.762 | 0.362 | 2.105 | 0.043 | 0.101 | >23years |
| ODI.PTR.combat | 22q11.2Deletion (Ref) vs 22q11.2Duplication | -1.178 | 0.371 | -3.172 | 0.003 | 0.015 | >23years |
| ODI.RLIC.combat | 22q11.2Deletion (Ref) vs 22q11.2Duplication | -0.116 | 0.403 | -0.288 | 0.775 | 0.842 | >23years |
| ODI.SCC.combat | 22q11.2Deletion (Ref) vs 22q11.2Duplication | -0.402 | 0.399 | -1.009 | 0.321 | 0.479 | >23years |
| ODI.SCR.combat | 22q11.2Deletion (Ref) vs 22q11.2Duplication | -0.208 | 0.397 | -0.525 | 0.604 | 0.714 | >23years |
| ODI.SFO.combat | 22q11.2Deletion (Ref) vs 22q11.2Duplication | -0.523 | 0.407 | -1.284 | 0.209 | 0.340 | >23years |
| ODI.SLF.combat | 22q11.2Deletion (Ref) vs 22q11.2Duplication | -0.602 | 0.404 | -1.490 | 0.146 | 0.262 | >23years |
| ODI.SS.combat | 22q11.2Deletion (Ref) vs 22q11.2Duplication | -0.648 | 0.380 | -1.703 | 0.099 | 0.195 | >23years |
| ODI.TAP.combat | 22q11.2Deletion (Ref) vs 22q11.2Duplication | 0.113 | 0.349 | 0.322 | 0.749 | 0.826 | >23years |
| ICVF.combat | GeneDosage (1 = del, 2 = con, 3 dup) | -0.824 | 0.208 | -3.964 | 0.000 | 0.001 | >23years |
| ISO.combat | GeneDosage (1 = del, 2 = con, 3 dup) | -0.406 | 0.233 | -1.744 | 0.082 | 0.169 | >23years |
| ODI.combat | GeneDosage (1 = del, 2 = con, 3 dup) | -0.130 | 0.229 | -0.567 | 0.571 | 0.683 | >23years |
| ICVF.ACR.combat | GeneDosage (1 = del, 2 = con, 3 dup) | -0.799 | 0.206 | -3.883 | 0.000 | 0.001 | >23years |
| ICVF.ALIC.combat | GeneDosage (1 = del, 2 = con, 3 dup) | -0.414 | 0.190 | -2.182 | 0.030 | 0.074 | >23years |
| ICVF.BCC.combat | GeneDosage (1 = del, 2 = con, 3 dup) | -1.108 | 0.197 | -5.616 | 0.000 | 0.000 | >23years |
| ICVF.CC.combat | GeneDosage (1 = del, 2 = con, 3 dup) | -1.064 | 0.199 | -5.352 | 0.000 | 0.000 | >23years |
| ICVF.CGC.combat | GeneDosage (1 = del, 2 = con, 3 dup) | -0.650 | 0.206 | -3.158 | 0.002 | 0.009 | >23years |
| ICVF.CGH.combat | GeneDosage (1 = del, 2 = con, 3 dup) | -0.285 | 0.204 | -1.394 | 0.164 | 0.277 | >23years |
| ICVF.CR.combat | GeneDosage (1 = del, 2 = con, 3 dup) | -0.905 | 0.204 | -4.428 | 0.000 | 0.000 | >23years |
| ICVF.CST.combat | GeneDosage (1 = del, 2 = con, 3 dup) | -0.167 | 0.208 | -0.802 | 0.423 | 0.564 | >23years |
| ICVF.EC.combat | GeneDosage (1 = del, 2 = con, 3 dup) | -0.485 | 0.211 | -2.297 | 0.022 | 0.060 | >23years |
| ICVF.FX.combat | GeneDosage (1 = del, 2 = con, 3 dup) | -0.255 | 0.212 | -1.207 | 0.229 | 0.361 | >23years |
| ICVF.FXST.combat | GeneDosage (1 = del, 2 = con, 3 dup) | -0.525 | 0.209 | -2.516 | 0.012 | 0.041 | >23years |
| ICVF.GCC.combat | GeneDosage (1 = del, 2 = con, 3 dup) | -0.947 | 0.200 | -4.737 | 0.000 | 0.000 | >23years |
| ICVF.IC.combat | GeneDosage (1 = del, 2 = con, 3 dup) | -0.584 | 0.200 | -2.922 | 0.004 | 0.017 | >23years |
| ICVF.UNC.combat | GeneDosage (1 = del, 2 = con, 3 dup) | -0.823 | 0.211 | -3.904 | 0.000 | 0.001 | >23years |
| ICVF.PCR.combat | GeneDosage (1 = del, 2 = con, 3 dup) | -1.073 | 0.201 | -5.350 | 0.000 | 0.000 | >23years |
| ICVF.PLIC.combat | GeneDosage (1 = del, 2 = con, 3 dup) | -0.457 | 0.201 | -2.280 | 0.023 | 0.061 | >23years |
| ICVF.PTR.combat | GeneDosage (1 = del, 2 = con, 3 dup) | -1.000 | 0.203 | -4.926 | 0.000 | 0.000 | >23years |
| ICVF.RLIC.combat | GeneDosage (1 = del, 2 = con, 3 dup) | -0.772 | 0.208 | -3.713 | 0.000 | 0.002 | >23years |
| ICVF.SCC.combat | GeneDosage (1 = del, 2 = con, 3 dup) | -0.959 | 0.205 | -4.672 | 0.000 | 0.000 | >23years |
| ICVF.SCR.combat | GeneDosage (1 = del, 2 = con, 3 dup) | -0.862 | 0.207 | -4.166 | 0.000 | 0.000 | >23years |
| ICVF.SFO.combat | GeneDosage (1 = del, 2 = con, 3 dup) | -0.548 | 0.209 | -2.624 | 0.009 | 0.034 | >23years |
| ICVF.SLF.combat | GeneDosage (1 = del, 2 = con, 3 dup) | -0.928 | 0.206 | -4.499 | 0.000 | 0.000 | >23years |
| ICVF.SS.combat | GeneDosage (1 = del, 2 = con, 3 dup) | -0.916 | 0.204 | -4.479 | 0.000 | 0.000 | >23years |
| ICVF.TAP.combat | GeneDosage (1 = del, 2 = con, 3 dup) | -1.081 | 0.196 | -5.516 | 0.000 | 0.000 | >23years |
| ISO.ACR.combat | GeneDosage (1 = del, 2 = con, 3 dup) | -0.286 | 0.233 | -1.227 | 0.221 | 0.352 | >23years |
| ISO.ALIC.combat | GeneDosage (1 = del, 2 = con, 3 dup) | -0.056 | 0.218 | -0.257 | 0.797 | 0.860 | >23years |
| ISO.BCC.combat | GeneDosage (1 = del, 2 = con, 3 dup) | -0.101 | 0.229 | -0.444 | 0.658 | 0.748 | >23years |
| ISO.CC.combat | GeneDosage (1 = del, 2 = con, 3 dup) | 0.044 | 0.228 | 0.193 | 0.847 | 0.879 | >23years |
| ISO.CGC.combat | GeneDosage (1 = del, 2 = con, 3 dup) | 0.107 | 0.209 | 0.512 | 0.609 | 0.714 | >23years |
| ISO.CGH.combat | GeneDosage (1 = del, 2 = con, 3 dup) | -0.170 | 0.195 | -0.874 | 0.383 | 0.535 | >23years |
| ISO.CR.combat | GeneDosage (1 = del, 2 = con, 3 dup) | -0.432 | 0.231 | -1.872 | 0.062 | 0.137 | >23years |
| ISO.CST.combat | GeneDosage (1 = del, 2 = con, 3 dup) | 0.202 | 0.216 | 0.939 | 0.349 | 0.510 | >23years |
| ISO.EC.combat | GeneDosage (1 = del, 2 = con, 3 dup) | -0.003 | 0.214 | -0.012 | 0.990 | 0.990 | >23years |
| ISO.FX.combat | GeneDosage (1 = del, 2 = con, 3 dup) | -0.254 | 0.214 | -1.184 | 0.237 | 0.373 | >23years |
| ISO.FXST.combat | GeneDosage (1 = del, 2 = con, 3 dup) | 0.123 | 0.210 | 0.587 | 0.557 | 0.674 | >23years |
| ISO.GCC.combat | GeneDosage (1 = del, 2 = con, 3 dup) | 0.097 | 0.224 | 0.433 | 0.665 | 0.753 | >23years |
| ISO.IC.combat | GeneDosage (1 = del, 2 = con, 3 dup) | -0.186 | 0.206 | -0.902 | 0.368 | 0.520 | >23years |
| ISO.UNC.combat | GeneDosage (1 = del, 2 = con, 3 dup) | -0.016 | 0.216 | -0.073 | 0.941 | 0.964 | >23years |
| ISO.PCR.combat | GeneDosage (1 = del, 2 = con, 3 dup) | -0.335 | 0.215 | -1.558 | 0.120 | 0.221 | >23years |
| ISO.PLIC.combat | GeneDosage (1 = del, 2 = con, 3 dup) | -0.041 | 0.212 | -0.193 | 0.847 | 0.879 | >23years |
| ISO.PTR.combat | GeneDosage (1 = del, 2 = con, 3 dup) | -0.390 | 0.217 | -1.793 | 0.074 | 0.158 | >23years |
| ISO.RLIC.combat | GeneDosage (1 = del, 2 = con, 3 dup) | -0.301 | 0.208 | -1.452 | 0.148 | 0.262 | >23years |
| ISO.SCC.combat | GeneDosage (1 = del, 2 = con, 3 dup) | 0.180 | 0.220 | 0.818 | 0.414 | 0.555 | >23years |
| ISO.SCR.combat | GeneDosage (1 = del, 2 = con, 3 dup) | -0.563 | 0.227 | -2.483 | 0.014 | 0.044 | >23years |
| ISO.SFO.combat | GeneDosage (1 = del, 2 = con, 3 dup) | -0.325 | 0.232 | -1.402 | 0.162 | 0.275 | >23years |
| ISO.SLF.combat | GeneDosage (1 = del, 2 = con, 3 dup) | -0.496 | 0.229 | -2.166 | 0.031 | 0.076 | >23years |
| ISO.SS.combat | GeneDosage (1 = del, 2 = con, 3 dup) | -0.090 | 0.214 | -0.420 | 0.675 | 0.758 | >23years |
| ISO.TAP.combat | GeneDosage (1 = del, 2 = con, 3 dup) | -0.504 | 0.220 | -2.293 | 0.023 | 0.060 | >23years |
| ODI.ACR.combat | GeneDosage (1 = del, 2 = con, 3 dup) | -0.159 | 0.231 | -0.687 | 0.492 | 0.623 | >23years |
| ODI.ALIC.combat | GeneDosage (1 = del, 2 = con, 3 dup) | 0.554 | 0.228 | 2.435 | 0.016 | 0.048 | >23years |
| ODI.BCC.combat | GeneDosage (1 = del, 2 = con, 3 dup) | -0.377 | 0.241 | -1.563 | 0.119 | 0.221 | >23years |
| ODI.CC.combat | GeneDosage (1 = del, 2 = con, 3 dup) | -0.365 | 0.232 | -1.570 | 0.118 | 0.220 | >23years |
| ODI.CGC.combat | GeneDosage (1 = del, 2 = con, 3 dup) | 0.163 | 0.215 | 0.760 | 0.448 | 0.584 | >23years |
| ODI.CGH.combat | GeneDosage (1 = del, 2 = con, 3 dup) | -0.572 | 0.216 | -2.655 | 0.008 | 0.033 | >23years |
| ODI.CR.combat | GeneDosage (1 = del, 2 = con, 3 dup) | -0.291 | 0.235 | -1.238 | 0.217 | 0.348 | >23years |
| ODI.CST.combat | GeneDosage (1 = del, 2 = con, 3 dup) | 0.531 | 0.208 | 2.560 | 0.011 | 0.037 | >23years |
| ODI.EC.combat | GeneDosage (1 = del, 2 = con, 3 dup) | -0.445 | 0.208 | -2.137 | 0.033 | 0.080 | >23years |
| ODI.FX.combat | GeneDosage (1 = del, 2 = con, 3 dup) | -0.060 | 0.208 | -0.291 | 0.771 | 0.842 | >23years |
| ODI.FXST.combat | GeneDosage (1 = del, 2 = con, 3 dup) | -0.131 | 0.213 | -0.612 | 0.541 | 0.665 | >23years |
| ODI.GCC.combat | GeneDosage (1 = del, 2 = con, 3 dup) | -0.167 | 0.231 | -0.724 | 0.469 | 0.599 | >23years |
| ODI.IC.combat | GeneDosage (1 = del, 2 = con, 3 dup) | 0.402 | 0.201 | 2.001 | 0.046 | 0.106 | >23years |
| ODI.UNC.combat | GeneDosage (1 = del, 2 = con, 3 dup) | -0.213 | 0.217 | -0.981 | 0.327 | 0.486 | >23years |
| ODI.PCR.combat | GeneDosage (1 = del, 2 = con, 3 dup) | -0.696 | 0.213 | -3.274 | 0.001 | 0.006 | >23years |
| ODI.PLIC.combat | GeneDosage (1 = del, 2 = con, 3 dup) | 0.559 | 0.200 | 2.798 | 0.005 | 0.023 | >23years |
| ODI.PTR.combat | GeneDosage (1 = del, 2 = con, 3 dup) | -0.771 | 0.215 | -3.589 | 0.000 | 0.002 | >23years |
| ODI.RLIC.combat | GeneDosage (1 = del, 2 = con, 3 dup) | -0.047 | 0.216 | -0.218 | 0.828 | 0.872 | >23years |
| ODI.SCC.combat | GeneDosage (1 = del, 2 = con, 3 dup) | -0.331 | 0.218 | -1.516 | 0.131 | 0.235 | >23years |
| ODI.SCR.combat | GeneDosage (1 = del, 2 = con, 3 dup) | -0.068 | 0.237 | -0.289 | 0.773 | 0.842 | >23years |
| ODI.SFO.combat | GeneDosage (1 = del, 2 = con, 3 dup) | -0.521 | 0.214 | -2.439 | 0.015 | 0.048 | >23years |
| ODI.SLF.combat | GeneDosage (1 = del, 2 = con, 3 dup) | -0.565 | 0.217 | -2.609 | 0.010 | 0.035 | >23years |
| ODI.SS.combat | GeneDosage (1 = del, 2 = con, 3 dup) | -0.386 | 0.241 | -1.600 | 0.111 | 0.213 | >23years |
| ODI.TAP.combat | GeneDosage (1 = del, 2 = con, 3 dup) | -0.009 | 0.234 | -0.039 | 0.969 | 0.979 | >23years |
| ICVF.combat | Control (Ref) vs 22q11.2Deletion | 0.877 | 0.316 | 2.778 | 0.006 | 0.028 | >30years |
| ISO.combat | Control (Ref) vs 22q11.2Deletion | 0.084 | 0.318 | 0.263 | 0.793 | 0.853 | >30years |
| ODI.combat | Control (Ref) vs 22q11.2Deletion | 0.239 | 0.296 | 0.808 | 0.420 | 0.555 | >30years |
| ICVF.ACR.combat | Control (Ref) vs 22q11.2Deletion | 0.927 | 0.313 | 2.965 | 0.003 | 0.018 | >30years |
| ICVF.ALIC.combat | Control (Ref) vs 22q11.2Deletion | 0.502 | 0.284 | 1.769 | 0.078 | 0.168 | >30years |
| ICVF.BCC.combat | Control (Ref) vs 22q11.2Deletion | 1.023 | 0.311 | 3.292 | 0.001 | 0.009 | >30years |
| ICVF.CC.combat | Control (Ref) vs 22q11.2Deletion | 1.047 | 0.309 | 3.386 | 0.001 | 0.007 | >30years |
| ICVF.CGC.combat | Control (Ref) vs 22q11.2Deletion | 0.597 | 0.311 | 1.919 | 0.056 | 0.136 | >30years |
| ICVF.CGH.combat | Control (Ref) vs 22q11.2Deletion | -0.013 | 0.304 | -0.042 | 0.967 | 0.977 | >30years |
| ICVF.CR.combat | Control (Ref) vs 22q11.2Deletion | 0.938 | 0.313 | 2.995 | 0.003 | 0.017 | >30years |
| ICVF.CST.combat | Control (Ref) vs 22q11.2Deletion | 0.563 | 0.302 | 1.864 | 0.064 | 0.150 | >30years |
| ICVF.EC.combat | Control (Ref) vs 22q11.2Deletion | 0.696 | 0.313 | 2.226 | 0.027 | 0.078 | >30years |
| ICVF.FX.combat | Control (Ref) vs 22q11.2Deletion | 0.186 | 0.319 | 0.584 | 0.560 | 0.677 | >30years |
| ICVF.FXST.combat | Control (Ref) vs 22q11.2Deletion | 0.739 | 0.310 | 2.380 | 0.018 | 0.061 | >30years |
| ICVF.GCC.combat | Control (Ref) vs 22q11.2Deletion | 0.905 | 0.307 | 2.942 | 0.004 | 0.019 | >30years |
| ICVF.IC.combat | Control (Ref) vs 22q11.2Deletion | 0.745 | 0.287 | 2.593 | 0.010 | 0.041 | >30years |
| ICVF.UNC.combat | Control (Ref) vs 22q11.2Deletion | 1.243 | 0.309 | 4.022 | 0.000 | 0.002 | >30years |
| ICVF.PCR.combat | Control (Ref) vs 22q11.2Deletion | 1.060 | 0.313 | 3.389 | 0.001 | 0.007 | >30years |
| ICVF.PLIC.combat | Control (Ref) vs 22q11.2Deletion | 0.617 | 0.290 | 2.130 | 0.034 | 0.091 | >30years |
| ICVF.PTR.combat | Control (Ref) vs 22q11.2Deletion | 0.938 | 0.309 | 3.037 | 0.003 | 0.016 | >30years |
| ICVF.RLIC.combat | Control (Ref) vs 22q11.2Deletion | 0.986 | 0.313 | 3.150 | 0.002 | 0.012 | >30years |
| ICVF.SCC.combat | Control (Ref) vs 22q11.2Deletion | 1.067 | 0.312 | 3.420 | 0.001 | 0.007 | >30years |
| ICVF.SCR.combat | Control (Ref) vs 22q11.2Deletion | 0.780 | 0.315 | 2.481 | 0.014 | 0.049 | >30years |
| ICVF.SFO.combat | Control (Ref) vs 22q11.2Deletion | 0.505 | 0.296 | 1.705 | 0.089 | 0.189 | >30years |
| ICVF.SLF.combat | Control (Ref) vs 22q11.2Deletion | 0.787 | 0.316 | 2.490 | 0.013 | 0.049 | >30years |
| ICVF.SS.combat | Control (Ref) vs 22q11.2Deletion | 0.892 | 0.313 | 2.849 | 0.005 | 0.023 | >30years |
| ICVF.TAP.combat | Control (Ref) vs 22q11.2Deletion | 1.201 | 0.300 | 4.004 | 0.000 | 0.002 | >30years |
| ISO.ACR.combat | Control (Ref) vs 22q11.2Deletion | -0.129 | 0.322 | -0.399 | 0.690 | 0.781 | >30years |
| ISO.ALIC.combat | Control (Ref) vs 22q11.2Deletion | -0.157 | 0.320 | -0.490 | 0.624 | 0.726 | >30years |
| ISO.BCC.combat | Control (Ref) vs 22q11.2Deletion | -0.112 | 0.319 | -0.350 | 0.727 | 0.813 | >30years |
| ISO.CC.combat | Control (Ref) vs 22q11.2Deletion | -0.242 | 0.320 | -0.756 | 0.450 | 0.577 | >30years |
| ISO.CGC.combat | Control (Ref) vs 22q11.2Deletion | 0.004 | 0.309 | 0.014 | 0.989 | 0.989 | >30years |
| ISO.CGH.combat | Control (Ref) vs 22q11.2Deletion | 0.512 | 0.298 | 1.716 | 0.087 | 0.186 | >30years |
| ISO.CR.combat | Control (Ref) vs 22q11.2Deletion | -0.047 | 0.322 | -0.146 | 0.884 | 0.921 | >30years |
| ISO.CST.combat | Control (Ref) vs 22q11.2Deletion | -0.535 | 0.319 | -1.679 | 0.094 | 0.197 | >30years |
| ISO.EC.combat | Control (Ref) vs 22q11.2Deletion | 0.072 | 0.317 | 0.228 | 0.820 | 0.872 | >30years |
| ISO.FX.combat | Control (Ref) vs 22q11.2Deletion | 0.314 | 0.313 | 1.004 | 0.316 | 0.461 | >30years |
| ISO.FXST.combat | Control (Ref) vs 22q11.2Deletion | -0.457 | 0.297 | -1.536 | 0.126 | 0.244 | >30years |
| ISO.GCC.combat | Control (Ref) vs 22q11.2Deletion | -0.327 | 0.319 | -1.026 | 0.306 | 0.454 | >30years |
| ISO.IC.combat | Control (Ref) vs 22q11.2Deletion | 0.272 | 0.317 | 0.856 | 0.393 | 0.538 | >30years |
| ISO.UNC.combat | Control (Ref) vs 22q11.2Deletion | -0.209 | 0.317 | -0.659 | 0.510 | 0.641 | >30years |
| ISO.PCR.combat | Control (Ref) vs 22q11.2Deletion | -0.026 | 0.321 | -0.082 | 0.935 | 0.954 | >30years |
| ISO.PLIC.combat | Control (Ref) vs 22q11.2Deletion | 0.398 | 0.314 | 1.266 | 0.207 | 0.342 | >30years |
| ISO.PTR.combat | Control (Ref) vs 22q11.2Deletion | 0.038 | 0.318 | 0.119 | 0.905 | 0.937 | >30years |
| ISO.RLIC.combat | Control (Ref) vs 22q11.2Deletion | 0.232 | 0.316 | 0.736 | 0.462 | 0.589 | >30years |
| ISO.SCC.combat | Control (Ref) vs 22q11.2Deletion | -0.204 | 0.320 | -0.638 | 0.524 | 0.653 | >30years |
| ISO.SCR.combat | Control (Ref) vs 22q11.2Deletion | 0.097 | 0.318 | 0.306 | 0.760 | 0.826 | >30years |
| ISO.SFO.combat | Control (Ref) vs 22q11.2Deletion | 0.080 | 0.316 | 0.253 | 0.801 | 0.858 | >30years |
| ISO.SLF.combat | Control (Ref) vs 22q11.2Deletion | -0.101 | 0.318 | -0.318 | 0.751 | 0.822 | >30years |
| ISO.SS.combat | Control (Ref) vs 22q11.2Deletion | -0.134 | 0.320 | -0.418 | 0.676 | 0.771 | >30years |
| ISO.TAP.combat | Control (Ref) vs 22q11.2Deletion | 0.011 | 0.319 | 0.033 | 0.974 | 0.977 | >30years |
| ODI.ACR.combat | Control (Ref) vs 22q11.2Deletion | 0.477 | 0.314 | 1.520 | 0.130 | 0.248 | >30years |
| ODI.ALIC.combat | Control (Ref) vs 22q11.2Deletion | -0.496 | 0.310 | -1.600 | 0.111 | 0.220 | >30years |
| ODI.BCC.combat | Control (Ref) vs 22q11.2Deletion | 0.391 | 0.312 | 1.252 | 0.212 | 0.347 | >30years |
| ODI.CC.combat | Control (Ref) vs 22q11.2Deletion | 0.446 | 0.311 | 1.434 | 0.153 | 0.273 | >30years |
| ODI.CGC.combat | Control (Ref) vs 22q11.2Deletion | 0.049 | 0.311 | 0.158 | 0.875 | 0.917 | >30years |
| ODI.CGH.combat | Control (Ref) vs 22q11.2Deletion | 0.900 | 0.308 | 2.919 | 0.004 | 0.020 | >30years |
| ODI.CR.combat | Control (Ref) vs 22q11.2Deletion | 0.429 | 0.312 | 1.375 | 0.170 | 0.294 | >30years |
| ODI.CST.combat | Control (Ref) vs 22q11.2Deletion | -0.305 | 0.316 | -0.965 | 0.335 | 0.476 | >30years |
| ODI.EC.combat | Control (Ref) vs 22q11.2Deletion | 0.674 | 0.295 | 2.284 | 0.023 | 0.072 | >30years |
| ODI.FX.combat | Control (Ref) vs 22q11.2Deletion | 0.438 | 0.294 | 1.492 | 0.137 | 0.251 | >30years |
| ODI.FXST.combat | Control (Ref) vs 22q11.2Deletion | 0.451 | 0.308 | 1.464 | 0.145 | 0.261 | >30years |
| ODI.GCC.combat | Control (Ref) vs 22q11.2Deletion | 0.212 | 0.315 | 0.675 | 0.501 | 0.634 | >30years |
| ODI.IC.combat | Control (Ref) vs 22q11.2Deletion | -0.182 | 0.308 | -0.593 | 0.554 | 0.675 | >30years |
| ODI.UNC.combat | Control (Ref) vs 22q11.2Deletion | 0.012 | 0.316 | 0.038 | 0.970 | 0.977 | >30years |
| ODI.PCR.combat | Control (Ref) vs 22q11.2Deletion | 0.728 | 0.313 | 2.326 | 0.021 | 0.067 | >30years |
| ODI.PLIC.combat | Control (Ref) vs 22q11.2Deletion | -0.359 | 0.304 | -1.181 | 0.239 | 0.381 | >30years |
| ODI.PTR.combat | Control (Ref) vs 22q11.2Deletion | 0.951 | 0.309 | 3.082 | 0.002 | 0.014 | >30years |
| ODI.RLIC.combat | Control (Ref) vs 22q11.2Deletion | 0.259 | 0.312 | 0.828 | 0.408 | 0.547 | >30years |
| ODI.SCC.combat | Control (Ref) vs 22q11.2Deletion | 0.563 | 0.311 | 1.810 | 0.071 | 0.162 | >30years |
| ODI.SCR.combat | Control (Ref) vs 22q11.2Deletion | 0.021 | 0.313 | 0.066 | 0.947 | 0.963 | >30years |
| ODI.SFO.combat | Control (Ref) vs 22q11.2Deletion | 0.336 | 0.308 | 1.091 | 0.276 | 0.422 | >30years |
| ODI.SLF.combat | Control (Ref) vs 22q11.2Deletion | 0.555 | 0.313 | 1.772 | 0.078 | 0.168 | >30years |
| ODI.SS.combat | Control (Ref) vs 22q11.2Deletion | 0.702 | 0.311 | 2.260 | 0.025 | 0.073 | >30years |
| ODI.TAP.combat | Control (Ref) vs 22q11.2Deletion | -0.060 | 0.317 | -0.188 | 0.851 | 0.899 | >30years |
| ICVF.combat | Control (Ref) vs 22q11.2Duplication | -0.480 | 0.262 | -1.830 | 0.068 | 0.158 | >30years |
| ISO.combat | Control (Ref) vs 22q11.2Duplication | -0.985 | 0.259 | -3.803 | 0.000 | 0.003 | >30years |
| ODI.combat | Control (Ref) vs 22q11.2Duplication | 0.083 | 0.249 | 0.334 | 0.739 | 0.817 | >30years |
| ICVF.ACR.combat | Control (Ref) vs 22q11.2Duplication | -0.428 | 0.265 | -1.614 | 0.108 | 0.216 | >30years |
| ICVF.ALIC.combat | Control (Ref) vs 22q11.2Duplication | -0.135 | 0.243 | -0.555 | 0.580 | 0.693 | >30years |
| ICVF.BCC.combat | Control (Ref) vs 22q11.2Duplication | -0.807 | 0.260 | -3.106 | 0.002 | 0.013 | >30years |
| ICVF.CC.combat | Control (Ref) vs 22q11.2Duplication | -0.741 | 0.261 | -2.839 | 0.005 | 0.023 | >30years |
| ICVF.CGC.combat | Control (Ref) vs 22q11.2Duplication | -0.436 | 0.259 | -1.686 | 0.093 | 0.195 | >30years |
| ICVF.CGH.combat | Control (Ref) vs 22q11.2Duplication | -0.304 | 0.250 | -1.217 | 0.225 | 0.361 | >30years |
| ICVF.CR.combat | Control (Ref) vs 22q11.2Duplication | -0.531 | 0.264 | -2.014 | 0.045 | 0.114 | >30years |
| ICVF.CST.combat | Control (Ref) vs 22q11.2Duplication | -0.043 | 0.254 | -0.169 | 0.866 | 0.911 | >30years |
| ICVF.EC.combat | Control (Ref) vs 22q11.2Duplication | -0.051 | 0.262 | -0.196 | 0.844 | 0.895 | >30years |
| ICVF.FX.combat | Control (Ref) vs 22q11.2Duplication | -0.204 | 0.267 | -0.764 | 0.445 | 0.576 | >30years |
| ICVF.FXST.combat | Control (Ref) vs 22q11.2Duplication | 0.026 | 0.261 | 0.098 | 0.922 | 0.944 | >30years |
| ICVF.GCC.combat | Control (Ref) vs 22q11.2Duplication | -0.671 | 0.260 | -2.581 | 0.010 | 0.041 | >30years |
| ICVF.IC.combat | Control (Ref) vs 22q11.2Duplication | -0.169 | 0.256 | -0.662 | 0.509 | 0.641 | >30years |
| ICVF.UNC.combat | Control (Ref) vs 22q11.2Duplication | -0.220 | 0.269 | -0.815 | 0.416 | 0.554 | >30years |
| ICVF.PCR.combat | Control (Ref) vs 22q11.2Duplication | -0.630 | 0.264 | -2.389 | 0.018 | 0.060 | >30years |
| ICVF.PLIC.combat | Control (Ref) vs 22q11.2Duplication | -0.163 | 0.260 | -0.626 | 0.532 | 0.656 | >30years |
| ICVF.PTR.combat | Control (Ref) vs 22q11.2Duplication | -0.635 | 0.264 | -2.409 | 0.017 | 0.058 | >30years |
| ICVF.RLIC.combat | Control (Ref) vs 22q11.2Duplication | -0.227 | 0.262 | -0.866 | 0.388 | 0.533 | >30years |
| ICVF.SCC.combat | Control (Ref) vs 22q11.2Duplication | -0.598 | 0.265 | -2.255 | 0.025 | 0.073 | >30years |
| ICVF.SCR.combat | Control (Ref) vs 22q11.2Duplication | -0.555 | 0.262 | -2.117 | 0.035 | 0.093 | >30years |
| ICVF.SFO.combat | Control (Ref) vs 22q11.2Duplication | -0.353 | 0.255 | -1.387 | 0.167 | 0.292 | >30years |
| ICVF.SLF.combat | Control (Ref) vs 22q11.2Duplication | -0.575 | 0.264 | -2.181 | 0.030 | 0.084 | >30years |
| ICVF.SS.combat | Control (Ref) vs 22q11.2Duplication | -0.379 | 0.269 | -1.410 | 0.160 | 0.283 | >30years |
| ICVF.TAP.combat | Control (Ref) vs 22q11.2Duplication | -0.579 | 0.261 | -2.222 | 0.027 | 0.078 | >30years |
| ISO.ACR.combat | Control (Ref) vs 22q11.2Duplication | -0.951 | 0.262 | -3.629 | 0.000 | 0.004 | >30years |
| ISO.ALIC.combat | Control (Ref) vs 22q11.2Duplication | -0.559 | 0.243 | -2.299 | 0.022 | 0.071 | >30years |
| ISO.BCC.combat | Control (Ref) vs 22q11.2Duplication | -0.683 | 0.262 | -2.610 | 0.010 | 0.039 | >30years |
| ISO.CC.combat | Control (Ref) vs 22q11.2Duplication | -0.675 | 0.264 | -2.552 | 0.011 | 0.043 | >30years |
| ISO.CGC.combat | Control (Ref) vs 22q11.2Duplication | -0.358 | 0.260 | -1.377 | 0.170 | 0.294 | >30years |
| ISO.CGH.combat | Control (Ref) vs 22q11.2Duplication | -0.276 | 0.252 | -1.094 | 0.275 | 0.422 | >30years |
| ISO.CR.combat | Control (Ref) vs 22q11.2Duplication | -1.098 | 0.258 | -4.259 | 0.000 | 0.001 | >30years |
| ISO.CST.combat | Control (Ref) vs 22q11.2Duplication | 0.192 | 0.229 | 0.837 | 0.403 | 0.542 | >30years |
| ISO.EC.combat | Control (Ref) vs 22q11.2Duplication | -0.211 | 0.264 | -0.801 | 0.424 | 0.555 | >30years |
| ISO.FX.combat | Control (Ref) vs 22q11.2Duplication | 0.130 | 0.262 | 0.495 | 0.621 | 0.725 | >30years |
| ISO.FXST.combat | Control (Ref) vs 22q11.2Duplication | -0.634 | 0.250 | -2.540 | 0.012 | 0.043 | >30years |
| ISO.GCC.combat | Control (Ref) vs 22q11.2Duplication | -0.605 | 0.266 | -2.272 | 0.024 | 0.073 | >30years |
| ISO.IC.combat | Control (Ref) vs 22q11.2Duplication | -0.555 | 0.246 | -2.261 | 0.025 | 0.073 | >30years |
| ISO.UNC.combat | Control (Ref) vs 22q11.2Duplication | -0.346 | 0.270 | -1.281 | 0.201 | 0.338 | >30years |
| ISO.PCR.combat | Control (Ref) vs 22q11.2Duplication | -0.962 | 0.257 | -3.746 | 0.000 | 0.003 | >30years |
| ISO.PLIC.combat | Control (Ref) vs 22q11.2Duplication | -0.230 | 0.259 | -0.888 | 0.375 | 0.521 | >30years |
| ISO.PTR.combat | Control (Ref) vs 22q11.2Duplication | -0.708 | 0.265 | -2.671 | 0.008 | 0.035 | >30years |
| ISO.RLIC.combat | Control (Ref) vs 22q11.2Duplication | -0.562 | 0.250 | -2.251 | 0.025 | 0.074 | >30years |
| ISO.SCC.combat | Control (Ref) vs 22q11.2Duplication | -0.408 | 0.268 | -1.521 | 0.130 | 0.248 | >30years |
| ISO.SCR.combat | Control (Ref) vs 22q11.2Duplication | -1.144 | 0.255 | -4.491 | 0.000 | 0.001 | >30years |
| ISO.SFO.combat | Control (Ref) vs 22q11.2Duplication | -0.629 | 0.257 | -2.452 | 0.015 | 0.053 | >30years |
| ISO.SLF.combat | Control (Ref) vs 22q11.2Duplication | -1.025 | 0.257 | -3.988 | 0.000 | 0.002 | >30years |
| ISO.SS.combat | Control (Ref) vs 22q11.2Duplication | -0.474 | 0.261 | -1.814 | 0.071 | 0.162 | >30years |
| ISO.TAP.combat | Control (Ref) vs 22q11.2Duplication | -0.626 | 0.265 | -2.361 | 0.019 | 0.063 | >30years |
| ODI.ACR.combat | Control (Ref) vs 22q11.2Duplication | -0.088 | 0.265 | -0.332 | 0.741 | 0.817 | >30years |
| ODI.ALIC.combat | Control (Ref) vs 22q11.2Duplication | 0.270 | 0.258 | 1.048 | 0.295 | 0.441 | >30years |
| ODI.BCC.combat | Control (Ref) vs 22q11.2Duplication | 0.135 | 0.263 | 0.513 | 0.609 | 0.719 | >30years |
| ODI.CC.combat | Control (Ref) vs 22q11.2Duplication | 0.032 | 0.262 | 0.122 | 0.903 | 0.937 | >30years |
| ODI.CGC.combat | Control (Ref) vs 22q11.2Duplication | 0.373 | 0.259 | 1.437 | 0.152 | 0.273 | >30years |
| ODI.CGH.combat | Control (Ref) vs 22q11.2Duplication | -0.134 | 0.260 | -0.515 | 0.607 | 0.719 | >30years |
| ODI.CR.combat | Control (Ref) vs 22q11.2Duplication | -0.325 | 0.261 | -1.244 | 0.215 | 0.350 | >30years |
| ODI.CST.combat | Control (Ref) vs 22q11.2Duplication | 0.353 | 0.262 | 1.346 | 0.180 | 0.304 | >30years |
| ODI.EC.combat | Control (Ref) vs 22q11.2Duplication | 0.144 | 0.253 | 0.568 | 0.571 | 0.685 | >30years |
| ODI.FX.combat | Control (Ref) vs 22q11.2Duplication | 0.271 | 0.245 | 1.109 | 0.269 | 0.417 | >30years |
| ODI.FXST.combat | Control (Ref) vs 22q11.2Duplication | 0.160 | 0.256 | 0.623 | 0.534 | 0.656 | >30years |
| ODI.GCC.combat | Control (Ref) vs 22q11.2Duplication | -0.150 | 0.261 | -0.577 | 0.565 | 0.680 | >30years |
| ODI.IC.combat | Control (Ref) vs 22q11.2Duplication | 0.295 | 0.257 | 1.145 | 0.253 | 0.396 | >30years |
| ODI.UNC.combat | Control (Ref) vs 22q11.2Duplication | 0.110 | 0.266 | 0.415 | 0.678 | 0.771 | >30years |
| ODI.PCR.combat | Control (Ref) vs 22q11.2Duplication | -0.672 | 0.261 | -2.578 | 0.011 | 0.041 | >30years |
| ODI.PLIC.combat | Control (Ref) vs 22q11.2Duplication | 0.429 | 0.257 | 1.673 | 0.096 | 0.198 | >30years |
| ODI.PTR.combat | Control (Ref) vs 22q11.2Duplication | -0.116 | 0.268 | -0.435 | 0.664 | 0.763 | >30years |
| ODI.RLIC.combat | Control (Ref) vs 22q11.2Duplication | 0.041 | 0.262 | 0.155 | 0.877 | 0.917 | >30years |
| ODI.SCC.combat | Control (Ref) vs 22q11.2Duplication | 0.081 | 0.265 | 0.307 | 0.759 | 0.826 | >30years |
| ODI.SCR.combat | Control (Ref) vs 22q11.2Duplication | -0.305 | 0.263 | -1.161 | 0.247 | 0.392 | >30years |
| ODI.SFO.combat | Control (Ref) vs 22q11.2Duplication | -0.030 | 0.262 | -0.114 | 0.910 | 0.938 | >30years |
| ODI.SLF.combat | Control (Ref) vs 22q11.2Duplication | -0.225 | 0.267 | -0.843 | 0.400 | 0.542 | >30years |
| ODI.SS.combat | Control (Ref) vs 22q11.2Duplication | -0.101 | 0.265 | -0.380 | 0.704 | 0.791 | >30years |
| ODI.TAP.combat | Control (Ref) vs 22q11.2Duplication | 0.116 | 0.270 | 0.431 | 0.667 | 0.764 | >30years |
| ICVF.combat | 22q11.2Deletion (Ref) vs 22q11.2Duplication | -1.102 | 0.166 | -6.658 | 0.000 | 0.002 | >30years |
| ISO.combat | 22q11.2Deletion (Ref) vs 22q11.2Duplication | -1.089 | 0.356 | -3.058 | 0.011 | 0.043 | >30years |
| ODI.combat | 22q11.2Deletion (Ref) vs 22q11.2Duplication | -0.198 | 0.385 | -0.514 | 0.617 | 0.724 | >30years |
| ICVF.ACR.combat | 22q11.2Deletion (Ref) vs 22q11.2Duplication | -1.072 | 0.217 | -4.931 | 0.000 | 0.005 | >30years |
| ICVF.ALIC.combat | 22q11.2Deletion (Ref) vs 22q11.2Duplication | -0.598 | 0.329 | -1.819 | 0.096 | 0.198 | >30years |
| ICVF.BCC.combat | 22q11.2Deletion (Ref) vs 22q11.2Duplication | -1.271 | 0.175 | -7.272 | 0.000 | 0.001 | >30years |
| ICVF.CC.combat | 22q11.2Deletion (Ref) vs 22q11.2Duplication | -1.268 | 0.197 | -6.448 | 0.000 | 0.002 | >30years |
| ICVF.CGC.combat | 22q11.2Deletion (Ref) vs 22q11.2Duplication | -1.057 | 0.205 | -5.152 | 0.000 | 0.004 | >30years |
| ICVF.CGH.combat | 22q11.2Deletion (Ref) vs 22q11.2Duplication | -0.418 | 0.324 | -1.289 | 0.225 | 0.361 | >30years |
| ICVF.CR.combat | 22q11.2Deletion (Ref) vs 22q11.2Duplication | -1.081 | 0.223 | -4.840 | 0.001 | 0.005 | >30years |
| ICVF.CST.combat | 22q11.2Deletion (Ref) vs 22q11.2Duplication | -0.833 | 0.376 | -2.216 | 0.047 | 0.116 | >30years |
| ICVF.EC.combat | 22q11.2Deletion (Ref) vs 22q11.2Duplication | -0.924 | 0.223 | -4.151 | 0.002 | 0.011 | >30years |
| ICVF.FX.combat | 22q11.2Deletion (Ref) vs 22q11.2Duplication | -0.383 | 0.347 | -1.106 | 0.291 | 0.437 | >30years |
| ICVF.FXST.combat | 22q11.2Deletion (Ref) vs 22q11.2Duplication | -0.831 | 0.338 | -2.460 | 0.031 | 0.087 | >30years |
| ICVF.GCC.combat | 22q11.2Deletion (Ref) vs 22q11.2Duplication | -1.187 | 0.209 | -5.670 | 0.000 | 0.003 | >30years |
| ICVF.IC.combat | 22q11.2Deletion (Ref) vs 22q11.2Duplication | -0.884 | 0.269 | -3.281 | 0.008 | 0.034 | >30years |
| ICVF.UNC.combat | 22q11.2Deletion (Ref) vs 22q11.2Duplication | -1.239 | 0.144 | -8.603 | 0.000 | 0.001 | >30years |
| ICVF.PCR.combat | 22q11.2Deletion (Ref) vs 22q11.2Duplication | -1.109 | 0.237 | -4.672 | 0.001 | 0.007 | >30years |
| ICVF.PLIC.combat | 22q11.2Deletion (Ref) vs 22q11.2Duplication | -0.878 | 0.273 | -3.222 | 0.009 | 0.036 | >30years |
| ICVF.PTR.combat | 22q11.2Deletion (Ref) vs 22q11.2Duplication | -1.305 | 0.241 | -5.419 | 0.000 | 0.003 | >30years |
| ICVF.RLIC.combat | 22q11.2Deletion (Ref) vs 22q11.2Duplication | -1.098 | 0.261 | -4.213 | 0.001 | 0.011 | >30years |
| ICVF.SCC.combat | 22q11.2Deletion (Ref) vs 22q11.2Duplication | -1.201 | 0.235 | -5.117 | 0.000 | 0.004 | >30years |
| ICVF.SCR.combat | 22q11.2Deletion (Ref) vs 22q11.2Duplication | -1.033 | 0.239 | -4.327 | 0.001 | 0.009 | >30years |
| ICVF.SFO.combat | 22q11.2Deletion (Ref) vs 22q11.2Duplication | -0.836 | 0.229 | -3.652 | 0.004 | 0.021 | >30years |
| ICVF.SLF.combat | 22q11.2Deletion (Ref) vs 22q11.2Duplication | -1.119 | 0.165 | -6.796 | 0.000 | 0.002 | >30years |
| ICVF.SS.combat | 22q11.2Deletion (Ref) vs 22q11.2Duplication | -1.153 | 0.224 | -5.153 | 0.000 | 0.004 | >30years |
| ICVF.TAP.combat | 22q11.2Deletion (Ref) vs 22q11.2Duplication | -1.236 | 0.272 | -4.538 | 0.001 | 0.007 | >30years |
| ISO.ACR.combat | 22q11.2Deletion (Ref) vs 22q11.2Duplication | -0.952 | 0.356 | -2.674 | 0.021 | 0.067 | >30years |
| ISO.ALIC.combat | 22q11.2Deletion (Ref) vs 22q11.2Duplication | -0.373 | 0.353 | -1.054 | 0.313 | 0.459 | >30years |
| ISO.BCC.combat | 22q11.2Deletion (Ref) vs 22q11.2Duplication | -0.707 | 0.377 | -1.874 | 0.086 | 0.185 | >30years |
| ISO.CC.combat | 22q11.2Deletion (Ref) vs 22q11.2Duplication | -0.571 | 0.341 | -1.674 | 0.122 | 0.240 | >30years |
| ISO.CGC.combat | 22q11.2Deletion (Ref) vs 22q11.2Duplication | -0.363 | 0.479 | -0.758 | 0.463 | 0.589 | >30years |
| ISO.CGH.combat | 22q11.2Deletion (Ref) vs 22q11.2Duplication | -0.919 | 0.370 | -2.481 | 0.030 | 0.084 | >30years |
| ISO.CR.combat | 22q11.2Deletion (Ref) vs 22q11.2Duplication | -1.055 | 0.328 | -3.213 | 0.008 | 0.034 | >30years |
| ISO.CST.combat | 22q11.2Deletion (Ref) vs 22q11.2Duplication | 0.687 | 0.349 | 1.968 | 0.073 | 0.162 | >30years |
| ISO.EC.combat | 22q11.2Deletion (Ref) vs 22q11.2Duplication | -0.621 | 0.283 | -2.190 | 0.049 | 0.120 | >30years |
| ISO.FX.combat | 22q11.2Deletion (Ref) vs 22q11.2Duplication | -0.208 | 0.406 | -0.512 | 0.618 | 0.724 | >30years |
| ISO.FXST.combat | 22q11.2Deletion (Ref) vs 22q11.2Duplication | -0.290 | 0.470 | -0.617 | 0.549 | 0.672 | >30years |
| ISO.GCC.combat | 22q11.2Deletion (Ref) vs 22q11.2Duplication | -0.414 | 0.399 | -1.037 | 0.321 | 0.465 | >30years |
| ISO.IC.combat | 22q11.2Deletion (Ref) vs 22q11.2Duplication | -0.721 | 0.268 | -2.690 | 0.021 | 0.067 | >30years |
| ISO.UNC.combat | 22q11.2Deletion (Ref) vs 22q11.2Duplication | -0.304 | 0.363 | -0.840 | 0.419 | 0.555 | >30years |
| ISO.PCR.combat | 22q11.2Deletion (Ref) vs 22q11.2Duplication | -1.057 | 0.331 | -3.190 | 0.008 | 0.034 | >30years |
| ISO.PLIC.combat | 22q11.2Deletion (Ref) vs 22q11.2Duplication | -0.500 | 0.310 | -1.612 | 0.136 | 0.251 | >30years |
| ISO.PTR.combat | 22q11.2Deletion (Ref) vs 22q11.2Duplication | -1.134 | 0.315 | -3.605 | 0.004 | 0.019 | >30years |
| ISO.RLIC.combat | 22q11.2Deletion (Ref) vs 22q11.2Duplication | -0.866 | 0.290 | -2.990 | 0.011 | 0.043 | >30years |
| ISO.SCC.combat | 22q11.2Deletion (Ref) vs 22q11.2Duplication | -0.278 | 0.257 | -1.085 | 0.314 | 0.459 | >30years |
| ISO.SCR.combat | 22q11.2Deletion (Ref) vs 22q11.2Duplication | -1.100 | 0.296 | -3.715 | 0.003 | 0.018 | >30years |
| ISO.SFO.combat | 22q11.2Deletion (Ref) vs 22q11.2Duplication | -0.825 | 0.363 | -2.273 | 0.044 | 0.112 | >30years |
| ISO.SLF.combat | 22q11.2Deletion (Ref) vs 22q11.2Duplication | -1.047 | 0.268 | -3.911 | 0.002 | 0.013 | >30years |
| ISO.SS.combat | 22q11.2Deletion (Ref) vs 22q11.2Duplication | -0.509 | 0.345 | -1.473 | 0.166 | 0.292 | >30years |
| ISO.TAP.combat | 22q11.2Deletion (Ref) vs 22q11.2Duplication | -0.522 | 0.199 | -2.627 | 0.032 | 0.088 | >30years |
| ODI.ACR.combat | 22q11.2Deletion (Ref) vs 22q11.2Duplication | -0.407 | 0.227 | -1.794 | 0.124 | 0.242 | >30years |
| ODI.ALIC.combat | 22q11.2Deletion (Ref) vs 22q11.2Duplication | 0.748 | 0.380 | 1.970 | 0.072 | 0.162 | >30years |
| ODI.BCC.combat | 22q11.2Deletion (Ref) vs 22q11.2Duplication | -0.324 | 0.373 | -0.868 | 0.402 | 0.542 | >30years |
| ODI.CC.combat | 22q11.2Deletion (Ref) vs 22q11.2Duplication | -0.421 | 0.370 | -1.138 | 0.277 | 0.422 | >30years |
| ODI.CGC.combat | 22q11.2Deletion (Ref) vs 22q11.2Duplication | 0.512 | 0.444 | 1.155 | 0.271 | 0.419 | >30years |
| ODI.CGH.combat | 22q11.2Deletion (Ref) vs 22q11.2Duplication | -1.476 | 0.393 | -3.759 | 0.006 | 0.030 | >30years |
| ODI.CR.combat | 22q11.2Deletion (Ref) vs 22q11.2Duplication | -0.459 | 0.258 | -1.779 | 0.123 | 0.242 | >30years |
| ODI.CST.combat | 22q11.2Deletion (Ref) vs 22q11.2Duplication | 0.339 | 0.420 | 0.807 | 0.435 | 0.568 | >30years |
| ODI.EC.combat | 22q11.2Deletion (Ref) vs 22q11.2Duplication | -0.554 | 0.384 | -1.443 | 0.175 | 0.299 | >30years |
| ODI.FX.combat | 22q11.2Deletion (Ref) vs 22q11.2Duplication | -0.152 | 0.447 | -0.341 | 0.739 | 0.817 | >30years |
| ODI.FXST.combat | 22q11.2Deletion (Ref) vs 22q11.2Duplication | -0.261 | 0.483 | -0.539 | 0.600 | 0.714 | >30years |
| ODI.GCC.combat | 22q11.2Deletion (Ref) vs 22q11.2Duplication | -0.468 | 0.359 | -1.301 | 0.218 | 0.353 | >30years |
| ODI.IC.combat | 22q11.2Deletion (Ref) vs 22q11.2Duplication | 0.404 | 0.443 | 0.910 | 0.381 | 0.526 | >30years |
| ODI.UNC.combat | 22q11.2Deletion (Ref) vs 22q11.2Duplication | 0.152 | 0.467 | 0.326 | 0.750 | 0.822 | >30years |
| ODI.PCR.combat | 22q11.2Deletion (Ref) vs 22q11.2Duplication | -0.995 | 0.320 | -3.111 | 0.009 | 0.038 | >30years |
| ODI.PLIC.combat | 22q11.2Deletion (Ref) vs 22q11.2Duplication | 0.691 | 0.428 | 1.616 | 0.132 | 0.251 | >30years |
| ODI.PTR.combat | 22q11.2Deletion (Ref) vs 22q11.2Duplication | -1.007 | 0.364 | -2.767 | 0.017 | 0.059 | >30years |
| ODI.RLIC.combat | 22q11.2Deletion (Ref) vs 22q11.2Duplication | -0.286 | 0.440 | -0.649 | 0.529 | 0.655 | >30years |
| ODI.SCC.combat | 22q11.2Deletion (Ref) vs 22q11.2Duplication | -0.463 | 0.383 | -1.207 | 0.251 | 0.396 | >30years |
| ODI.SCR.combat | 22q11.2Deletion (Ref) vs 22q11.2Duplication | -0.285 | 0.426 | -0.670 | 0.516 | 0.644 | >30years |
| ODI.SFO.combat | 22q11.2Deletion (Ref) vs 22q11.2Duplication | -0.545 | 0.398 | -1.368 | 0.196 | 0.331 | >30years |
| ODI.SLF.combat | 22q11.2Deletion (Ref) vs 22q11.2Duplication | -0.672 | 0.422 | -1.593 | 0.137 | 0.251 | >30years |
| ODI.SS.combat | 22q11.2Deletion (Ref) vs 22q11.2Duplication | -0.811 | 0.365 | -2.223 | 0.046 | 0.115 | >30years |
| ODI.TAP.combat | 22q11.2Deletion (Ref) vs 22q11.2Duplication | -0.004 | 0.119 | -0.038 | 0.972 | 0.977 | >30years |
| ICVF.combat | GeneDosage (1 = del, 2 = con, 3 dup) | -0.899 | 0.277 | -3.249 | 0.001 | 0.010 | >30years |
| ISO.combat | GeneDosage (1 = del, 2 = con, 3 dup) | -0.864 | 0.279 | -3.093 | 0.002 | 0.013 | >30years |
| ODI.combat | GeneDosage (1 = del, 2 = con, 3 dup) | -0.066 | 0.269 | -0.246 | 0.806 | 0.860 | >30years |
| ICVF.ACR.combat | GeneDosage (1 = del, 2 = con, 3 dup) | -0.889 | 0.278 | -3.198 | 0.002 | 0.011 | >30years |
| ICVF.ALIC.combat | GeneDosage (1 = del, 2 = con, 3 dup) | -0.392 | 0.262 | -1.493 | 0.137 | 0.251 | >30years |
| ICVF.BCC.combat | GeneDosage (1 = del, 2 = con, 3 dup) | -1.243 | 0.271 | -4.591 | 0.000 | 0.001 | >30years |
| ICVF.CC.combat | GeneDosage (1 = del, 2 = con, 3 dup) | -1.204 | 0.271 | -4.441 | 0.000 | 0.001 | >30years |
| ICVF.CGC.combat | GeneDosage (1 = del, 2 = con, 3 dup) | -0.710 | 0.276 | -2.578 | 0.010 | 0.041 | >30years |
| ICVF.CGH.combat | GeneDosage (1 = del, 2 = con, 3 dup) | -0.246 | 0.270 | -0.911 | 0.363 | 0.509 | >30years |
| ICVF.CR.combat | GeneDosage (1 = del, 2 = con, 3 dup) | -0.975 | 0.277 | -3.525 | 0.001 | 0.005 | >30years |
| ICVF.CST.combat | GeneDosage (1 = del, 2 = con, 3 dup) | -0.370 | 0.272 | -1.359 | 0.175 | 0.299 | >30years |
| ICVF.EC.combat | GeneDosage (1 = del, 2 = con, 3 dup) | -0.454 | 0.279 | -1.628 | 0.105 | 0.211 | >30years |
| ICVF.FX.combat | GeneDosage (1 = del, 2 = con, 3 dup) | -0.275 | 0.286 | -0.963 | 0.337 | 0.476 | >30years |
| ICVF.FXST.combat | GeneDosage (1 = del, 2 = con, 3 dup) | -0.414 | 0.277 | -1.493 | 0.137 | 0.251 | >30years |
| ICVF.GCC.combat | GeneDosage (1 = del, 2 = con, 3 dup) | -1.070 | 0.271 | -3.950 | 0.000 | 0.002 | >30years |
| ICVF.IC.combat | GeneDosage (1 = del, 2 = con, 3 dup) | -0.582 | 0.271 | -2.146 | 0.033 | 0.088 | >30years |
| ICVF.UNC.combat | GeneDosage (1 = del, 2 = con, 3 dup) | -0.918 | 0.281 | -3.269 | 0.001 | 0.009 | >30years |
| ICVF.PCR.combat | GeneDosage (1 = del, 2 = con, 3 dup) | -1.122 | 0.276 | -4.072 | 0.000 | 0.002 | >30years |
| ICVF.PLIC.combat | GeneDosage (1 = del, 2 = con, 3 dup) | -0.503 | 0.273 | -1.844 | 0.066 | 0.154 | >30years |
| ICVF.PTR.combat | GeneDosage (1 = del, 2 = con, 3 dup) | -1.071 | 0.276 | -3.878 | 0.000 | 0.002 | >30years |
| ICVF.RLIC.combat | GeneDosage (1 = del, 2 = con, 3 dup) | -0.762 | 0.279 | -2.732 | 0.007 | 0.031 | >30years |
| ICVF.SCC.combat | GeneDosage (1 = del, 2 = con, 3 dup) | -1.102 | 0.276 | -3.995 | 0.000 | 0.002 | >30years |
| ICVF.SCR.combat | GeneDosage (1 = del, 2 = con, 3 dup) | -0.905 | 0.277 | -3.270 | 0.001 | 0.009 | >30years |
| ICVF.SFO.combat | GeneDosage (1 = del, 2 = con, 3 dup) | -0.557 | 0.275 | -2.028 | 0.044 | 0.112 | >30years |
| ICVF.SLF.combat | GeneDosage (1 = del, 2 = con, 3 dup) | -0.929 | 0.278 | -3.341 | 0.001 | 0.008 | >30years |
| ICVF.SS.combat | GeneDosage (1 = del, 2 = con, 3 dup) | -0.838 | 0.282 | -2.976 | 0.003 | 0.018 | >30years |
| ICVF.TAP.combat | GeneDosage (1 = del, 2 = con, 3 dup) | -1.171 | 0.270 | -4.338 | 0.000 | 0.001 | >30years |
| ISO.ACR.combat | GeneDosage (1 = del, 2 = con, 3 dup) | -0.718 | 0.284 | -2.528 | 0.012 | 0.044 | >30years |
| ISO.ALIC.combat | GeneDosage (1 = del, 2 = con, 3 dup) | -0.373 | 0.271 | -1.375 | 0.170 | 0.294 | >30years |
| ISO.BCC.combat | GeneDosage (1 = del, 2 = con, 3 dup) | -0.507 | 0.282 | -1.795 | 0.074 | 0.163 | >30years |
| ISO.CC.combat | GeneDosage (1 = del, 2 = con, 3 dup) | -0.420 | 0.285 | -1.471 | 0.142 | 0.259 | >30years |
| ISO.CGC.combat | GeneDosage (1 = del, 2 = con, 3 dup) | -0.299 | 0.279 | -1.075 | 0.284 | 0.430 | >30years |
| ISO.CGH.combat | GeneDosage (1 = del, 2 = con, 3 dup) | -0.528 | 0.268 | -1.968 | 0.050 | 0.122 | >30years |
| ISO.CR.combat | GeneDosage (1 = del, 2 = con, 3 dup) | -0.891 | 0.281 | -3.172 | 0.002 | 0.011 | >30years |
| ISO.CST.combat | GeneDosage (1 = del, 2 = con, 3 dup) | 0.413 | 0.250 | 1.652 | 0.100 | 0.202 | >30years |
| ISO.EC.combat | GeneDosage (1 = del, 2 = con, 3 dup) | -0.218 | 0.283 | -0.769 | 0.443 | 0.575 | >30years |
| ISO.FX.combat | GeneDosage (1 = del, 2 = con, 3 dup) | -0.074 | 0.281 | -0.264 | 0.792 | 0.853 | >30years |
| ISO.FXST.combat | GeneDosage (1 = del, 2 = con, 3 dup) | -0.259 | 0.271 | -0.957 | 0.339 | 0.478 | >30years |
| ISO.GCC.combat | GeneDosage (1 = del, 2 = con, 3 dup) | -0.305 | 0.286 | -1.067 | 0.287 | 0.433 | >30years |
| ISO.IC.combat | GeneDosage (1 = del, 2 = con, 3 dup) | -0.612 | 0.268 | -2.284 | 0.023 | 0.072 | >30years |
| ISO.UNC.combat | GeneDosage (1 = del, 2 = con, 3 dup) | -0.169 | 0.287 | -0.589 | 0.557 | 0.676 | >30years |
| ISO.PCR.combat | GeneDosage (1 = del, 2 = con, 3 dup) | -0.796 | 0.280 | -2.844 | 0.005 | 0.023 | >30years |
| ISO.PLIC.combat | GeneDosage (1 = del, 2 = con, 3 dup) | -0.413 | 0.276 | -1.500 | 0.135 | 0.251 | >30years |
| ISO.PTR.combat | GeneDosage (1 = del, 2 = con, 3 dup) | -0.612 | 0.285 | -2.149 | 0.033 | 0.088 | >30years |
| ISO.RLIC.combat | GeneDosage (1 = del, 2 = con, 3 dup) | -0.603 | 0.273 | -2.206 | 0.028 | 0.080 | >30years |
| ISO.SCC.combat | GeneDosage (1 = del, 2 = con, 3 dup) | -0.219 | 0.288 | -0.760 | 0.448 | 0.577 | >30years |
| ISO.SCR.combat | GeneDosage (1 = del, 2 = con, 3 dup) | -1.012 | 0.277 | -3.656 | 0.000 | 0.004 | >30years |
| ISO.SFO.combat | GeneDosage (1 = del, 2 = con, 3 dup) | -0.572 | 0.278 | -2.056 | 0.041 | 0.106 | >30years |
| ISO.SLF.combat | GeneDosage (1 = del, 2 = con, 3 dup) | -0.798 | 0.280 | -2.854 | 0.005 | 0.023 | >30years |
| ISO.SS.combat | GeneDosage (1 = del, 2 = con, 3 dup) | -0.324 | 0.283 | -1.146 | 0.253 | 0.396 | >30years |
| ISO.TAP.combat | GeneDosage (1 = del, 2 = con, 3 dup) | -0.524 | 0.283 | -1.850 | 0.065 | 0.153 | >30years |
| ODI.ACR.combat | GeneDosage (1 = del, 2 = con, 3 dup) | -0.354 | 0.281 | -1.263 | 0.208 | 0.342 | >30years |
| ODI.ALIC.combat | GeneDosage (1 = del, 2 = con, 3 dup) | 0.521 | 0.276 | 1.886 | 0.060 | 0.145 | >30years |
| ODI.BCC.combat | GeneDosage (1 = del, 2 = con, 3 dup) | -0.123 | 0.282 | -0.437 | 0.662 | 0.763 | >30years |
| ODI.CC.combat | GeneDosage (1 = del, 2 = con, 3 dup) | -0.239 | 0.281 | -0.853 | 0.395 | 0.538 | >30years |
| ODI.CGC.combat | GeneDosage (1 = del, 2 = con, 3 dup) | 0.287 | 0.280 | 1.023 | 0.307 | 0.454 | >30years |
| ODI.CGH.combat | GeneDosage (1 = del, 2 = con, 3 dup) | -0.632 | 0.278 | -2.271 | 0.024 | 0.073 | >30years |
| ODI.CR.combat | GeneDosage (1 = del, 2 = con, 3 dup) | -0.517 | 0.278 | -1.862 | 0.064 | 0.150 | >30years |
| ODI.CST.combat | GeneDosage (1 = del, 2 = con, 3 dup) | 0.463 | 0.279 | 1.657 | 0.099 | 0.202 | >30years |
| ODI.EC.combat | GeneDosage (1 = del, 2 = con, 3 dup) | -0.268 | 0.272 | -0.987 | 0.325 | 0.468 | >30years |
| ODI.FX.combat | GeneDosage (1 = del, 2 = con, 3 dup) | -0.026 | 0.267 | -0.099 | 0.921 | 0.944 | >30years |
| ODI.FXST.combat | GeneDosage (1 = del, 2 = con, 3 dup) | -0.109 | 0.277 | -0.395 | 0.693 | 0.781 | >30years |
| ODI.GCC.combat | GeneDosage (1 = del, 2 = con, 3 dup) | -0.252 | 0.279 | -0.904 | 0.367 | 0.512 | >30years |
| ODI.IC.combat | GeneDosage (1 = del, 2 = con, 3 dup) | 0.349 | 0.276 | 1.264 | 0.207 | 0.342 | >30years |
| ODI.UNC.combat | GeneDosage (1 = del, 2 = con, 3 dup) | 0.082 | 0.285 | 0.288 | 0.773 | 0.837 | >30years |
| ODI.PCR.combat | GeneDosage (1 = del, 2 = con, 3 dup) | -0.994 | 0.277 | -3.592 | 0.000 | 0.004 | >30years |
| ODI.PLIC.combat | GeneDosage (1 = del, 2 = con, 3 dup) | 0.557 | 0.274 | 2.029 | 0.043 | 0.112 | >30years |
| ODI.PTR.combat | GeneDosage (1 = del, 2 = con, 3 dup) | -0.665 | 0.281 | -2.361 | 0.019 | 0.063 | >30years |
| ODI.RLIC.combat | GeneDosage (1 = del, 2 = con, 3 dup) | -0.122 | 0.281 | -0.435 | 0.664 | 0.763 | >30years |
| ODI.SCC.combat | GeneDosage (1 = del, 2 = con, 3 dup) | -0.273 | 0.283 | -0.963 | 0.337 | 0.476 | >30years |
| ODI.SCR.combat | GeneDosage (1 = del, 2 = con, 3 dup) | -0.271 | 0.282 | -0.963 | 0.336 | 0.476 | >30years |
| ODI.SFO.combat | GeneDosage (1 = del, 2 = con, 3 dup) | -0.225 | 0.280 | -0.803 | 0.423 | 0.555 | >30years |
| ODI.SLF.combat | GeneDosage (1 = del, 2 = con, 3 dup) | -0.508 | 0.283 | -1.795 | 0.074 | 0.163 | >30years |
| ODI.SS.combat | GeneDosage (1 = del, 2 = con, 3 dup) | -0.502 | 0.281 | -1.785 | 0.075 | 0.165 | >30years |
| ODI.TAP.combat | GeneDosage (1 = del, 2 = con, 3 dup) | 0.097 | 0.287 | 0.339 | 0.735 | 0.817 | >30years |
